# The CoREST repressor complex mediates phenotype switching and therapy resistance in melanoma

**DOI:** 10.1172/JCI171063

**Published:** 2024-02-01

**Authors:** Muzhou Wu, Ailish Hanly, Frederick Gibson, Robert Fisher, Samantha Rogers, Kihyun Park, Angelina Zuger, Kevin Kuang, Jay H. Kalin, Sarah Nocco, Matthew Cole, Amy Xiao, Filisia Agus, Adam Labadorf, Samuel Beck, Marianne Collard, Philip A. Cole, Rhoda M. Alani

**Affiliations:** 1 Department of Dermatology, Boston University Chobanian and Avedisian School of Medicine, Boston, Massachusetts, USA.; 2 Division of Genetics, Departments of Medicine and Biological Chemistry and Molecular Pharmacology, Harvard Medical School and Brigham and Women’s Hospital, Boston, Massachusetts, USA.; 3 Bioinformatics Program, Boston University, Boston, Massachusetts, USA.; 4 Department of Neurology, Boston University School of Medicine, Boston, Massachusetts, USA.

**Keywords:** Dermatology, Oncology, Drug therapy, Epigenetics, Melanoma

## Abstract

Virtually all patients with BRAF-mutant melanoma develop resistance to MAPK inhibitors largely through nonmutational events. Although the epigenetic landscape is shown to be altered in therapy-resistant melanomas and other cancers, a specific targetable epigenetic mechanism has not been validated. Here, we evaluated the corepressor for element 1–silencing transcription factor (CoREST) epigenetic repressor complex and the recently developed bivalent inhibitor corin within the context of melanoma phenotype plasticity and therapeutic resistance. We found that CoREST was a critical mediator of the major distinct melanoma phenotypes and that corin treatment of melanoma cells led to phenotype reprogramming. Global assessment of transcript and chromatin changes conferred by corin revealed specific effects on histone marks connected to epithelial-mesenchymal transition–associated (EMT-associated) transcription factors and the dual-specificity phosphatases (DUSPs). Remarkably, treatment of BRAF inhibitor–resistant (BRAFi-R) melanomas with corin promoted resensitization to BRAFi therapy. DUSP1 was consistently downregulated in BRAFi-R melanomas, which was reversed by corin treatment and associated with inhibition of p38 MAPK activity and resensitization to BRAFi therapies. Moreover, this activity was recapitulated by the p38 MAPK inhibitor BIRB 796. These findings identify the CoREST repressor complex as a central mediator of melanoma phenotype plasticity and resistance to targeted therapy and suggest that CoREST inhibitors may prove beneficial for patients with BRAFi-resistant melanoma.

## Abstract 2

The CoREST epigenetic repressor complex has a role in melanoma resistance to targeted therapy that can be overcome in models by a CoREST inhibitor.

## Graphical abstract

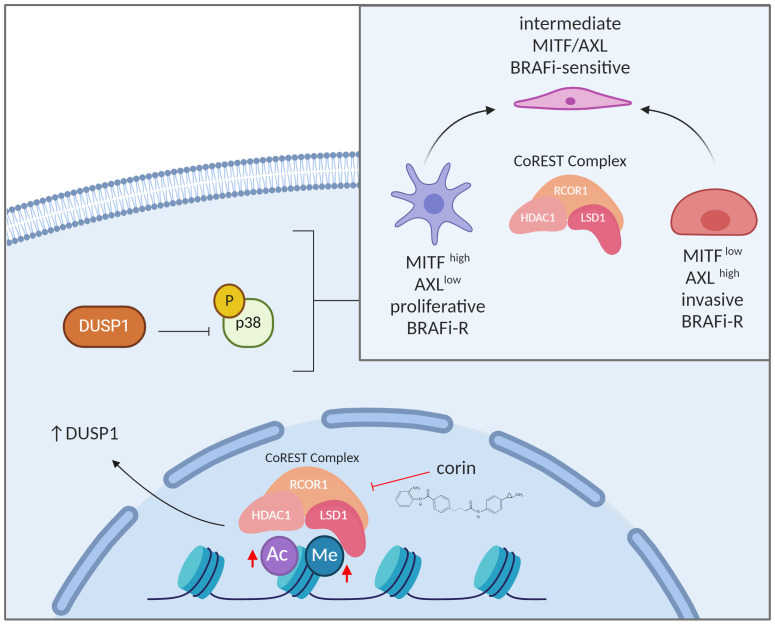

## Introduction

Melanomas exhibit tremendous intratumoral heterogeneity and phenotype plasticity, which allows them to switch between distinctive transcriptional programs in response to external stressors, including targeted therapies (1). These transcriptional phenotypes, which are mediated largely through epigenetic mechanisms (1, 2), are characterized by altered differentiation and metabolic states including a proliferative/differentiated/MITF^hi^/AXL^lo^ phenotype and an undifferentiated/invasive/MITF^lo^/AXL^hi^ phenotype (3, 4) with associated changes in the epigenetic landscape (5, 6). Moreover, cellular plasticity is a driver of resistance to targeted therapies in melanoma and other cancers, with dynamic transitions between distinctive molecular phenotypes promoting MAPK inhibitor (MAPKi) bypass mechanisms (7). As reversible transcriptional reprogramming dictates the plasticity of molecular phenotypes, research efforts have focused on the role of epigenetic regulation in this process (8–10).

The corepressor for element 1–silencing transcription factor (CoREST) epigenetic repressor complex is a member of the class I histone deacetylase family of repressor complexes that was originally identified as a cofactor for REST repression (11) and regulation of neuron-specific gene silencing during development (12), but more recently has been connected with the snail family of transcription factors (13). RE1-silencing transcription factor (REST) corepressor 1 (RCOR1) functions as a scaffold for the CoREST repressor complex promoting crosstalk between histone deacetylases 1 and 2 (HDAC1/2) and lysine demethylase 1A (LSD1), an H3K4 demethylase (14), and has been shown to regulate Treg function and antitumor immunity (15). We have recently described corin, a potent and specific dual-warhead inhibitor of the CoREST complex targeting HDAC1/2 and LSD1, that demonstrates growth inhibition in melanoma (16), cutaneous squamous cell carcinoma (16), breast cancers (17), and diffuse intrinsic pontine glioma (18). We therefore hypothesized that CoREST inhibition may elicit synergistic growth inhibition with BRAF inhibitor (BRAFi) therapies through epigenetic reprogramming of BRAF-mutant melanoma.

Here, we show that CoREST inhibition in human melanoma cell lines reversed the 2 major melanoma cell phenotypes, those characterized as either MITF^hi^/AXL^lo^ or MITF^lo^/AXL^hi^, and resensitized BRAFi-resistant (BRAFi-R) melanoma cells to BRAFi therapy. Additionally, we explored the transcriptomic and epigenomic landscapes regulated by CoREST inhibition and found specific alteration of epithelial-mesenchymal transition–associated (EMT-associated) transcription factors following corin treatment of BRAFi-R melanomas in addition to upregulation of the dual-specificity phosphatases and downstream inhibition of p38 MAPK activity. We further noted specific reactivation of BRAFi sensitivity in BRAFi-R melanoma cells following treatment with the p38 inhibitor BIRB 796, suggesting a specific mechanism of action for corin-associated resensitization of BRAFi-R melanomas to BRAFi therapies in this setting. In vivo studies demonstrate enhanced inhibition of BRAFi-R melanoma growth following treatment with the combination of corin plus BRAFi versus corin alone, further supporting a role for CoREST inhibition in resensitizing BRAFi-R melanomas to BRAF therapies and suggesting a potential role for CoREST inhibition as a therapeutic modality to enhance MAPK-targeted therapies in patients with advanced melanoma.

## Results

### The CoREST repressor complex mediates phenotype switching in melanoma.

**Figure 1:**
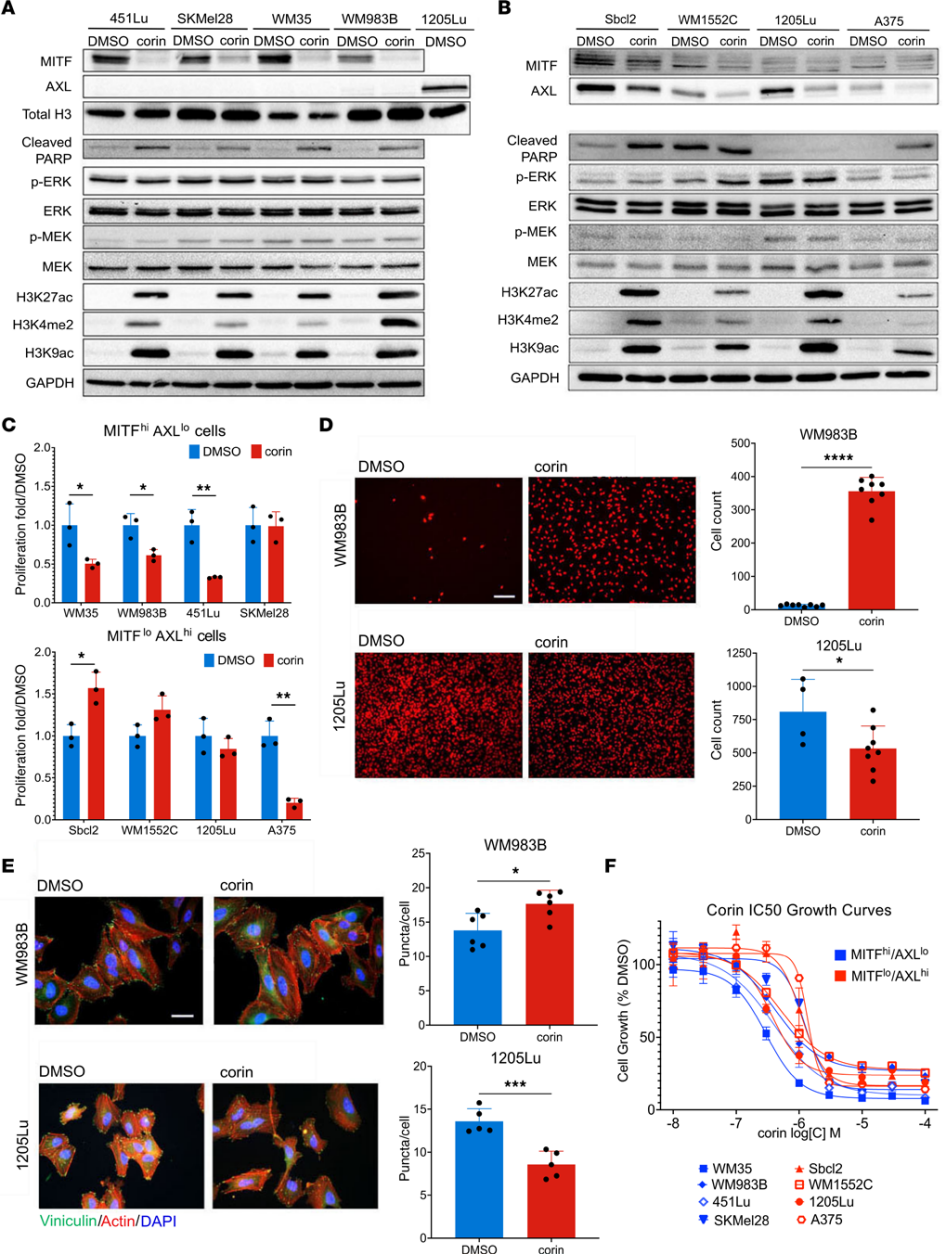
The CoREST repressor complex mediates phenotype switching in melanoma cell lines. (**A** and **B**) Western blot analysis of the MITF^hi^/AXL^lo^ melanoma cell lines 451Lu, SKMel28, WM35, and WM983B (**A**) and the MITF^lo^/AXL^hi^ melanoma cell lines Sbcl2, WM1552C, 1205Lu, and A375 (**B**) following 24 hours of treatment with DMSO or 2.5 μM corin. 1205Lu lysates were used as a positive control for AXL in **A**. Western blots were run contemporaneously. (**C**) Cellular proliferation of MITF^hi^/AXL^lo^ (upper panel) and MITF^lo^/AXL^hi^ (lower panel) melanoma cell lines following 24 hours of treatment with DMSO or 2.5 μM corin (*n* = 3). (**D**) Invasion assay and quantification of MITF^hi^/AXL^lo^ (WM983B, upper panel) and MITF^lo^/AXL^hi^ (1205Lu, lower panel) melanoma cells following 24 hours treatment with DMSO or 2.5 μM corin (*n* = 4–8). Representative images are shown. Scale bar: 100 μm. (**E**) Vinculin staining of focal adhesions in MITF^hi^/AXL^lo^ melanoma cells (WM983B, upper panel) and MITF^lo^/AXL^hi^ melanoma cells (1205Lu, lower panel) following a 24-hour treatment with DMSO or 2.5 μM corin (*n* = 5–6), with quantification of focal adhesions (puncta/cell) on the right (*n* = 5–6). Representative images are shown. Scale bar: 20 μm. (**F**) Dose-response proliferation assays of MITF^hi^/AXL^lo^ (WM35, WM983B, 451Lu, SkMel28) and MITF^lo^/AXL^hi^ (Sbcl2, WM1552C, 1205Lu, A375) melanoma cell lines treated with increasing doses of corin for 72 hours. *P < 0.05, **P < 0.01, ***P < 0.001, and ****P < 0.0001, by 2-tailed, unpaired *t* test compared with DMSO controls.

**Figure 2:**
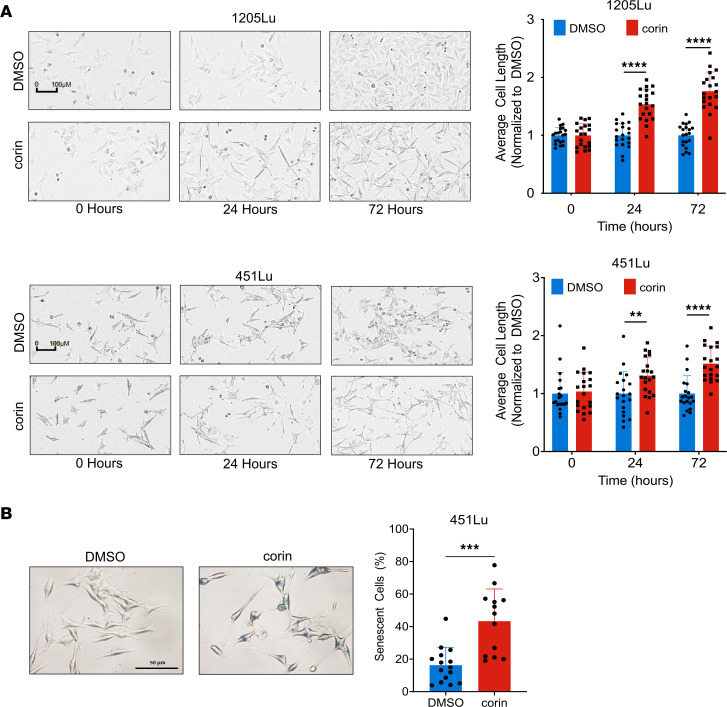
CoREST inhibition induces morphological changes in melanoma cells. (**A**) Average cell length for MITF^hi^/AXL^lo^ 451Lu and MITF^lo^/AXL^hi^ 1205Lu melanoma cells treated with 1 μM corin for 0, 24, and 72 hours (*n* = 20). Representative images shown. Scale bars: 100 μm. (**B**) Senescence-associated β-galactosidase staining of 451Lu melanoma cells treated with DMSO or 1 μM corin for 72 hours and quantification. Scale bar: 50 μm. **P < 0.01, ***P < 0.001, and ****P < 0.0001, by 2-tailed, unpaired *t* test compared with DMSO controls.

In order to further explore the role of the CoREST repressor complex in human melanoma development, we treated a panel of phenotypically distinct melanoma cell lines with the CoREST inhibitor corin for 24 hours (Figure 1). Remarkably, all tumor cell lines demonstrating an MITF^hi^/AXL^lo^ melanoma phenotype showed decreased expression of MITF and increased expression of histone H3K4me2, a common readout of LSD1 inhibition (19, 20), and H3K9ac/K27ac marks following corin treatment, without significant effects on the MEK/ERK pathway (Figure 1A). In addition, corin treatment of MITF^lo^/AXL^hi^ melanoma cells led to decreased AXL expression and increased histone acetylation and methylation marks in all cell lines evaluated, without affecting the MEK/ERK pathway (Figure 1B). These data suggest a specific reversion of melanoma differentiation phenotypes to intermediate, non-MITF^hi^, and non-AXL^hi^ cellular programs following corin treatment. Moreover, treatment of melanoma cells for 24 hours with corin inhibited tumor cell growth in the majority of MITF^hi^/AXL^lo^ melanoma cells evaluated; however, minimal growth inhibition and even enhanced cellular proliferation was seen in MITF^lo^/AXL^hi^ cells following 24 hours of treatment with corin (Figure 1C), consistent with conversion of both distinctive melanoma cell line phenotypes to intermediate proliferation states following early corin treatment. Corin treatment also led to increased cellular invasion and expression of focal adhesions in MITF^hi^/AXL^lo^ cells, while decreasing invasion and expression of focal adhesions in MITF^lo^/AXL^hi^ cells (Figure 1, D and E). Notably, extended corin treatment of all melanoma cell lines (72 hours) led to substantial**)** growth inhibition with IC_50_ values consistently in the submicromolar range (Figure 1F, Table 1, Supplemental Figure 1, A–C, and Supplemental Table 1; supplemental material available online with this article; https://doi.org/10.1172/JCI171063DS1). We also observed morphological changes following corin treatment, with both 1205Lu and 451Lu cells displaying an elongated morphology (Figure 2A). Notably, corin-treated MITF^lo^/AXL^hi^ (1205Lu) cells demonstrated a more melanocytic phenotype compared with the DMSO control–treated cells, whereas MITF^hi^/AXL^lo^ (451Lu) cells had a more senescent cellular phenotype, which was confirmed by β-galactosidase staining (Figure 2B).

### CoREST inhibition resensitizes BRAFi-R melanoma cells to BRAFi therapy.

**Figure 3:**
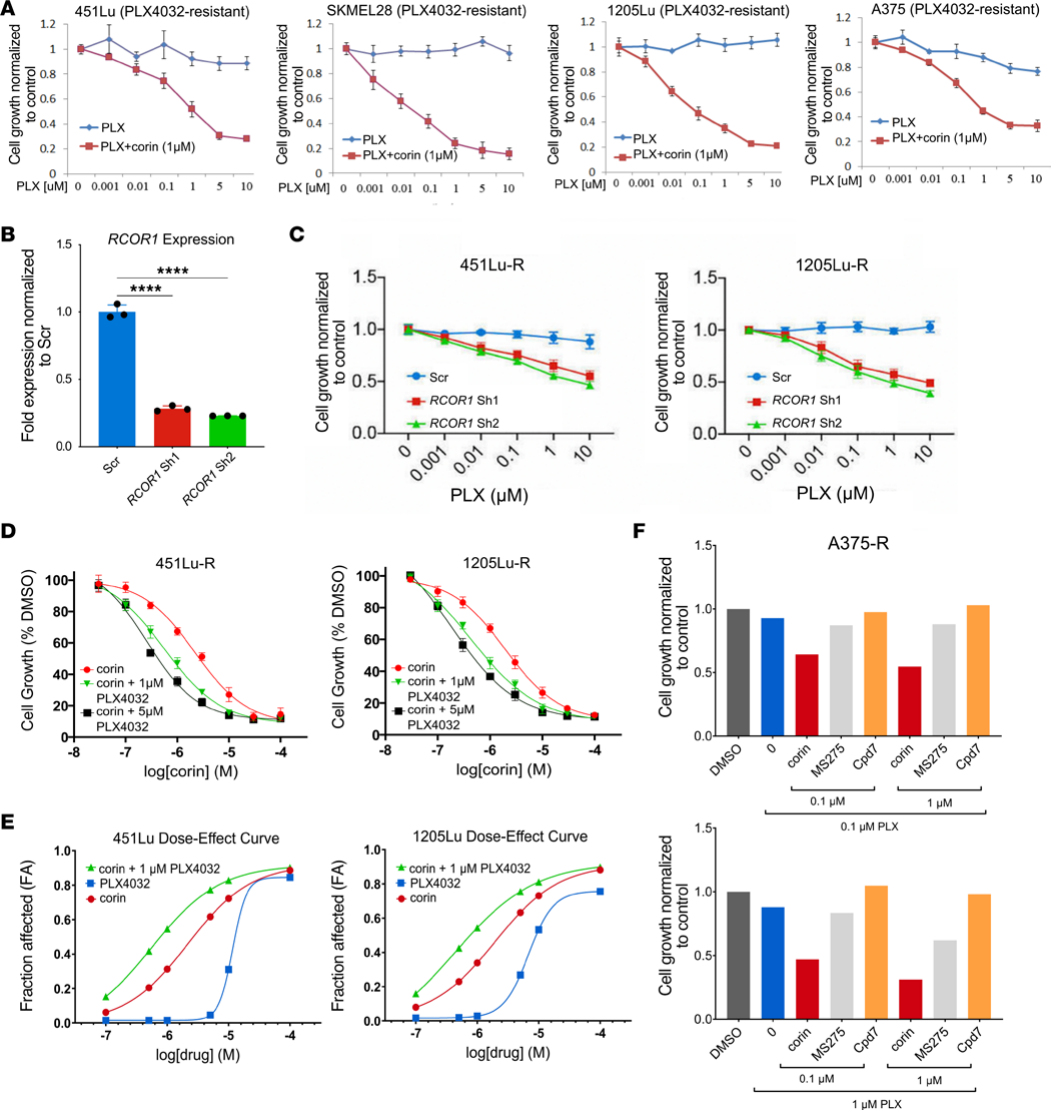
CoREST inhibition resensitizes BRAFi-R melanoma cells to BRAFi therapy. (**A**) Proliferation assays of 451Lu-R and SkMel28-R (MITF^hi^/AXL^lo^) and 1205Lu-R and A375-R (MITF^lo^/AXL^hi^) BRAFi-R melanoma cell lines treated with increasing doses of PLX4032 with or without 1 μM corin for 72 hours (*n* = 3). (**B**) *RCOR1* knockdown by shRNA in 1205Lu-R melanoma cells, confirmed by RT-qPCR (data are representative of 2 independent experiments). (**C**) PLX4032 (PLX) dose-response curves (72-hour treatment) in 451Lu-R and 1205Lu-R melanoma cells following knockdown of *RCOR*1 (*n* = 3). (**D**) Proliferation assays of 451Lu-R and 1205Lu-R BRAFi-R melanoma cell lines treated with increasing doses of corin with or without 1 μM or 5 μM PLX4032 for 72 hours (*n* = 3). (**E**) Drug synergy graphs for corin and PLX4032 in 451Lu-R (left) and 1205Lu-R (right) BRAFi-R melanoma lines. (**F**) Proliferation assays of A375-R melanoma cells treated with 0.1 μM (top panel) or 1 μM (bottom panel) PLX4032 with or without corin, MS275, or compound 7 (Cpd7) at 0.1 μM and 1 μM for 72 hours (*n* = 1).

**Figure 4:**
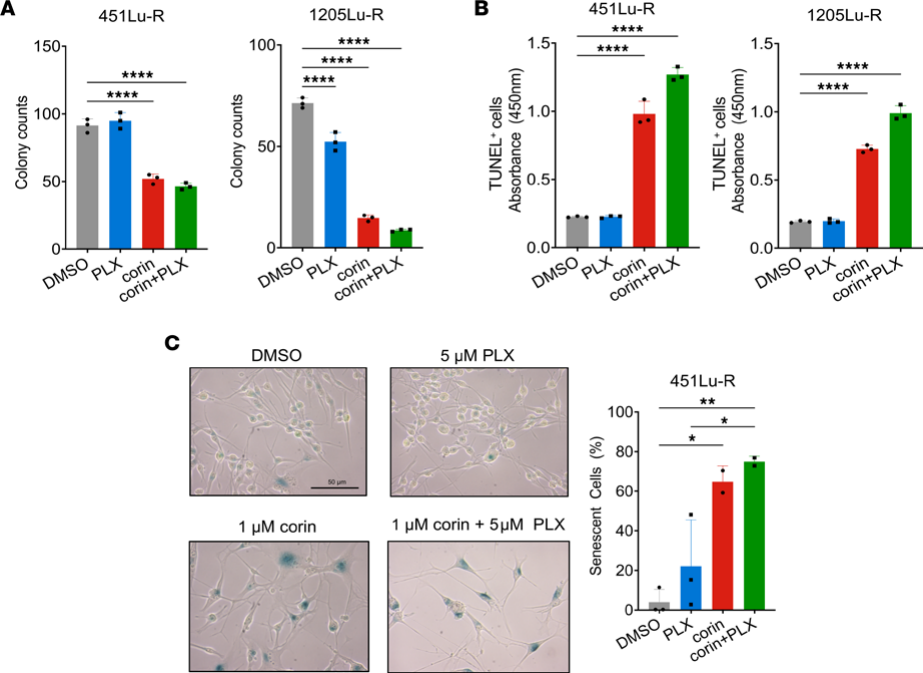
CoREST inhibition reduces colony formation and induces apoptosis in BRAFi-R melanoma cells with or without BRAFi. (**A**) Colony formation assay quantification in 451Lu-R (left) and 1205Lu-R (right) melanoma cells treated with DMSO, 5 μM PLX4032 alone, 2.5 μM corin alone, or 2.5 μM corin plus 5 μM PLX4032 for 10 days (*n* = 3). (**B**) Quantification of TUNEL^+^ cells in 451Lu-R and 1205Lu-R melanoma cells following 72 hours of treatment with DMSO, 5 μM PLX4032 alone, 1 μM corin alone, or 1 μM corin plus 5 μM PLX4032 (*n* = 3). (**C**) Senescence-associated β-galactosidase staining of 451Lu-R melanoma cells treated with DMSO, 5 μM PLX4032 alone, 1 μM corin alone, or 1 μM corin plus 5 μM PLX4032 for 72 hours and quantification (*n* = 2–3). Scale bar: 50 μm. *P < 0.05, **P < 0.01, and ****P < 0.0001, by 1-way ANOVA with Tukey’s test.

As tumor cell plasticity and phenotype switching is associated with resistance to targeted therapies in melanoma and other cancers, and intermediate phenotypes are generally considered to be treatment sensitive (21, 22), we next investigated the effect of CoREST inhibition combined with the BRAFi PLX4032 (vemurafenib, referred to herein as PLX4032) on BRAFi-R melanoma cell proliferation. Notably, we found that corin markedly increased the antiproliferative effects of PLX4032 in all BRAFi-R melanoma cells evaluated (Figure 3A). In addition, silencing of the CoREST scaffolding protein RCOR1 also led to resensitization of BRAFi-R MITF^hi^/AXL^lo^ and MITF^lo^/AXL^hi^ melanoma cells to PLX4032 (Figure 3, B and C). Interestingly, we also found that PLX4032 enhanced the antiproliferative effects of corin treatment alone in both BRAFi-R MITF^hi^/AXL^lo^ and MITF^lo^/AXL^hi^ melanoma cells (Figure 3D), with synergy identified between PLX4032 and corin in MITF^hi^/AXL^lo^ and MITF^lo^/AXL^hi^ melanoma cells (combination index [CI] <1.0 [ref. 23]) (Figure 3E, Table 2, Supplemental Figure 1D, and Supplemental Table 2). Of note, growth inhibition in BRAFi-R melanoma cells treated with hi-dose or low-dose PLX4032 was increased following corin treatment versus treatment with the LSD1 inhibitor Cpd7 or the HDAC1 inhibitor MS275 alone, suggesting specific synergies with PXL4032 activity relevant to targeting of the CoREST complex (Figure 3F). In addition, corin treatment significantly reduced colony formation and increased apoptosis in BRAFi-R melanoma cells (Figure 4, A and B, Supplemental Figure 1, E and F), which was further enhanced in combination with PLX4032 (Figure 4, A and B). While the synergy between corin and PLX was not as evident in the colony formation assays compared with the proliferation assays, this may reflect differences between corin’s efficacy in bulk cell proliferation compared with single-cell clonogenic potential. Corin treatment also induced cellular senescence in BRAFi-R MITF^hi^/AXL^lo^ melanoma cells (Figure 4C) but not in MITF^lo^/AXL^hi^ melanoma cells, without obvious effects on autophagy (Supplemental Figure 1, G–I).

### Inhibition of the CoREST complex in BRAFi-R melanoma promotes transcriptional changes associated with the phenotype switch and increased expression of DUSP family MAPK inhibitors.

**Figure 5:**
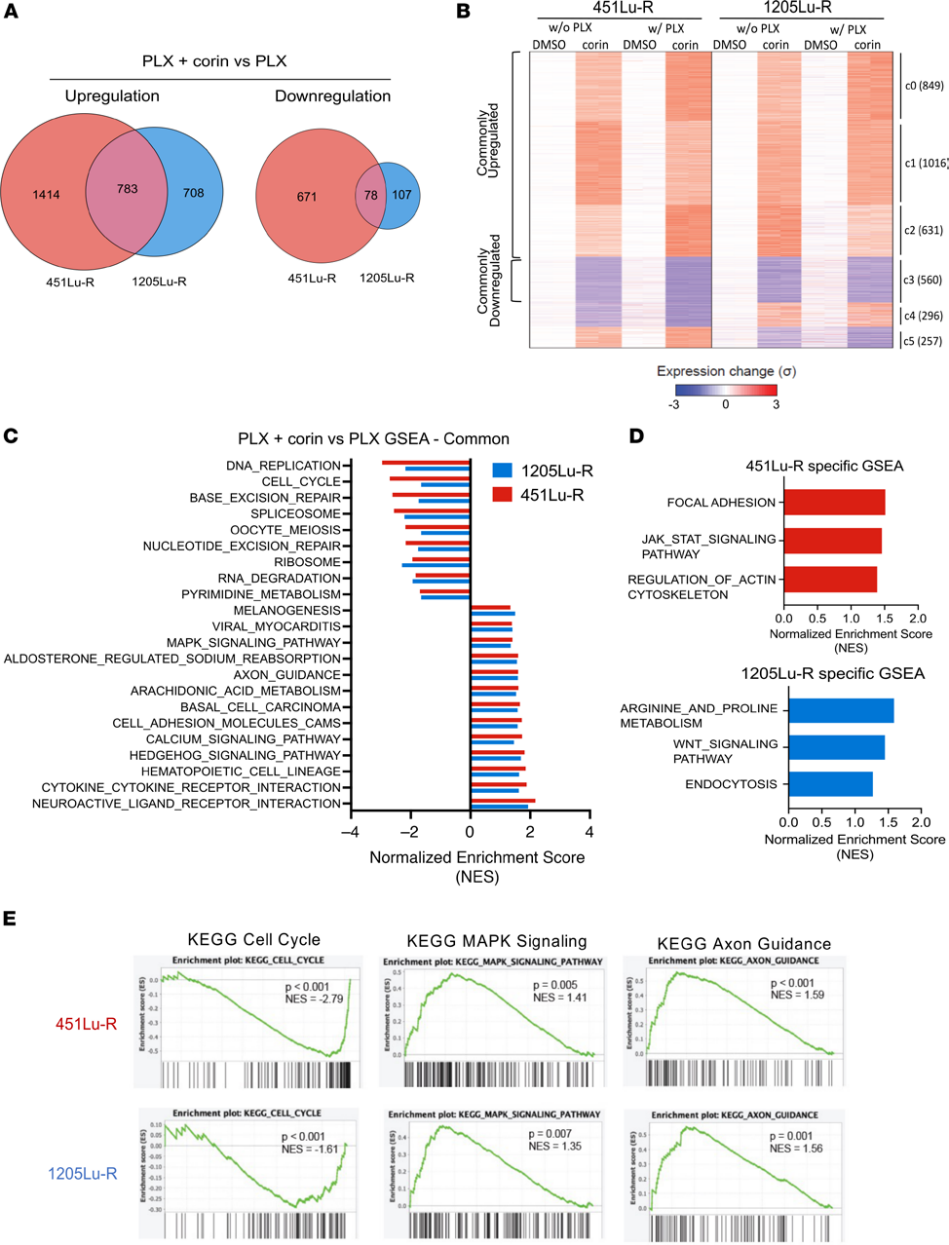
CoREST complex inhibition in BRAFi-R melanoma cells induces global transcriptional upregulation and causes changes in cell-cycle, MAPK, and axon guidance signaling pathways. (**A**) RNA-Seq profiling of 451Lu-R and 1205Lu-R melanoma cells following 24 hours of treatment with 5 μM PLX4032 versus 2.5 μM corin plus 5 μM PLX4032. Venn diagram illustrates corin plus PLX4032–induced upregulated and downregulated expression of cell line–specific and common genes compared with PLX4032 treatment alone (fold change [FC] ≥4, FDR <0.001). (**B**) K-means clustered (K = 6) heatmap of all genes showing significant expression changes (*P*adj < 0.01, |log_2_ FC| >2) upon corin treatment in at least 1 condition/cell line (i.e., with or without [w/o] PLX4032; 451Lu-R or 1205Lu-R). In each cell line, expression in the absence of PLX4032 and corin was set as reference (i.e., 0), and the relative expression changes were summarized as SD (σ). Numbers in the brackets indicate the gene counts in clusters. (**C**) GSEA of corin-induced common enriched pathways in 451Lu-R and 1205Lu-R melanoma cells. All gene sets displayed are significantly (*P* < 0.05) enriched with corin plus PLX4032 versus PLX4032 treatment alone. (**D**) GSEA of corin-induced distinct enriched pathways in 451Lu-R (top) and 1205Lu-R (bottom) melanoma cells. All gene sets displayed are significantly (*P* < 0.05) enriched with corin plus PLX4032 versus PLX4032 treatment alone. (**E**) GSEA in samples treated with corin plus PLX4032 versus those treated with PLX4032 alone illustrating representative common enriched gene sets in 451Lu-R (top) and 1205Lu-R (bottom) melanoma cells. NES, normalized enrichment score. *n* = 2 for all panels in this figure.

In order to investigate mechanisms of corin resensitization of BRAFi-R melanoma cells to PLX4032, RNA-Seq was performed on 451Lu-R (MITF^hi^/AXL^lo^) and 1205Lu-R (MITF^lo^/AXL^hi^) cells treated with PLX4032 alone, corin alone, or corin in combination with PLX4032 for 24 hours. Differentially regulated genes were identified by comparison with PLX4032-treated controls (Figure 5A). Not surprisingly, we found that corin treatment led to substantially greater numbers of upregulated genes than downregulated genes, consistent with the repressive functions of the CoREST complex. K-means clustering of differentially regulated genes revealed common corin-affected gene clusters as well as cell line–specific, corin-affected gene clusters (Figure 5B), which were characterized ontologically (Supplemental Figure 2, A and B). Although ontological analysis paired with gene set enrichment analysis (GSEA) using the Kyoto Encyclopedia of Genes and Genomes (KEGG) pathway gene sets revealed common corin-regulated gene sets (Figure 5C), we also identified distinct pathways specific to each cell line (Figure 5D). Cell-cycle and DNA repair–related gene sets were commonly downregulated in both cell lines with corin treatment (Figure 5, C and E, and Supplemental Figure 2A), confirmed by real-time quantitative PCR (RT-qPCR) (Supplemental Figure 2, C and D), while MAPK signaling, cell adhesion molecules, axon guidance, hedgehog signaling, and neuronal pathways were commonly upregulated with corin treatment (Figure 5, C and E and Supplemental Figure 2A). Differential expression of key genes involved in focal adhesion, axon guidance, and EMT were confirmed by RT-qPCR (Supplemental Figure 2, E and F and Supplemental Figure 3, A–C), suggesting a cellular phenotype switch similar to what was observed in the BRAFi-sensitive (BRAFi-S) melanoma cells treated with corin.

**Figure 6:**
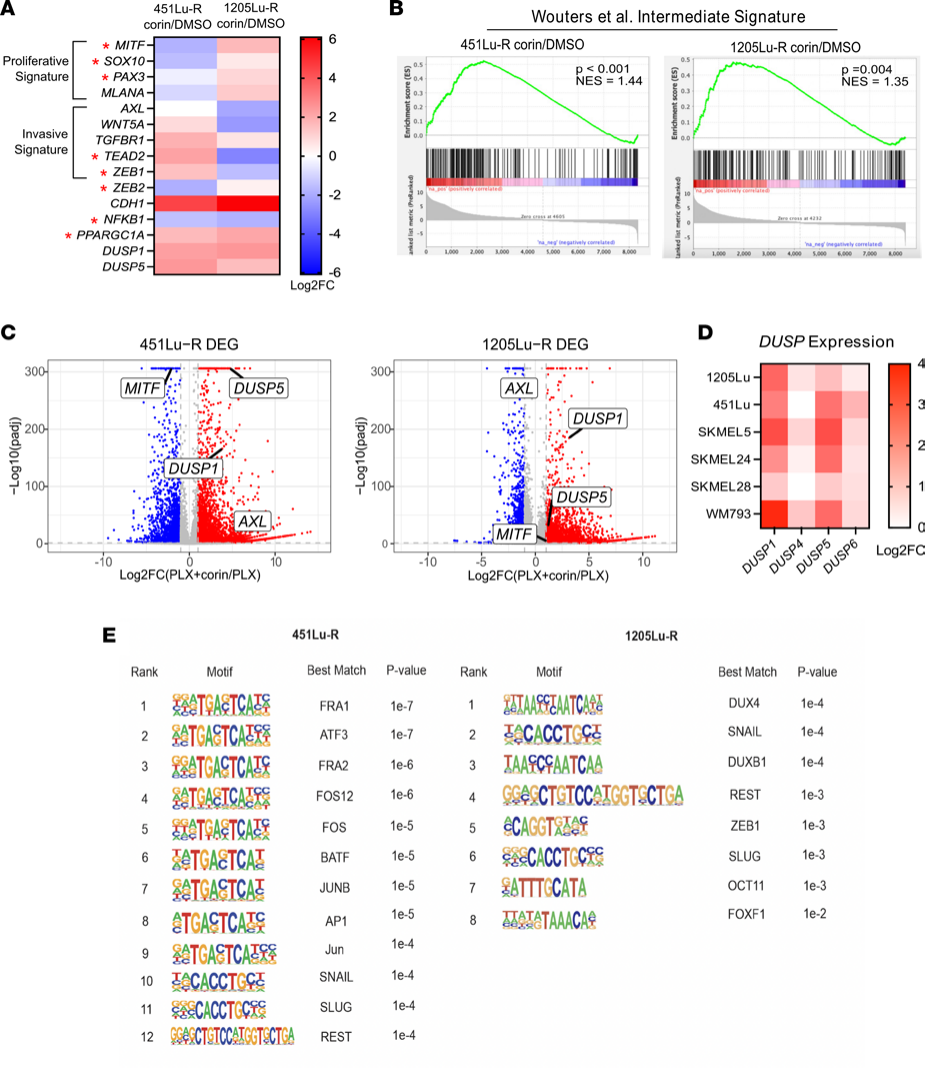
Inhibition of the CoREST complex in BRAFi-R melanoma cells promotes transcriptional changes associated with the phenotype switch and increased expression of DUSP family MAPK inhibitors. (**A**) Heatmap of differential expression patterns of proliferative versus invasive gene signatures and transcriptional regulators associated with distinct melanoma phenotypes^22^ (indicated by a red asterisk) in 451Lu-R and 1205Lu-R melanoma cells treated with 2.5 μM corin or DMSO for 24 hours. (**B**) Comparison of the corin-associated intermediate phenotype gene expression signature in 451Lu-R and 1205Lu-R melanoma cells treated with 2.5 μM corin or DMSO for 24 hours and the published intermediate phenotype defined by Wouters et al. (26). (**C**) Volcano plots of differentially expressed genes (DEGs) (log_2_ FC >1, *P*adj < 0.01) in 451Lu-R (left) and 1205Lu-R (right) melanoma cells following 24 hours of treatment with 2.5 μM corin plus 5 μM PLX4032 versus 5 μM PLX4032 alone with highlighted changes in *DUSP1*, *DUSP5*, *MITF*, and *AXL* expression. (**D**) Heatmap of *DUSP1/-4/-5/-6* expression in corin-treated BRAFi-S melanoma cells relative to DMSO treatment (2.5 μM, 24 hours). (**E**) Top known transcription factor–binding motifs enriched in corin-upregulated genes in 451Lu-R (left) and 1205Lu-R (right) melanoma cells.

Comparison of corin-associated RNA-Seq data with publicly available data sets (3, 24, 25) supported a phenotype switch signature following corin treatment in both 451Lu-R and 1205Lu-R cells; genes associated with the corin-induced phenotype switch included *AXL, MITF, SOX10, WNT5A, PAX3, ZEB1, ZEB2, PGC1a*, *DUSP1*, and *DUSP5* (Figure 6A) and were significantly associated with the intermediate melanoma phenotype signature recently reported by Wouters et al. (26) (Figure 6B and Supplemental Figure 3D). Consistent with RNA-Seq results, Western blot analysis of BRAFi-R cell lines treated with corin showed reduced MITF protein expression in MITF^hi^/AXL^lo^ cells and reduced AXL protein expression in MITF^lo^/AXL^hi^ cells (Supplemental Figure 4, A and B). Of note, the dual-specificity phosphatases *DUSP1* and *DUSP5* were among the genes whose expression was most highly upregulated in the MAPK signaling GSEA and significantly (adjusted *P* [*P*adj] < 1 × 10^–31^ ) upregulated following corin treatment in both BRAFi-R cell lines (Figure 6C) as well as additional melanoma cell lines (Figure 6D). Corin treatment of 451Lu-R and 1205Lu-R melanoma cells also led to increased expression of type 1 and type 2 IFN response genes and repetitive elements (Supplemental Figure 5 and Supplemental Tables 3–6) and decreased expression of RNA-induced silencing complex (RISC) components (Supplemental Table 7), similar to previously reported changes associated with LSD1 ablation in tumor cells (27). Interestingly, Hugo et al. (28) previously noted genes with loss-of-function gene-based events (including transcriptional downregulation) in tumors from patients following acquired MAPKi resistance, and this gene signature was positively enriched with corin plus PLX4032 treatment of 451Lu-R and 1205Lu-R cells compared with PLX4032 alone, with several of the corin-upregulated genes confirmed by RT-qPCR, suggesting a specific effect on tumor-associated MAPKi resistance pathways (Supplemental Figure 6, A–C and Supplemental Table 8). Expression of the core components of the CoREST complex did not significantly change during acquired MAPKi resistance (Supplemental Figure 6D) (28).

Analysis of consensus transcription factor binding sites associated with corin-induced transcriptomic changes in 451Lu-R and 1205Lu-R melanoma cells revealed significant increases in EMT-associated transcription factor motifs in both cell lines as well as increases in AP-1 transcription factor motifs (Figure 6E), supporting the specific relevance of corin effects on melanoma cell plasticity and phenotype switching. Analysis of the top de novo motifs enriched in corin-upregulated genes in 451Lu-R and 1205Lu-R cells included those associated with cellular differentiation, tumor suppression, tumor phenotype switching, and repression of transposable elements (Supplemental Figure 7A).

### ChIP-Seq reveals global alterations in H3K27ac-,H3K9ac-, and H3K4me2-modified chromatin associated with the phenotype switch in BRAFi-R cells treated with corin.

**Figure 7:**
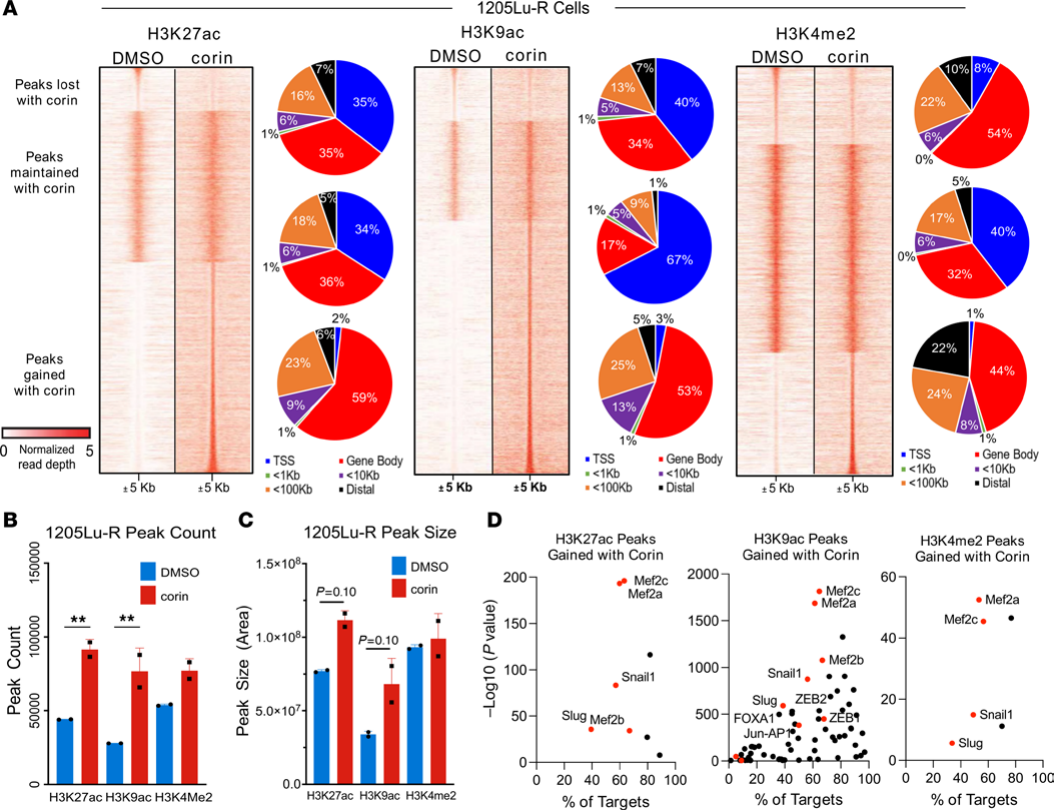
Global alterations in H3K27ac-,H3K9ac-, and H3K4me2-modified chromatin are enriched in phenotype switch–associated motifs in BRAFi-R cells treated with corin. (**A**) Tornado plots of H3K27ac, H3K9ac, and H3K4me2 ChIP-Seq peaks lost, maintained, or gained in 1205Lu-R cells treated with 2.5 μM corin for 24 hours, with the respective genomic location of peaks for each category shown to the right of each tornado plot. Window is ± 5 kb from the peak center and input signal subtracted (log_10_ likelihood ratio [logLR], 0:5). Data are representative of 2 independent experiments. (**B** and **C**) Total peak count (**B**) and peak size (**C**) for H3K27ac, H3K9ac, and H3K4me2 ChIP-Seq peaks in 1205Lu-R cells treated with 2.5 μM corin for 24 hours versus treatment with DMSO control (*n* = 2). **P < 0.01, by 2-tailed, unpaired *t* tests compared with DMSO control. (**D**) HOMER Motif analysis of H3K27ac, H3K9ac, and H3K4me2 ChIP-Seq peaks gained in 1205Lu-R cells treated with 2.5 μM corin for 24 hours compared with DMSO control. Data are representative of 2 independent experiments.

**Figure 8:**
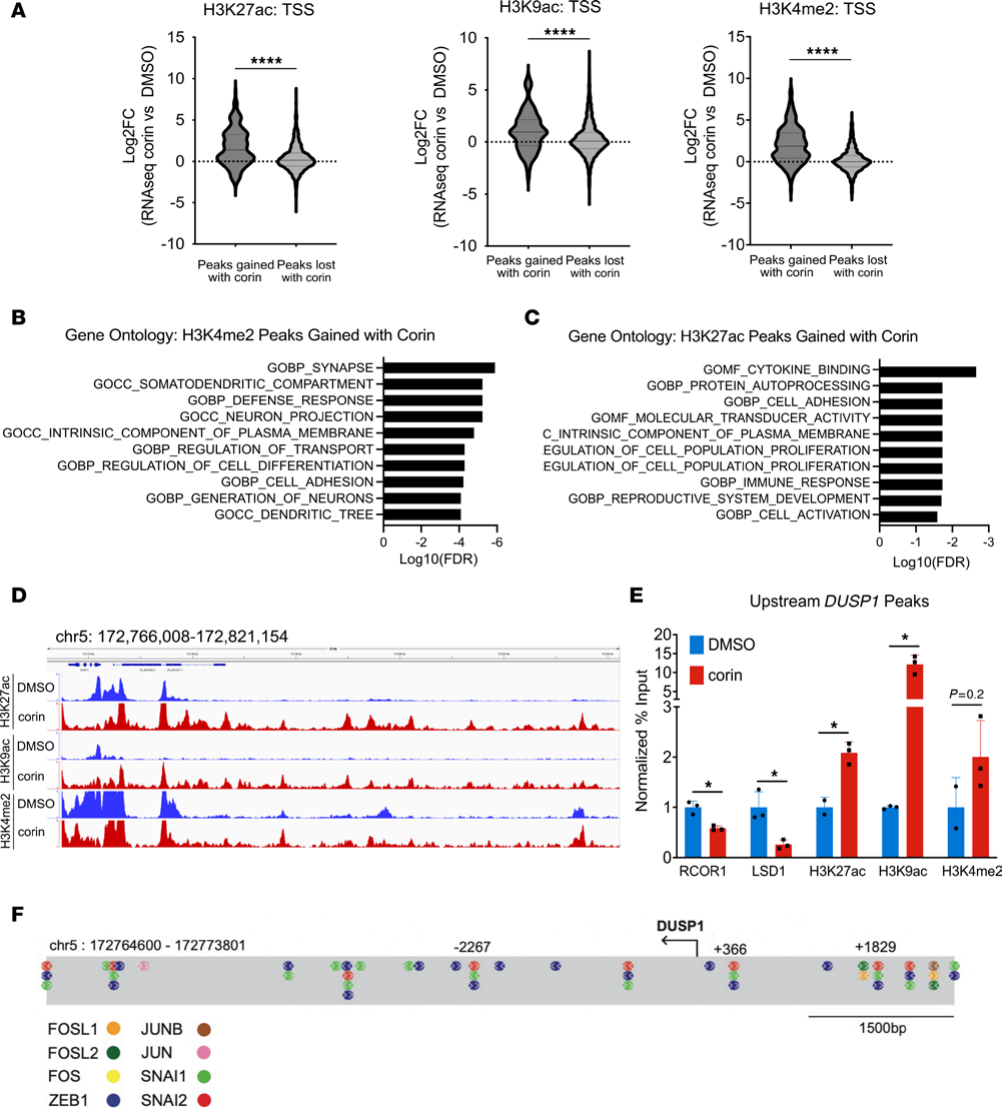
DUSP1 is a direct target of the CoREST complex. (**A**) Comparison of average RNA-Seq log_2_ FC for genes that gained or lost H3K27ac, H3K9ac, or H3K4me2 peaks at the TSS with corin treatment in 1205Lu-R cells (2.5 μM, 24 hours). (**B** and **C**) GO plots of genes with H3K27ac (**B**) or H3K4me2 (**C**) ChIP-Seq peaks gained at their TSS in 1205Lu-R cells treated with 2.5 μM corin for 24 hours compared with DMSO control. (**D**) Integrative Genome Viewer track of input-subtracted H3K27ac, H3K9ac, and H3K4me2 ChIP signals in the genomic region upstream of the DUSP1 TSS for 1205Lu-R cells treated with 2.5 μM corin for 24 hours compared with DMSO control. (**E**) ChIP-qPCR validation of RCOR1, LSD1, H3K27ac, H3K9ac, and H3K4me2 peaks gained upstream of the DUSP1 TSS in 1205Lu-R cells treated with 2.5 μM corin for 24 hours compared with DMSO control. Data from 1 representative biological replicate are shown. *P < 0.05 and ****P < 0.0001, by 2-tailed, unpaired *t* test. (**F**) EMT and AP-1 family member transcription factors enriched in the HOMER motif analysis mapped to the DUSP1 promoter sequence. Coordinates relative to the TSS were obtained from the UCSC Genome Browser (GRCh38).

To identify the genomic regions where corin-induced histone posttranslational modification (PTM) changes take place in BRAFi-R melanoma cells following corin treatment, we performed ChIP-Seq for H3K27ac, H3K9ac, and H3K4me2 on chromatin extracts from BRAFi-R cells treated with DMSO or corin for 24 hours (Figure 7A). Analysis of histone PTMs demonstrated that 47,746 H3K27ac, 54,223 H3K9ac, and 27,456 H3K4me2 peaks were gained by corin treatment, whereas 8,142 H3K27ac, 8,221 H3K9ac, and 11,457 H3K4me2 peaks were lost by corin treatment. We next analyzed the distribution of the H3K27ac/H3K9ac/H3K4me2-enriched corin-activated and corin-silenced regions relative to various genomic elements (29). Regions that gained H3K27ac, H3K9ac, and H3K4me2 due to corin treatment (“activated” chromatin) were primarily localized to gene bodies, with only 2% of increased marks being found at transcription start sites (TSSs) (Figure 7A). Histone PTM peak counts and peak sizes were also assessed following corin treatment and found to be increased most significantly for H3K27ac and H3K9ac (Figure 7, B and C). A search for enriched transcription factor–binding sites in chromatin regions where corin induced a more activated chromatin state using HOMER Motif analysis of H3K27ac, H3K9ac, or H3K4me2 ChIP-Seq peaks identified activated chromatin peaks at sites enriched for binding of the known CoREST-associated SNAG domain EMT transcription factors Slug and Snail1 (13), as well as the EMT-associated transcription factors ZEB1, ZEB2, Mef2a/2b/2c, Jun/AP1 (30–34), and FOXA1 (Figure 7D), similar to changes noted in the RNA-Seq analysis following corin treatment (Figure 6, E). Comparison of the average RNA-Seq expression changes for genes that gain or lose H3K27ac, H3K9ac, or H3K4me2 peaks at the TSS with corin treatment in 1205Lu-R cells showed significantly more peaks gained at TSSs in the setting of corin treatment for all histone marks evaluated (Figure 8A). In addition, Gene Ontology (GO) analyses of H3K27ac (Figure 8B) and H3K4me2 (Figure 8C) ChIP-Seq peaks gained at their TSS in 1205Lu-R cells treated with corin showed significant (FDR < 0.05) changes in cell adhesion, cell growth, neuronal development, and cellular differentiation, similar to changes seen in the RNA-Seq analyses (Figure 5). Overall, these data suggest that LSD1 and HDACs interacted with EMT-associated transcription factors to create a repressive chromatin environment in untreated cells and that corin blocked the removal of activating chromatin marks by LSD1 and HDACs at these regions. In addition, input-subtracted ChIP-Seq signals showed a broad increase in histone marks for H3K27ac, H3K9ac, and H3K4me2 in the genomic region upstream of the *DUSP1* gene (Figure 8D). In particular, RCOR1 and LSD1 directly interacted with DNA regions upstream of the DUSP1 promoter (Supplemental Figure 7, B and C); RCOR1 and LSD1 occupancy upstream of DUSP1 was reduced with corin treatment, while H3K27ac, H3K9ac, and H3K4me2 active histone marks were found to be increased upstream of the DUSP1 promoter following corin treatment, which was validated by ChIP-qPCR (Figure 8E). In addition, the *DUSP1* promoter was found to possess a significant number of AP-1– and EMT-associated transcription factor binding sites (Figure 8F) that were notably associated with corin effects on BRAFi-R melanoma cells (Figure 6E and Figure 7D). This suggests that DUSP1 is a direct target of the CoREST complex and is consistent with increased *DUSP1* mRNA expression in response to corin. We observed similar findings in 451Lu-R cells following corin treatment (Supplemental Tables 9 and 10).

### DUSP1 is a direct target of the CoREST complex and promotes a response to BRAFi therapy in BRAFi-R melanoma.

**Figure 9:**
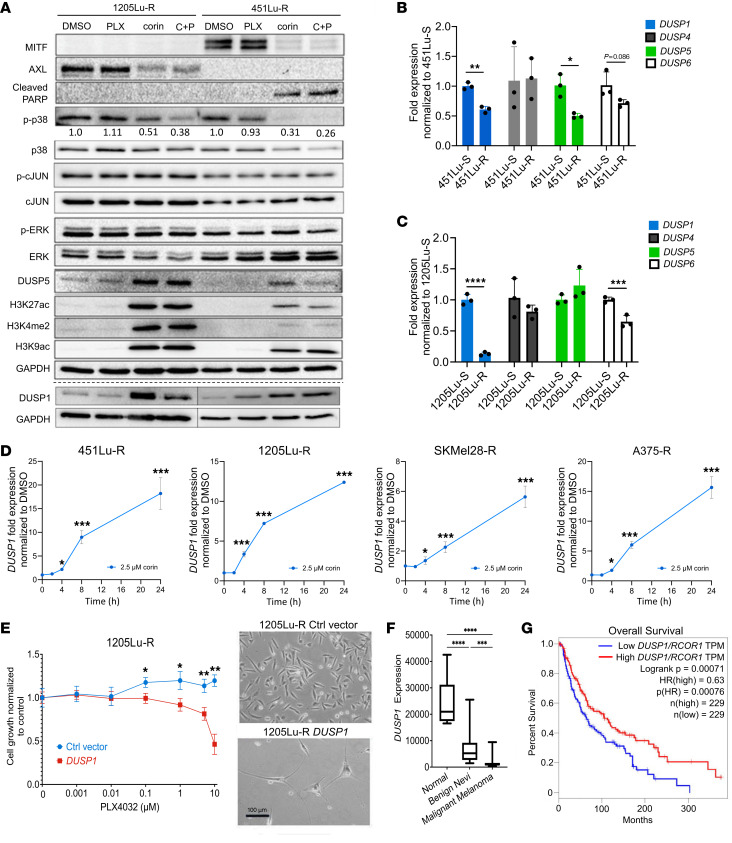
CoREST complex inhibition promotes a response to BRAFi therapy in BRAFi-R melanoma through DUSP1 upregulation. (**A**) Western blot analysis of 1205Lu-R and 451Lu-R melanoma cells treated with DMSO, 5 μM PLX4032 alone, 2.5 μM corin alone, or 2.5 μM corin plus 5 μM PLX4032 for 24 hours. Western blots were run contemporaneously with the exception of DUSP1. DUSP1 expression in 1205Lu-R and 451Lu-R cells was evaluated on separate Western blots, as denoted by the separating dotted line. Quantification of the relative expression of p-p38 (active) versus p38 (total) protein expression relative to the DMSO control is shown below each p-p38 band. (**B** and **C**) Quantification of *DUSP1*, *-4*, *-5*, and *-6* mRNA expression levels (RT-qPCR) in BRAFi-S (S) versus BRAFi-R (R) 451Lu (**B**) and 1205Lu (**C**) melanoma cells (*n* = 3). *P < 0.05, **P < 0.01, ***P < 0.001 and ****P < 0.0001, by 2-tailed, unpaired *t* test. (**D**) *DUSP1* expression levels (RT-qPCR) in 451Lu-R, 1205Lu-R, SkMel28-R, and A375-R melanoma cells treated with DMSO or 2.5 μM corin for 2, 4, 8, or 24 hours. *P < 0.05 and ***P < 0.001, by 1-way ANOVA with Tukey’s test. (**E**) Proliferation of 1205Lu-R melanoma cells overexpressing *DUSP1* versus control (Ctrl) vector, treated with increasing doses of PLX4032 for 72 hours. *P < 0.05 and **P < 0.01, by 2-way ANOVA with Tukey’s test. The morphology of 1205Lu-R melanoma cells following vector control (top) or DUSP1 overexpression (bottom) is depicted on the right. Representative images shown. Scale bar: 100 μm. (**F**) Quantification of *DUSP1* expression in normal skin (*n* = 7), benign nevi (*n* = 18), and malignant melanoma (*n* = 44) tissues from patients. Data were generated using microarray data from Talantov et al. (61). ***P < 0.001 and ****P < 0.0001, by 1-way ANOVA with Tukey’s test. (**G**) Kaplan-Meier curves illustrating the correlation of DUSP1/RCOR1 expression in patients’ tumor specimens (split at the median) and overall survival using data obtained from TCGA melanoma database (https://portal.gdc.cancer.gov).

As *DUSP* gene expression was among the most highly upregulated following corin/corin plus PLX4032 treatment of melanoma cells, and DUSP proteins are known inhibitors of the MAPK pathway, we sought to further explore the relationship between the CoREST complex and *DUSP* gene expression. Western blotting confirmed corin-associated increased expression of the dual-specificity phosphatases DUSP1 and DUSP5 in BRAFi-R cell lines (Figure 9A), which notably demonstrated significantly increased expression of DUSP1 and DUSP5 proteins following both corin and corin plus PLX4032 treatment of 1205Lu-R and 451Lu-R melanoma cells, and induction of DUSP transcripts by corin/corin plus PLX4032 was confirmed by RT-qPCR (Supplemental Figure 8A). As DUSPs are phosphatases that negatively regulate MAPK activity, we sought to further explore the functional significance of DUSP1 and DUSP5 as downstream effectors of corin with regard to the MAPK family members ERK, JNK, and p38 MAPK, which have all been implicated in melanoma resistance to targeted therapies (32, 35–37). Further evaluation of the ERK/MAPK signaling cascade revealed no significant effects of corin on ERK activation (Figure 9A), suggesting that ERK-specific DUSP5 was not a critical mediator of corin-induced BRAFi resensitization in BRAFi-R cells; however, examination of corin effects on p38 MAPK and JNK/c-JUN demonstrated significantly decreased phosphorylated p38 (p-p38) levels in BRAFi-R cells following corin treatment with or without PLX4032, whereas JNK/c-Jun activity was not significantly affected (Figure 9A and Supplemental Figure 8B), indicating selective DUSP1-mediated inhibition of p38 MAPK by corin.

As increased DUSP1 and DUSP5 expression occurred with corin treatment of BRAFi-R melanoma cells, we sought to establish baseline levels of DUSPs in BRAF-sensitive and BRAF-resistant melanoma cell lines. Remarkably, we found significantly reduced expression of several DUSPs, including *DUSP1*, in 451Lu-R and 1205Lu-R melanoma cell lines compared with their BRAFi-S counterparts (Figure 9, B and C), suggesting a potential role for DUSP proteins in regulating BRAFi resistance. Treatment of BRAFi-R melanoma cells with corin alone or combination treatment with corin plus PLX4032 resulted in up to 75-fold increases in *DUSP1* expression compared with treatment with vehicle (Figure 9D and Supplemental Figure 8A). In addition, we found that induction of *DUSP1* expression by corin was an early effect of corin, with significantly increased induction of *DUSP1* transcripts noted within 4 hours of corin treatment (Figure 9D).

To further explore the functional significance of *DUSP1* expression in BRAFi-R melanoma, we induced constitutive expression of *DUSP1* in BRAFi-R melanoma cells. 1205Lu-R cells overexpressing *DUSP1* showed significant growth inhibition following PLX4032 treatment compared with vector control, suggesting that *DUSP1* expression could resensitize BRAFi-R melanoma cells to PLX4032 (Figure 9E). In addition, *DUSP1* overexpression in 1205Lu-R cells resulted in distinct morphological changes resembling differentiated human melanocytes, supporting a potential role in the observed molecular phenotype switch (Figure 9E). In contrast, *DUSP1* knockdown in human melanoma cells led to loss of PLX4032 sensitivity in BRAFi-S melanoma cells (Supplemental Figure 9, A–C), with associated activation of p38 MAPK in 1205Lu cells (Supplemental Figure 9D), further supporting a critical role for DUSP1 in mediating melanoma resistance to BRAFi treatment.

In order to explore the relationship between *DUSP1* expression and melanomagenesis in patients, we mined publicly available gene expression data sets for normal skin, benign nevi, and patients’ primary melanoma tissues from The Cancer Genome Atlas (TCGA) repository. Notably, *DUSP1* expression was significantly decreased in malignant melanoma compared with both benign nevi and normal skin (Figure 9F), and decreased DUSP1 protein expression was noted in malignant melanoma tissues versus benign nevi by immunohistochemistry (Supplemental Figure 9E). TCGA data were further analyzed to explore the relationship between *DUSP1* and *RCOR1* expression in 412 melanoma specimens from patients. Kaplan-Meier plots of overall survival revealed that patients whose tumors showed higher ratios of *DUSP1/RCOR1* expression had significantly increased overall survival compared with patients with lower *DUSP1/RCOR1* ratios (Figure 9G), suggesting that high *DUSP1* expression in the setting of low *RCOR1* expression may be predictive of improved survival in patients. Of note, increased *DUSP1* expression alone was associated with improved overall survival in patients with melanoma, as previously reported (38); however, those data did not achieve statistical significance in the cohort we evaluated (Supplemental Figure 9F).

### Inhibition of p38 MAPK resensitizes BRAFi-R melanoma cells to BRAFi therapy.

**Figure 10:**
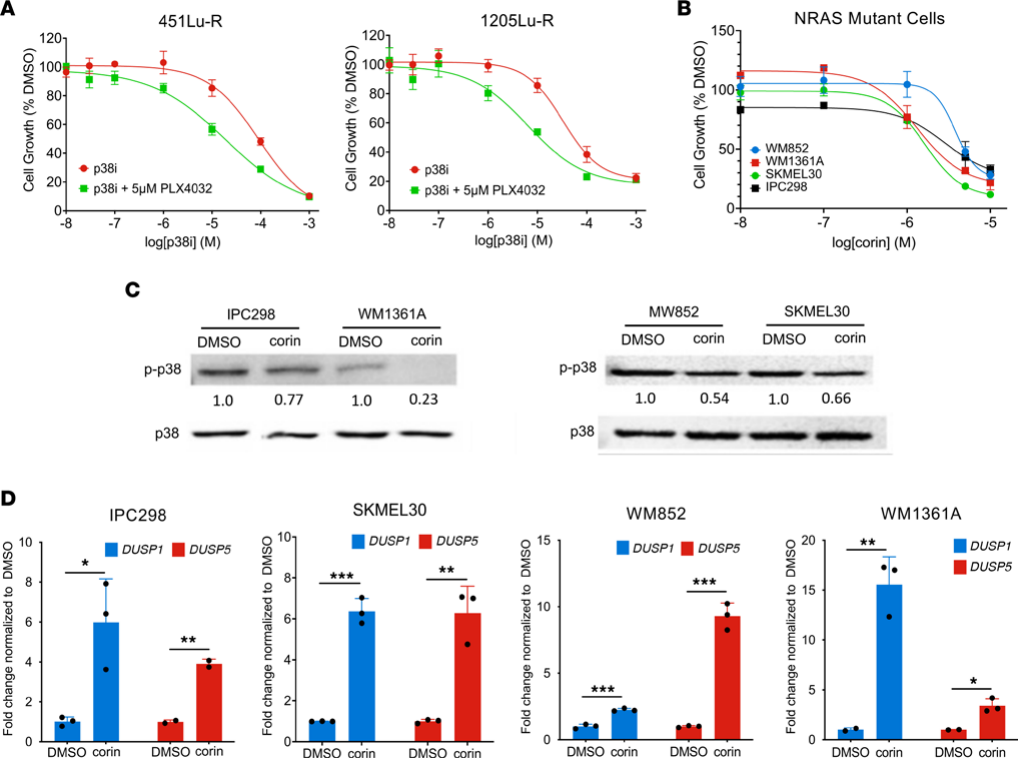
Inhibition of p38 MAPK resensitizes BRAFi-R melanoma cells to BRAFi therapy. (**A**) Proliferation assays of 451Lu-R and 1205Lu-R melanoma cells treated with increasing doses of p38i (BIRB-796) with or without 5 μM PLX4032 for 72 hours (*n* = 3). (**B**) Proliferation assays of WM852, WM1361A, Sk-Mel-30, and IPC298 NRAS-mutant melanoma cell lines treated with increasing doses of corin for 72 hours (*n* = 3). (**C**) Western blot data and quantification of the relative expression of p-p38 (active) versus p38 (total) protein in IPC298, WM1361A, MW852, and Sk-Mel-30 NRAS-mutant melanoma cell lines treated with DMSO or 2.5 μM corin for 24 hours. (**D**) DUSP1 and DUSP5 expression levels (RT-qPCR) in IPC298, Sk-Mel-30, WM852, and WM1361A NRAS-mutant melanoma cells treated with DMSO or 2.5 μM corin for 24 hours. *P < 0.05, **P < 0.01, and ***P < 0.001, by 2-tailed, unpaired *t* test.

As DUSP1 effects on p38 MAPK appeared to be a critical mediator of corin effects on melanoma resistance to BRAFi therapy, we sought to determine whether inhibition of the p38 MAPK target of DUSP1 would phenocopy the effects of corin on BRAFi-R melanoma cells. 451Lu-R (MITF^hi^/AXL^lo^) and 1205Lu-R (MITF^lo^/AXL^hi^) melanoma cells were treated with the p38 MAPK inhibitor BIRB 796 with or without 5 μM PLX4032. In both BRAFi-R melanoma cell lines, inhibition of p38 MAPK resensitized tumor cells to the BRAFi PLX4032 (Figure 10A). CI assessments identified synergies between the BRAFi PLX4032 and the p38 inhibitor BIRB 796 in both cell lines within the same range of p38i dosing (Supplemental Figure 9, G and H and Supplemental Tables 11 and 12). Recent studies in a zebrafish melanoma model (39) suggest that p38 MAPK functions as a tumor suppressor in NRAS-mutant melanomas. We therefore explored the role of DUSP1/p38 MAPK in NRAS-mutant melanomas and evaluated the effect of corin in dose-response assays. All NRAS-mutant melanomas demonstrated IC_50_ values in the micromolar range, with a 3-fold average lower sensitivity to corin versus BRAF-mutant melanomas (Figure 10B and Table 3). In addition, p38 MAPK activity was shown to be inhibited in all NRAS-mutant melanoma cell lines following corin treatment (Figure 10C), with associated increases in *DUSP1* and *DUSP5* expression (Figure 10D).

### Corin treatment of BRAFi-R melanoma inhibits tumors growth and promotes sensitization to PLX4032 in vivo.

**Figure 11:**
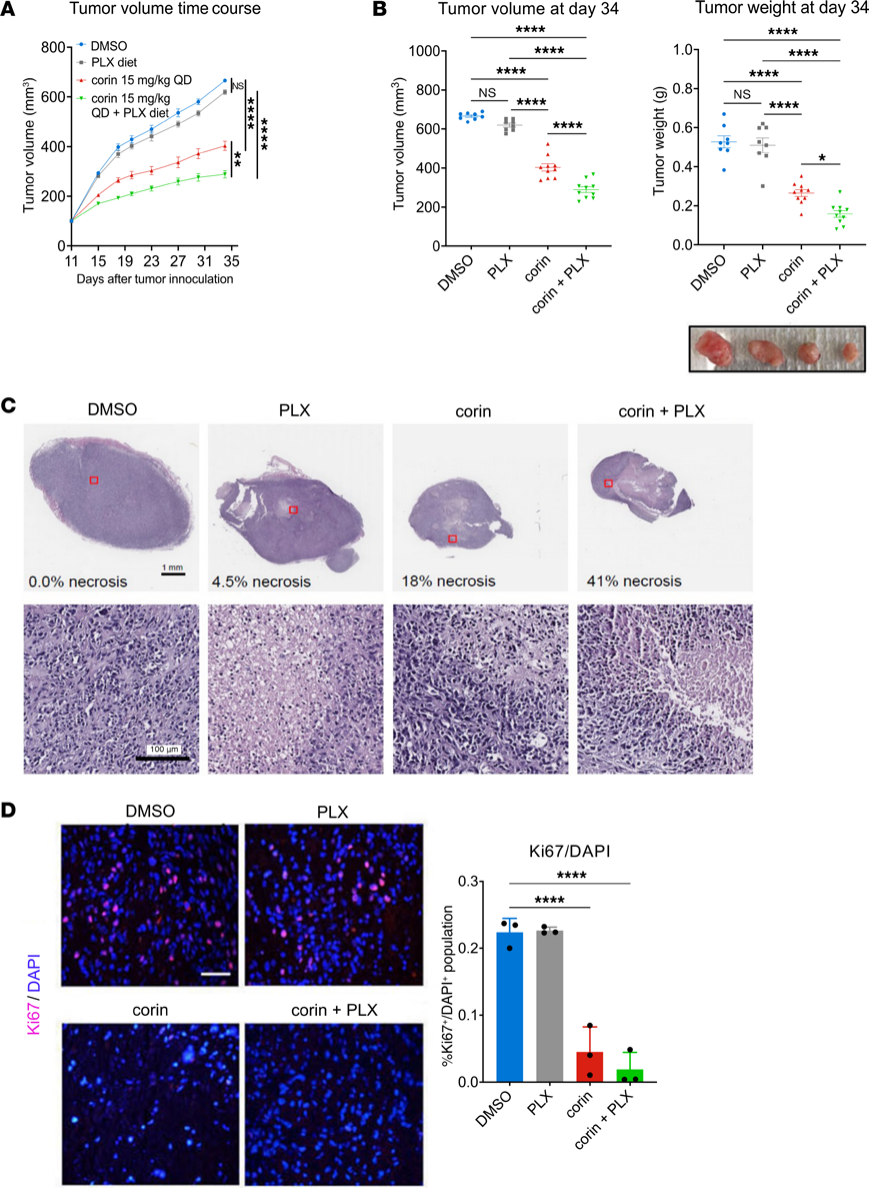
Corin treatment of BRAFi-R melanoma inhibits tumor growth and resensitizes tumors to PLX4032 in a mouse xenograft model. (**A**) 1205Lu-R melanoma tumor growth in a mouse xenograft model following administration of corin (15 mg/kg) with or without PLX4032 (*n* = 8–10). (**B**) Tumor volume and tumor weight following treatment of 1205Lu-R melanoma xenografts with corin (15 mg/kg), with or without PLX4032 (*n* = 8–10). A photograph of representative tumors is shown below. (**C**) H&E staining of tumor xenografts showing areas of significant necrosis (light purple stain) in corin and corin plus PLX4032 treatment groups. The percentage of area with necrosis (necrotic area/total tumor area) is noted in the upper panels (*n* = 2–3). Representative images are shown. Scale bars: 1 mm and 100 μm. (**D**) Cell proliferation in melanoma xenografts treated with corin, with or without PLX4032, as depicted by Ki67 staining and quantification (*n* = 3). Representative images are shown. Scale bar: 100 μm. *P < 0.05, **P < 0.01, and ****P < 0.0001, by 2-way ANOVA with Tukey’s test (**A**) or 1-way ANOVA with Tukey’s (**B**) or Dunnett’s (**D**) test.

**Figure 12:**
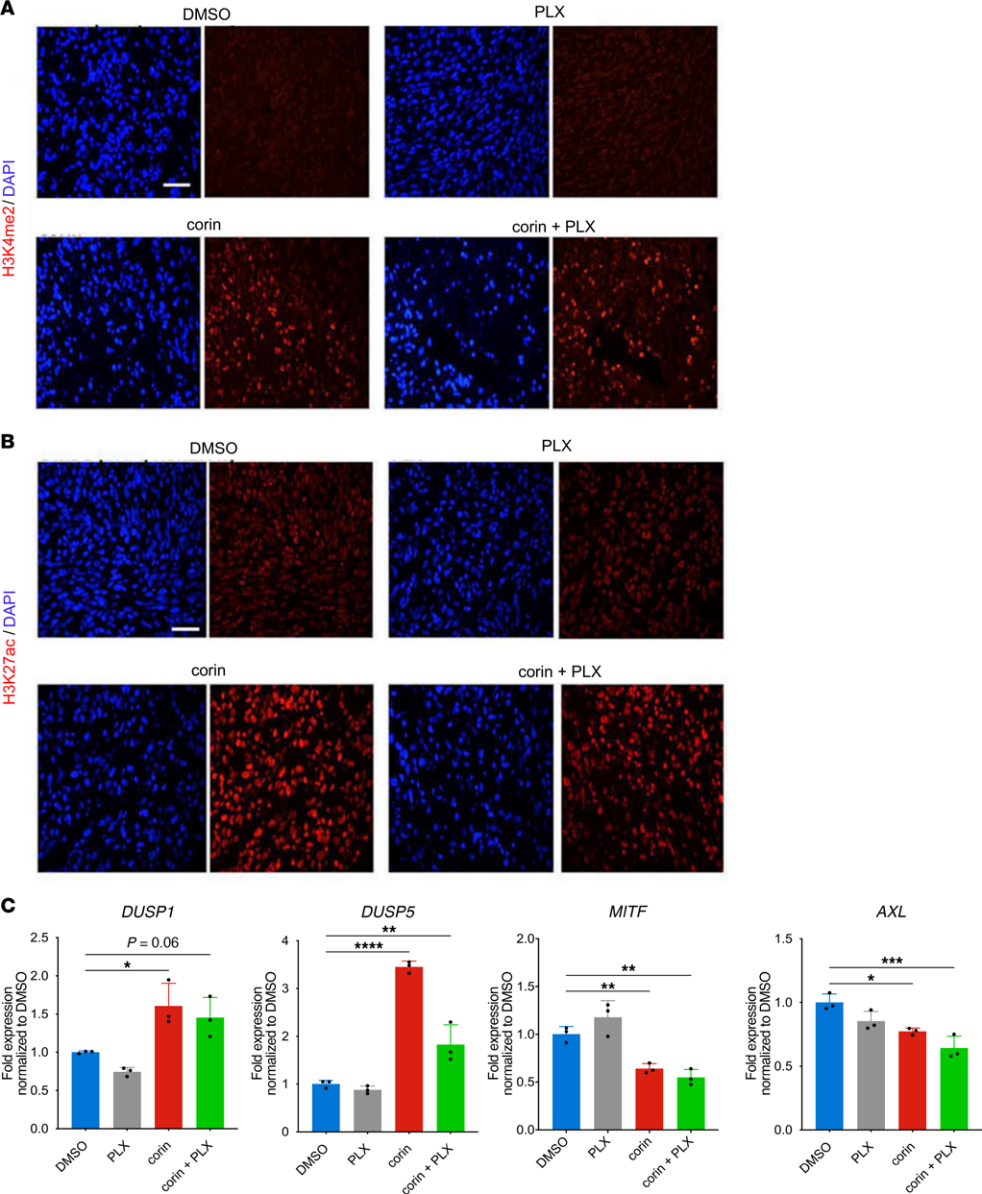
In vivo corin treatment increases DUSP1 expression in BRAFi-R melanoma tumors with associated activating histone marks and phenotype switch changes. (**A** and **B**) Immunofluorescence staining for H3K4me2 (**A**) and H3K27ac (**B**) expression in 1205Lu-R melanoma tumor xenografts following corin treatment with or without PLX4032. (**C**) Expression (RT-qPCR) of *DUSP1, DUSP5, MITF*, and *AXL* in 1205Lu-R melanoma xenografts treated with corin with or without PLX4032 (*n* = 3). *P < 0.05, **P < 0.01, ***P < 0.001, and ****P < 0.0001, by 1-way ANOVA with Dunnett’s test.

We next evaluated the effect of combining corin and PLX4032 in a BRAFi-R (1205Lu-R) melanoma mouse xenograft model. Mice were treated daily with 15 mg/kg corin i.p. and oral PLX4032. BRAFi-R tumors treated with PLX4032 alone showed progressive tumor growth that was similar to that seen in vehicle-treated animals (Figure 11A). In contrast, corin alone significantly inhibited tumor growth in the treated mice, whereas the combination treatment of corin plus PLX4032 showed significantly greater inhibition of tumor growth compared with corin treatment alone (Figure 11, A and B). Notable necrotic areas (Figure 11C) and decreased tumor cell proliferation (Figure 11D) were observed in tumor specimens following corin treatment alone or in combination with PLX4032. Additionally, corin treatment with or without PLX4032 led to increased expression of H3K4Me2, H3K27Ac, *DUSP1*, and *DUSP5* (Figure 12, A–C), as well as increased expression of markers of apoptosis and tumor hypoxia (Supplemental Figure 10) in BRAFi-R tumors, with associated decreases in the expression of both *MITF* and *AXL* (Figure 12C).

## Discussion

One of the major challenges in cancer research and clinical care is defining the molecular underpinnings of innate and acquired therapeutic resistance. In melanoma, the main targeted therapeutic strategy is directed against the MAPK pathway, with resistance to such therapies developing in the vast majority of patients. In this study, we demonstrate that the CoREST repressor complex plays a critical role in promoting cellular plasticity and phenotype switching in melanoma as well as in resistance to targeted BRAFi therapies through repression of the dual-specificity phosphatase DUSP1. Furthermore, targeting of the CoREST complex with the small-molecule inhibitor corin led to increased expression of DUSP1, inhibition of p38 MAPK activity, and resensitization of BRAF-resistant melanomas to BRAFi therapy. In addition, treatment of BRAFi-R melanomas with an inhibitor of p38 MAPK activity led to resensitization of these tumor cells to BRAFi therapy. Notably, Singh and colleagues (38) recently reported that melanoma cells are reliant on DUSP1 and DUSP8 expression for proliferation and that DUSP1 protein expression is reduced in MAPKi-sensitive and -resistant melanoma cells treated with BRAFi, consistent with the data reported here.

Recent studies have defined the cellular plasticity and dynamic phenotypes associated with acquired MAPKi drug resistance in melanoma and the epigenetic reprogramming observed in conditions of drug-induced stress that are ultimately fixed upon prolonged drug exposure, and suggest that intermediate cellular phenotypes are notably drug sensitive (1, 40). Here, we determined that the CoREST repressor complex modulated the predominant MAPKi-resistant melanoma phenotypes including the differentiated/proliferative (MITF^hi^/AXL^lo^) and neural crest stem cell–like/invasive (MITF^lo^/AXL^hi^) states and that corin treatment of BRAFi-R melanomas promoted the emergence of intermediate, BRAFi-S phenotypes that are concordant with recently profiled intermediate melanoma phenotypes (26). Such phenotype plasticity and associated drug resistance has been widely studied during the process of EMT in epithelial cancers, a process that is largely dictated by the expression of embryonic transcriptional programming (7); however, melanomas have a neural crest origin, suggesting that phenotype switch control mechanisms probably differ in this cell lineage (41). As the REST-CoREST complex specifically regulates repression of differentiation-associated genes during neural development (12), it is not wholly surprising that this complex would be reactivated to dictate the differentiation/proliferation balance in human melanoma cells. Indeed, expression of REST, the binding partner for CoREST, has been shown to be critical for early neural crest specification of developing melanoblasts (42), a phenotype that resembles the undifferentiated/invasive/MITF^lo^/AXL^hi^ neural crest stem cell phenotype (40). It is notable that our RNA-Seq data from 2 phenotypically distinct melanoma cell lines treated with the CoREST inhibitor corin showed lineage-specific effects on expression of the EMT transcription factors *ZEB1* and *ZEB2*, with downregulation of *ZEB2* in the MITF^hi^/AXL^lo^/differentiated melanoma cells (451Lu-R) and downregulation of *ZEB1* in the MITF^lo^/AXL^hi^/invasive/neural crest stem cells (1205Lu-R), consistent with corin suppression of the known distinct functions of these EMT-associated transcription factors in driving specific melanoma phenotypes (Figure 6A and Supplemental Figure 3, A–C) (33, 43, 44). Upregulation of *CDH1* in both melanoma phenotypes following corin treatment also suggests corin-associated inhibition of an EMT-like phenotype switch in these cells (Figure 6A). Indeed, corin-associated phenotypic changes in both MITF^hi^ and AXL^hi^ melanomas, including increased expression of E-cadherin and altered expression of MAPK, hedgehog signaling, focal adhesion, and axonal guidance–associated genes (Figure 5, C–E, Figure 6A, and Supplemental Figure 2, E and F), are reminiscent of those seen following inhibition of the neural crest differentiation–associated snail transcription factors during EMT in epithelial cancers (45). Not surprisingly, the HOMER Motif analysis of H3K27ac, H3K9ac, and H3K4me2 ChIP-Seq peaks gained in melanoma cells following corin treatment (Figure 7D) also demonstrated significantly (*P* < 0.0001) increased peaks for the EMT-associated transcription factors Snail1, Slug, ZEB1, andZEB2, consistent with a predominant effect on melanoma phenotypes. As the neural crest is an embryonic cell population of migratory and pluripotent cells that differentiate into diverse cell types including melanocytes, it is not surprising that melanoma cells hijack these developmental programs for tumor initiation and progression in a manner similar to EMT-associated changes in epithelial tumors.

Given the significance of snail-regulated transcriptional programs during development, it would be expected that a higher-order transcriptional network involving epigenetic reprogramming regulates the neural crest differentiation/migratory phenotype during development and the differentiation/proliferation phenotype in melanoma cells in a manner similar to the EMT seen in epithelial cancers. Notably, the SNAG domain of snail transcriptional repressor proteins has been shown to directly bind the CoREST repressor complex, acting as a histone H3 tail mimic in engaging the LSD1 active site, and this interaction has been shown to be responsible for regulating EMT in breast cancers and hematologic malignancies (13). Moreover, LSD1 inhibitors have been shown to disrupt LSD1/SNAG domain protein-protein interactions. Such LSD1/SNAG binding antagonism is very likely to occur with corin, which possesses an LSD1-targeting phenyl-cyclopropylamine warhead similar to that of canonical LSD1 inhibitors (46). Snail transcriptional repressors have also been shown to positively regulate the expression of ZEB transcription factors (47), which also associate with the CoREST complex during development (48). We therefore hypothesize that CoREST mediates transcriptional programs governing melanoma phenotypes and BRAFi resistance through interactions with EMT-associated transcription factors, including snail and ZEB repressor proteins, which are downstream of p38 MAPK (49). Furthermore, we anticipate that such epigenetic hierarchical regulation is an essential feature of critical EMT-like embryonic and wound-healing transcriptional programs as well as cellular responses to environmental stressors, as seen in phenotypic reprogramming of cancer cells exposed to pharmacologic therapies (50). Indeed, a survey of melanoma cell lines using the Broad Institute’s DepMap portal (https://depmap.org/portal/) found statistically significant inverse dependencies of expression of *MITF* with *SNAI1* and *AXL* with *SNAI2* as well as a significant inverse relationship between *SNAI1* and *SNAI2* as well as *ZEB1* and *ZEB2* (Supplemental Figure 11, A–F). The DepMap survey also found significant direct relationships between *SNAI1* and *ZEB1* as well as *SNAI2* and *ZEB2* in melanoma cell lines, further supporting a potential role for snail and ZEB transcription factors in the CoREST-mediated phenotype switch (Supplemental Figure 11, G and H). Interestingly, our previous data demonstrated strong growth inhibition by corin in all melanoma and leukemia cell lines tested (16); however, 50% of breast cancer cell lines and 70% of colon cancer cell lines evaluated also demonstrated strong growth inhibition by corin, suggesting that specific transcriptional programs, possibly relevant to a neural differentiation phenotype, may promote particular dependencies on CoREST-mediated cell growth. Recent studies of endocrine resistance and breast cancer plasticity have confirmed specific functions of the CoREST complex in mediating resistance to endocrine therapies in estrogen receptor–positive (ER^+^) breast cancers and associated tumor cell plasticity (17). This plasticity and therapy resistance are associated with a shift from CoREST bound to ERa and FOXA1 to CoREST recruitment to AP-1 sites with cJun binding and gene activation through recruitment of the SWI-SNF complex. In addition, CoREST inhibition blocks breast tumor cell growth and metastasis of both endocrine-sensitive and endocrine-resistant xenografts largely by inhibiting a gene signature associated with tumor invasiveness in patients (17).

As in development, the chromatin structure in tumor cells is critical to cellular phenotype and cell fate, with notable plasticity that lends itself to rapid reorganization under conditions of stress. Additionally, the rapid transitions afforded by epigenetic controls of gene expression serve as important mechanisms to evade therapeutic interventions (51, 52). Although global changes in the chromatin landscape have been noted during tumor progression in melanoma (5, 6), and histone H3 demethylases, including LSD1, have been shown to promote multidrug resistance (8, 36) and bypassing of oncogene-induced senescence (53), the specific targeting of these pathways has not led to meaningful clinical effects to date, while targeting of the HDACs in the setting of MAPKi resistance in melanoma has also failed to yield significant therapeutic benefits (54–56).

Our finding that inhibition of CoREST activity in human melanoma specifically inhibited both distinctive proliferative and invasive cellular phenotypes and that such inhibition promoted resensitization of treatment-resistant melanoma cells to targeted BRAF inhibition has, we believe, significant potential for clinical application, particularly since corin treatment of tumors in immunocompetent mice also promotes antitumor immunity and impaired Foxp3^+^ Treg function (15), suggesting that a therapeutic strategy targeting CoREST might enhance anticancer effects both through direct effects on tumor phenotype plasticity as well as by an enhanced immune response (Supplemental Figure 5). We therefore suggest that targeting of the CoREST complex in melanoma may provide a successful therapeutic approach to inhibit cellular plasticity and therapeutic resistance in human melanomas, while promoting antitumor immunity, which should be broadly applicable to other cancers that undergo similar epigenetic reprogramming.

## Methods

### Sex as a biological variable.

Our study exclusively examined female mice. It is unknown whether the findings would be similar for male mice, although we would not expect significant differences in the results.

Additional details can be found in the Supplemental Methods.

### Cell culturing.

The melanoma cell lines 451Lu, WM35, WM983B, Sbcl2, WM1552C, 1205Lu, and A375 were obtained from Meenard Herlyn (The Wistar Institute, Philadelphia, Pennsylvania, USA). SKMel28 cells were obtained from Levi A. Garraway (Dana-Farber Cancer Institute, Boston, Massachusetts, USA). SKMel28 BRAFi-R cells were obtained from Deborah Lang (Boston University, Boston, Massachusetts, USA). 451Lu BRAFi-R and A375 BRAFi-R cells were obtained from Jong-In Park (Medical College of Wisconsin, Milwaukee, Wisconsin, USA). 1205Lu BRAFi-R cells were generated as described below. The NRAS-mutant cells WM852, WM1361A, SK-Mel-30, and IPC298 were obtained from Anurag Singh (Boston University). All cell lines used were routinely checked for and found to be free of mycoplasma contamination. Melanoma cell lines were cultured in DMEM (Invitrogen, Thermo Fisher Scientific) supplemented with 10% FBS, l-glutamine (2 mM), and 1% penicillin/streptomycin. All BRAFi-R cell lines were generated by treating cells with increasing doses of PLX4032 (0.1–5 μM) over several months. BRAFi-R cell lines were cultured in 5 μM PLX to maintain BRAFi resistance. BRAFi resistance was routinely checked by treating BRAFi-S and BRAFi-R cell pairs with increasing doses of PLX4032 for 72 hours and assessing cell proliferation by PicoGreen. All cell lines were maintained in a 37°C incubator at 5% CO_2_.

### Compounds.

PLX4032 (vemurafenib, no. S1267) and MS275 (entinostat, no. 1053) were purchased from Selleck Chemicals. p38 inhibitor doramapimod (BIRB 796, no. HY-10320) was purchased from MedChemExpress. Corin, Cpd7, and A485 were provided by Philip Cole (Harvard Medical School, Boston, Massachusetts, USA). Compound stocks were prepared in DMSO with an equal amount of DMSO used as a vehicle control.

### Western blot analysis.

Whole-cell lysates were prepared in 3D-RIPA buffer. Proteins (20 μg) were separated by 10% or 12% SDS-PAGE and transferred onto a polyvinylidene difluoride membrane. Membranes were blocked using 5% nonfat dry milk in PBS containing 0.05% Tween 20 and then incubated with a primary antibody (Supplemental Table 13) overnight at 4°C. An HRP-conjugated secondary antibody was used and detected using the Pierce ECL Western Blot Substrate (Thermo Fisher Scientific). Blots shown are representative of at least 2 independent experiments.

### Western blot imaging and densitometric analysis.

Chemiluminescent blots were imaged with the ChemiDoc MP imager (Bio-Rad). The Band Analysis tools of ImageLab software, version 4.1 (Bio-Rad), were used to select and determine the background-subtracted density of the bands in all blots. The p-p38/p38 ratio was calculated using densitometric readings of individual bands of p-p38 and p38 for each treatment condition and normalized to the control.

### PicoGreen cell proliferation assay.

Cells were seeded in a 96-well plate and treated with an inhibitor at the indicated concentrations. Quanti-iT PicoGreen dsDNA assay was performed per the manufacturer’s protocol (Thermo Fisher Scientific), and fluorescence was measured at excitation and emission wavelengths of 480 nm and 520 nm, respectively, using a SpectraMax microplate reader.

### Cell morphology assays.

Cells were plated at a density of 100,000 cells per well, treated with either DMSO or 1 μM corin, and imaged every 12 hours for 72 hours. Twenty representative images from each treatment time point were evaluated for cell length using ImageJ software (NIH) (57).

### Senescence assays.

Senescent cells were detected by staining for lysosomal senescence–activated β-galactosidase activity with a commercial kit from Cell Signaling Technology (no. 9860). Treatment of 451Lu or 451Lu-R cells with 10 μM A485 for 4 days served as a positive control for senescence (58).

### LysoTracker staining.

451Lu and 1205Lu cells were seeded in 6-well plates at densities of 20,000 cells/well. Cells were treated for 24 hours with either 1:1,000 DMSO, 5 μM PLX4032, 2.5 μM corin, or 5 μM PLX4032 plus 2.5 μM corin. One well was left unstained as a negative control, and 1 well underwent a 6-hour HBSS starvation as a positive control. After treatment, cells were stained with 0.3 μM LysoTracker (L7528, Thermo Fisher Scientific) and visualized with a Nikon wide-field microscope. All images were analyzed with CellProfiler.

### Immunofluorescence.

Glass coverslips were placed into 6-well plates, and 50,000 cells were plated in each well. Cells were treated with DMSO control or 100 nM or 1 μM corin for 72 hours. Media were aspirated and cells were fixed to slides with 4% paraformaldehyde in PBS for 10 minutes. Slides were then washed twice with 0.1% Triton X-100 in PBS. Blocking solution (10% goat serum in PBS with 0.25% Triton X-100) was added for 30 minutes, followed by primary antibodies (Supplemental Table 13) diluted in blocking solution at 1:500, incubated at 4°C overnight. The next day, slides were washed twice with 0.1% Triton X-100 in PBS. Secondary antibodies were FITC-conjugated donkey anti–rabbit IgG, Alexa Fluor 594–conjugated donkey anti–rabbit IgG, and Alexa Fluor 488–conjugated goat anti–mouse IgG1 (Invitrogen, Thermo Fisher Scientific). Isotype-matched rabbit, mouse, or rat Ig was used in place of the primary antibody for the control. Sections were mounted with VECTASHIELD containing DAPI (H-1800, Vector Laboratories) and examined under a Nikon Eclipse E400 microscope equipped with FITC and TRITC filters (Nikon) and a Mercury 100W lamp (Chiu Technical Corp.).

### CoREST1 and DUSP1 knockdown.

shRNA clones targeting CoREST (TRCN0000128570 and TRCN0000129660) were obtained from the Hi Throughput Biology Center at Johns Hopkins University (Baltimore, Maryland, USA). DUSP1 Mission shRNA Bacterial Glycerol Stock (SHCLNG-NM_004417; a total of 5 constructs were tested: TRCN0000367616, TRCN0000367617, TRCN0000231489, TRCN0000002517, and TRCN0000002516) was purchased from MilliporeSigma.

Lentiviruses were produced in HEK293T cells using Lipofectamine 2000 (Invitrogen, Thermo Fisher Scientific) according to the manufacturer’s instructions and stored at –80°C after 0.22 μm filtration. For lentiviral infection, 451Lu BRAFi-R and 1205Lu BRAFi-R cells were incubated with CoREST shRNA. 451Lu BRAFi-S and 1205Lu BRAFi-S cells were incubated with DUSP1 shRNA or scrambled shRNA containing lentiviral particles overnight. Cells were selected with puromycin 48 hours after transduction to create stable cell lines. CoREST knockdown was determined by RT-qPCR and confirmed by Western blotting. The shRNA sequences are provided in Supplemental Table 14.

### DUSP1 overexpression.

The DUSP1 human overexpression plasmid (Origene, NM_004417, catalog RC205220) was expanded and transfected into 451Lu BRAFi-R and 1205Lu BRAFi-R cells using jetPRIME Transfection Reagent (Polyplus Transfection). DUSP1 transcript levels were determined by RT-qPCR to confirm overexpression efficiency.

### Colony formation assay.

451Lu BRAFi-R and 1205Lu BRAFi-R cells were seeded in 6-well tissue culture plates at 5,000 cells per well and treated with DMSO as a control, 5 μM PLX4032 alone, or 2.5 μM corin with or without 5 μM PLX4032 for 10 days. Following treatment, each well was fixed for 10 minutes with 4% paraformaldehyde (Electron Microscopy Services) diluted in PBS (Gibco, Thermo Fisher Scientific) and then stained for 20 minutes with Crystal Violet (Fisher Chemical). After washing and drying, each well was photographed and quantified using ImageJ software (57).

### Apoptosis detection.

451Lu BRAFi-R and 1205Lu BRAFi-R cells were treated with DMSO as a control, 5 μM PLX4032 alone, or 1 μM corin with or without 5 μM PLX4032 for 72 hours. Apoptosis was detected used the TUNEL-based TiterTACS Colorimetric Apoptosis Detection Kit (R&D Systems).

### Boyden chamber invasion assay.

Polycarbonate membrane (8 μm pore) Transwell inserts were placed in each well of a 24-well plate. Each insert was coated with 50 μg Matrigel diluted in 30 μL serum-free DMEM and allowed to polymerize at 37°C for 30 minutes (Corning). A total of 15,000 cells were pretreated with DMSO or 2.5 μM corin for 24 hours and seeded in 300 μL serum-free media in the top chamber; 600 μL of 20% FBS DMEM was added to the bottom chamber. After 24 hours of invasion, cells remaining in the top chamber were removed with a cotton swab, and cells that invaded to the bottom of the membrane were fixed in 70% ethanol, stained with 50 μg/mL propidium iodide, and washed in PBS. Cut-out membranes were mounted onto a slide with UltraCruz Mounting Medium (Santa Cruz Biotechnology). Four 10× and 20× images were acquired per membrane using the Nikon Eclipse E400 microscope and SPOT Advanced software. Fiji ImageJ was used to count invaded cells using the protocol previously described (57).

### Focal adhesion staining and quantification.

Glass coverslips were placed into 6-well plates, and 70, 000 cells were plated in each well. Cells were treated with DMSO control or 2.5 μM corin for 24 hours. Next, media were aspirated and cells were fixed to slides with 4% paraformaldehyde in PBS for 10 minutes. Slides were then washed twice with 0.1% Triton X-100 in PBS. Blocking solution (10% goat serum in PBS with 0.25% Triton X-100) was added for 30 minutes, followed by anti-vinculin antibody (MAB3574, MilliporeSigma) diluted in blocking solution at 1:300. Slides were left to incubate on a rocker for 2 hours at room temperature and then washed twice with 0.1% Triton X-100 in PBS. Texas Red-X Phalloidin, diluted 1:200 in PBS, was added to each slide and left to incubate for 20 minutes at room temperature. Slides were washed twice with 0.1% Triton X-100 in PBS and mounted with VECTASHIELD medium with DAPI (H-1800, Vector Laboratories). Quantification of focal adhesions was performed according to a previously described protocol (59).

### RNA-Seq.

451Lu BRAFi-R and 1205Lu BRAFi-R cells were treated for 24 hours with 5 μM PLX4032, 2.5 μM corin, 5 μM PLX4032 plus 2.5 μM corin, or DMSO as a control. RNA was isolated from cells using the RNeasy Plus Mini kit (QIAGEN) following the manufacturer’s instructions. A workflow diagram is depicted in Supplemental Figure 12.

### ChIP-Seq/qPCR.

451Lu-R or 1205Lu-R melanoma cells were treated with 2.5 μM corin or DMSO for 24 hours and grown to 80% confluence in 150 cm^2^ plates. ChIP was conducted as previously described (60). The following antibodies were used for IP: RCOR1 (10 μg/sample, 07-455, MilliporeSigma), LSD1 (10 μg/sample, ab17721), H3K27ac (4 μg/sample, ab4729), H3K9ac (4 μg/sample, ab32129), H3K4me2 (4 μg/sample, ab32356), and IgG, with input samples used as controls. Two biological replicates were performed for each cell line. For ChIP-Seq, library preparation and Illumina sequencing of ChIP DNA was performed by Azenta, with 40 million 2 × 150 bp paired-end reads for each sample (the workflow diagram is depicted in Supplemental Figure 12 for clarification). For ChIP-qPCR, DNA was purified with the PCR Purification Kit (28104, QIAGEN) following the manufacturer’s instructions, and RT-qPCR was performed. The relevant *DUSP1* primer is listed in Supplemental Table 14.

### RNA-Seq, ChIP-Seq, and open-source database analyses.

See Supplemental Methods for details.

### RT-qPCR.

RNA was isolated from melanoma cells following the manufacturer’s instructions and cleaned using the RNeasy Mini kit (QIAGEN). RNA (1 μg) was reverse transcribed using the SuperScript III First-Strand Synthesis System kit (Invitrogen, Thermo Fisher Scientific). RT-qPCR was performed for 40 cycles of 15 seconds each at 95°C and for 30 seconds at 60°C using the Step One Plus Real-time PCR System (Applied Biosystems). Data represent 2 independent experiments with 3 technical replicates per experiment. The data were quantified using the ΔCt method. Where *P* values are reported, an unpaired *t* test was used to determine significance. Transcripts were amplified using the primers listed in Supplemental Table 14.

### Mouse melanoma xenograft.

Mice were maintained under pathogen-free conditions in an American Association for Accreditation of Laboratory Animal Care–accredited (AAALAC-accredited) facility at the Boston University Medical Center, under the supervision of the Laboratory Animal Science Center (LASC) and its staff of veterinarians and support personnel. For the 1205Lu BRAFi-R xenograft studies, 6- to 8-week-old female BALB/C nude mice were purchased from Charles River Laboratories and allowed to acclimate for 1 week prior to beginning the experiment. For each animal, 1205Lu BRAFi-R (6 × 10^6^) cells in 100 μL growth media mixed with 50% Matrigel (BD) were injected bilaterally into the subcutaneous tissue of both flanks. On day 3 after tumor inoculation, animals were randomly assigned to 1 of 2 groups that were administered a vemurafenib diet or a control diet by the Research Randomizer (http://www.randomizer.org). The vemurafenib diet (5.67 g/kg body weight to achieve a 100 mg/kg body weight daily dose) and the control diet were prepared at Envigo. When the xenograft size reached an approximate volume of 100 mm^3^, the 20 mice were randomized to groups of 5 mice per group, with the average tumor volume distributed equally between the groups. Vehicle control (5% DMSO/H_2_O) and corin (15 mg/kg) were administered at 10 mL/kg i.p. once a day. The mice were maintained in a pathogen-free environment with free access to food and water. Body weight and tumor volume were measured twice weekly. Tumor size was measured with linear calipers and calculated using the following formula: length in mm × width in mm^2^/2. The mice were sacrificed after 23 days, and tumor weights were measured. Three animals from each treatment group were chosen at random for follow-up data analysis.

### Xenograft tumor sample processing and immunohistochemistry.

After the mice were sacrificed, tumors were removed and fixed in 3.7% formaldehyde. Serial sections of 5 μm thickness were cut from the formalin-fixed, paraffin-embedded tissue blocks, floated onto charged glass slides (Super-Frost Plus, Thermo Fisher Scientific), and dried overnight at 60°C.

Sections were deparaffinized and hydrated using graded concentrations of ethanol to deionized water prior to immunohistochemical analysis. Tumor sections were blocked in serum (5% serum in PBS-T containing 0.5% Triton X-100 in PBS) and then incubated overnight at 4°C with primary antibodies (Supplemental Table 13). Sections were then incubated with a fluorescence-conjugated (FITC) goat anti-rabbit secondary antibody for 1 hour at room temperature, washed with PBS, and mounted using VECTASHIELD mounting medium (Vector Laboratories).

### H&E staining.

Animal tissue H&E staining was performed at Applied Pathology (Worcester, Massachusetts, USA) using their standard protocol (https://files.sitebuilder.name.tools/enom52979/file/hestainingprotocol.pdf).

### Statistics.

Results were considered significant for an adjusted *P* value of less than 0.05. Data are presented as the mean ± SD and are representative of at least 2 independent experiments. Tests used to determine statistical significance are noted in the figure legends and include an unpaired, 2-tailed *t* test, a 1-way ANOVA with Tukey’s or Dunnett’s test, or a 2-way ANOVA with Tukey’s test.

### Study approval.

Animal studies were approved by the IACUC of Boston University (protocol 15503).

### Data availability.

Raw data, processed data, and metadata from the RNA-Seq and ChIP-Seq experiments have been deposited in the NCBI’s Gene Expression Omnibus (GEO) database (Series GSE254703). All data supporting the graphs and tables are provided in the Supplemental Supporting Data Values file. All codes used for analysis of sequencing were deposited in GitHub (https://github.com/robertfisher002/Wu_Corin_Manuscript; branch main; commit ID 92ac17b).

## Author contributions

MW, M Collard, PAC, and RMA conceived the study, interpreted data, and wrote the manuscript. MW, AH, FG, RF, SR, KP, AZ, KK, SN, M Cole, and AX, and M Collard carried out the cell-based work. MW and KK performed the mouse xenograft studies. JHK designed and synthesized the CoREST inhibitor corin. AL, FA, RF, M Collard, SB, and MW performed RNA-Seq/ChIP-Seq data analysis. All authors were involved in the experimental design, data interpretation, and editing of the manuscript.

## Supplementary Material

Supplemental data

Unedited blot and gel images

Supporting data values

## Figures and Tables

**Table 3:**
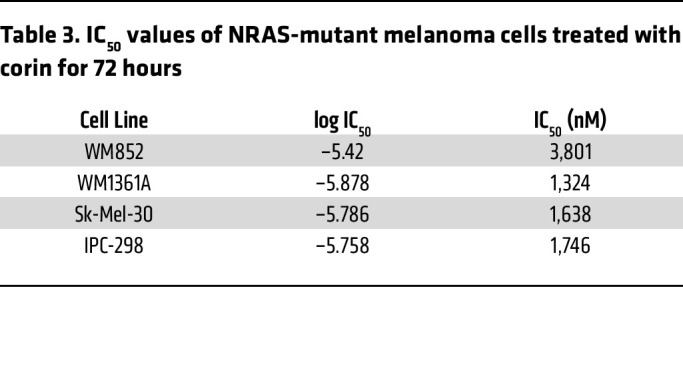
IC_50_ values of NRAS-mutant melanoma cells treated with corin for 72 hours

**Table 1:**
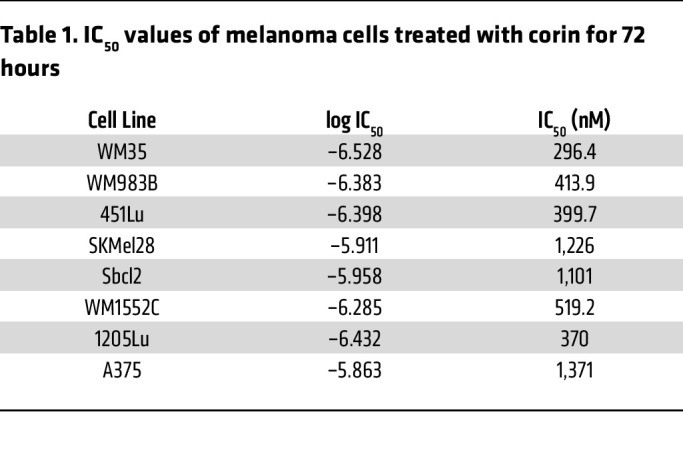
IC_50_ values of melanoma cells treated with corin for 72 hours

**Table 2:**
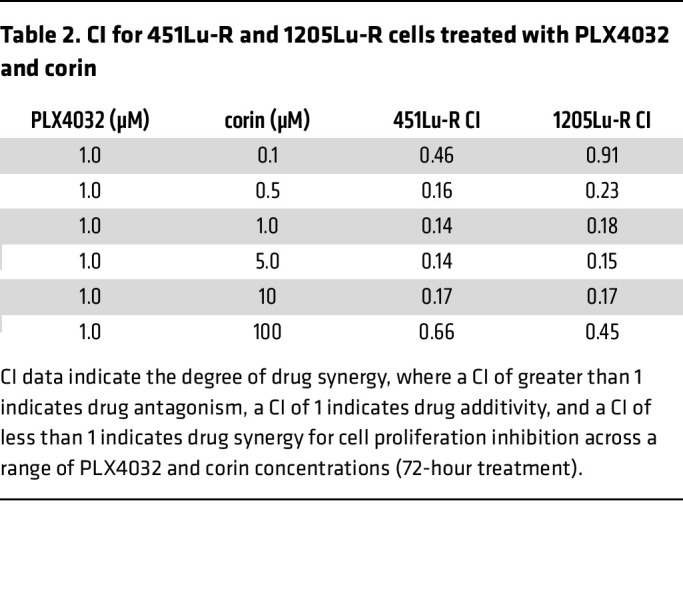
CI for 451Lu-R and 1205Lu-R cells treated with PLX4032 and corin

## Version 1 02/01/2024

In-Press Preview

## Version 2 03/15/2024

Electronic publication
