# Supplementary material for: The CoREST repressor complex mediates phenotype switching and therapy resistance in melanoma
Source: J Clin Invest. 2024 Feb 1;134(6):e171063. doi: 10.1172/JCI171063 (PMC10940100; doi:10.1172/JCI171063)
Supplement: Supplemental data [file jci-134-171063-s110.pdf]

The CoREST Repressor Complex Mediates Phenotype Switching and Therapy Resistance in Melanoma

Muzhou Wu[1], Ailish Hanly[1], Frederick Gibson[1], Robert Fisher[1], Samantha Rogers[1], Kihyun Park[1], Angelina Zuger[1], Kevin Kuang[1], Jay H. Kalin[2], Sarah Nocco[1], Matthew Cole[1], Amy Xiao[1], Filisia Agus[3], Adam Labadorf[3,4], Samuel Beck[1], Marianne Collard[1], Philip A. Cole[2*], Rhoda M. Alani[1]*

[1]Department of Dermatology, Boston University Chobanian & Avedisian School of Medicine, Boston, MA, 02118, USA.

[2]Division of Genetics, Departments of Medicine and Biological Chemistry and Molecular Pharmacology, Harvard Medical School and Brigham and Women's Hospital, Boston, MA, 02115, USA

[3]Bioinformatics Program, Boston University, Boston, 02118 USA

[4]Department of Neurology, Boston University Chobanian & Avedisian School of Medicine, Boston, 02118 USA

Supplemental Methods

RNA-Seq data analysis

Paired-end RNA-Seq reads were quality- and adapter-trimmed using trimmomatic (1) and gene expression was quantified using the salmon software package (2) against the GENCODE v32 transcriptome (3) summarized to the gene level. Differential expression analysis for pairwise cell type/treatment conditions was conducted using DESeq2 (4) modeling counts as a function of treatment vs the relevant control. Genes with a false discovery rate < 0.01 were considered significant. Gene set enrichment analysis was performed using the differential expression results ranked by decreasing effect size (i.e. log2 fold change) for each pairwise comparison using the fgsea R package (5) with the KEGG pathways (6) and REACTOME pathways (7) gene set databases. Gene sets with a false discovery rate of < 0.01 were considered significant. The analysis code is available at https://bitbucket.org/bucab/corin_melanoma. Box plots and Volcano plots were generated using Prism 6 software (GraphPad Software). A workflow diagram is included as Supplemental Figure 12 for clarification.

HOMER Motif Analysis

We analyzed corin-upregulated genes (Corin+PLX vs PLX; p<0.01, LF>2) for enrichment of transcription factor motifs using HOMER v4.10.1 *findMotifs* against random background sequences (len = 8-10bp, promoters approximated to -400 to +100 TSS). Both "de novo" and "known" motif analyses were generated.

DUSP1 Promoter Mapping

EMT and AP-1 family member transcription factors enriched in the HOMER motif analysis were mapped to the DUSP1 promoter (annotated in Ensembl GRCh38.p130) using TFmotifView (8). Coordinates relative to the TSS were obtained from the UCSC Genome Browser (GRCh38).

ChIP-seq Analysis

FASTQ files were aligned using Bowtie 2.5.0 (9) to the reference genomes (hg38, Genome Reference Consortium Human Build 38). Peak calling was performed using Model-based Analysis of ChIP-seq (MACS) 1.4.2 (10) with '--nomodel' and '--nolambda' options. For signal-based analyses, duplicate reads were filtered using Picard 3.0.0 [https://broadinstitute.github.io/picard/]. In order to address the potential effects of global histone modification alteration (Figure 1, A and B), a scale factor (SF) was determined for each experimental sample using ChIPseqSpikeInFree (11). Subsequently, the read density for each genomic region was normalized by scaling it with the factor of 1e7 / (mapped read counts * SF) using the 'genomecov' command available in the BEDTools suite (12). This normalization process helped to account for variations stemming from global histone modification changes, ensuring more accurate data analysis and interpretation. Unique and overlapping peaks were defined using 'intertect' command in BEDTools suite. Motif occurrence analysis was performed using 'findMotifs.pl' command in hypergeometric optimization of motif enrichment (HOMER) suite version 4.4 (13). For ChIP-seq result correlation with RNA-seq data, expression level of each transcript isoform was quantified using Salmon version 1.9.0 (2). Peaks were visualized in the Integrative Genomics Viewer (IGV) (14). Figures show data from one representative biological replicate.

Open Source Database Analyses

DUSP1 expression in normal skin, benign nevi, and malignant melanoma was obtained from publicly available microarray data (15). Kaplan-Meier curves were generated by GEPIA (http://gepia.cancer-pku.cn) using the TCGA database (http://cancergenome.nih.gov/). Comparison analysis was performed using publicly available whole-exome sequence (WES) datasets of serial tumor biopsies (baseline and acquired resistant tumors) from patients with advanced melanoma treated with MAPK inhibitor (MAPKi) regimens (16) by comparing the list of 'Loss of Function (LOF)' genes from the dataset with corin-regulated genes. RCOR1, HDAC1, HDAC2, and KDM1A expression in BRAFi-resistant tumors compared to baseline was analyzed with data from the validation microarray dataset (GSE50509 and GSE61992) used by Song et al (16).

Calculation of the combination index (CI)

The combined activity of corin and PLX4032 was determined by calculating the CI for both compounds in 451Lu-R, 1205Lu-R and A375-R cells using Compusyn software (Biosoft). The concentrations used for PLX4032 alone are: 0.01µM, 0.1µM, 1µM, 5µM, 10µM; concentrations used for corin alone are: 0.01µM, 0.1µM, 1µM, 5µM, 10µM. Concentrations used for combination treatment are: 1µM PLX4032 + corin (0.01µM, 0.1µM, 1µM, 5µM, 10µM). The results are interpreted as: $0 < CI < 1$ indicates synergism; $CI = 1$ indicates an addictive effect; and $CI > 1$ indicates antagonism.

1. Bolger AM, et al. Trimmomatic: a flexible trimmer for Illumina sequence data. *Bioinformatics.* 2014;30(15):2114-20.
2. Patro R, et al. Salmon provides fast and bias-aware quantification of transcript expression. *Nat Methods.* 2017;14(4):417-9.
3. Harrow J, et al. GENCODE: the reference human genome annotation for The ENCODE Project. *Genome Res.* 2012;22(9):1760-74.
4. Love MI, et al. Moderated estimation of fold change and dispersion for RNA-seq data with DESeq2. *Genome Biol.* 2014;15(12):550.
5. Gennady K, et al. Fast gene set enrichment analysis. *bioRxiv.* 2021:060012.
6. Kanehisa M, and Goto S. KEGG: kyoto encyclopedia of genes and genomes. *Nucleic Acids Res.* 2000;28(1):27-30.
7. Croft D, et al. The Reactome pathway knowledgebase. *Nucleic Acids Res.* 2014;42(Database issue):D472-7.
8. Leporcq C, et al. TFmotifView: a webserver for the visualization of transcription factor motifs in genomic regions. *Nucleic Acids Res.* 2020;48(W1):W208-W17.
9. Langmead B, and Salzberg SL. Fast gapped-read alignment with Bowtie 2. *Nat Methods.* 2012;9(4):357-9.
10. Zhang Y, et al. Model-based analysis of ChIP-Seq (MACS). *Genome Biol.* 2008;9(9):R137.
11. Jin H, et al. ChIPseqSpikeInFree: a ChIP-seq normalization approach to reveal global changes in histone modifications without spike-in. *Bioinformatics.* 2020;36(4):1270-2.
12. Quinlan AR, and Hall IM. BEDTools: a flexible suite of utilities for comparing genomic features. *Bioinformatics.* 2010;26(6):841-2.
13. Heinz S, et al. Simple combinations of lineage-determining transcription factors prime cis-regulatory elements required for macrophage and B cell identities. *Mol Cell.* 2010;38(4):576-89.
14. Robinson JT, et al. Integrative genomics viewer. *Nat Biotechnol.* 2011;29(1):24-6.
15. Talantov D, et al. Novel genes associated with malignant melanoma but not benign melanocytic lesions. *Clin Cancer Res.* 2005;11(20):7234-42.
16. Song C, et al. Recurrent Tumor Cell-Intrinsic and -Extrinsic Alterations during MAPKi-Induced Melanoma Regression and Early Adaptation. *Cancer Discov.* 2017;7(11):1248-65.
17. Wouters J, et al. Robust gene expression programs underlie recurrent cell states and phenotype switching in melanoma. *Nat Cell Biol.* 2020;22(8):986-98.

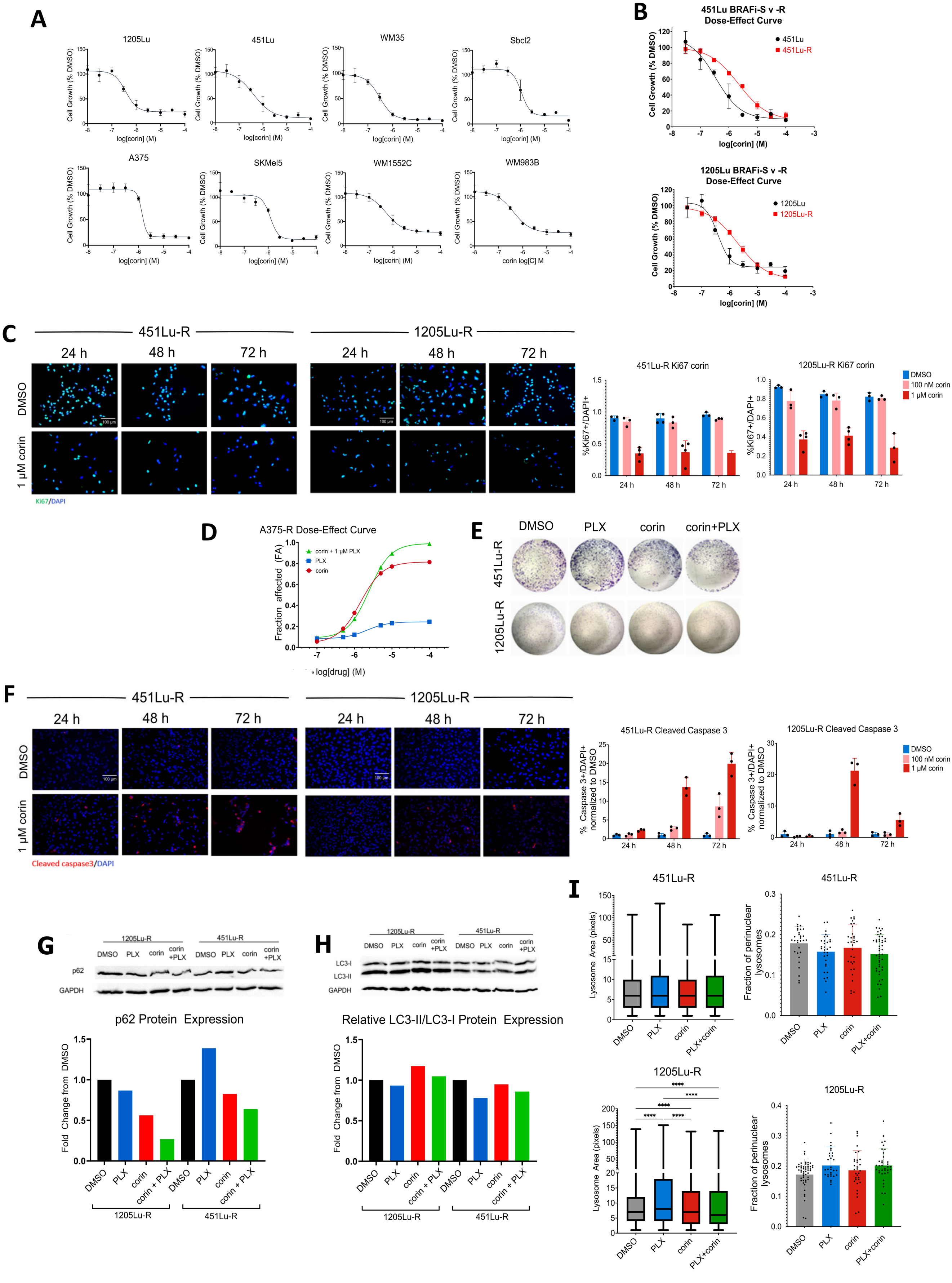
A
1205Lu
451Lu
WM35
Sbcl2
A375
SKMel5
WM1552C
WM983B
Cell Growth (% DMSO)
log[corin] (M)
corin log[C] M
B
451Lu BRAFi-S v -R
Dose-Effect Curve
451Lu
451Lu-R
1205Lu BRAFi-S v -R
Dose-Effect Curve
1205Lu
1205Lu-R
Cell Growth (% DMSO)
log[corin] (M)
C
451Lu-R
1205Lu-R
24 h
48 h
72 h
DMSO
1 μM corin
Ki67/DAPI
451Lu-R Ki67 corin
1205Lu-R Ki67 corin
%Ki67+/DAPI+
DMSO
100 nM corin
1 μM corin
D
A375-R Dose-Effect Curve
corin + 1 μM PLX
PLX
corin
Fraction affected (FA)
log[drug] (M)
E
DMSO
PLX
corin
corin+PLX
451Lu-R
1205Lu-R
F
451Lu-R
1205Lu-R
24 h
48 h
72 h
DMSO
1 μM corin
Cleaved caspase3/DAPI
451Lu-R Cleaved Caspase 3
1205Lu-R Cleaved Caspase 3
% Caspase 3+/DAPI+ normalized to DMSO
DMSO
100 nM corin
1 μM corin
G
1205Lu-R
451Lu-R
DMSO
PLX
corin
corin +PLX
p62
GAPDH
p62 Protein Expression
Fold Change from DMSO
DMSO
PLX
corin
corin + PLX
H
1205Lu-R
451Lu-R
LC3-I
LC3-II
GAPDH
Relative LC3-II/LC3-I Protein Expression
Fold Change from DMSO
I
451Lu-R
1205Lu-R
Lysosome Area (pixels)
Fraction of perinuclear lysosomes
DMSO
PLX
corin
PLX+corin
****

Supplemental Figure 1. CoREST inhibition promotes response to BRAFi-therapy in BRAFi-resistant melanoma cells. **(A)** Proliferation assays of BRAFi-sensitive cell lines (1205Lu, 451Lu, WM35, Sbcl2, A375, SKMel28, WM1552C and WM983B) treated with increasing doses of corin for 72 h. **(B)** Cell proliferation assay of 451Lu and 1205Lu BRAFi-sensitive (-S) and -resistant (-R) cell lines treated with increasing doses of corin for 72 h with the corin. **(C)** Immunofluorescent staining of Ki67 and quantification in 451Lu-R and 1205Lu-R BRAFi-resistant melanoma cell lines following 24 h, 48 h, 72 h treatment with DMSO, 100 nm corin, or 1 μM corin. Representative images shown for 1 μM corin, scale bar = 100 μm. **(D)** Drug synergy graph for corin and PLX4032 in A375-R BRAFi-R melanoma lines. **(E)** Colony formation assays for 451Lu-R and 1205Lu-R melanoma cells treated with DMSO, 5 μM PLX4032 alone, 2.5 μM corin alone, and 2.5 μM corin + 5 μM PLX4032 for 10 days. **(F)** Immunofluorescence staining of cleaved caspase-3 and quantification in 451Lu-R and 1205Lu-R BRAFi-resistant melanoma cell lines following 24 h, 48 h, 72 h treatment with DMSO, 100 nM corin, or 1 μM corin. Representative images shown for 1 μM corin, scale bar = 100 μm. **(G-I)** Autophagy analysis in 1205Lu-R and 451Lu-R melanoma cells following 24 h treatment with DMSO, 5μM PLX4032 alone, 2.5 μM corin alone, or 5μM PLX4032 + 2.5 μM corin using p62 Western blot protein expression and quantification **(G)** LC3-II/LC3-I Western blot protein expression and quantification (**H**), and **(I)**, Lysotracker staining of lysosomes in 451Lu-R (top) and 1205Lu-R (bottom). Representative images shown, scale bar = 100 μm. Significance asterisks indicate 1-way ANOVA with Turkey's test. All data represents mean + SE. ***$P < 0.001$, **$P < 0.0001$.

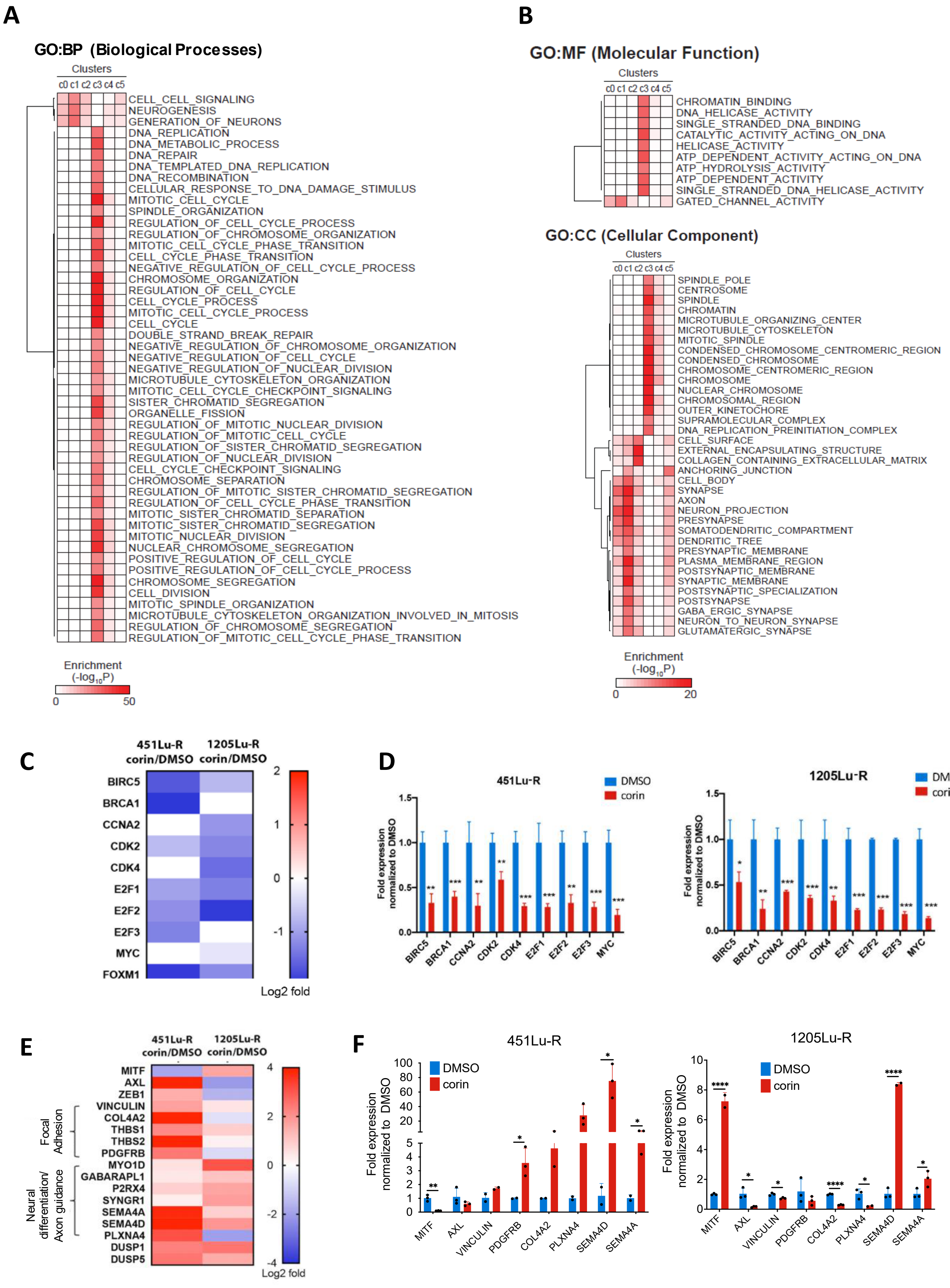
A
GO:BP (Biological Processes)
Clusters
c0 c1 c2 c3 c4 c5
CELL_CELL_SIGNALING
NEUROGENESIS
GENERATION_OF_NEURONS
DNA_REPLICATION
DNA_METABOLIC_PROCESS
DNA_REPAIR
DNA_TEMPLATED_DNA_REPLICATION
DNA_RECOMBINATION
CELLULAR_RESPONSE_TO_DNA_DAMAGE_STIMULUS
MITOTIC_CELL_CYCLE
SPINDLE_ORGANIZATION
REGULATION_OF_CELL_CYCLE_PROCESS
REGULATION_OF_CHROMOSOME_ORGANIZATION
MITOTIC_CELL_CYCLE_PHASE_TRANSITION
CELL_CYCLE_PHASE_TRANSITION
NEGATIVE_REGULATION_OF_CELL_CYCLE_PROCESS
CHROMOSOME_ORGANIZATION
REGULATION_OF_CELL_CYCLE
CELL_CYCLE_PROCESS
MITOTIC_CELL_CYCLE_PROCESS
CELL_CYCLE
DOUBLE_STRAND_BREAK_REPAIR
NEGATIVE_REGULATION_OF_CHROMOSOME_ORGANIZATION
NEGATIVE_REGULATION_OF_CELL_CYCLE
NEGATIVE_REGULATION_OF_NUCLEAR_DIVISION
MICROTUBULE_CYTOSKELETON_ORGANIZATION
MITOTIC_CELL_CYCLE_CHECKPOINT_SIGNALING
SISTER_CHROMATID_SEGREGATION
ORGANELLE_FISSION
REGULATION_OF_MITOTIC_NUCLEAR_DIVISION
REGULATION_OF_MITOTIC_CELL_CYCLE
REGULATION_OF_SISTER_CHROMATID_SEGREGATION
REGULATION_OF_NUCLEAR_DIVISION
CELL_CYCLE_CHECKPOINT_SIGNALING
CHROMOSOME_SEPARATION
REGULATION_OF_MITOTIC_SISTER_CHROMATID_SEGREGATION
REGULATION_OF_CELL_CYCLE_PHASE_TRANSITION
MITOTIC_SISTER_CHROMATID_SEPARATION
MITOTIC_SISTER_CHROMATID_SEGREGATION
MITOTIC_NUCLEAR_DIVISION
NUCLEAR_CHROMOSOME_SEGREGATION
POSITIVE_REGULATION_OF_CELL_CYCLE
POSITIVE_REGULATION_OF_CELL_CYCLE_PROCESS
CHROMOSOME_SEGREGATION
CELL_DIVISION
MITOTIC_SPINDLE_ORGANIZATION
MICROTUBULE_CYTOSKELETON_ORGANIZATION_INVOLVED_IN_MITOSIS
REGULATION_OF_CHROMOSOME_SEGREGATION
REGULATION_OF_MITOTIC_CELL_CYCLE_PHASE_TRANSITION
Enrichment
(-log10P)
0
50
B
GO:MF (Molecular Function)
Clusters
c0 c1 c2 c3 c4 c5
CHROMATIN_BINDING
DNA_HELICASE_ACTIVITY
SINGLE_STRANDED_DNA_BINDING
CATALYTIC_ACTIVITY_ACTING_ON_DNA
HELICASE_ACTIVITY
ATP_DEPENDENT_ACTIVITY_ACTING_ON_DNA
ATP_HYDROLYSIS_ACTIVITY
ATP_DEPENDENT_ACTIVITY
SINGLE_STRANDED_DNA_HELICASE_ACTIVITY
GATED_CHANNEL_ACTIVITY
GO:CC (Cellular Component)
Clusters
c0 c1 c2 c3 c4 c5
SPINDLE_POLE
CENTROSOME
SPINDLE
CHROMATIN
MICROTUBULE_ORGANIZING_CENTER
MICROTUBULE_CYTOSKELETON
MITOTIC_SPINDLE
CONDENSED_CHROMOSOME_CENTROMERIC_REGION
CONDENSED_CHROMOSOME
CHROMOSOME_CENTROMERIC_REGION
CHROMOSOME
NUCLEAR_CHROMOSOME
CHROMOSOMAL_REGION
OUTER_KINETOCHORE
SUPRAMOLECULAR_COMPLEX
DNA_REPLICATION_PREINITIATION_COMPLEX
CELL_SURFACE
EXTERNAL_ENCAPSULATING_STRUCTURE
COLLAGEN_CONTAINING_EXTRACELLULAR_MATRIX
ANCHORING_JUNCTION
CELL_BODY
SYNAPSE
AXON
NEURON_PROJECTION
PRESYNAPSE
SOMATODENDRITIC_COMPARTMENT
DENDRITIC_TREE
PRESYNAPTIC_MEMBRANE
PLASMA_MEMBRANE_REGION
POSTSYNAPTIC_MEMBRANE
SYNAPTIC_MEMBRANE
POSTSYNAPTIC_SPECIALIZATION
POSTSYNAPSE
GABA_ERGIC_SYNAPSE
NEURON_TO_NEURON_SYNAPSE
GLUTAMATERGIC_SYNAPSE
Enrichment
(-log10P)
0
20
C
451Lu-R corin/DMSO
1205Lu-R corin/DMSO
BIRC5
BRCA1
CCNA2
CDK2
CDK4
E2F1
E2F2
E2F3
MYC
FOXM1
2
1
0
-1
Log2 fold
D
451Lu-R
DMSO
corin
Fold expression normalized to DMSO
1.5
1.0
0.5
0.0
BIRC5
BRCA1
CCNA2
CDK2
CDK4
E2F1
E2F2
E2F3
MYC
1205Lu-R
E
451Lu-R corin/DMSO
1205Lu-R corin/DMSO
MITF
AXL
ZEB1
VINCULIN
COL4A2
THBS1
THBS2
PDGFRB
MYO1D
GABARAPL1
P2RX4
SYNGR1
SEMA4A
SEMA4D
PLXNA4
DUSP1
DUSP5
Focal Adhesion
Neural differentiation/ Axon guidance
4
2
0
-2
-4
Log2 fold
F
451Lu-R
DMSO
corin
Fold expression normalized to DMSO
100
80
60
40
20
5
4
3
2
1
0
MITF
AXL
VINCULIN
PDGFRB
COL4A2
PLXNA4
SEMA4D
SEMA4A
1205Lu-R
10
8
6
4
2
0

Supplemental Figure 2. Corin +/- PLX4032 differentially regulates signaling pathways in BRAFi-R melanomas with specific changes in cell cycle, focal adhesion, and neural differentiation gene sets. (A-B) Gene ontology analysis of k-means clusters shown in main Figure 3B. Significantly enriched ontologies with hypergeometric P < 1e-20 for biological processes (BP) (**A**) or P < 1e-10 for molecular function (MF) and cellular component (CC) (**B**) are shown. **(C)** heatmap of RNA-seq data illustrating gene expression patterns associated with cell cycle/DNA repair in 451Lu-R vs 1205Lu-R melanoma cells following corin treatment (2.5 µM, 24 h). **(D)** qPCR validated expression of differentially expressed genes from RNA-seq data associated with cell cycle/DNA repair in 451Lu-R and 1205Lu-R melanoma cells following corin treatment (2.5 µM, 24 h). **(E)** heatmap of RNA-seq data illustrating gene expression patterns associated with focal adhesions and neural differentiation/axon guidance in 451Lu-R and 1205Lu-R melanoma cells following corin treatment (2.5 µM, 24 h). **(F)** qPCR validated expression of differentially expressed genes from RNA-seq data associated with focal adhesions and neural differentiation/axon guidance in 451Lu-R and 1205Lu-R following corin treatment (2.5 µM, 24h). Significance asterisks indicate unpaired, two-tailed student's *t* test. All data represents mean + SE. *$P < 0.05$, **$P < 0.01$, ***$P < 0.001$, ****$P < 0.0001$.

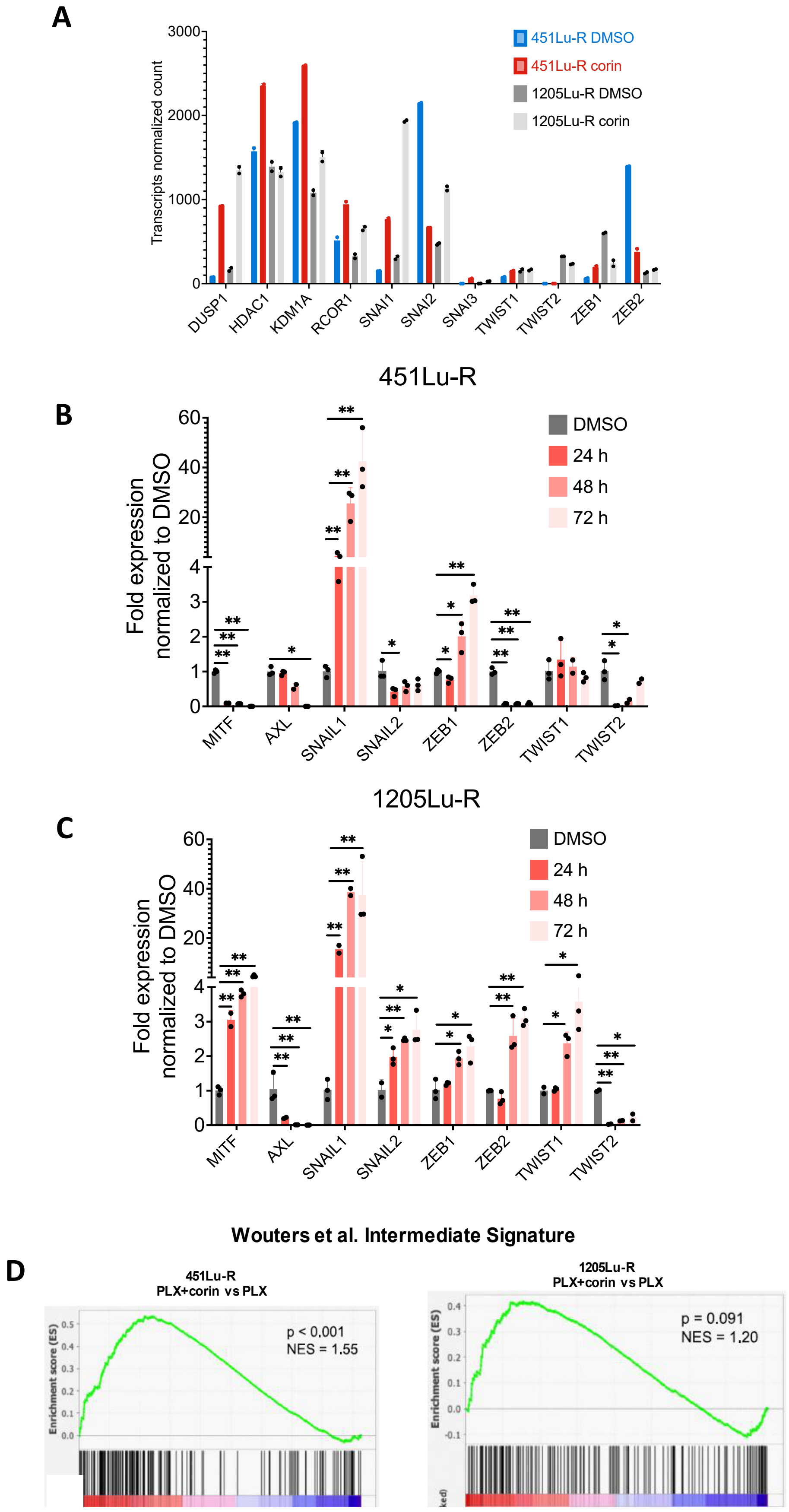
A
3000
2000
1000
0
Transcripts normalized count
451Lu-R DMSO
451Lu-R corin
1205Lu-R DMSO
1205Lu-R corin
DUSP1
HDAC1
KDM1A
RCOR1
SNAI1
SNAI2
SNAI3
TWIST1
TWIST2
ZEB1
ZEB2
451Lu-R
B
Fold expression normalized to DMSO
DMSO
24 h
48 h
72 h
MITF
AXL
SNAIL1
SNAIL2
ZEB1
ZEB2
TWIST1
TWIST2
1205Lu-R
C
Wouters et al. Intermediate Signature
D
451Lu-R
PLX+corin vs PLX
p < 0.001
NES = 1.55
1205Lu-R
PLX+corin vs PLX
p = 0.091
NES = 1.20
Enrichment score (ES)

Supplemental Figure 3. Corin impacts the expression of EMT-related genes and induces an intermediate phenotype gene expression signature. (A) Raw RNA-seq transcript counts of EMT transcription factors in 451Lu-R and 1205Lu-R melanoma cells following corin treatment (2.5 µM, 24 h). **(B, C)** qPCR validation of differentially expressed genes from RNA-seq data associated with the phenotype switch and EMT in 451Lu-R (**B**) and 1205Lu-R (**C**) melanoma cells following corin treatment (2.5 µM; 24h, 48h, and 72h). **(D)** Corin-induced intermediate phenotype gene expression signature, defined in Wouters et al. (17), in 451Lu-R (left) and 1205Lu-R (right) melanoma cells treated with 5 µM PLX4032 versus 2.5 µM corin + 5 µM PLX4032 for 24 h. Significance asterisks indicate 1-way ANOVA with Turkey's test. All data represents mean + SD. **P* < 0.05, ** *P* < 0.01.

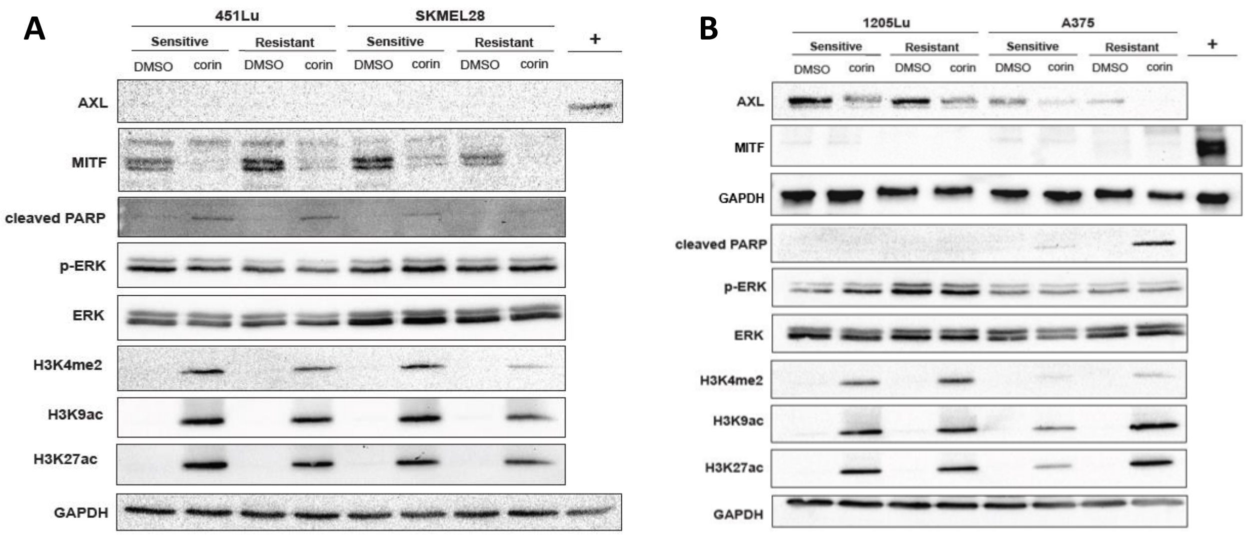
A
451Lu
SKMEL28
Sensitive
Resistant
Sensitive
Resistant
+
DMSO
corin
DMSO
corin
DMSO
corin
DMSO
corin
AXL
MITF
cleaved PARP
p-ERK
ERK
H3K4me2
H3K9ac
H3K27ac
GAPDH
B
1205Lu
A375
Sensitive
Resistant
Sensitive
Resistant
+
DMSO
corin
DMSO
corin
DMSO
corin
DMSO
corin
AXL
MITF
GAPDH
cleaved PARP
p-ERK
ERK
H3K4me2
H3K9ac
H3K27ac
GAPDH

Supplemental Figure 4. Corin mediates phenotype switching in both BRAFi-sensitive and -resistant melanoma cell pairs. (A, B) Western blot analysis of MAPK pathway activity in BRAFi-sensitive and -resistant melanoma cell lines treated with 2.5 μM corin for 24 h (**A**), in 451Lu and SKMel28 $MITF^{high}/AXL^{low}$ melanoma cell lines with 1205Lu-R lysates run as a positive control for AXL (Western blots were run contemporaneously) and (**B**), 1205Lu and A375 $MITF^{low}/AXL^{high}$ melanoma cell lines with 451Lu-R cell lysates run as a positive control for MITF. The 8-lane Western blots with same loading control were run contemporaneously.

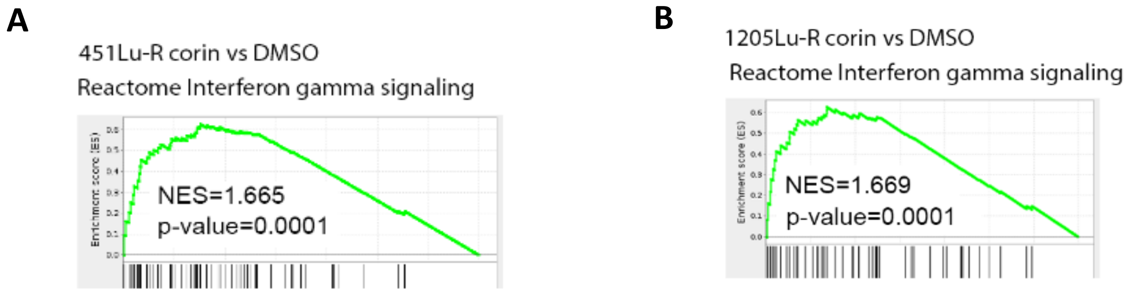
A
451Lu-R corin vs DMSO
Reactome Interferon gamma signaling
NES=1.665
p-value=0.0001
B
1205Lu-R corin vs DMSO
Reactome Interferon gamma signaling
NES=1.669
p-value=0.0001

Supplemental Figure 5. Corin treatment of melanoma cells promotes interferon gamma expression of gene signatures in 451Lu-R and 1205Lu-R cells. **(A, B)** GSEA analysis in corin (2.5 µM, 24 h) versus DMSO-treated melanoma cells illustrating enriched Interferon gamma signaling in 451Lu-R (**A**) and 1205Lu-R (**B**) melanoma cells. NES (Normalized Enrichment Score), (FDR < 0.05).

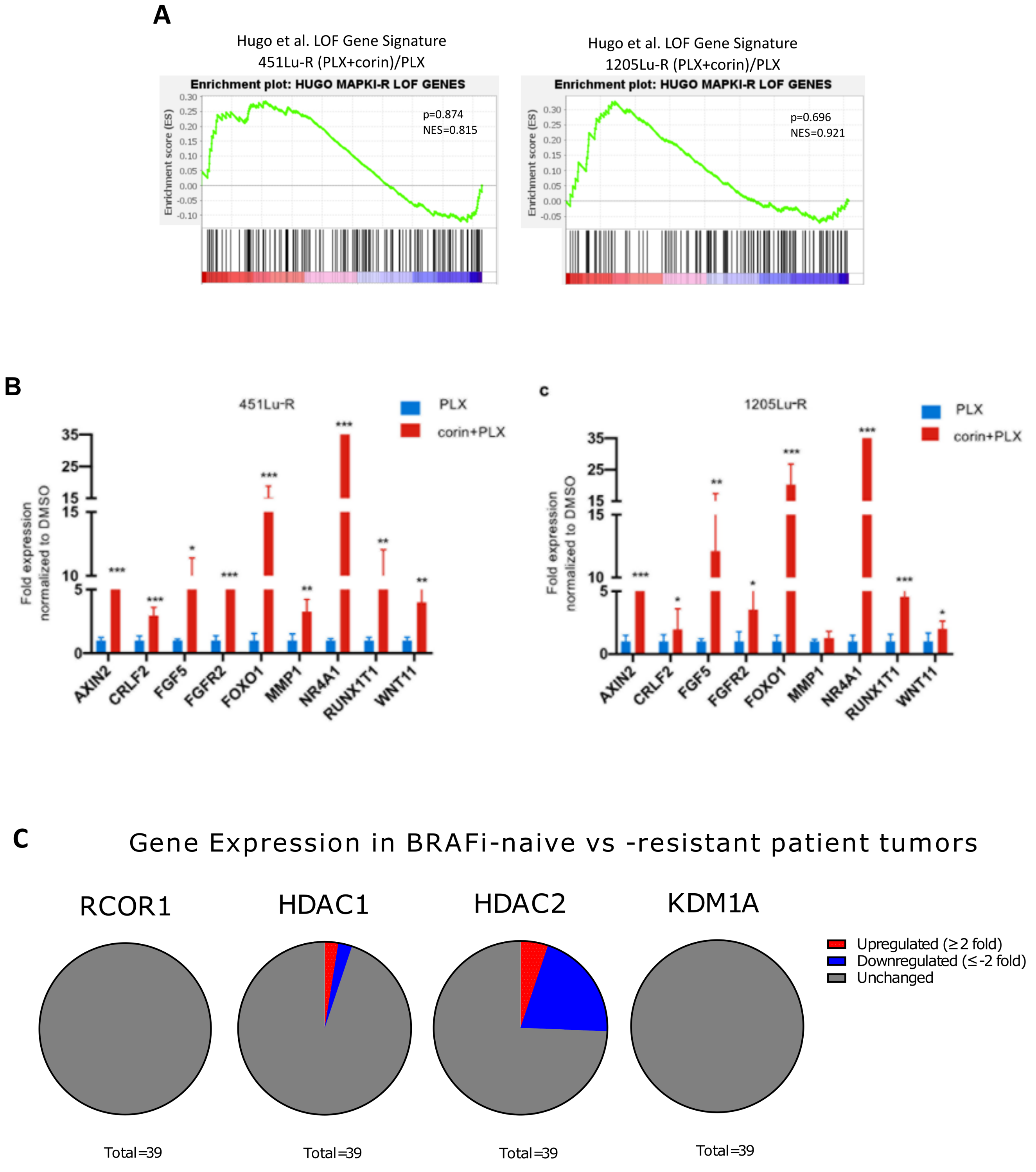
A
Hugo et al. LOF Gene Signature
451Lu-R (PLX+corin)/PLX
Enrichment plot: HUGO MAPKI-R LOF GENES
p=0.874
NES=0.815
Enrichment score (ES)
Hugo et al. LOF Gene Signature
1205Lu-R (PLX+corin)/PLX
Enrichment plot: HUGO MAPKI-R LOF GENES
p=0.696
NES=0.921
B
451Lu-R
PLX
corin+PLX
Fold expression normalized to DMSO
AXIN2
CRLF2
FGF5
FGFR2
FOXO1
MMP1
NR4A1
RUNX1T1
WNT11
c
1205Lu-R
C
Gene Expression in BRAFi-naive vs -resistant patient tumors
RCOR1
HDAC1
HDAC2
KDM1A
Upregulated (≥2 fold)
Downregulated (≤-2 fold)
Unchanged
Total=39

Supplemental Figure 6. Genes downregulated during the acquisition of MAPKi-resistance in melanoma patients are induced following corin treatment. (**A**) Corin-induced acquired MAPKi resistance LOF gene signature, defined in Hugo et al. (28), in 451Lu-R (left) and 1205Lu-R (right) melanoma cells treated with 2.5 μM corin + 5 μM PLX4032 versus 5 μM PLX4032 for 24 h. **(B)** qPCR validation of genes downregulated during acquired MAPKi-resistance in human melanomas which are induced following 24 h treatment with corin + PLX4032 (5 μM/2.5 μM) vs. PLX4032 alone (5 μM) in 451Lu-R (**C**) and 1205Lu-R (**D**) melanoma cells. (**C)** RCOR1, HDAC1, HDAC2, and KDM1A expression in BRAFi-resistant tumors compared to baseline analyzed with data from the validation microarray dataset (GSE50509 and GSE61992) used by Song et al.[36] Significance asterisks indicate unpaired, two-tailed student's *t* test. All data represents mean + SD. *$P < 0.05$, **$P < 0.01$, ***$P < 0.001$.

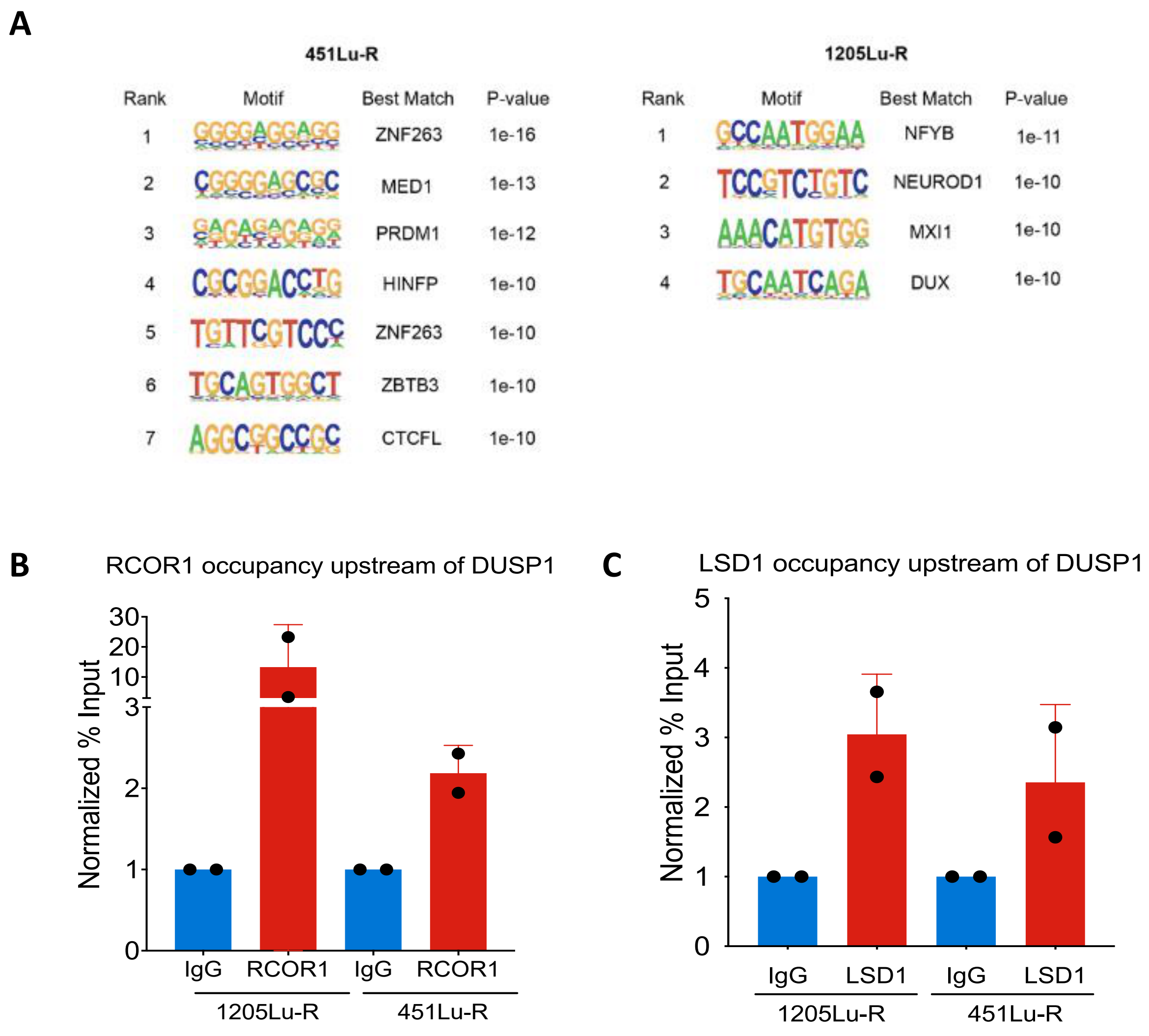
A
451Lu-R
Rank
Motif
Best Match
P-value
1 ZNF263 1e-16
2 MED1 1e-13
3 PRDM1 1e-12
4 HINFP 1e-10
5 ZNF263 1e-10
6 ZBTB3 1e-10
7 CTCFL 1e-10
1205Lu-R
Rank
Motif
Best Match
P-value
1 NFYB 1e-11
2 NEUROD1 1e-10
3 MXI1 1e-10
4 DUX 1e-10
B
RCOR1 occupancy upstream of DUSP1
Normalized % Input
30
20
10
3
2
1
0
IgG
RCOR1
IgG
RCOR1
1205Lu-R
451Lu-R
C
LSD1 occupancy upstream of DUSP1
Normalized % Input
5
4
3
2
1
0
IgG
LSD1
IgG
LSD1
1205Lu-R
451Lu-R

Supplemental Figure 7. The CoREST complex directly binds upstream of the DUSP1 promotor. (A) Top *de novo* motifs enriched in corin-upregulated genes in 451Lu-R (left) and 1205Lu-R (right) melanoma cells. (**B, C**) ChIP-qPCR analysis of RCOR1 (**B**) and LSD1 (**C**) occupancy upstream the DUSP1 promoter region in 1205Lu-R and 451Lu-R cells compared to IgG control (n=2).

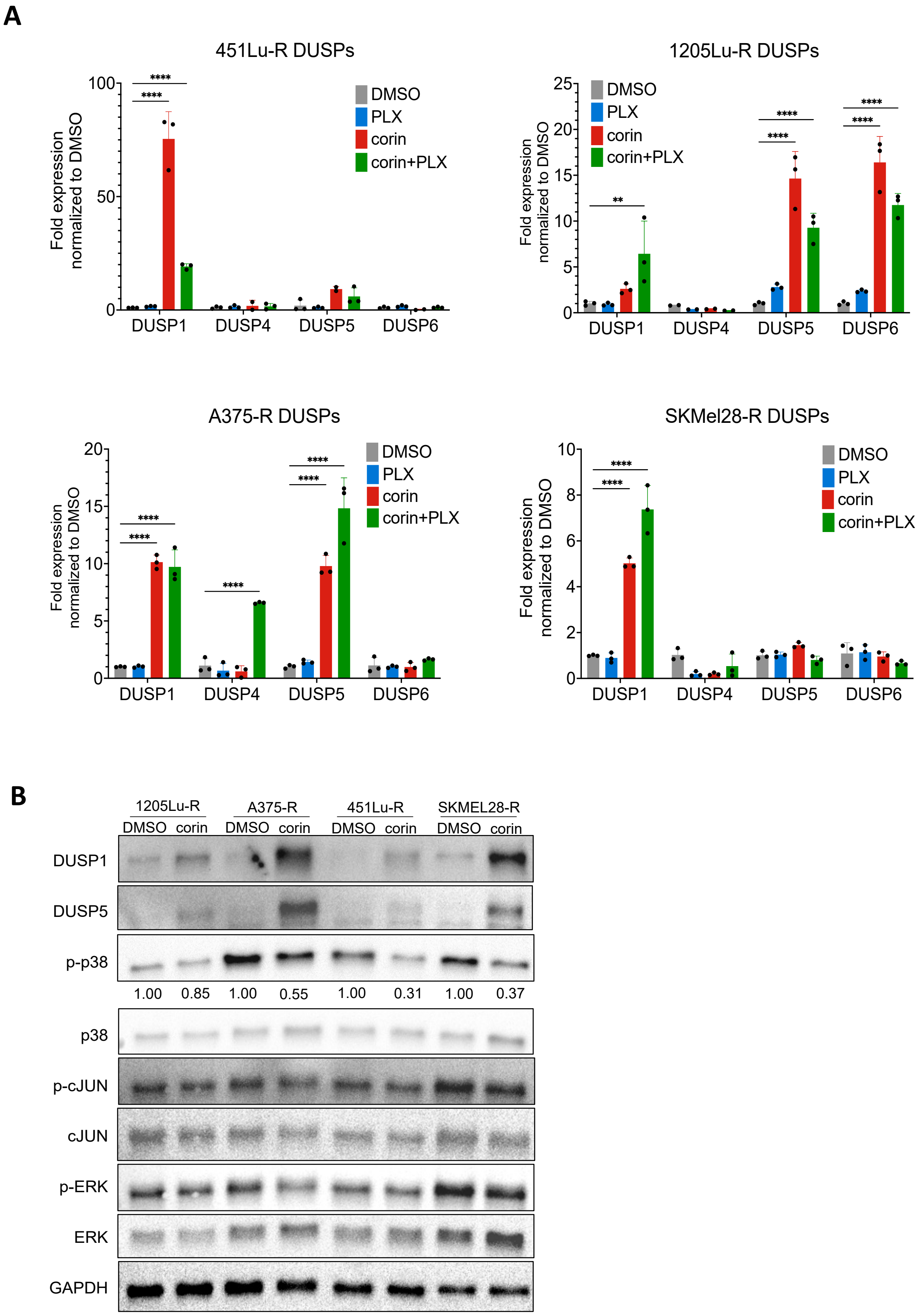
A
451Lu-R DUSPs
Fold expression normalized to DMSO
DMSO
PLX
corin
corin+PLX
DUSP1
DUSP4
DUSP5
DUSP6
1205Lu-R DUSPs
A375-R DUSPs
SKMel28-R DUSPs
B
1205Lu-R
A375-R
451Lu-R
SKMEL28-R
DMSO
corin
DUSP1
DUSP5
p-p38
1.00 0.85 1.00 0.55 1.00 0.31 1.00 0.37
p38
p-cJUN
cJUN
p-ERK
ERK
GAPDH

Supplemental Figure 8. Corin mediates re-sensitization to BRAFi therapy through DUSP1-associated inhibition of p38 MAPK in melanoma cells. **(A)** qPCR analysis of DUSP1, 4, 5, and 6 expression in 451Lu-R, 1205Lu-R, A375-R and SKMel28-R melanoma cells following 24 h treatment with 5 μM PLX4032, +/- 2.5 μM corin. **(B)** Western blot analysis of DUSP expression and MAPK-associated proteins in 1205Lu-R, A375-R, 451Lu-R and SKMel28-R melanoma cells treated with 2.5 μM corin for 48 hours. Quantification of relative expression of p-p38 (active) versus p38 (total) protein expression relative to the DMSO control is shown below each p-p38 band. Western blots were run contemporaneously. Significance asterisks indicate 1-way ANOVA with Turkey's test. All data represents mean + SD. ***P* < 0.01, *****P* < 0.0001.

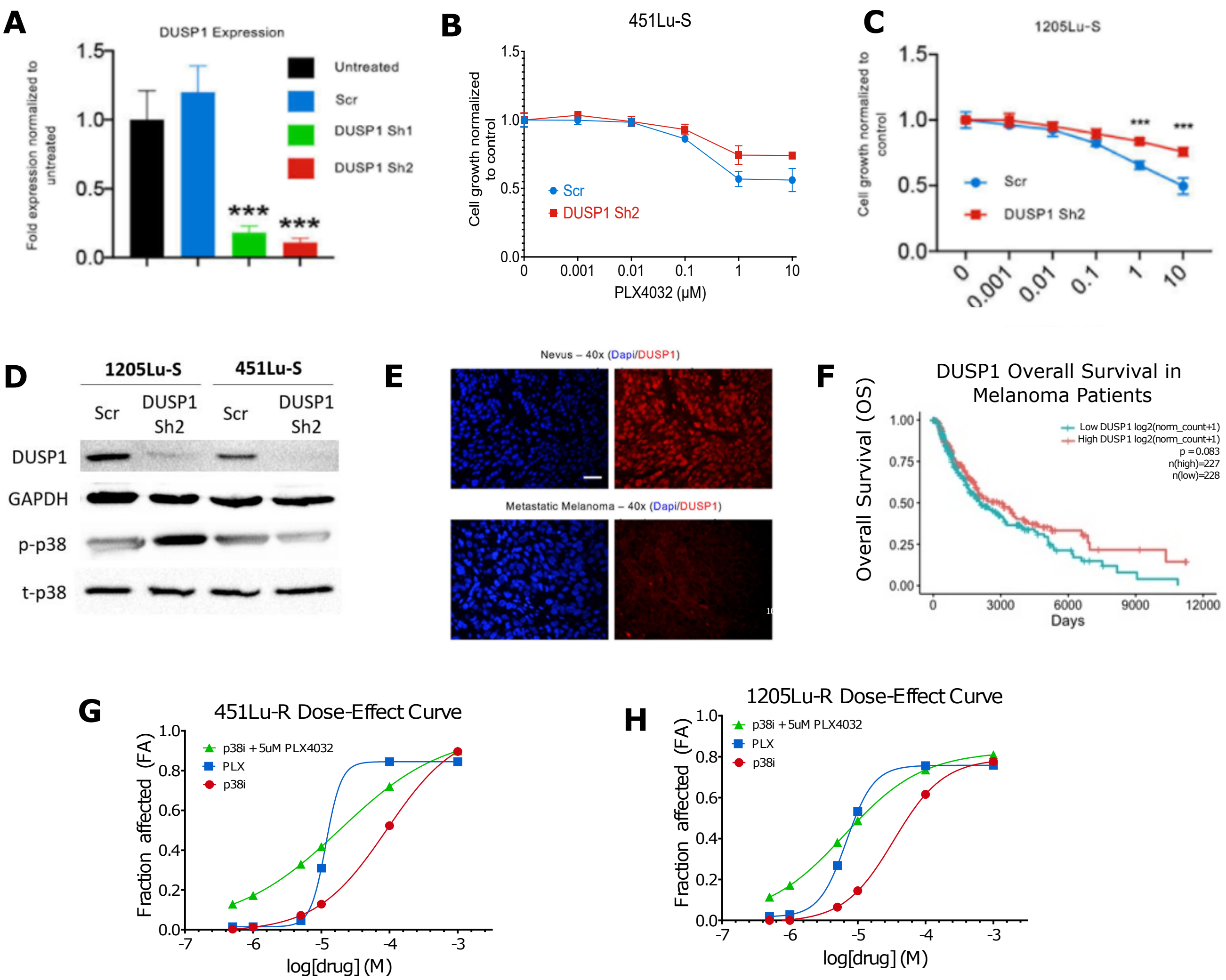

A
DUSP1 Expression
Fold expression normalized to untreated
Untreated
Scr
DUSP1 Sh1
DUSP1 Sh2
***
B
451Lu-S
Cell growth normalized to control
Scr
DUSP1 Sh2
PLX4032 (μM)
C
1205Lu-S
Cell growth normalized to control
Scr
DUSP1 Sh2
D
1205Lu-S
451Lu-S
Scr
DUSP1 Sh2
DUSP1
GAPDH
p-p38
t-p38
E
Nevus – 40x (Dapi/DUSP1)
Metastatic Melanoma – 40x (Dapi/DUSP1)
F
DUSP1 Overall Survival in Melanoma Patients
Overall Survival (OS)
Low DUSP1 log2(norm_count+1)
High DUSP1 log2(norm_count+1)
p = 0.083
n(high)=227
n(low)=228
Days
G
451Lu-R Dose-Effect Curve
Fraction affected (FA)
p38i + 5uM PLX4032
PLX
p38i
log[drug] (M)
H
1205Lu-R Dose-Effect Curve
Fraction affected (FA)
p38i + 5uM PLX4032
PLX
p38i
log[drug] (M)

Supplemental Figure 9. DUSP1 mediates melanoma cell growth, response to BRAF inhibitor therapy and p38 MAPK activity and is downregulated in human melanomas versus benign nevi. **(A)** DUSP1 knockdown by shRNA lentivirus in 1205Lu cells, confirmed by qRT-PCR. **(B, C)** Cell growth assays of 451Lu and 1205Lu melanoma cells treated with PLX4032 (72 h) following knockdown of DUSP1 versus scramble control, (n=3). **(D)** Western blot analysis of DUSP1 and pp38/p38 expression in 1205Lu-S and 451Lu-S melanoma cells following knockdown of DUSP1 versus scramble control. **(E)** Quantification of DUSP1 protein following DUSP1 knockdown vs scramble control. **(F)** Immunostain for DUSP1 expression in human melanoma specimens and benign nevi. Representative images shown, scale bar = 100 μm. **(E)** Kaplan-Meier curves illustrating the relationship between the expression of DUSP1 and overall survival in patient tumor specimens split at the median. Data obtained from the TCGA melanoma database (https://portal.gdc.cancer.gov). **(H, I)** Drug synergy graphs and Combination Index (CI) measurements for p38i and PLX4032 in 451Lu-R (**H**) and 1205Lu-R (**I**) BRAFi-R melanoma cells. Significance asterisks indicate 1-way ANOVA with Turkey's test. All data represents mean + SD. ****$P < 0.0001$.

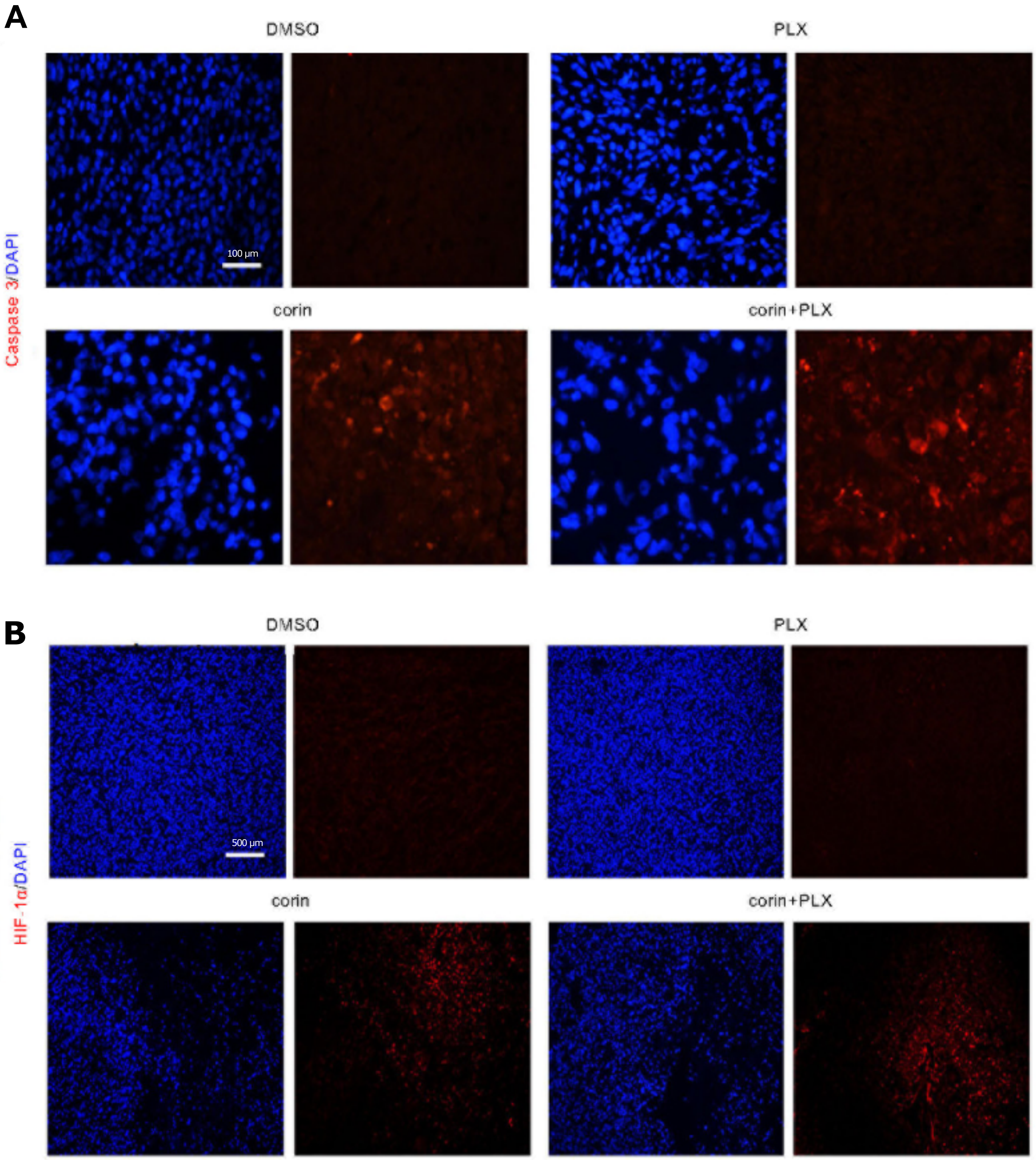
A
DMSO
PLX
Caspase 3/DAPI
100 µm
corin
corin+PLX
B
DMSO
PLX
HIF-1α/DAPI
500 µm
corin
corin+PLX

Supplemental Figure 10. Corin promotes apoptosis and hypoxia in BRAFi-resistant melanoma xenografts. (A, B) Immunofluorescence stain of cleaved caspase 3 (**A**) and HIF-1α (**B**) in 1205Lu-R melanoma xenografts treated with DMSO, PLX4032 alone, corin alone, or corin + PLX4032. Representative images shown, scale bar = 100 µm (**A**) or 500 µm (**B**).

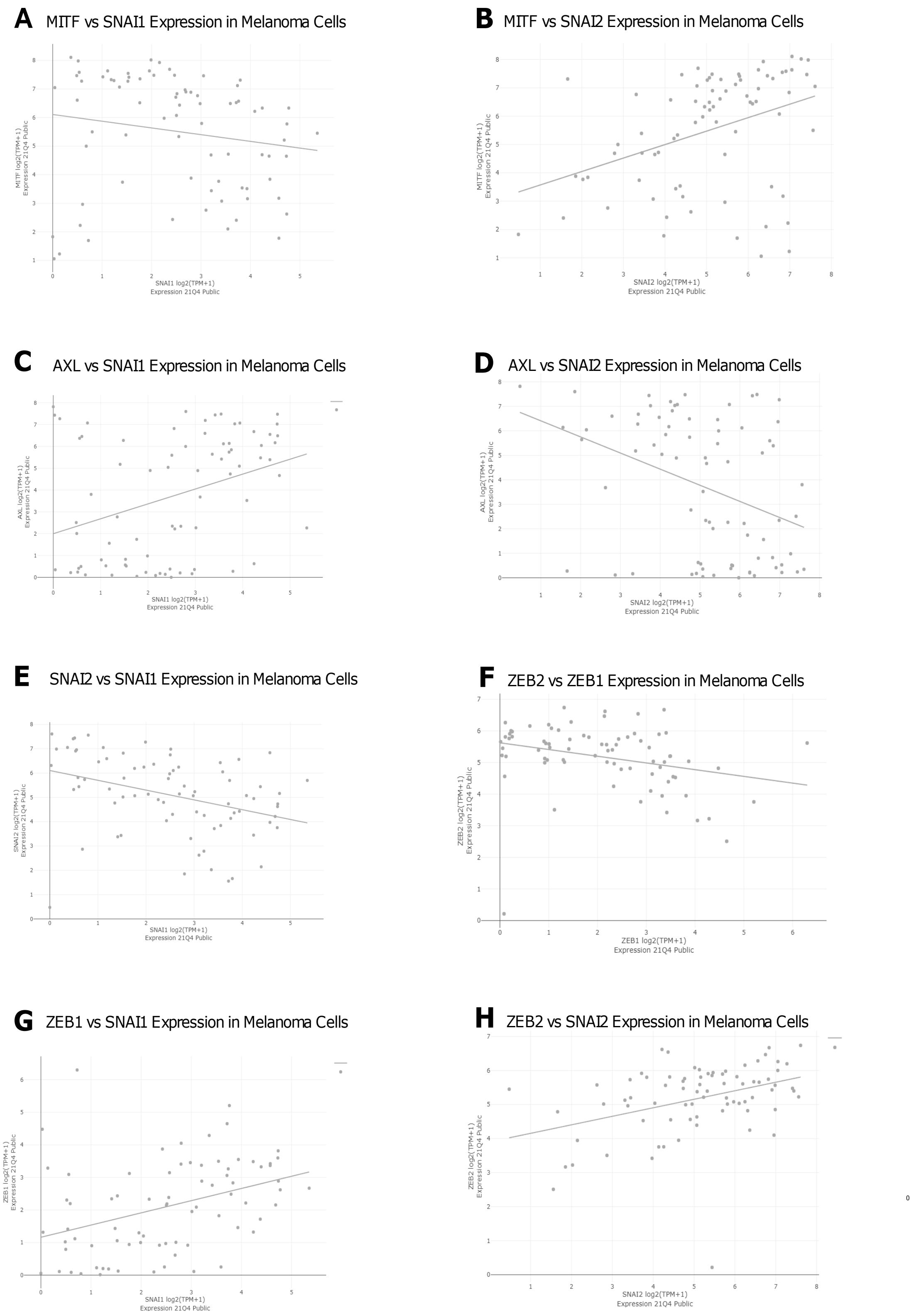
A
MITF vs SNAI1 Expression in Melanoma Cells
MITF log2(TPM+1)
Expression 21Q4 Public
SNAI1 log2(TPM+1)
Expression 21Q4 Public
B
MITF vs SNAI2 Expression in Melanoma Cells
MITF log2(TPM+1)
Expression 21Q4 Public
SNAI2 log2(TPM+1)
Expression 21Q4 Public
C
AXL vs SNAI1 Expression in Melanoma Cells
AXL log2(TPM+1)
Expression 21Q4 Public
SNAI1 log2(TPM+1)
Expression 21Q4 Public
D
AXL vs SNAI2 Expression in Melanoma Cells
AXL log2(TPM+1)
Expression 21Q4 Public
SNAI2 log2(TPM+1)
Expression 21Q4 Public
E
SNAI2 vs SNAI1 Expression in Melanoma Cells
SNAI2 log2(TPM+1)
Expression 21Q4 Public
SNAI1 log2(TPM+1)
Expression 21Q4 Public
F
ZEB2 vs ZEB1 Expression in Melanoma Cells
ZEB2 log2(TPM+1)
Expression 21Q4 Public
ZEB1 log2(TPM+1)
Expression 21Q4 Public
G
ZEB1 vs SNAI1 Expression in Melanoma Cells
ZEB1 log2(TPM+1)
Expression 21Q4 Public
SNAI1 log2(TPM+1)
Expression 21Q4 Public
H
ZEB2 vs SNAI2 Expression in Melanoma Cells
ZEB2 log2(TPM+1)
Expression 21Q4 Public
SNAI2 log2(TPM+1)
Expression 21Q4 Public

Supplemental Figure 11. Depmap analysis of EMT-associated transcription factors and their relevance to melanoma phenotypes in human melanoma cell lines. Survey of melanoma cell lines using the Broad Institute Depmap portal (https://depmap.org/portal/) **(A-H)** illustrating statistically significant inverse dependencies of expression of MITF with SNAI1 (**A**) and AXL with SNAI2 (**D**) as well as significant inverse dependencies of SNAI1 with SNAI2 (**E**) and ZEB1 with ZEB2 (**F**). The Depmap survey also found significant direct relationships between MITF and SNAI2 (**B**), AXL and SNAI1 (**C**) ZEB1 and SNAI1 (**G**) as well as ZEB2 and SNAI2 (**H**) in the melanoma cell lines evaluated.

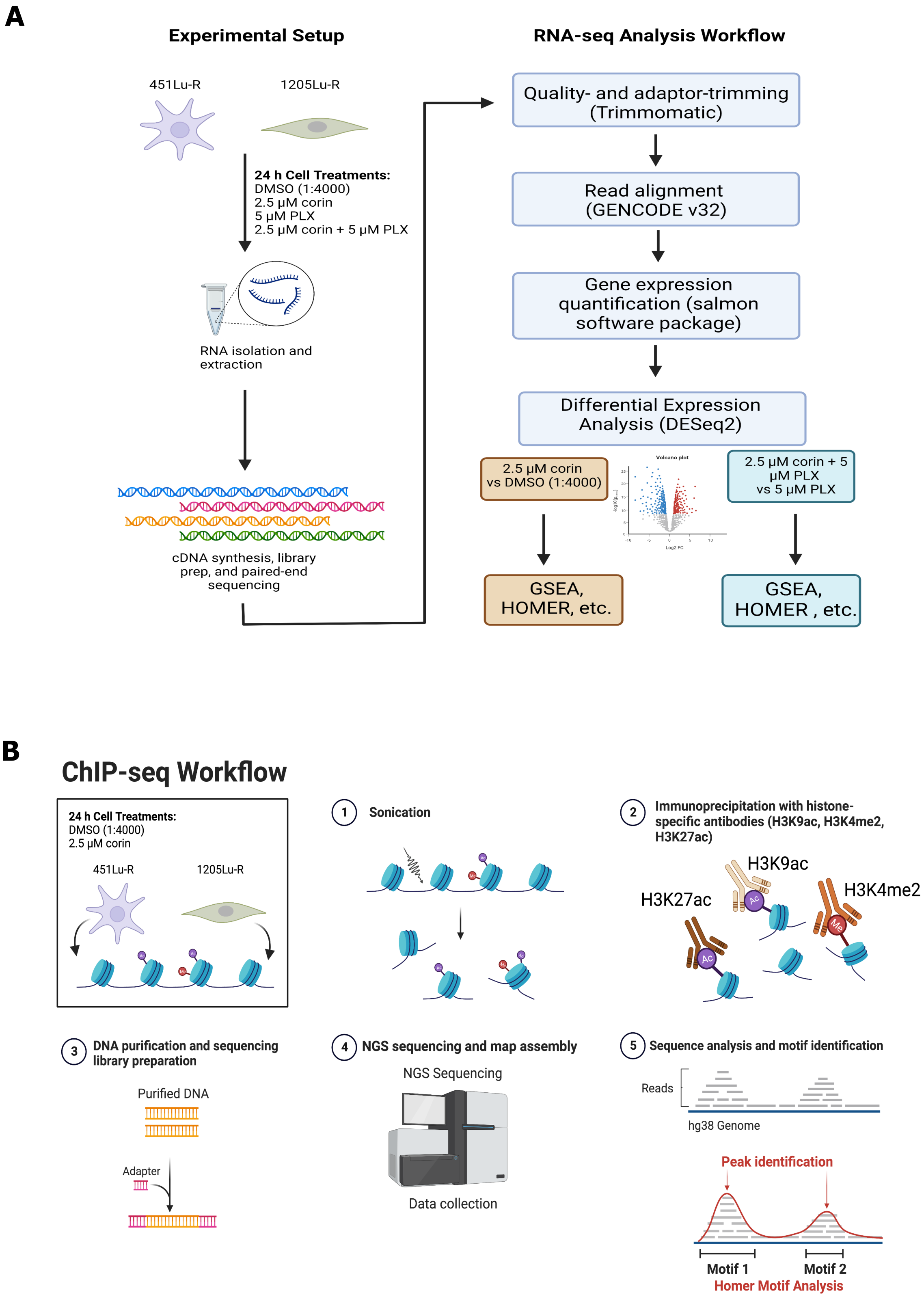
A
Experimental Setup
451Lu-R
1205Lu-R
24 h Cell Treatments:
DMSO (1:4000)
2.5 μM corin
5 μM PLX
2.5 μM corin + 5 μM PLX
RNA isolation and extraction
cDNA synthesis, library prep, and paired-end sequencing
RNA-seq Analysis Workflow
Quality- and adaptor-trimming (Trimmomatic)
Read alignment (GENCODE v32)
Gene expression quantification (salmon software package)
Differential Expression Analysis (DESeq2)
2.5 μM corin vs DMSO (1:4000)
Volcano plot
2.5 μM corin + 5 μM PLX vs 5 μM PLX
GSEA, HOMER, etc.
GSEA, HOMER , etc.
B
ChIP-seq Workflow
24 h Cell Treatments:
DMSO (1:4000)
2.5 μM corin
451Lu-R
1205Lu-R
1 Sonication
2 Immunoprecipitation with histone-specific antibodies (H3K9ac, H3K4me2, H3K27ac)
H3K9ac
H3K27ac
H3K4me2
3 DNA purification and sequencing library preparation
Purified DNA
Adapter
4 NGS sequencing and map assembly
NGS Sequencing
Data collection
5 Sequence analysis and motif identification
Reads
hg38 Genome
Peak identification
Motif 1
Motif 2
Homer Motif Analysis

Supplemental Figure 12. Experimental Workflows for RNA-seq (A) and ChIP-seq (B) studies. (A) Workflow schematic depicting RNA-sequencing experimental design. (**B**) Workflow schematic depicting chromatin immunoprecipitation-sequencing (ChIP-seq) experimental design. Created with BioRender.

Supplemental Table 1. IC50 values of BRAFi -sensitive vs -resistant 451Lu and 1205Lu cells.

Cell Line	LogIC50	IC50 (nM)
451Lu	-6.457	350
451Lu-R	-5.662	2177
1205Lu	-6.417	382
1205Lu-R	-5.711	1943

Supplemental Table 2. Combinations Index (CI) for A375-R cells treated with PLX4032 and corin. CIs indicate the degree of drug synergy where CI >1 indicates drug antagonism, CI=1 indicates drug additivity, and CI <1 indicates drug synergy for cell proliferation across a range of PLX and corin concentrations following 72 h treatment.

**PLX (µM)**	**corin (µM)**	A375-R Combination Index (CI)
1.0	0.1	40.52
1.0	0.5	4.12
1.0	1.0	1.72
1.0	5.0	0.06*
1.0	10	0.01*
1.0	100	7.43E-04*

Supplemental Table 3. Top leading edge genes of Reactome Interferon gamma signaling in 451Lu-R cells.

Gene	451Lu-R corin vs DMSO Log2foldChange	451Lu-R corin vs DMSO padj
HLA-DRA	9.622	3.30E-10
CAMK2B	7.493	6.72E-06
OASL	7.044	4.06E-05
HLA-DRB1	6.251	5.14E-08
TRIM29	5.935	2.31E-03
GBP5	4.834	9.49E-03
HLA-DQA2	4.798	4.82E-02
HLA-DRB5	4.778	5.05E-02
HLA-DPB1	4.483	3.99E-05
HLA-DQA1	4.116	4.64E-02

Supplemental Table 4. Top leading edge genes of Reactome Interferon gamma signaling in 1205Lu-R cells.

Gene	1205Lu-R corin vs DMSO Log2foldChange	1205Lu-R corin vs DMSO padj
HLA-DRB1	6.1749	7.46E-04
HLA-DQB1	6.0516	2.23E-04
GBP5	4.7505	1.89E-04
TRIM48	4.7095	5.10E-02
TRIM10	4.2114	1.18E-01
GBP2	4.0186	1.05E-41
HLA-DRA	3.5233	4.28E-17
HLA-DPA1	3.3493	3.66E-02
HLA-DPB1	3.2818	1.65E-01
IRF5	3.1357	1.06E-02

Supplementary Table 5. GSEA analysis of type I and type II IFN pathways for 451Lu-R and 1205Lu-R cells treated with corin vs DMSO.

	451Lu-R corin vs DMSO_pval	451Lu-R corin vs DMSO_NES	1205Lu-R corin vs DMSO_pval	1205Lu-R corin vs DMSO_NES
Reactome Interferon gamma signaling (type II)	0.0001	1.6653	0.0001	1.6694
Reactome Interferon gamma signaling (type I)	0.2715	1.1184	0.7778	0.8674

Supplementary Table 6. GSEA analysis of type I and type II IFN pathways for 451Lu-R and 1205Lu-R cells treated with PLX+corin vs PLX.

	451Lu-R PLX+corin vs PLX_pval	451Lu-R PLX+corin vs PLX_NES	1205Lu-R PLX+corin vs PLX_pval	1205Lu-R PLX+corin vs PLX_NES
Reactome Interferon gamma signaling (type II)	1.0036E-05	1.7631	1.0E-05	1.5643
Reactome Interferon gamma signaling (type I)	0.0272	1.3393	0.9723	0.6593

Supplementary Table 7. Transcript changes of RISC components and repetitive elements induced by corin.

	451Lu-R corin vs DMSO log2FoldChange	451Lu-R corin vs DMSO pvalue	1205Lu-R corin vs DMSO log2FoldChange	1205Lu-R corin vs DMSO pvalue
DICER1	-0.254	5.38E-02	-0.234	9.94E-02
AGO2	-0.674	2.59E-10	-0.816	3.19E-30
ERV1	0.336	1.28E-47	0.751	0
ERVL	0.286	3.74E-26	0.437	4.12E-64

Supplementary Table 8. Expression of genes downregulated in patient melanomas during acquired MAPKi resistance. Genes repressed in MAPKi-resistant melanoma specimens, reported by Hugo et al. (28), and associated level of induced expression following 24h treatment with corin + PLX4032 (2.5 µM/5 µM) versus PLX4032 alone (5 µM) in 451Lu-R and 1205Lu-R melanoma cells.

Gene	451Lu-R (PLX+corin)/PLX Log2FoldChange	1205Lu-R (PLX+corin)/PLX Log2FoldChange
AXIN2	3.02	3.22
CRLF2	7.52	4.13
CTNNA2	6.53	3.87
FGF12	5.93	2.29
FGF5	7.11	6.46
FGFR2	2.97	5.63
FOXO1	2.63	2.90
MMP1	13.73	4.02
NR4A1	3.30	4.19
PLD1	2.06	2.00
RUNX1T1	6.42	2.82
SIRT4	2.32	3.32
WNT11	7.47	6.98

Supplementary Table 9. MOTIFs enriched with corin treatment in 451Lu-R H3K9ac ChIPseq.

Motif name	Consensu	Log P-value	% of Target Sequences with Motif
**Mef2c(MADS)/GM12878-Mef2c-ChIP-Seq(GSE32465)/Homer**	DCYAAAAATAGM	-1640	0.6639
Mef2a(MADS)/HL1-Mef2a.biotin-ChIP-Seq(GSE21529)/Homer	CYAAAAATAG	-1495	0.6287
IDD5(C2H2)/colamp-IDD5-DAP-Seq(GSE60143)/Homer	TTTTGTCTTTTTBTK	-1467	0.7511
REM19(REM)/colamp-REM19-DAP-Seq(GSE60143)/Homer	AAAAAAAA	-1385	0.7387
IDD4(C2H2)/col-IDD4-DAP-Seq(GSE60143)/Homer	TTTGTCTTTWTB	-1257	0.7691
GSC(Homeobox)/FrogEmbryos-GSC-ChIP-Seq(DRA000576)/Homer	RGGATTAR	-1135	0.8286
VRN1(ABI3VP1)/col-VRN1-DAP-Seq(GSE60143)/Homer	TTTTTTTTTT	-1105	0.5166
RLR1?/SacCer-Promoters/Homer	WTTTTCYYTTTT	-1077	0.6953
Mef2b(MADS)/HEK293-Mef2b.V5-ChIP-Seq(GSE67450)/Homer	GCTATTTTTGGM	-953.8	0.6848
GLIS3(Zf)/Thyroid-Glis3.GFP-ChIP-Seq(GSE103297)/Homer	CTCCCTGGGAGGCCN	-704.8	0.825
Bapx1(Homeobox)/VertebralCol-Bapx1-ChIP-Seq(GSE36672)/Homer	TTRAGTGSYK	-583.7	0.8914
LXRE(NR),DR4/RAW-LXRb.biotin-ChIP-Seq(GSE21512)/Homer	RGGTTACTANAGGTCA	-579.4	0.2808
Hoxd12(Homeobox)/ChickenMSG-Hoxd12.Flag-ChIP-Seq(GSE86088)/Homer	HDGYAATGAAAN	-500.1	0.7851
EAR2(NR)/K562-NR2F6-ChIP-Seq(Encode)/Homer	NRBCARRGGTCA	-417.5	0.701
Atf1(bZIP)/K562-ATF1-ChIP-Seq(GSE31477)/Homer	GATGACGTCA	-370.6	0.4496
COUP-TFII(NR)/K562-NR2F1-ChIP-Seq(Encode)/Homer	GKBCARAGGTCA	-356.1	0.7104
Gfi1b(Zf)/HPC7-Gfi1b-ChIP-Seq(GSE22178)/Homer	MAATCACTGC	-343	0.4783
CRX(Homeobox)/Retina-Crx-ChIP-Seq(GSE20012)/Homer	GCTAATCC	-341.5	0.8691
Snail1(Zf)/LS174T-SNAIL1.HA-ChIP-Seq(GSE127183)/Homer	TRCACCTGCY	-326.9	0.5628
NGA4(ABI3VP1)/col-NGA4-DAP-Seq(GSE60143)/Homer	TKNTCAGGTG	-307.2	0.8968
Atf7(bZIP)/3T3L1-Atf7-ChIP-Seq(GSE56872)/Homer	NGRTGACGTCAY	-301.6	0.3556
THRa(NR)/C17.2-THRa-ChIP-Seq(GSE38347)/Homer	GGTCANYTGAGGWCA	-290.8	0.4406
Nkx2.2(Homeobox)/NPC-Nkx2.2-ChIP-Seq(GSE61673)/Homer	BTBRAGTGSN	-274.9	0.8148
FEA4(bZIP)/Corn-FEA4-ChIP-Seq(GSE61954)/Homer	TGACGTCACS	-263	0.5483
COUP-TFII(NR)/Artia-Nr2f2-ChIP-Seq(GSE46497)/Homer	AGRGGTCA	-257.2	0.7303
SUT1?/SacCer-Promoters/Homer	CCCCGCGC	-205.7	0.9507

RARa(NR)/K562-RARa-ChIP-Seq(Encode)/Homer	TTGAMCTTTG	-198.2	0.9008
THRb(NR)/HepG2-THRb.Flag-ChIP-Seq(Encode)/Homer	GGTCACCTGAGGTCA	-159.4	0.4446
RIN(MADS)/Tomato-RIN-ChIP-Seq(GSE116581)/Homer	CYAAAAWWGG	-153.6	0.6319
Nkx2.1(Homeobox)/LungAC-Nkx2.1-ChIP-Seq(GSE43252)/Homer	RSCACTYRAG	-150.3	0.8709
Slug(Zf)/Mesoderm-Snai2-ChIP-Seq(GSE61475)/Homer	SNGCACCTGCHS	-146.3	0.382
Tbx5(T-box)/HL1-Tbx5.biotin-ChIP-Seq(GSE21529)/Homer	AGGTGTCA	-140.5	0.8724
ZEB2(Zf)/SNU398-ZEB2-ChIP-Seq(GSE103048)/Homer	GNMCAGGTGTGC	-129.2	0.4972
GAGA-repeat/SacCer-Promoters/Homer	CTYTCTYTCTCTCTC	-122.8	0.9163
E2A(bHLH),near_PU.1/Bcell-PU.1-ChIP-Seq(GSE21512)/Homer	NVCACCTGBN	-119.9	0.6404
Nkx3.1(Homeobox)/LNCaP-Nkx3.1-ChIP-Seq(GSE28264)/Homer	AAGCACTTAA	-119.2	0.8235
ZEB1(Zf)/PDAC-ZEB1-ChIP-Seq(GSE64557)/Homer	VCAGGTRDRY	-117.4	0.6786
E2A(bHLH)/proBcell-E2A-ChIP-Seq(GSE21978)/Homer	DNRCAGCTGY	-117.2	0.6339
GAGA-repeat/Arabidopsis-Promoters/Homer	CTCTCTCTCY	-113.9	0.533
Srebp2(bHLH)/HepG2-Srebp2-ChIP-Seq(GSE31477)/Homer	CGGTCACSCCAC	-111	0.1624
ZNF416(Zf)/HEK293-ZNF416.GFP-ChIP-Seq(GSE58341)/Homer	WDNCTGGGCA	-104.2	0.6069
Nkx2.5(Homeobox)/HL1-Nkx2.5.biotin-ChIP-Seq(GSE21529)/Homer	RRSCACTYAA	-91.15	0.7889
Bcl11a(Zf)/HSPC-BCL11A-ChIP-Seq(GSE104676)/Homer	TYTGACCASWRG	-90.9	0.4169
Npas4(bHLH)/Neuron-Npas4-ChIP-Seq(GSE127793)/Homer	NHRTCACGACDN	-90.1	0.4462
At1g14580(C2H2)/colamp-At1g14580-DAP-Seq(GSE60143)/Homer	CASAAAAMGACAAAA	-83.4	0.1947
Srebp1a(bHLH)/HepG2-Srebp1a-ChIP-Seq(GSE31477)/Homer	RTCACSCCAY	-78.4	0.1822
HEB(bHLH)/mES-Heb-ChIP-Seq(GSE53233)/Homer	VCAGCTGBNN	-60.12	0.6795
ASHR1(ND)/col-ASHR1-DAP-Seq(GSE60143)/Homer	NTGGTGAN	-57.48	0.5859

Supplementary Table 10. MOTIFs enriched with corin treatment in 451Lu-R H3K4me2 ChIPseq.

Motif Name	Consensus	Log P-value	% of Target Sequences with Motif
**Usf2(bHLH)/C2C12-Usf2-ChIP-Seq(GSE36030)/Homer**	GTCACGTGGT	-85.47	0.2711
Mef2a(MADS)/HL1-Mef2a.biotin-ChIP-Seq(GSE21529)/Homer	CYAAAAATAG	-65.04	0.4921
Mef2c(MADS)/GM12878-Mef2c-ChIP-Seq(GSE32465)/Homer	DCYAAAAATAGM	-64.62	0.5213
IDD5(C2H2)/colamp-IDD5-DAP-Seq(GSE60143)/Homer	TTTTGTCTTTTTBTK	-54.38	0.6152
GLIS3(Zf)/Thyroid-Glis3.GFP-ChIP-Seq(GSE103297)/Homer	CTCCCTGGGAGGCCN	-41.06	0.7061
EAR2(NR)/K562-NR2F6-ChIP-Seq(Encode)/Homer	NRBCARRGGTCA	-19.29	0.6261
VRN1(ABI3VP1)/col-VRN1-DAP-Seq(GSE60143)/Homer	TTTTTTTTTT	-19.17	0.4213
Reverb(NR),DR2/RAW-Reverba.biotin-ChIP-Seq(GSE45914)/Homer	GTRGGTCASTGGGTCA	-18.15	0.1083
p53(p53)/mES-cMyc-ChIP-Seq(GSE11431)/Homer	ACATGCCCGGGCAT	-15.05	0.0162
IDD4(C2H2)/col-IDD4-DAP-Seq(GSE60143)/Homer	TTTGTCTTTWTB	-14.83	0.6456
GSC(Homeobox)/FrogEmbryos-GSC-ChIP-Seq(DRA000576)/Homer	RGGATTAR	-7.876	0.7121
COUP-TFII(NR)/K562-NR2F1-ChIP-Seq(Encode)/Homer	GKBCARAGGTCA	-5.949	0.6393
SUT1?/SacCer-Promoters/Homer	CCCCGCGC	-5.016	0.8883
REST-NRSF(Zf)/Jurkat-NRSF-ChIP-Seq/Homer	GGMGCTGTCCATGGTGCTGA	-4.619	0.0043

Supplementary Table 11. Combinations Index (CI) for 451Lu-R cells treated with PLX4032 and p38i. CI data indicate the degree of drug synergy where CI >1 indicates drug antagonism, CI=1 indicates drug additivity, and CI <1 indicates drug synergy for cell proliferation inhibition across a range of PLX and p38i concentrations (72 h treatment).

PLX (µM)	p38i (µM)	Combination Index (CI)
5.0	0.5	1.82
5.0	1.0	0.93
5.0	5.0	0.27
5.0	10.0	0.27
5.0	100	1.18
5.0	1000	7.35

Supplementary Table 12. Combinations Index (CI) for 1205Lu-R cells treated with PLX4032 and p38i. CI data indicate the degree of drug synergy where CI >1 indicates drug antagonism, CI=1 indicates drug additivity, and CI <1 indicates drug synergy for cell proliferation inhibition across a range of PLX and p38i concentrations (72 h treatment).

PLX (μM)	p38i (μM)	Combination Index (CI)
5.0	0.5	4.72
5.0	1.0	1.76
5.0	5.0	0.20
5.0	10.0	0.10
5.0	100	0.22
5.0	1000	1.83

Supplementary Table 13. Antibody Information.

Antibody	Company	Catalog Number	Application	Dilution
**H3K9ac**	Abcam	ab32129	WB, ChIP	1:1000
**H3K4me2**	Abcam	ab32356	WB, ChIP, IF	1:5000
**H3K27ac**	Abcam	ab4729	WB, ChIP, IF	1:1000
**Phospho-MEK1/2**	Cell Signaling	9121	WB	1:1000
**MEK1/2**	Cell Signaling	9122	WB	1:1000
**Total H3**	Abcam	ab1791	WB	1:10000
**DUSP1**	Millipore	07-535	WB	1:1000
**DUSP5**	Abcam	ab200708	WB	1:1000
**Phospho-c-Jun (Ser63)**	Cell Signaling	9261s	WB	1:1000
**c-Jun**	Cell Signaling	9165s	WB	1:1000
**Phospho-Erk**	Cell Signaling	9101s	WB	1:1000
**p44/42 MAPK (Erk1/2)**	Cell Signaling	9102s	WB	1:1000
**MITF**	Cell Signaling	12590s	WB	1:1000
**AXL**	Cell Signaling	8661s	WB	1:1000
**Cleaved PARP**	Cell Signaling	9541s	WB	1:1000
**Phospho-JNK**	Cell Signaling	4668s	WB	1:1000
**JNK**	Cell Signaling	9252s	WB	1:1000
**Phospho-p38 MAPK**	Cell Signaling	4631s	WB	1:1000
**p38 MAPK**	Cell Signaling	9212s	WB	1:1000
**GAPDH**	Cell Signaling	2118L	WB	1:2000
**p62**	Cell signaling	88588s	WB	1:1000
**LC3B**	Cell signaling	2775S	WB	1:1000
**Ki67**	Abcam	ab15580	IF, IHC	1:500
**Cleaved Caspase-3**	Cell Signaling	9661s	IF, IHC	1:500
**HIF1 alpha**	Abcam	ab16066	IHC	1:500
**Vinculin**	Millipore	MAB3574	IF	1:300
**HDAC1**	Abcam	ab19845	ChIP	
**LSD1**	Abcam	ab129195	ChIP	
**RCOR1**	Millipore	07-455	ChIP	

Supplementary Table 14. List of primers and oligonucleotide sequences

RT-qPCR Primer Sequences		
**Target**	**Forward Primer (5'->3')**	**Reverse Primer (5'->3')**
AXIN2	GGATCACTGGCTCCGCGA	AGTTCCTCTCAGCAATCGGC
AXL	CCAGGACACCCCAGAGGTGCTAAT	TGGTGGACTGGCTGTGCTTGC
BIRC5	CCACTGAGAACGAGCCAGACTTG	AGAAAGGAAAGCGCAACCGG
BRCA1	GAATTTATCGAGTGGCCAAAC	TCAAAGACTTGACTGTTGTGG
CCNA2	GCATGTCACCGTTCCTCCTT	GGGCATCTTCACGCTCTATTT
CDK2	GCTTTTGGAGTCCCTGTTCG	GGTCCCCAGAGTCCGAAAGA
COL4A2	CCTGAAGGCACAGCTAACCA	TGCTGTTGTCTCGTCTGTCC
CRLF2	ACTCCTGTTTCAGGCATGGG	AGTCAGGTTGGTCCTGGAGT
DUSP1	AGGACAACCACAAGGCAGAC	CAGTGGACAAACACCCTTCC
DUSP4	CAAAGGCGGCTATGAG	GGTTATCTTCCACTGGG
DUSP5	GCCCGCGGGTCTACTTCCTC	CTCGGAGGTCCGTCGGGAGA
DUSP6	CGAGACCCCAATAGTGC	AATGGCCTCAGGGAAA
E2F1	AGGGGTGTGGGGTTGATACC	TCAGACACTGCAGGAGGGAC
E2F2	CCTTGGAGGCTACTGACAGC	CCACAGGTAGTCGTCCTGGT
FGF5	CTCTCTCTTCCCCTCTCCCC	TAGGGCTGATTCTGGGCTCT
FGFR2	CCTGCGGAGACAGGTAACAG	GGTGTCTGCCGTTGAAGAGA
FOXO1	GATCCCGTAAGTCGGGCGG	GCTGCTGCCTGTTGAATGTG
GAPDH	GGCTCTCCAGAACATCATCCCTGC	GGGTGTCGCTGTTGAAGTCAGAGG
MITF	GGAAATCTTGGGCTTGATGGA	CCCGAGACAGGCAACGTATT
MMP1	AAGGCCAGTATGCACAGCTT	GGGCCACTATTTCTCCGCTT
MYC	TCACCAGCACAACTACGCCG	CAGGATGTAGGCGGTGGCTT
NR4A1	GCTACGAAACTTGGGGGAGT	ATGTGGCTTGCACCTGTTCT
PDGFRB	CAAGGACACCATGCGGCTTC	AGCAGGTCAGAACGAAGGTG
PLXNA4	AATTCCCGTCTTCACGGAGG	AGTCACTGTTGCTCTGTGGG
RUNX1T1	GTCAAGAAGCAGACCGGGAA	CTAGTGCAACTGGGTCTGGG
SEMA4A	CGGGATGGGGTTGAGAATGG	ATCATGCCAAAGCCAGCACT
SEMA4D	CTGGGGCTCATCTCTAGCAC	CTCTCACCACCGCAATGTCA
VCL	GCTGCTGTTAATGCCATCCAA	CCAACCGAGTCACCTCATCT
WNT11	GTGAAGGACTCGGAACTCGT	GTCGCTTCCGTTGGATGTCT

CHIP-qPCR Primer Sequences		
**Target**	**Forward Primer (5'->3')**	**Reverse Primer (5'->3')**
Upstream DUSP1	TGAGAGGTGGGCCTCAGTTA	GAGGTTGCAGTGAGCTGAGA

shRNA Sequences	
**Target**	**Target Sequence (5'->3')**
RCOR1 Sh1	GATGGTGGAATAGAACCATAT
RCOR1 Sh2	CAAACGACAGATCCAGAATAT
DUSP1 Sh1	GCCGCTCCTTCTTCGCTTTCA
DUSP1 Sh2	ACGAGGCCATTGACTTCATAG
DUSP1 Sh3	GAGGGTCACTACCAGTACAAG
DUSP1 Sh4	GCTGATTATTTATGACCTGAA
DUSP1 Sh5	GCTCTGTCAACGTGCGCTTCA
