# Supplementary material for: The CoREST repressor complex mediates phenotype switching and therapy resistance in melanoma
Source: J Clin Invest. 2024 Feb 1;134(6):e171063. doi: 10.1172/JCI171063 (PMC10940100; doi:10.1172/JCI171063)
Supplement: Unedited blot and gel images [file jci-134-171063-s111.pdf]

The CoREST repressor complex mediates phenotype switching and therapy resistance in melanoma (171063-JCI-RG-RV-2)

Unedited blot images

Full unedited gel for Figure 1A

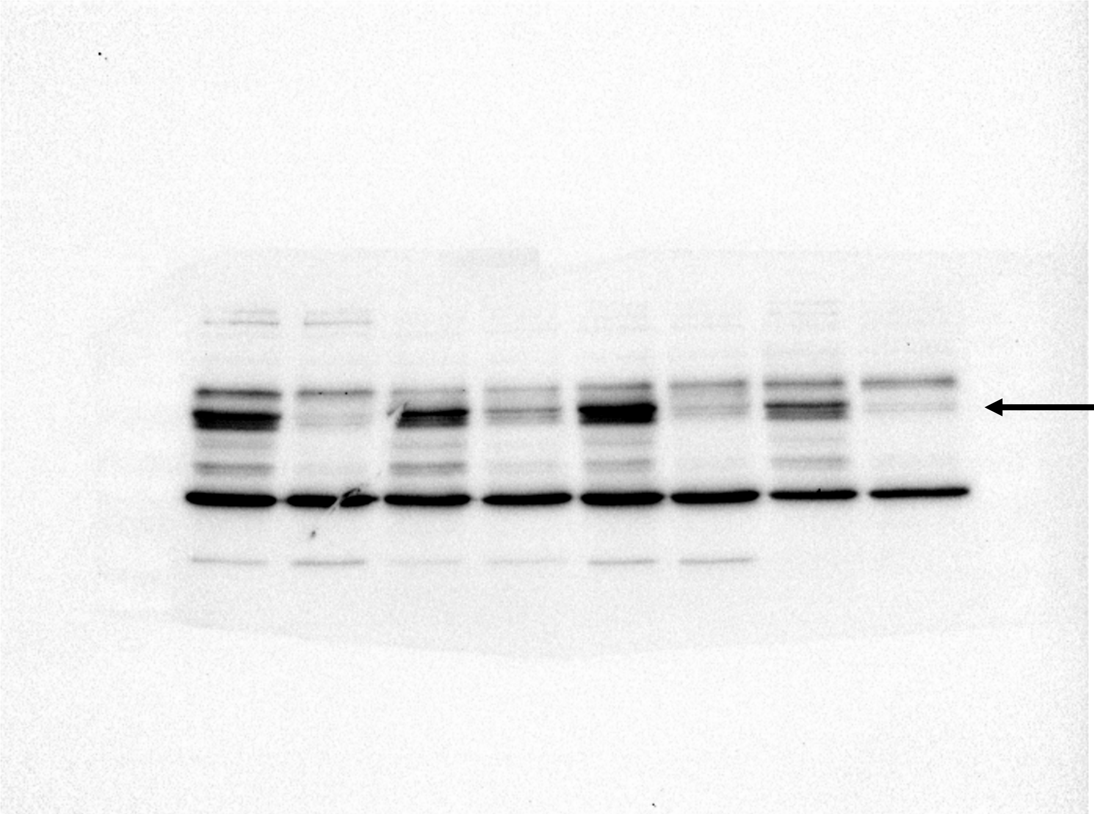

MITF
65 kDa
(Cell Signaling, 12590s, 1:1000)

Figure 1A

Full unedited gel for Figure 1A

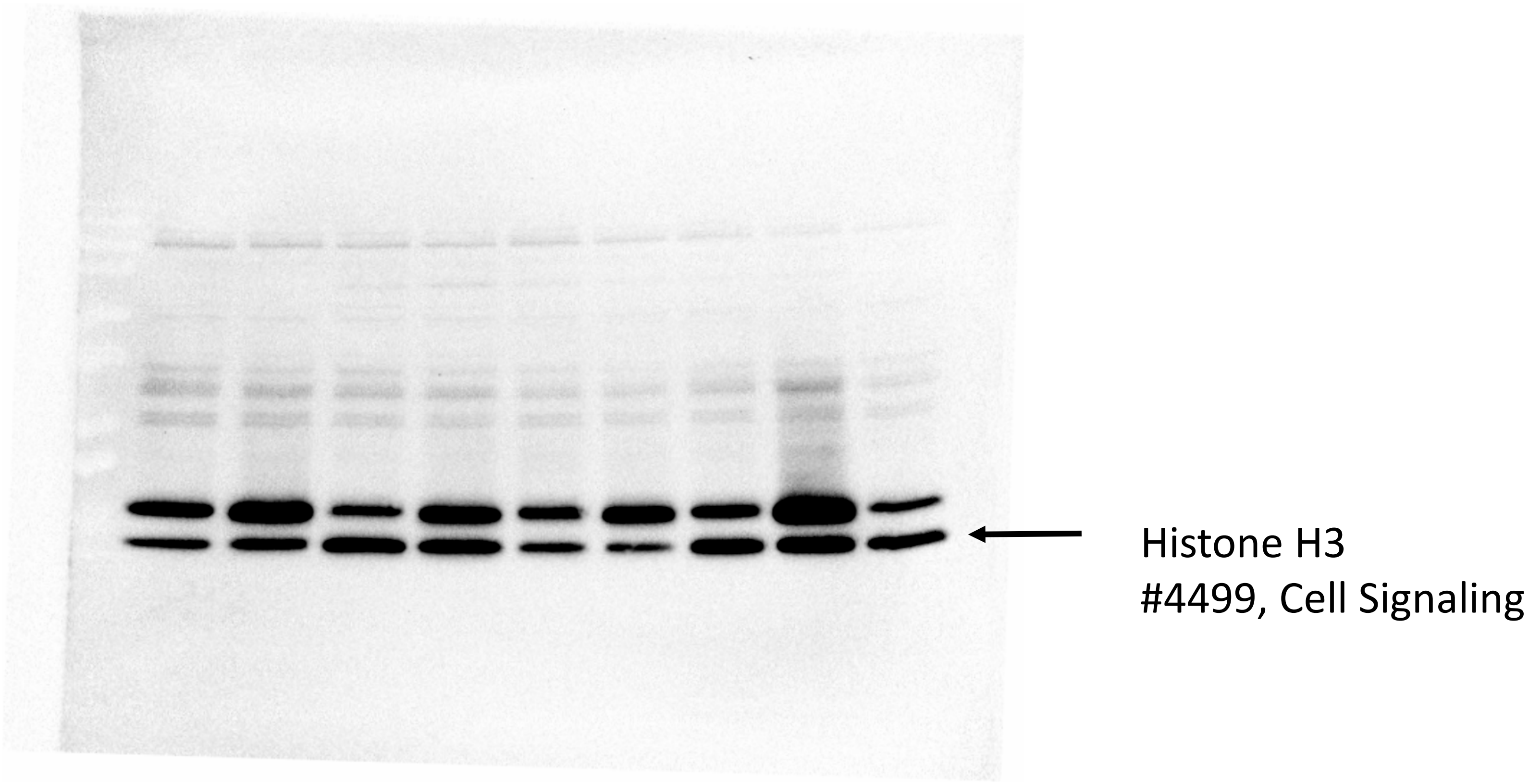

Figure 1A

Full unedited gel for Figure 1A

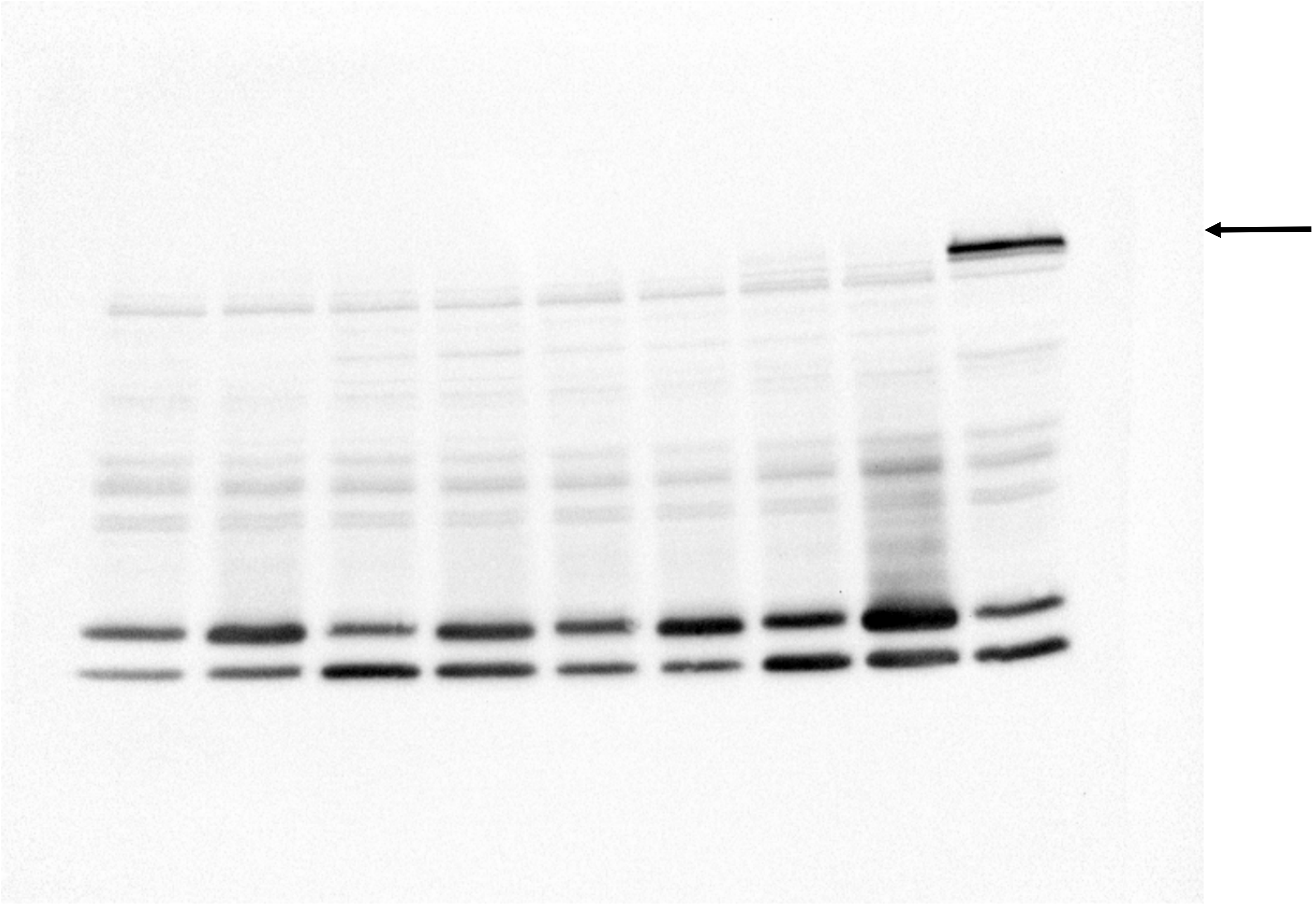

AXL
138 kDa
(Cell Signaling, 8661s, 1:1000)

AXL was run in an additional cell line (1205Lu) cells as a positive control. This shows that the absence of AXL bands in the other cell lines is due to the biology and not the quality of the antibody.

Figure 1A

Full unedited gel for Figure 1A

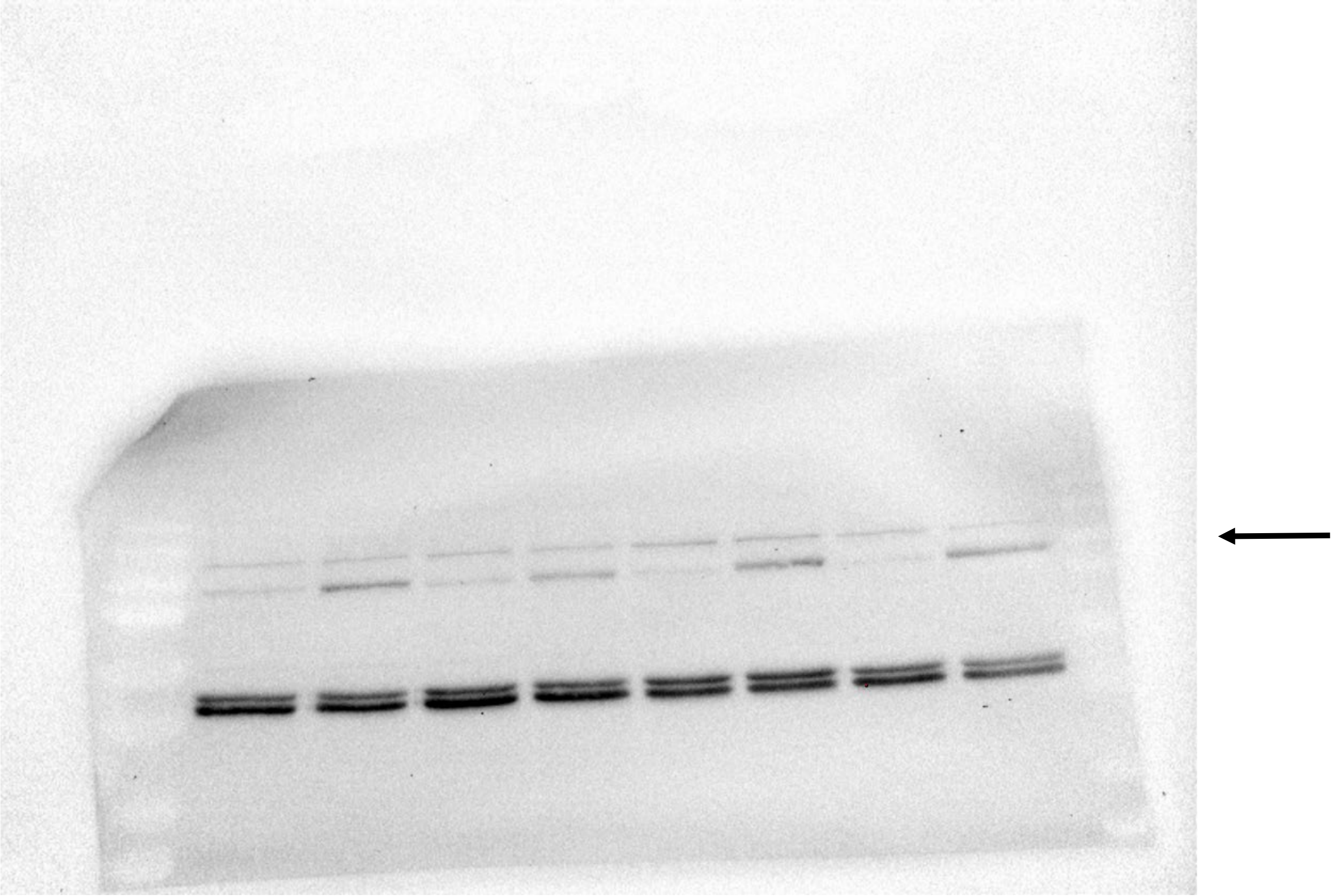

Cleaved PARP
89 kDa
(Cell Signaling, 9541s, 1:1000)

Figure 1A

Full unedited gel for Figure 1A

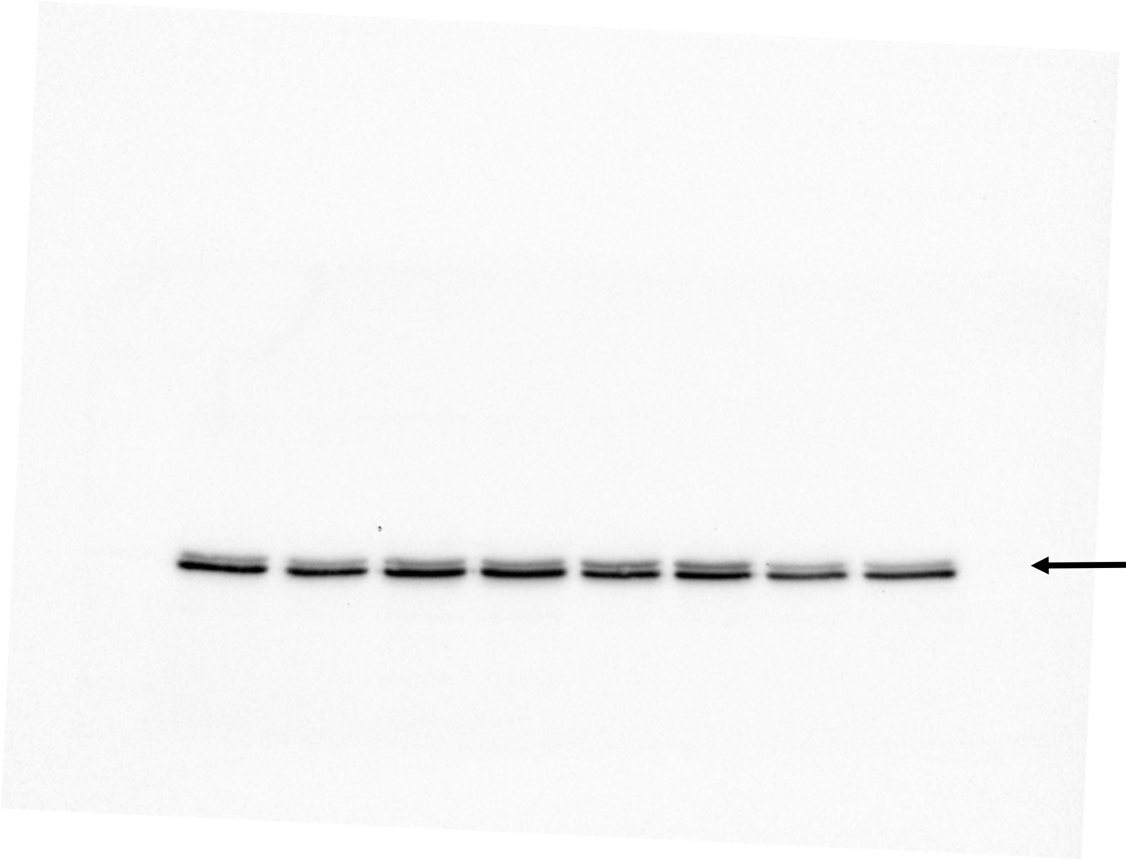

p-ERK
42,44 kDa
(Cell Signaling, 9101s, 1:1000)

Figure 1A

Full unedited gel for Figure 1A

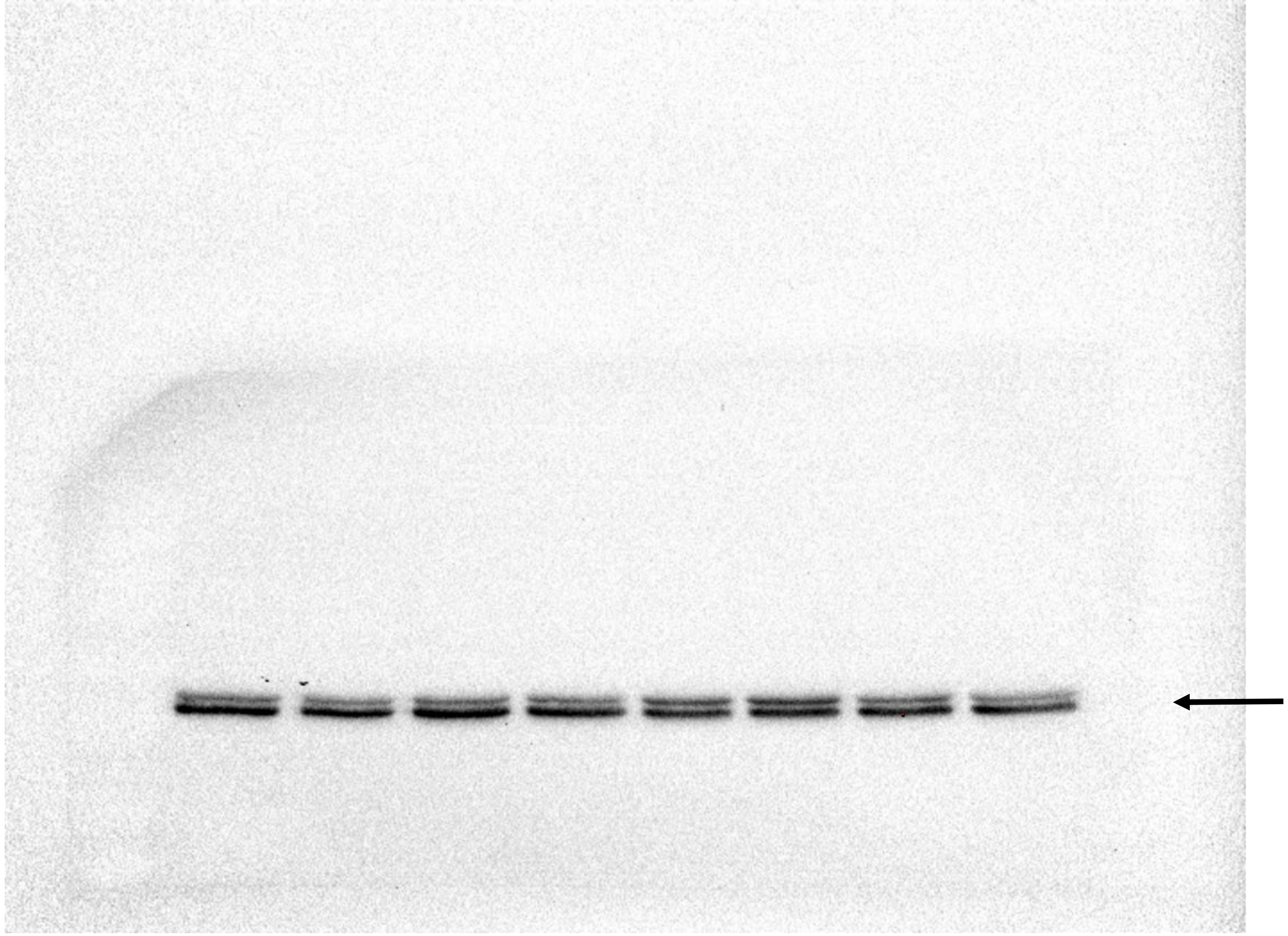

ERK – p44/42 MAPK
42,44 kDa
(Cell Signaling, 9102s, 1:1000)

Figure 1A

Full unedited gel for Figure 1A

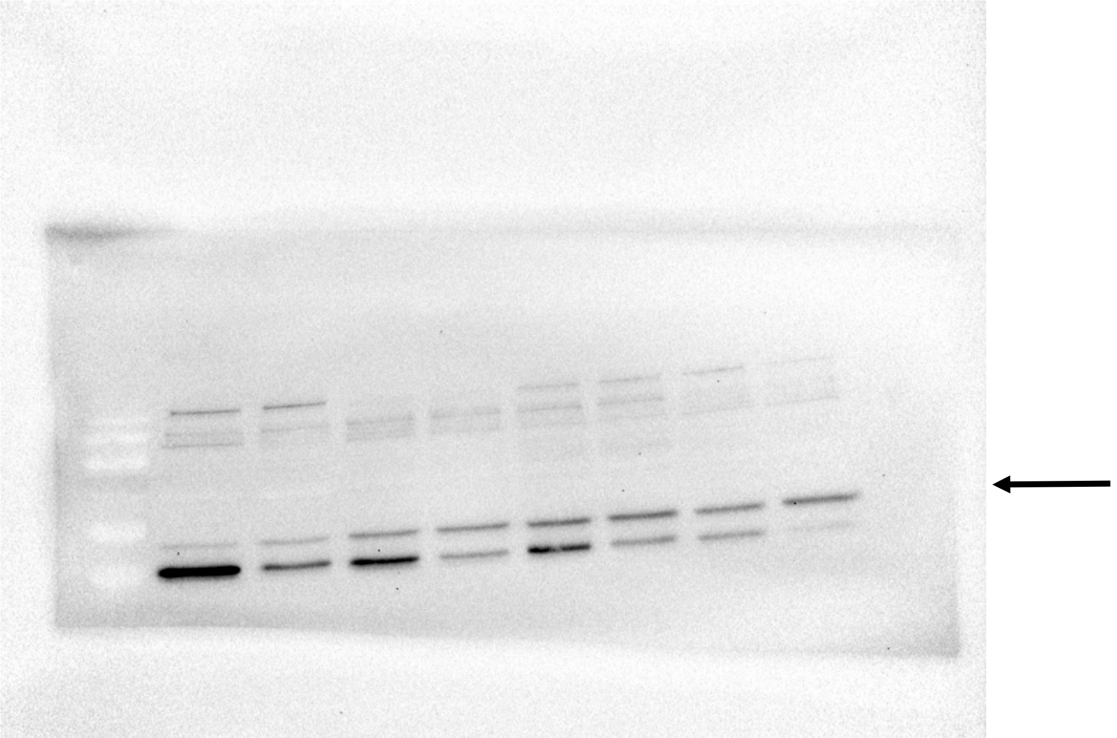

pMEK1/2
45 kDa
(Cell Signaling, 9121, 1:1000)

Figure 1A

Full unedited gel for Figure 1A

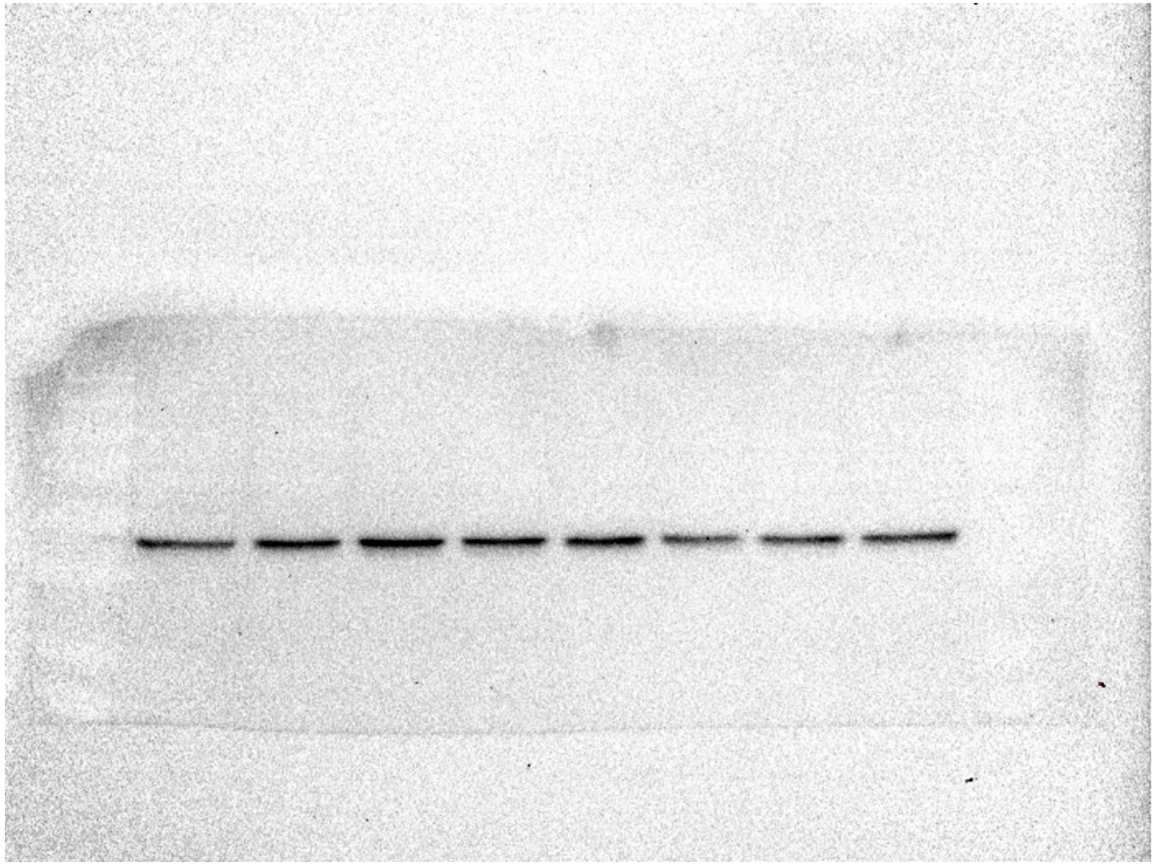

MEK
45 kDa
(Cell Signaling, 9122, 1:1000)

Figure 1A

Full unedited gel for Figure 1A

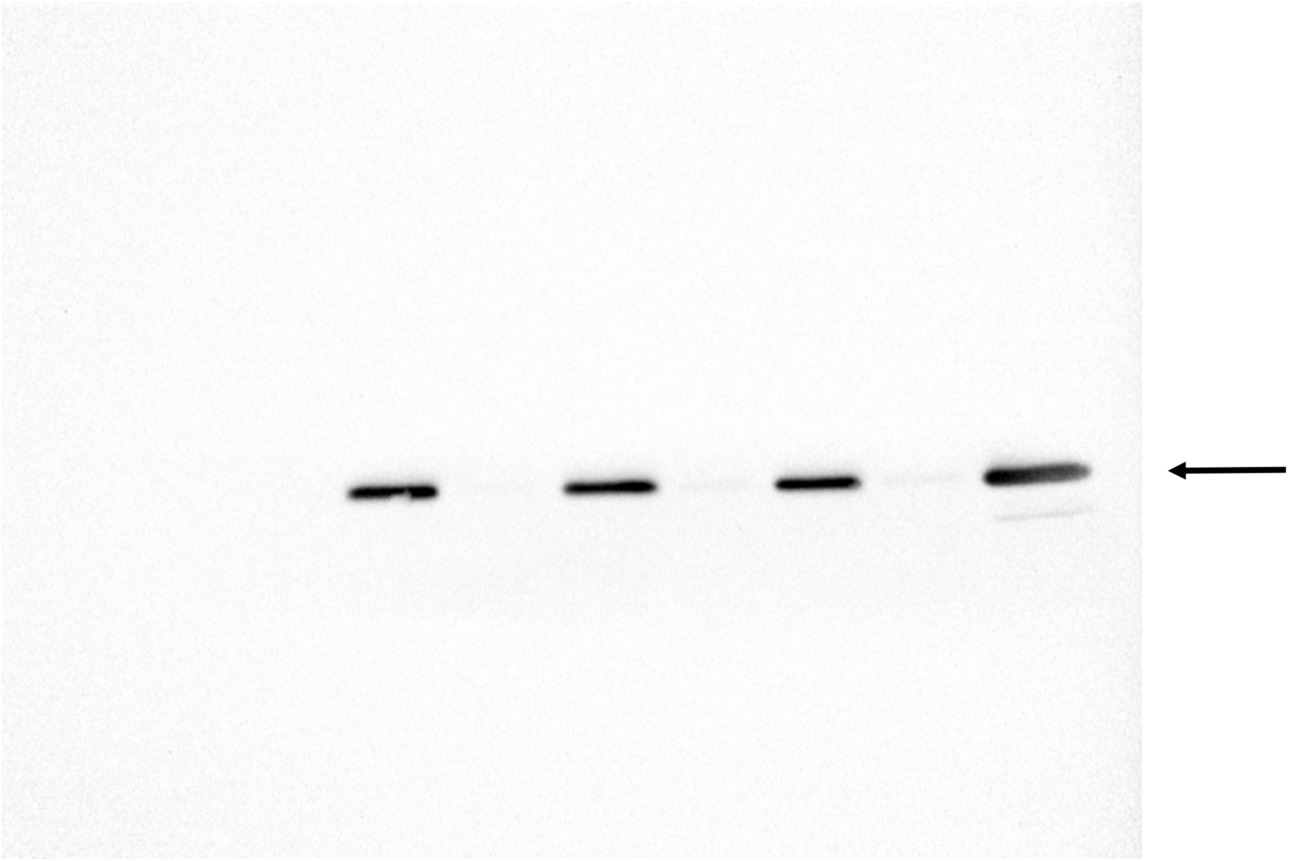

H3K27ac
15 kDa
(Abcam, ab4729, 1:1000)

Figure 1A

Full unedited gel for Figure 1A

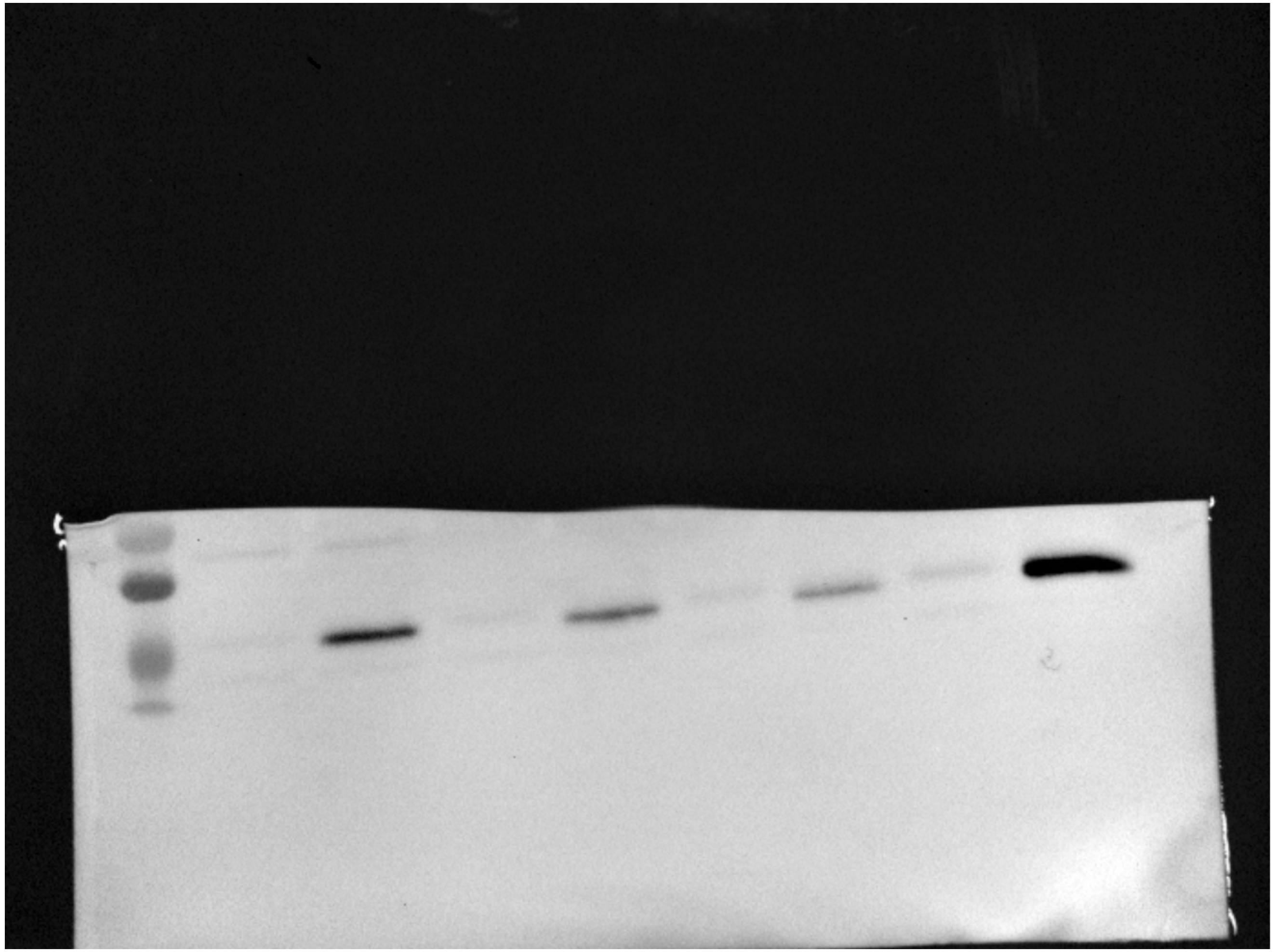

H3K4me2
18 kDa
(Abcam, ab32356, 1:5000)

Figure 1A

Full unedited gel for Figure 1A

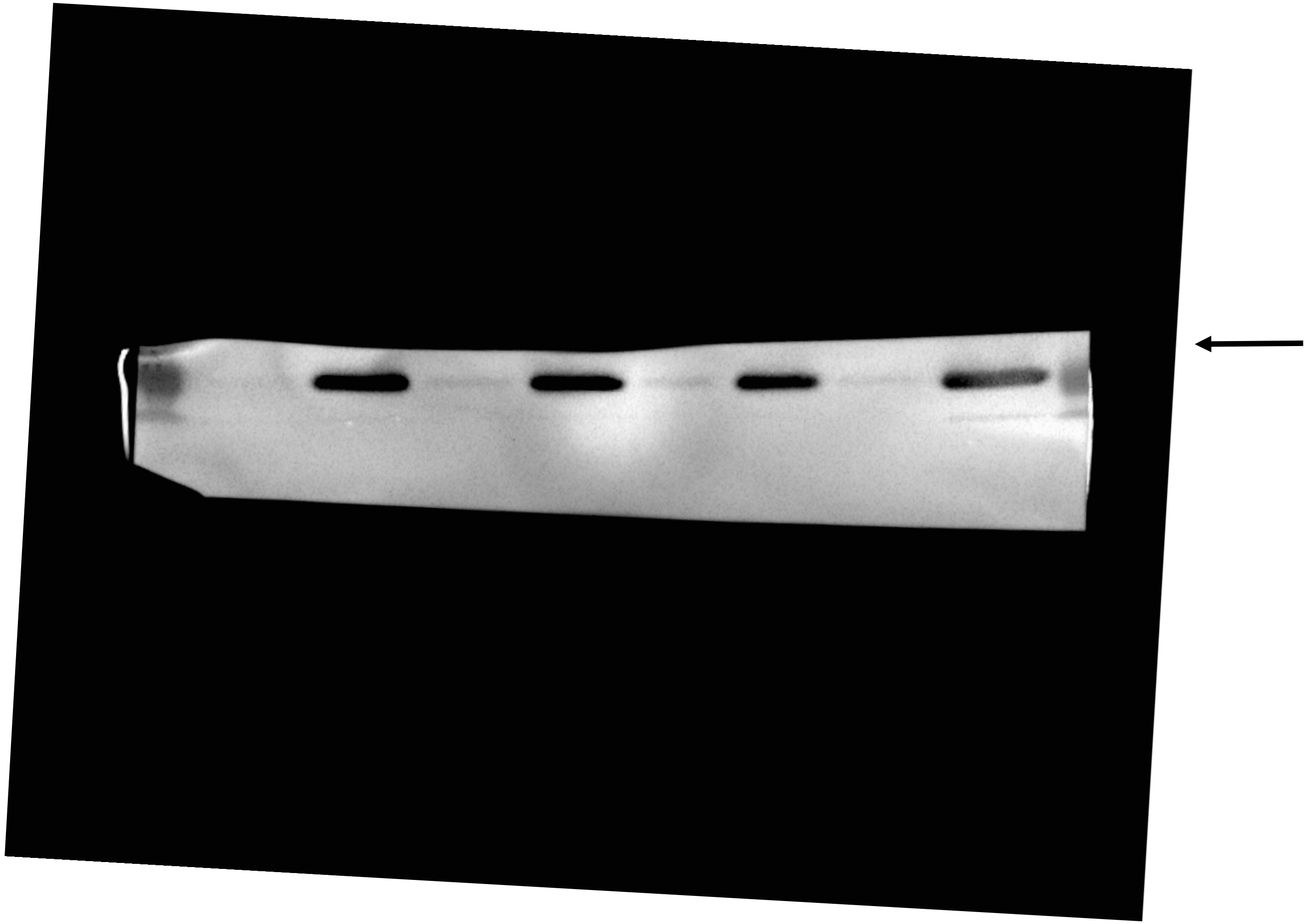

H3K9ac
18 kDa
(Abcam, ab32129, 1:1000)

Figure 1A

Full unedited gel for Figure 1A

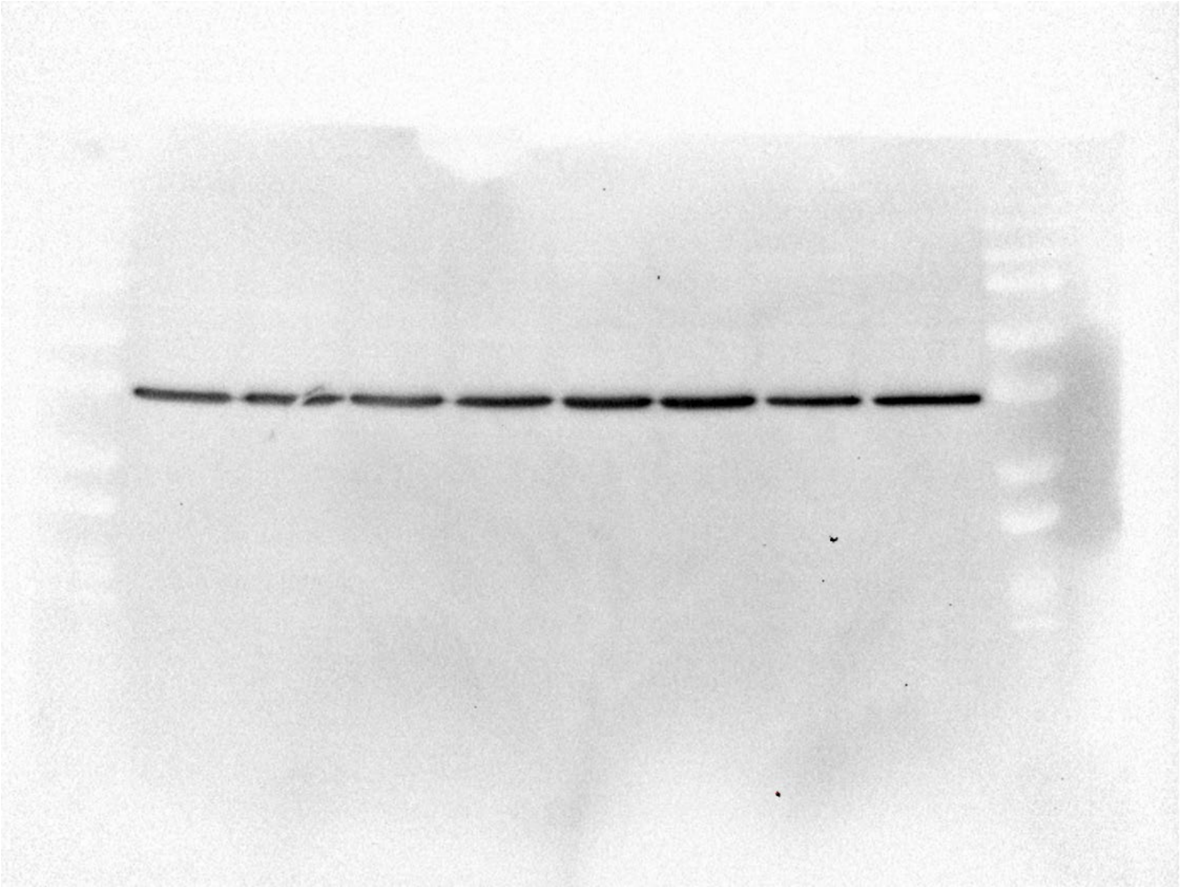

GAPDH
36 kDa
(Cell Signaling, 2118L, 1:2000)

Figure 1A

Full unedited gel for Figure 1B

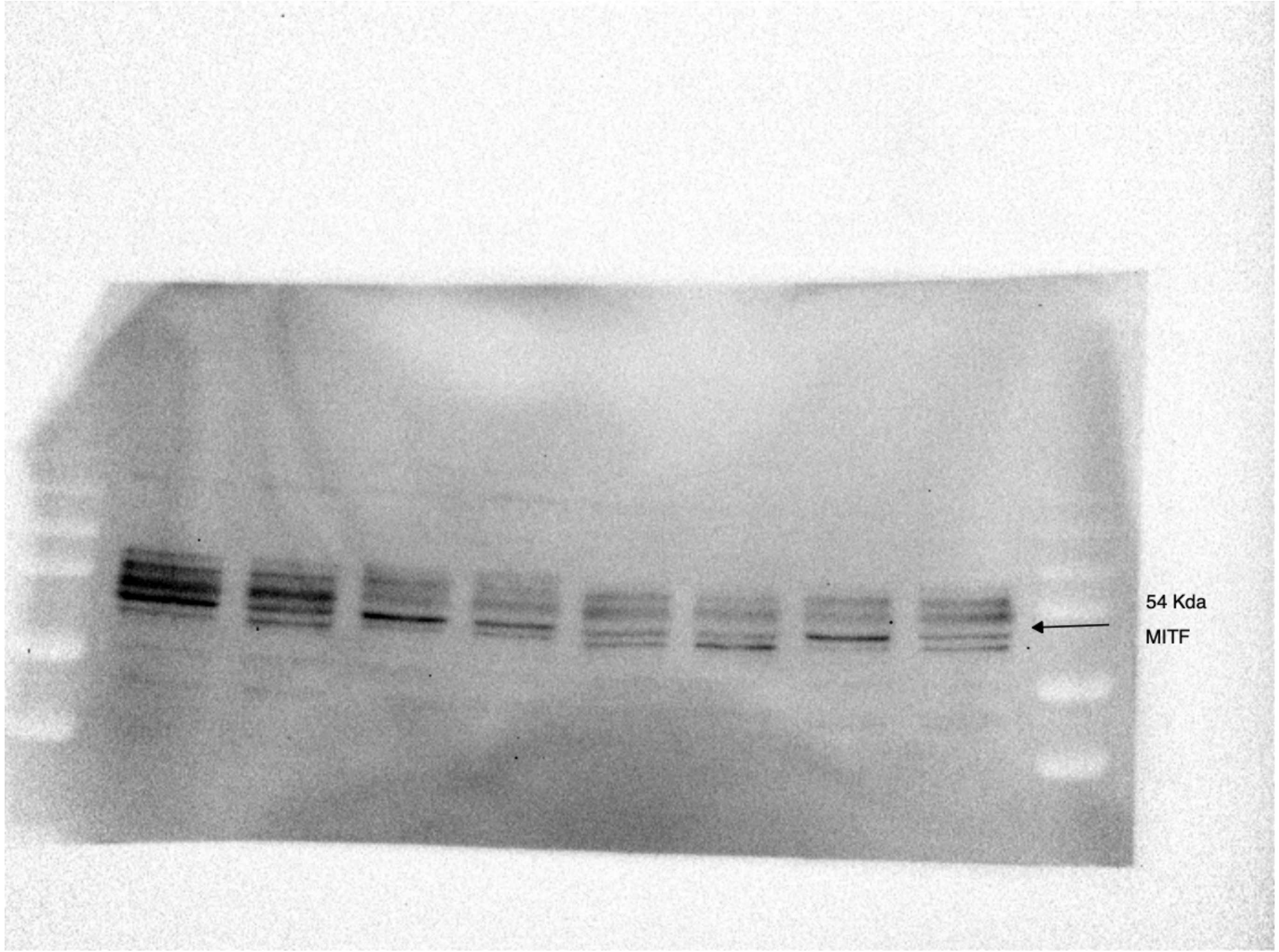

Figure 1B

(Cell Signaling, 12590s, 1:1000)

Full unedited gel for Figure 1B

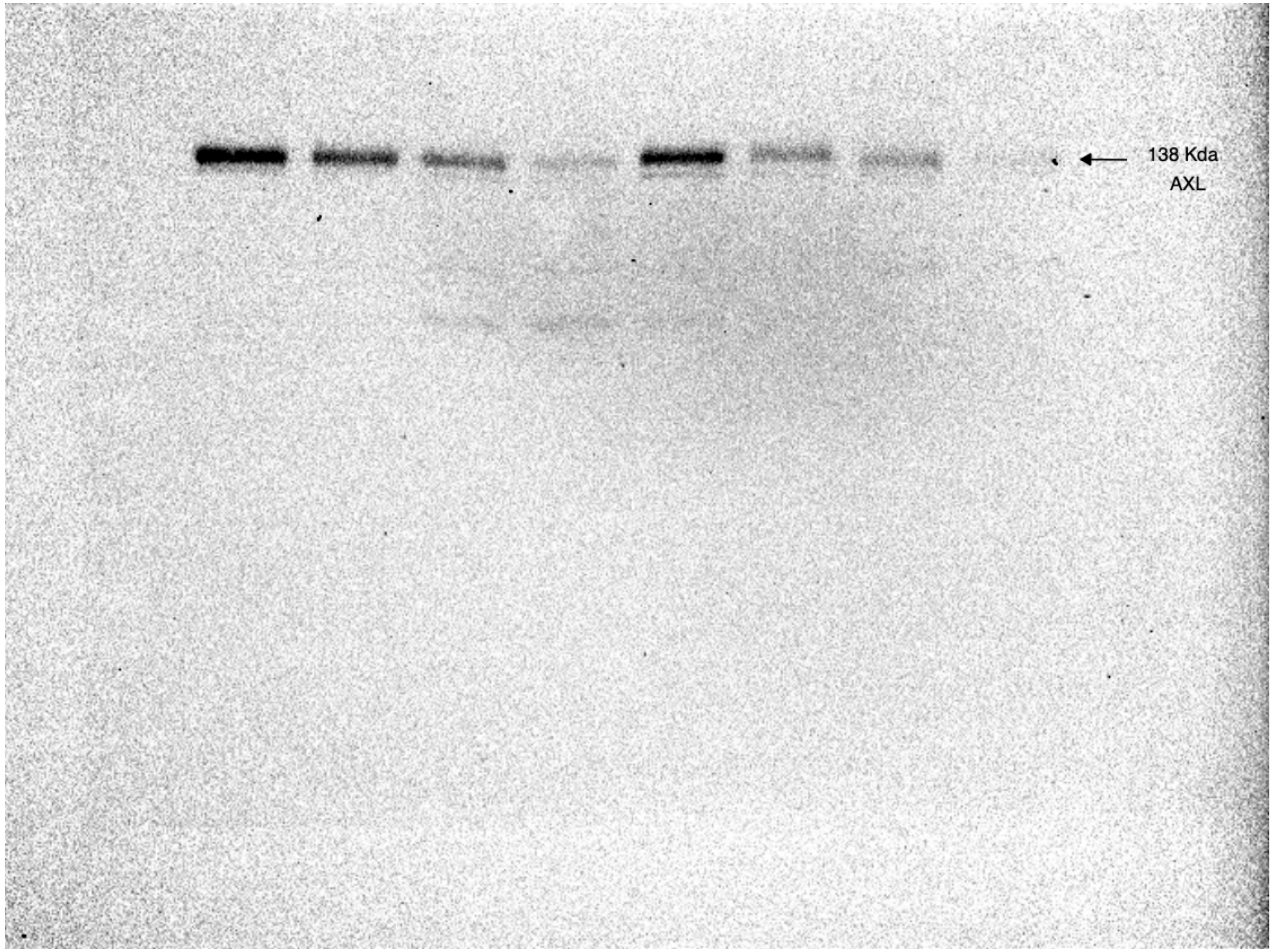

Figure 1B

(Cell Signaling, 8661s, 1:1000)

Full unedited gel for Figure 1B

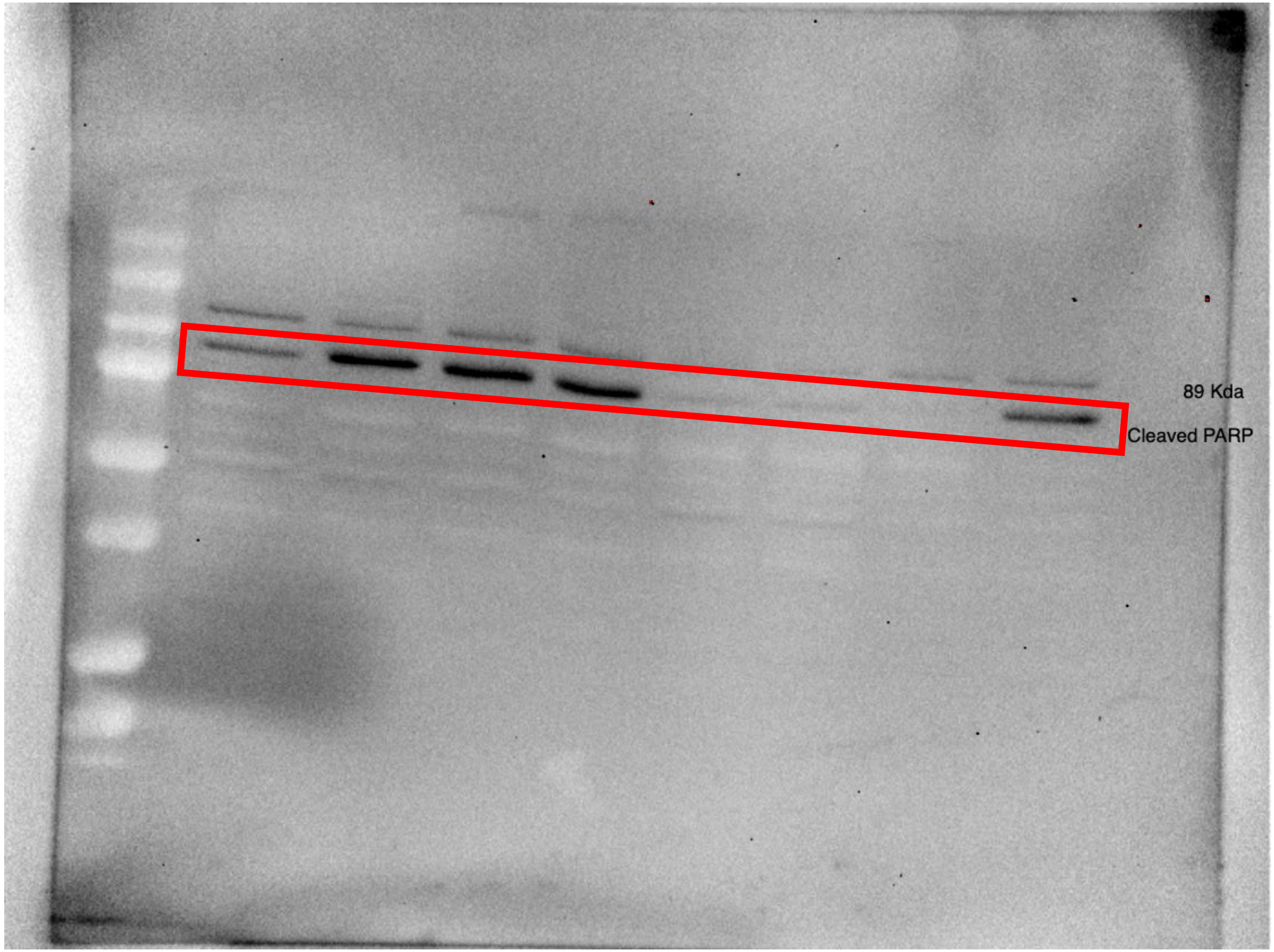

(Cell Signaling, 9541s, 1:1000)

Figure 1B

Full unedited gel for Figure 1B

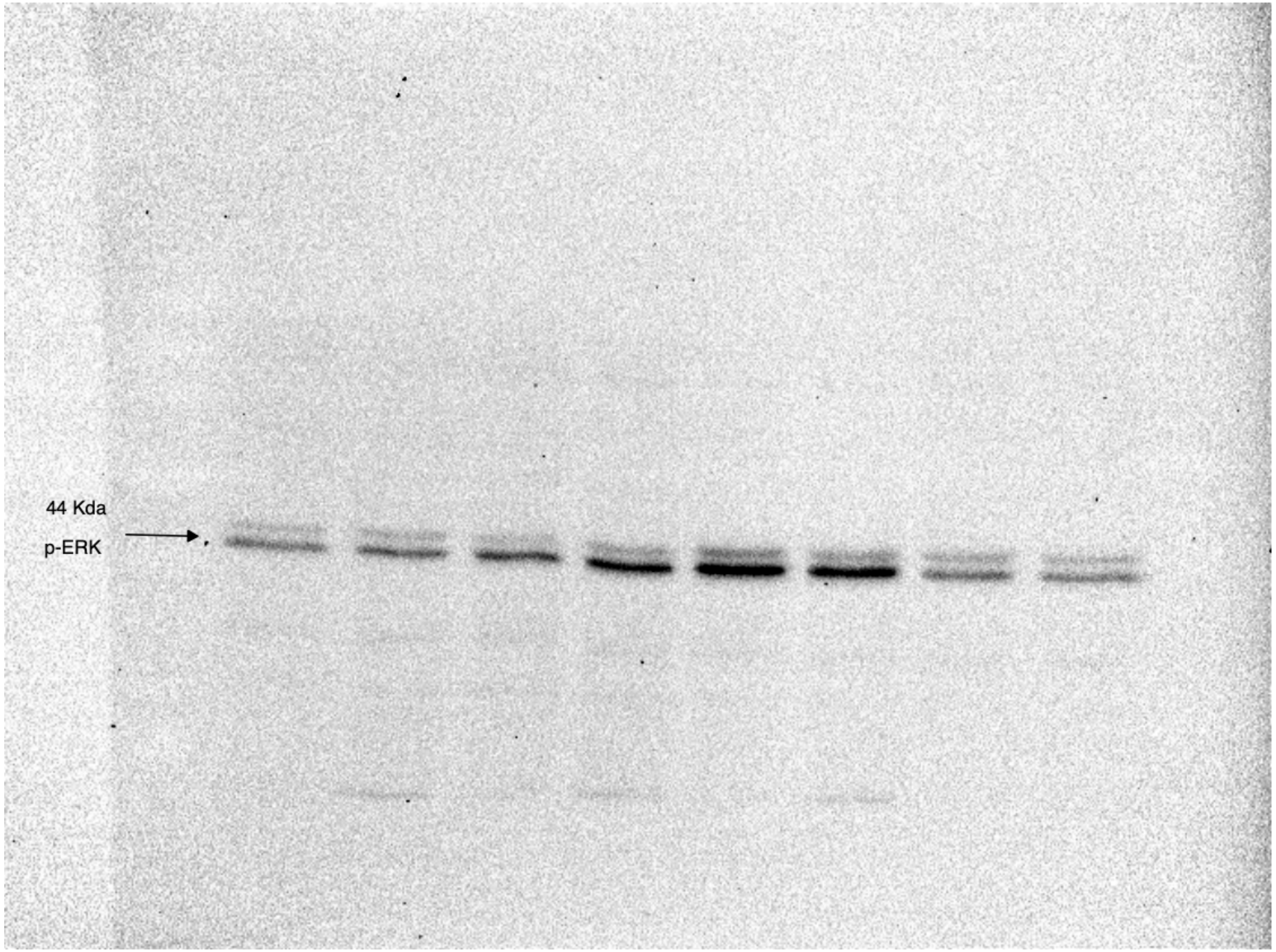

Figure 1B

(Cell Signaling, 9101s, 1:1000)

Full unedited gel for Figure 1B

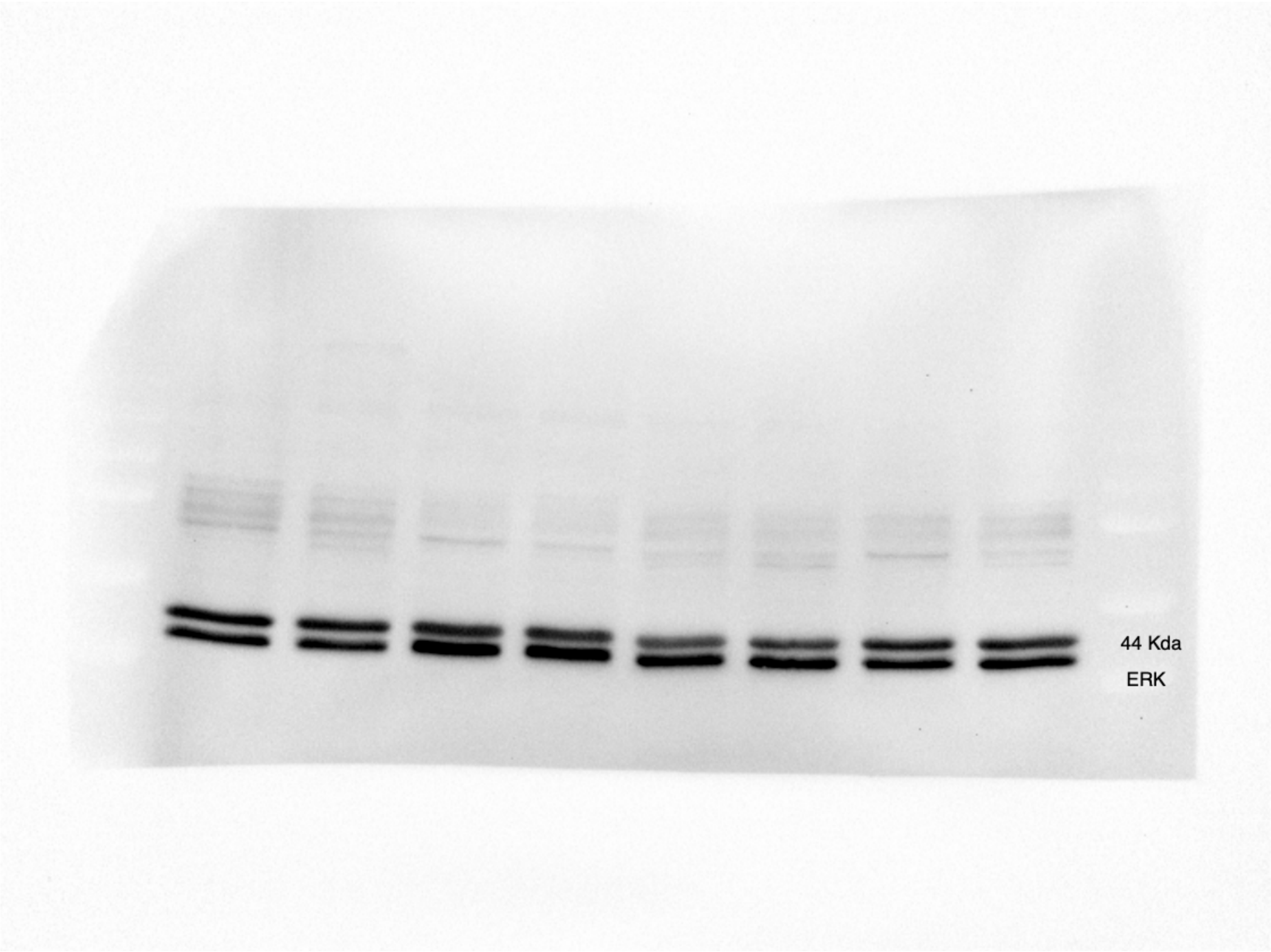

(Cell Signaling, 9102s, 1:1000)

Figure 1B

Full unedited gel for Figure 1B

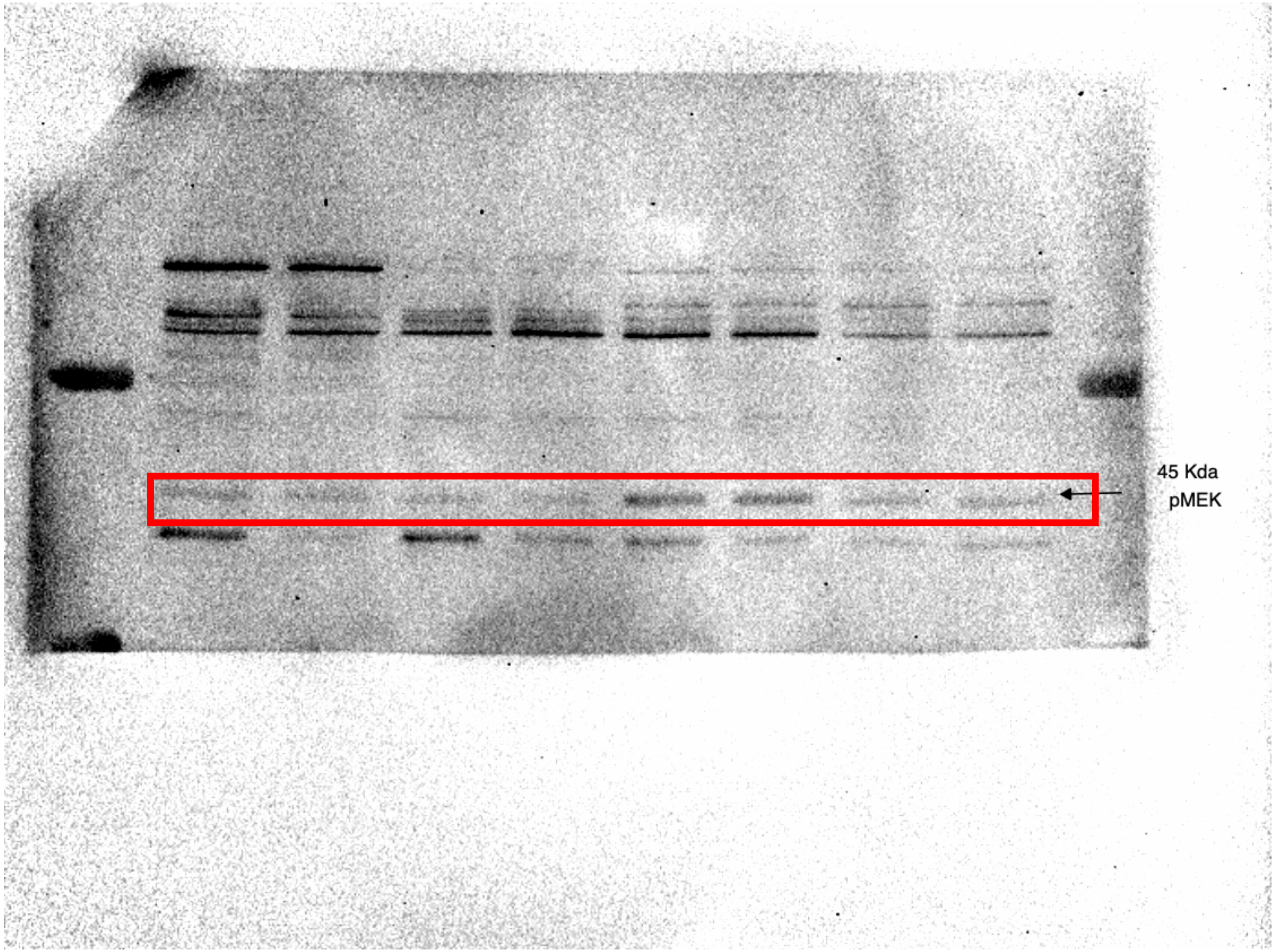

(Cell Signaling, 9121, 1:1000)

Figure 1B

Full unedited gel for Figure 1B

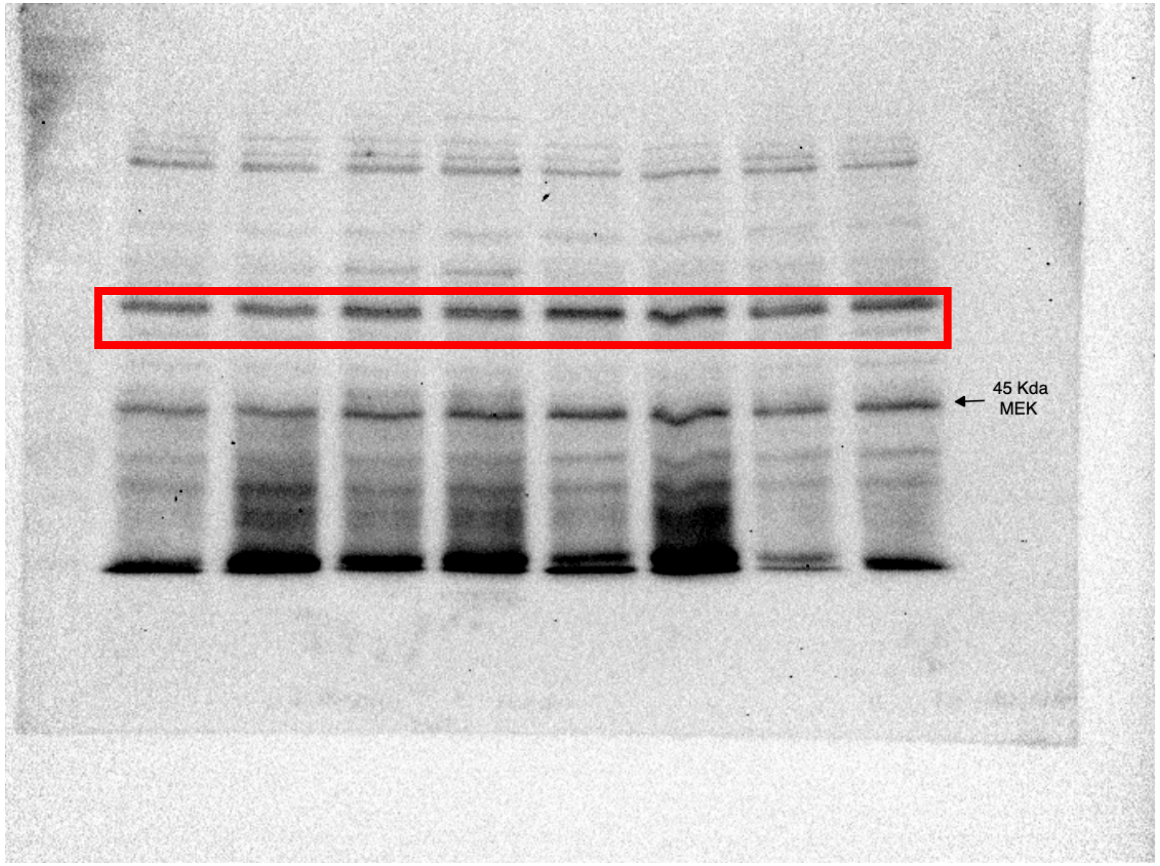

(Cell Signaling, 9122, 1:1000)

Figure 1B

Full unedited gel for Figure 1B

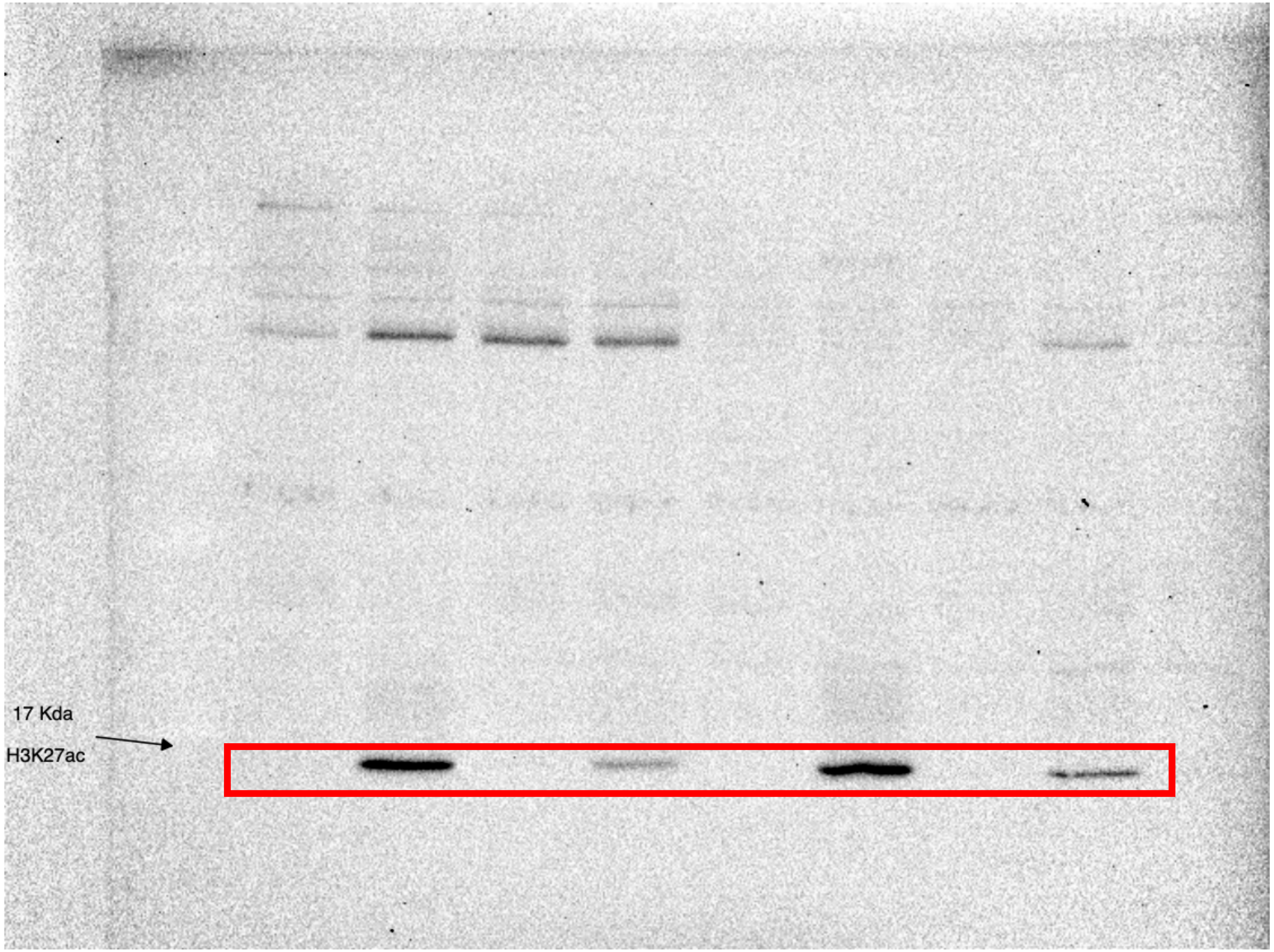

Main Figure 1B

(Abcam, ab4729, 1:1000)

Full unedited gel for Figure 1B

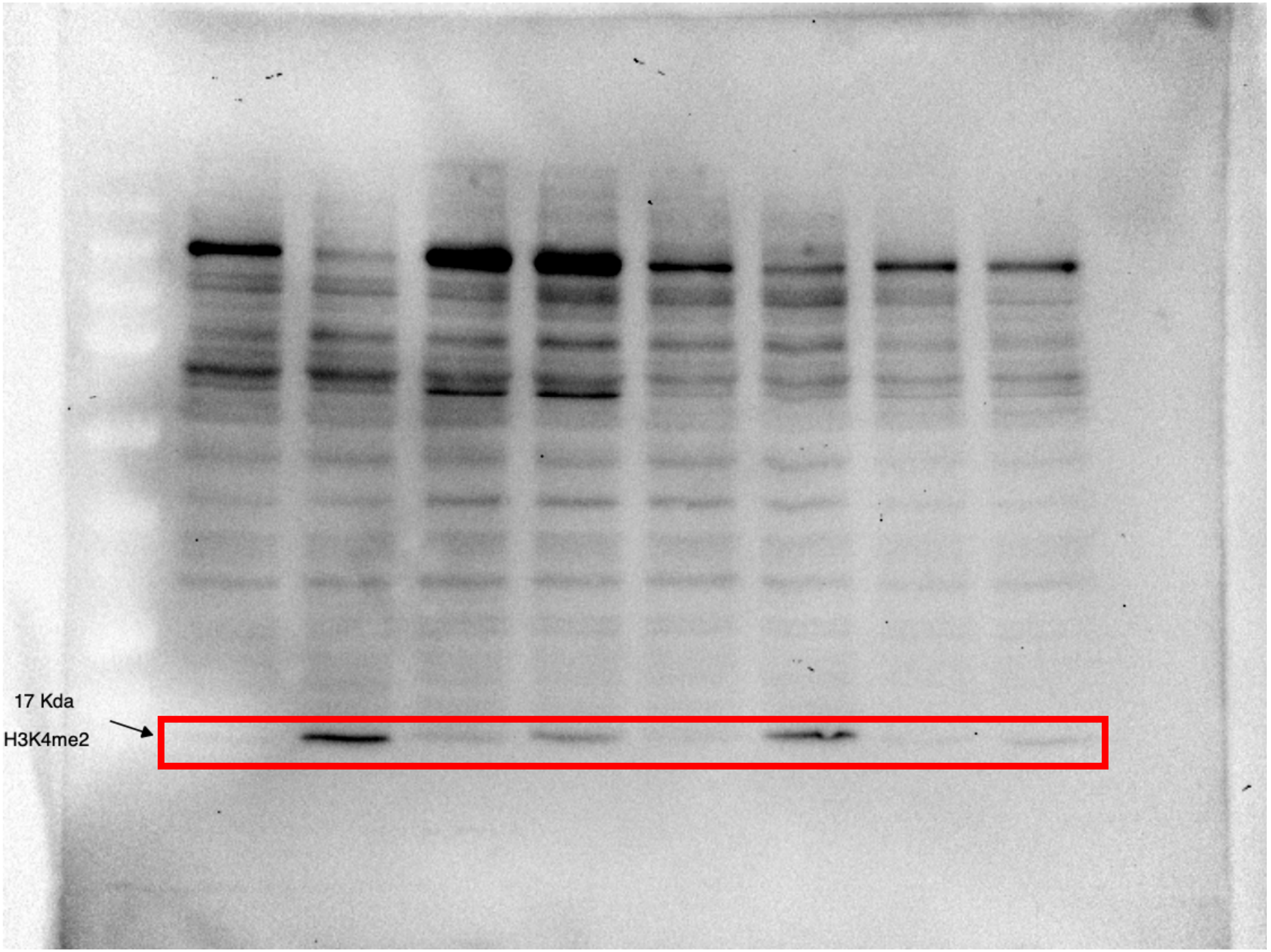

(Abcam, ab32356, 1:5000)

Figure 1B

Full unedited gel for Figure 1B

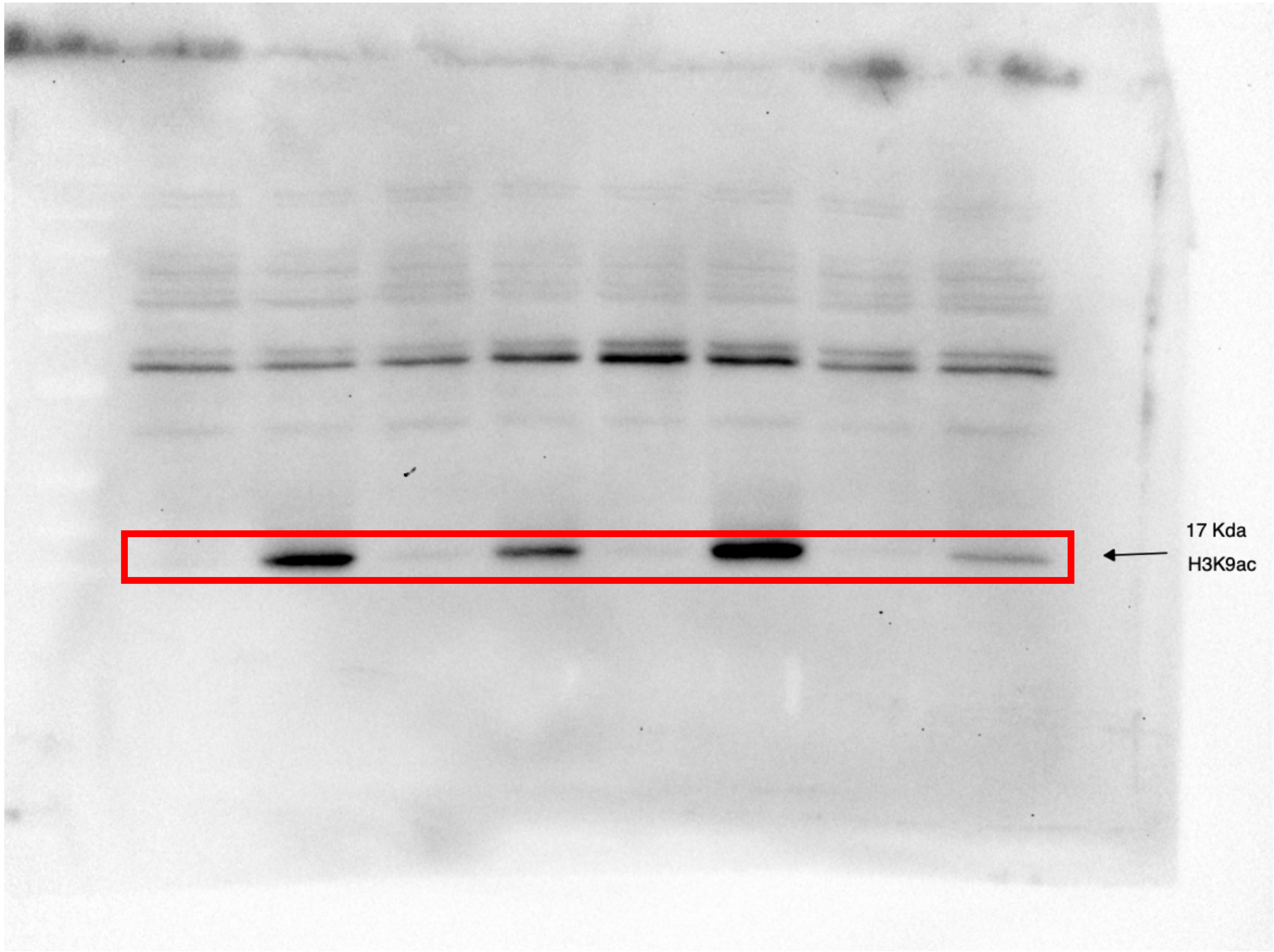

(Abcam, ab32129, 1:1000)

Figure 1B

Full unedited gel for Figure 1B

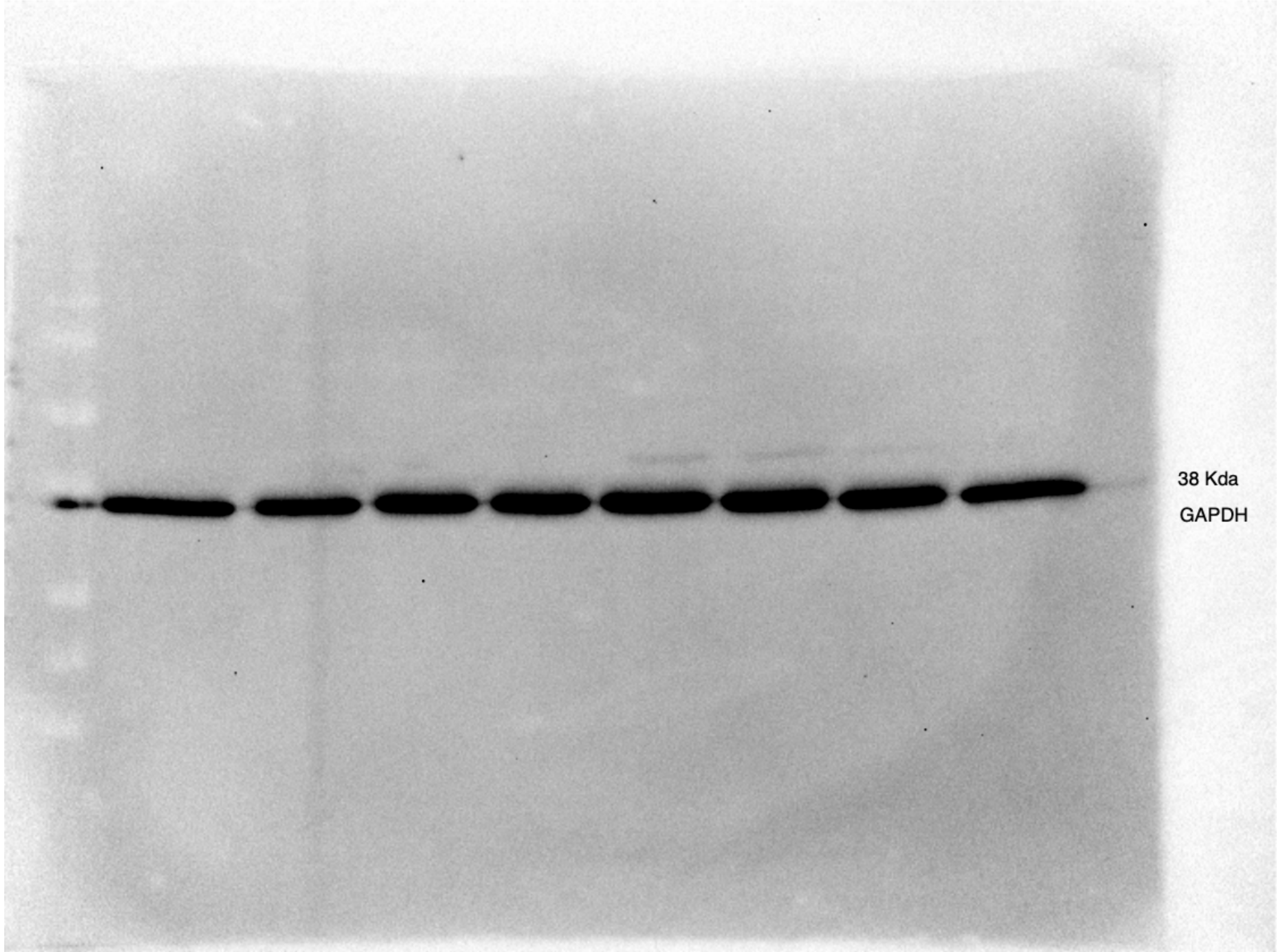

(Cell Signaling, 2118L, 1:2000)

Figure 1B

Full unedited gel for Figure 9A

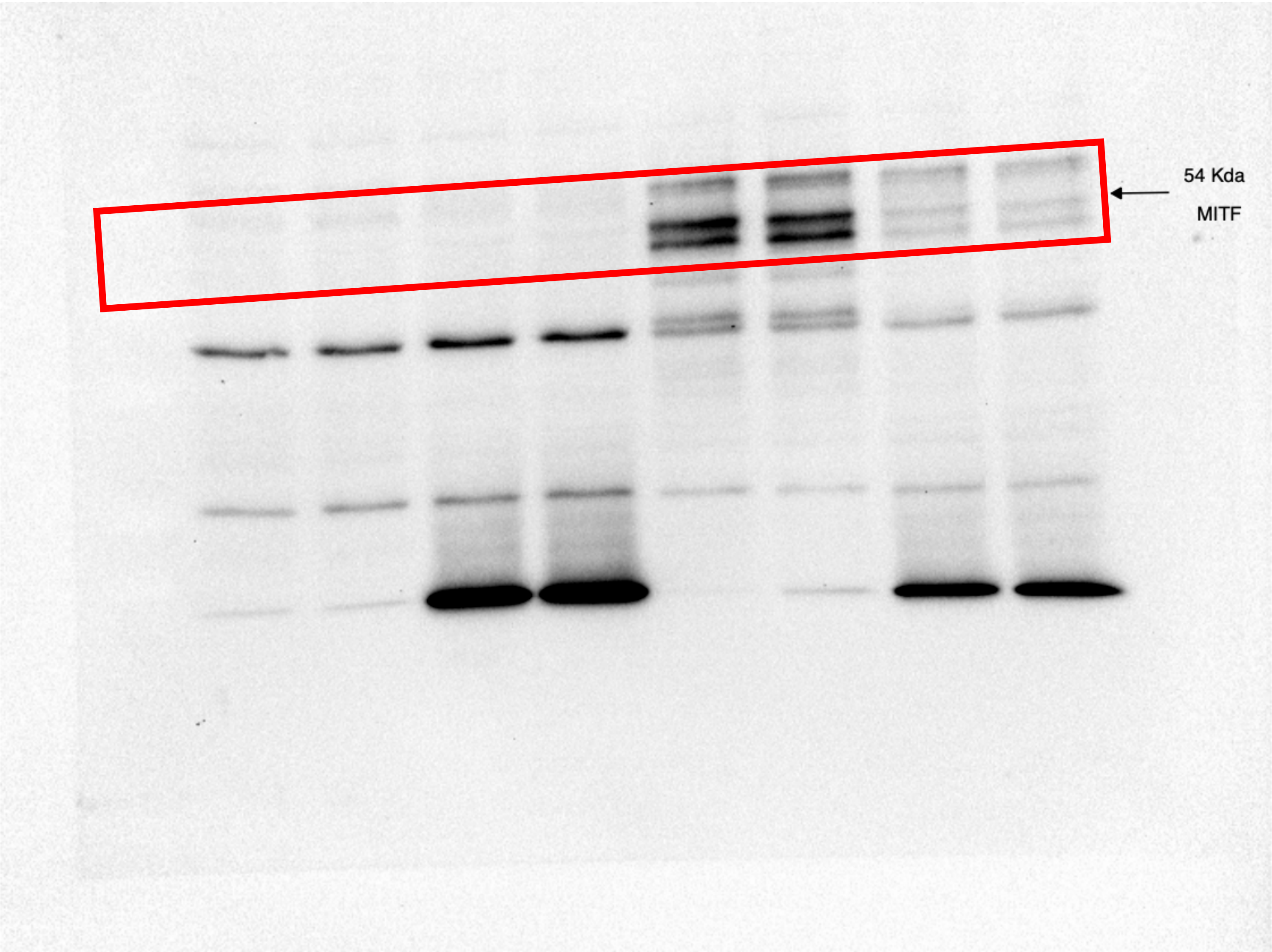

(MITF, Cell Signaling, 12590s, 1:1000)

Figure 9A

Full unedited gel for Figure 9A

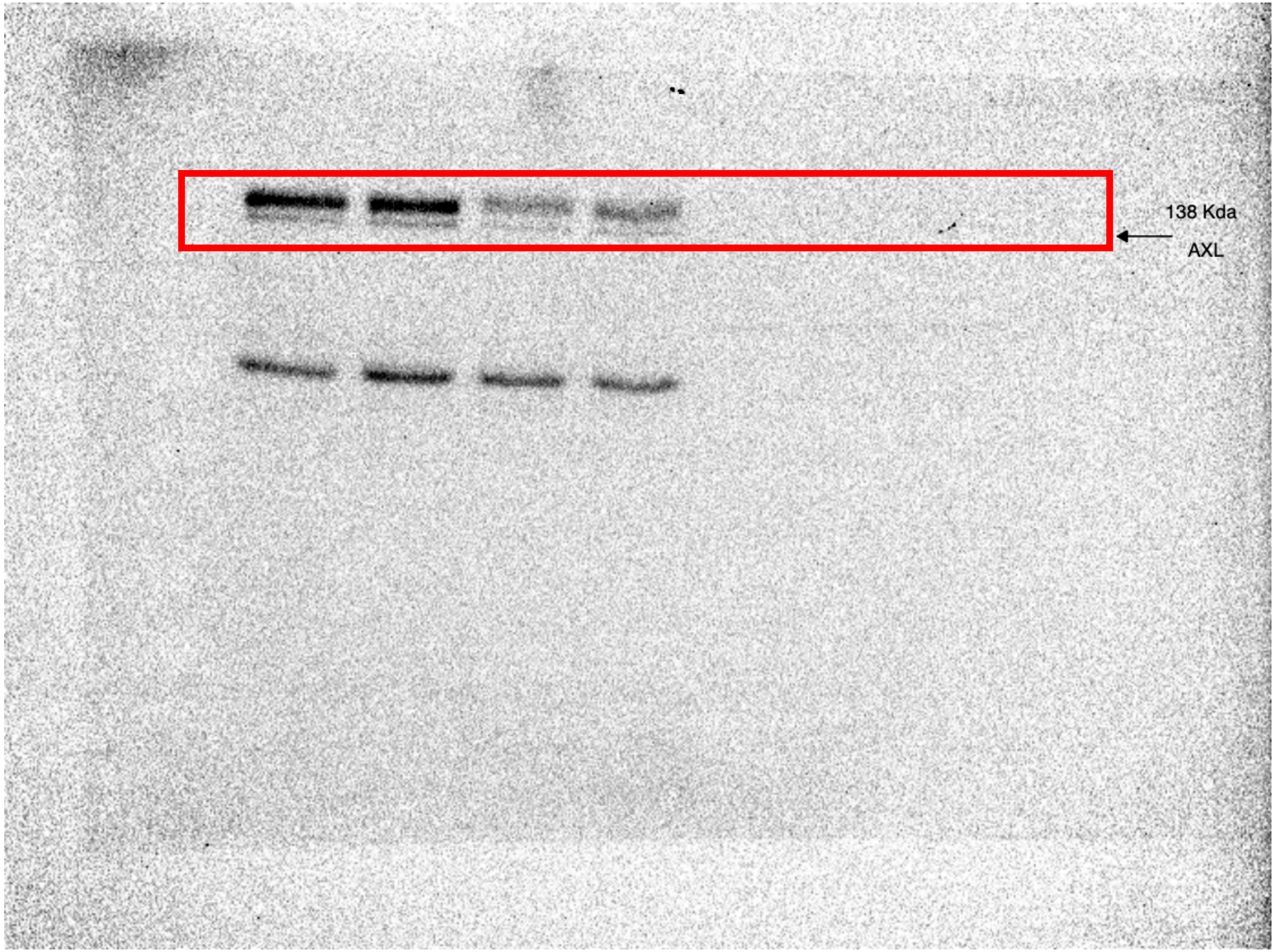

(AXL, Cell Signaling, 8661s, 1:1000)

Figure 9A

Full unedited gel for Figure 9A

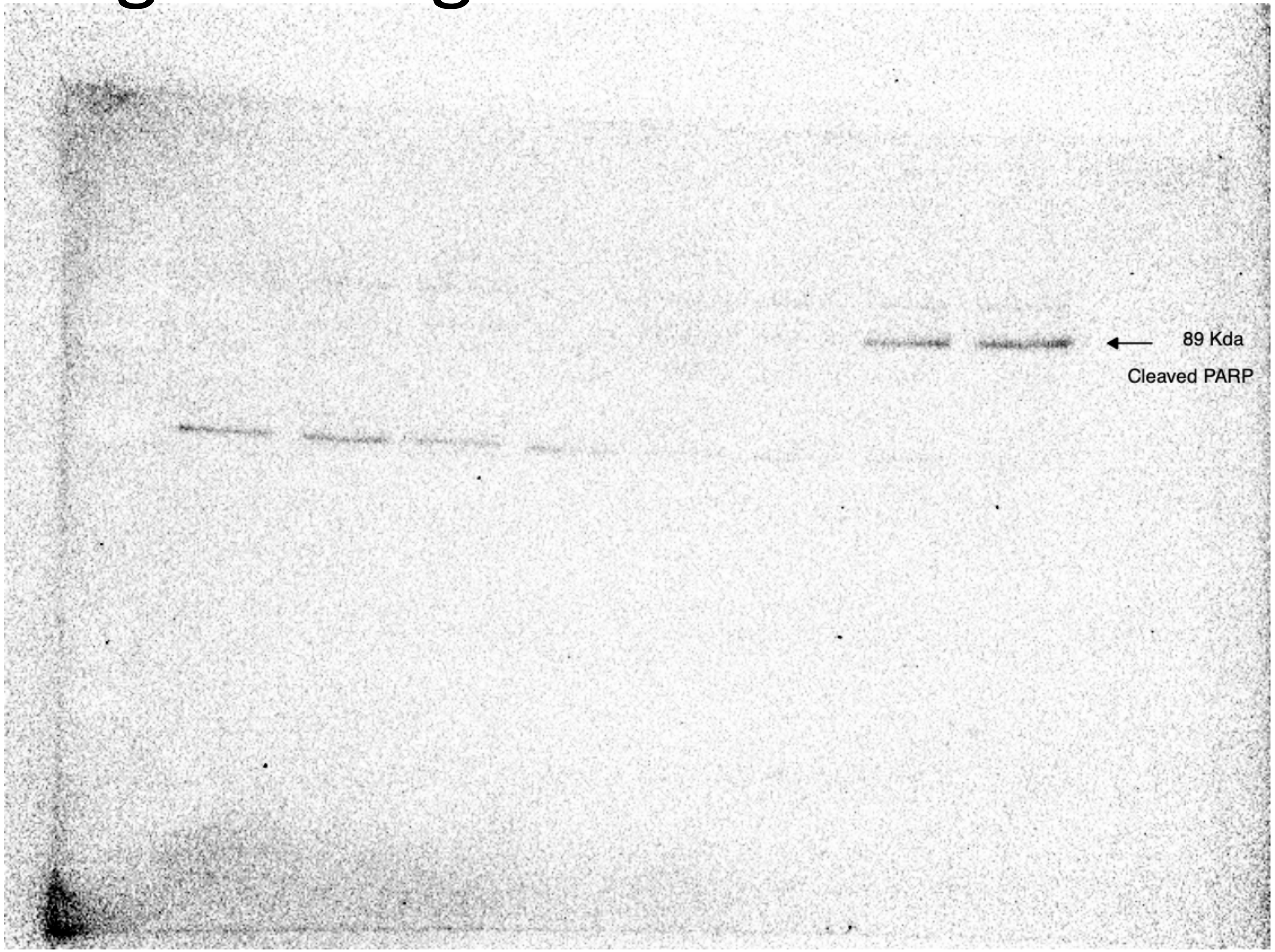

(Cleaved PARP, Cell Signaling, 9541s, 1:1000)

Figure 9A

Full unedited gel for Figure 9A

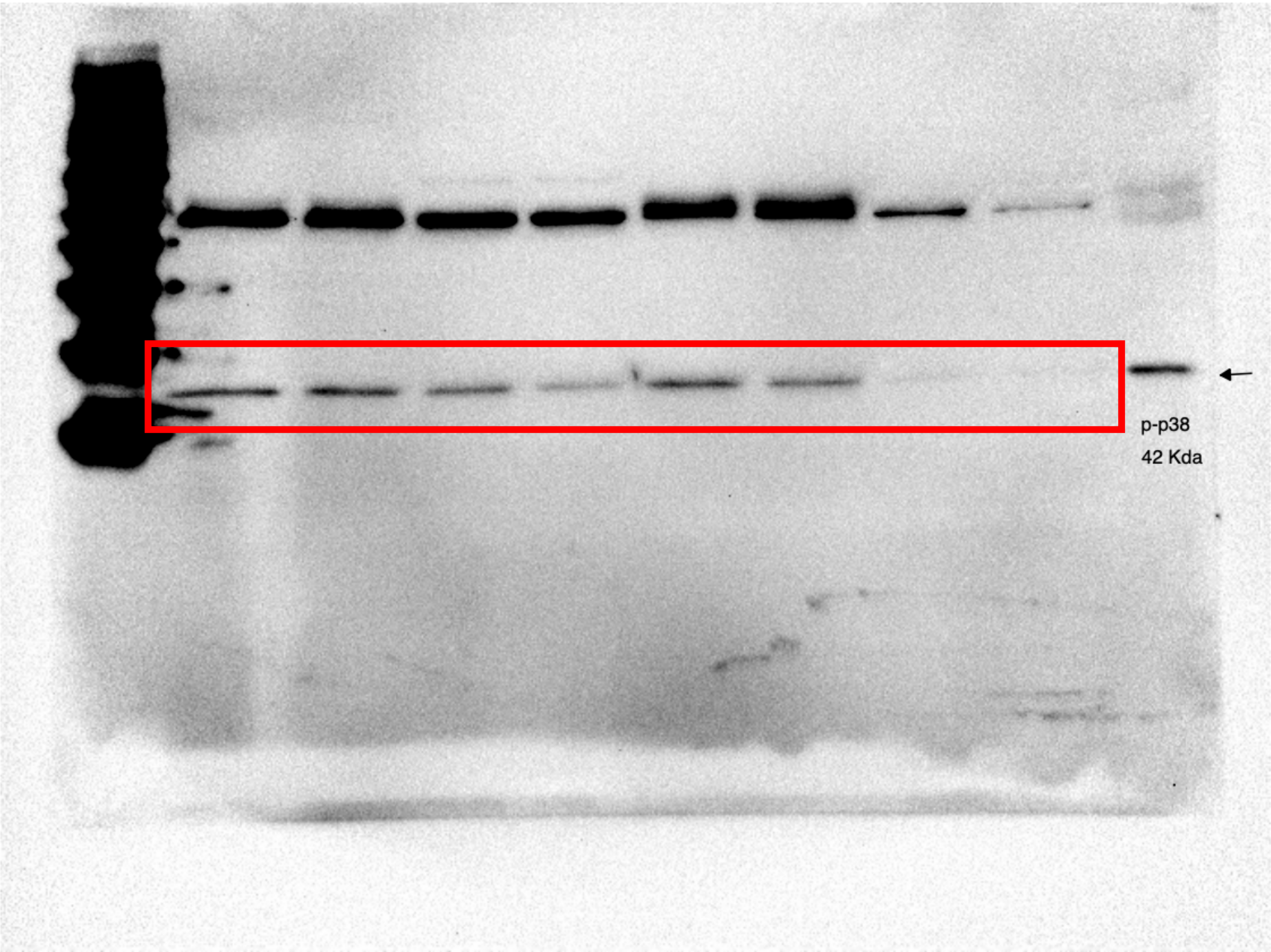

(p-p38, Cell Signaling, 4631s, 1:1000)

Figure 9A

Full unedited gel for Figure 9A

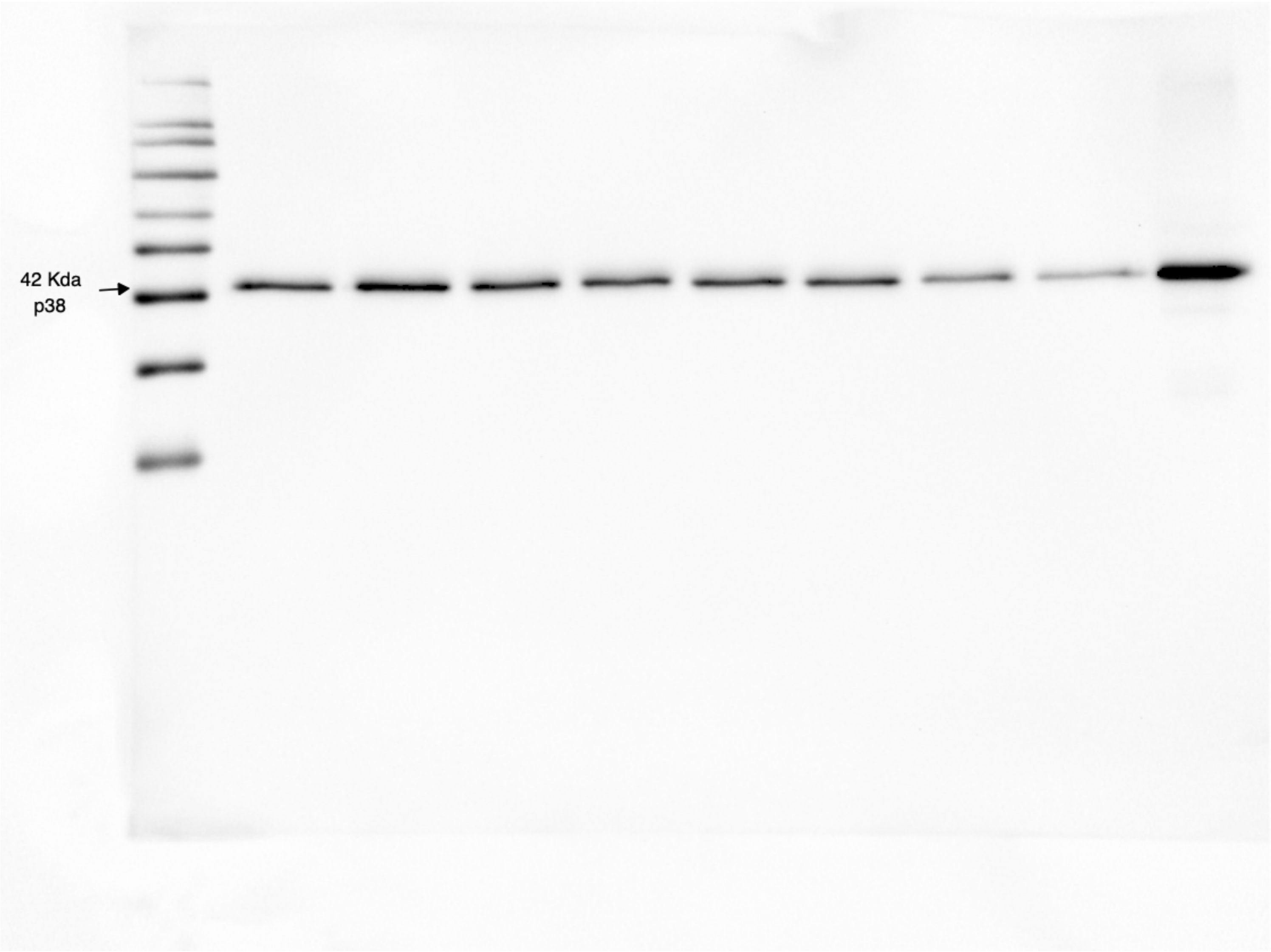

(p38, Cell Signaling, 9212s, 1:1000)

Figure 9A

Full unedited gel for Figure 9A

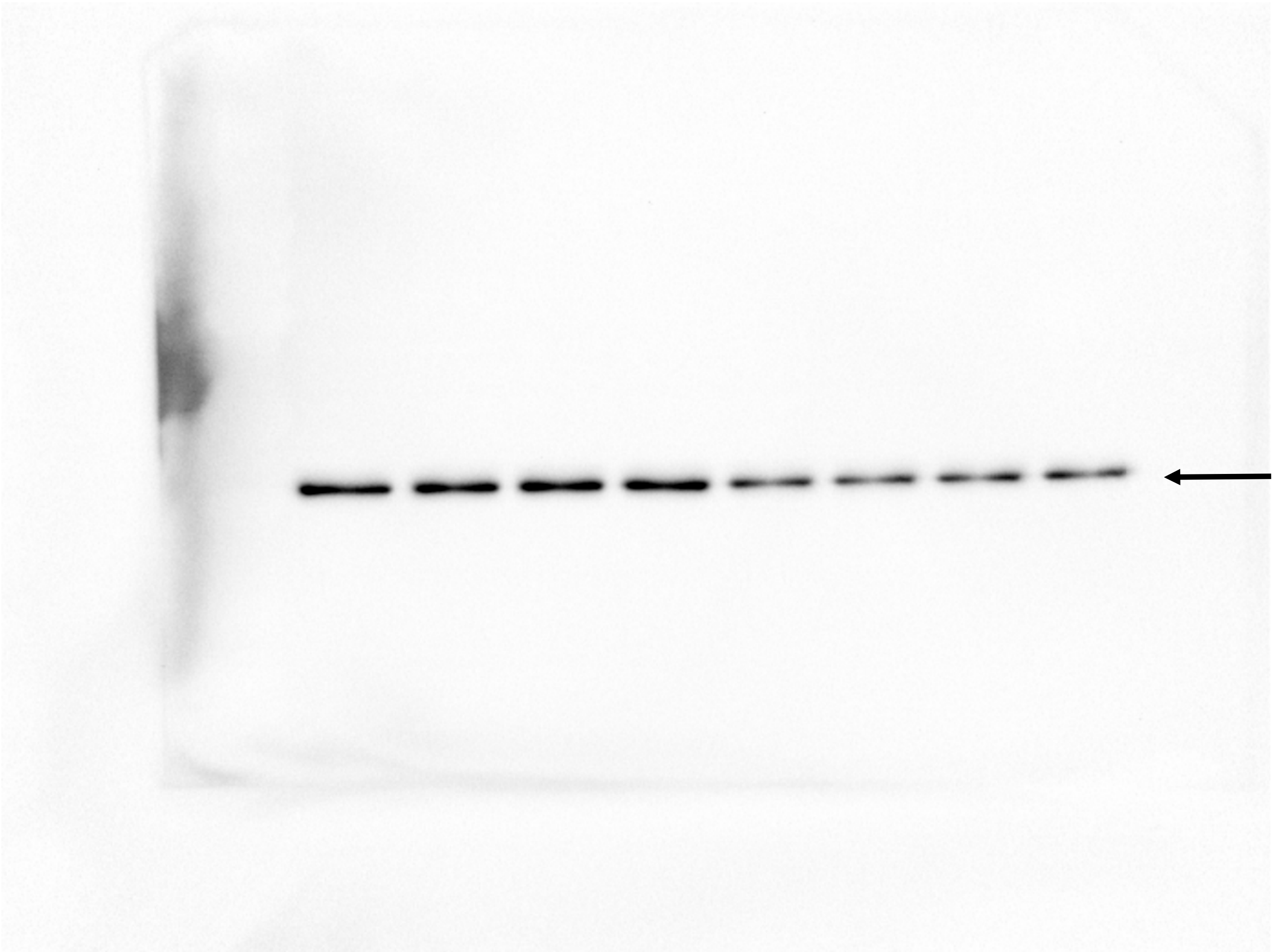

p-c-JUN
40 kDa
(Cell Signaling, 9261s, 1:1000)

Figure 9A

Full unedited gel for Figure 9A

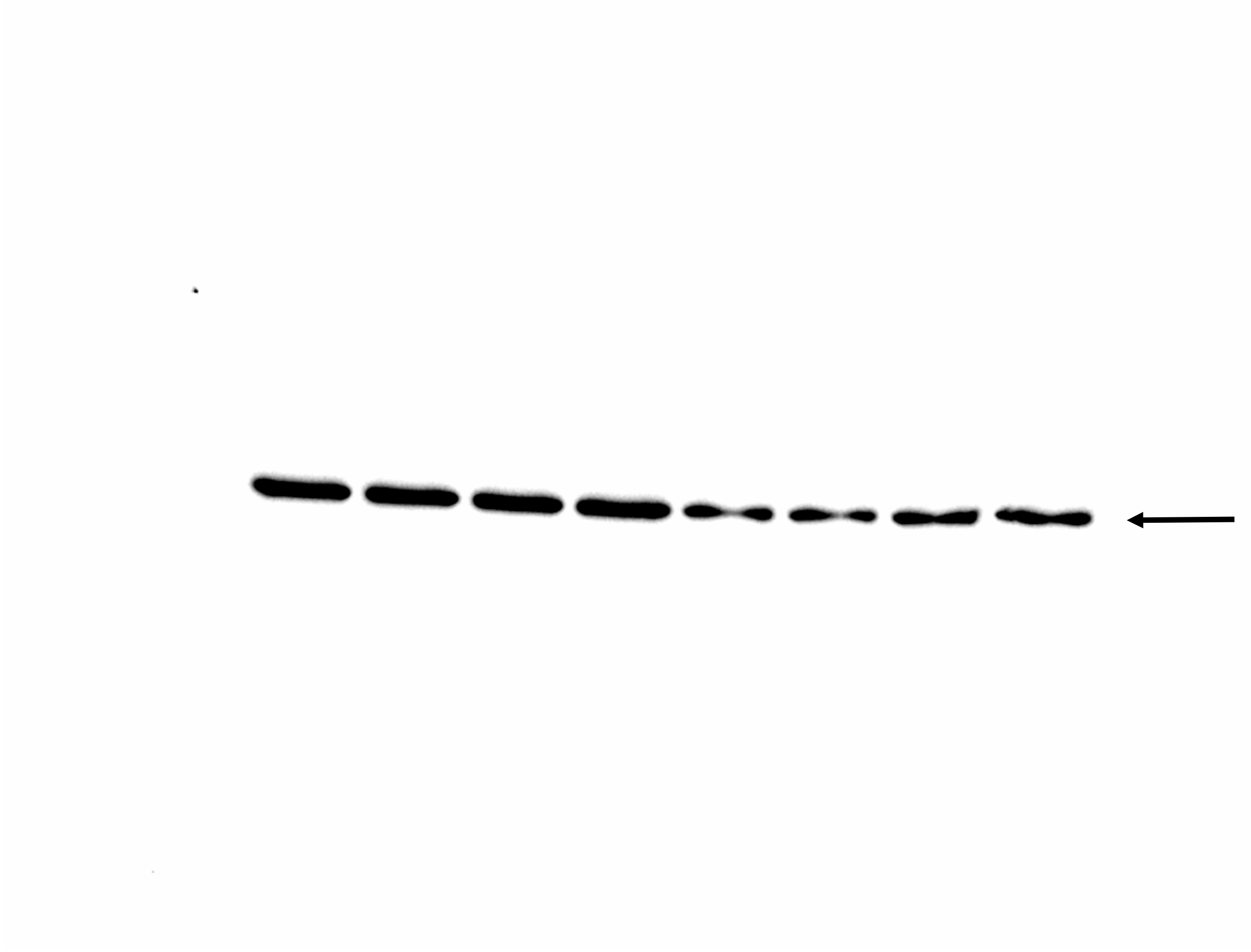

Figure 9A

Full unedited gel for Figure 9A

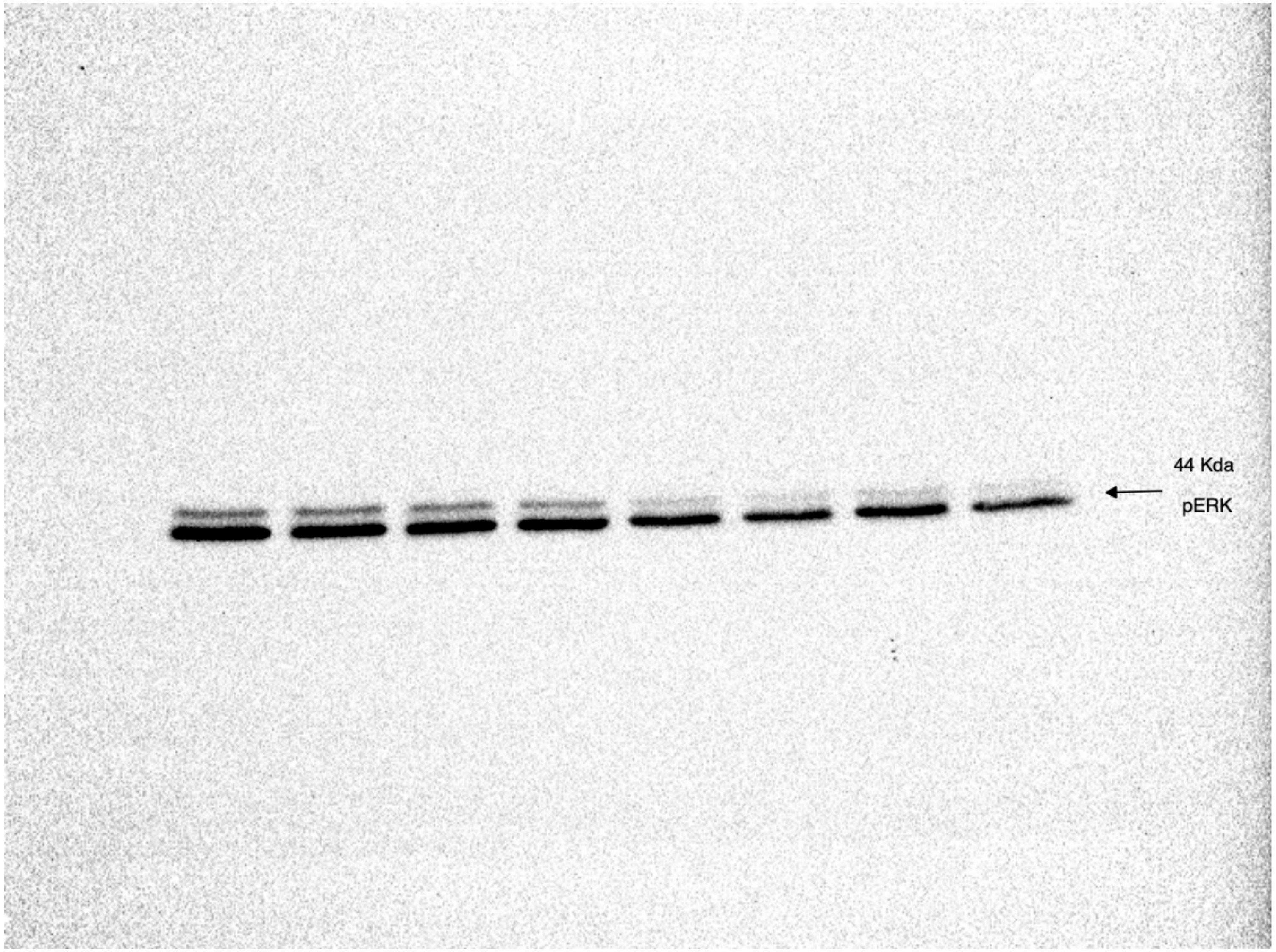

(p-ERK, Cell Signaling, 9101s, 1:1000)

Figure 9A

Full unedited gel for Figure 9A

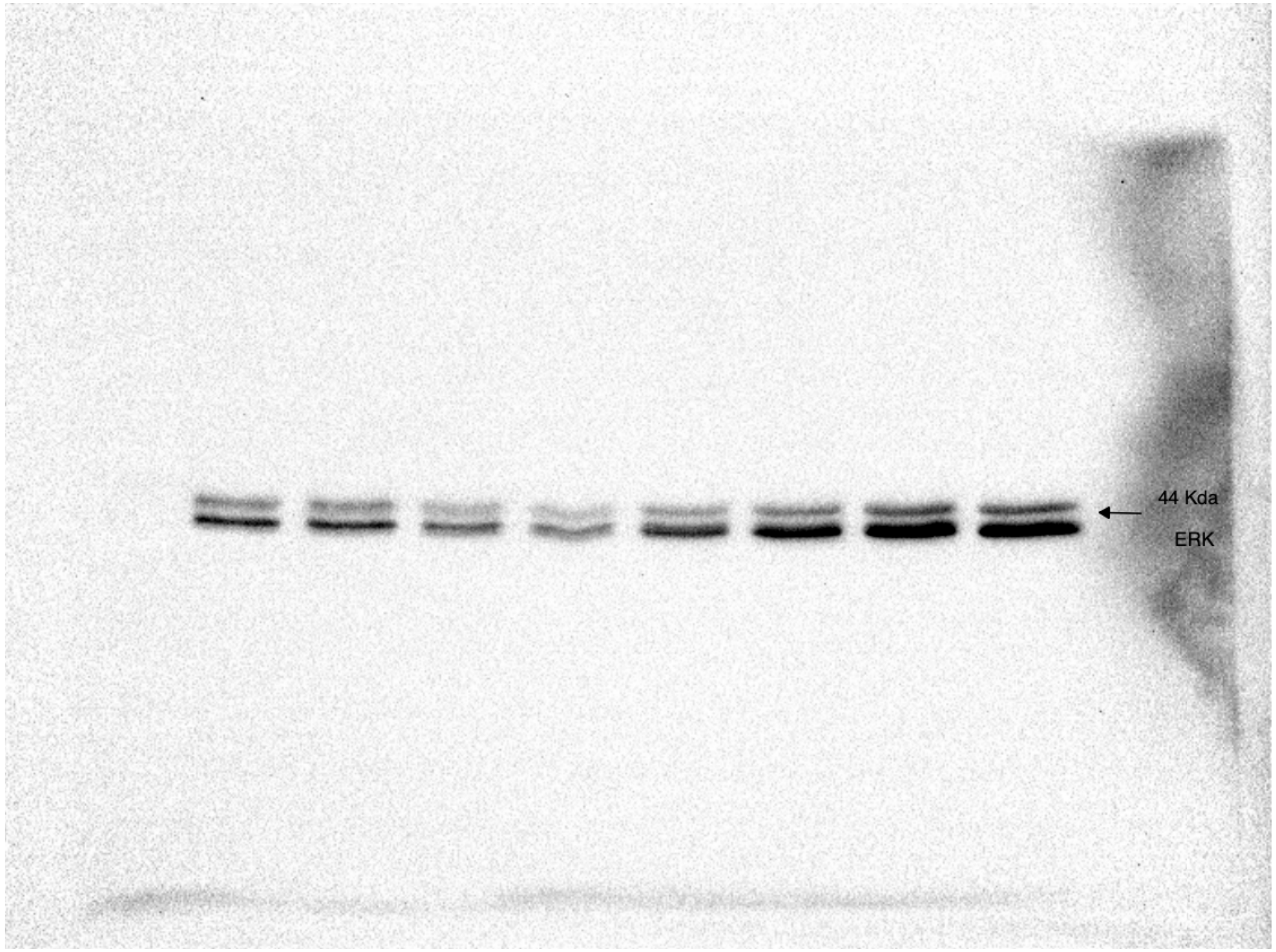

(ERK ½, Cell Signaling, 9102s, 1:1000)

Figure 9A

Full unedited gel for Figure 9A

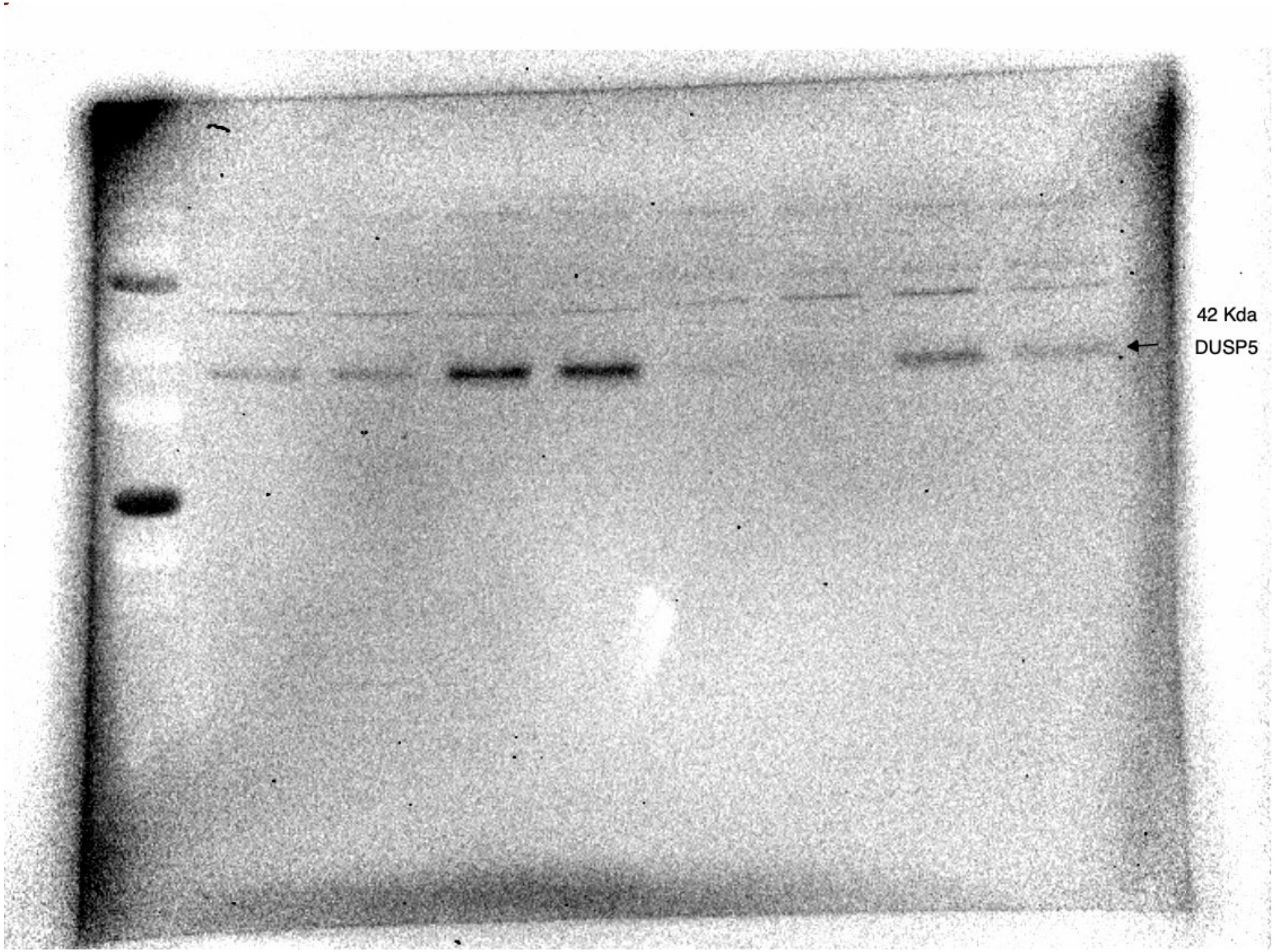

(DUSP5, Abcam, ab200708, 1:1000)

Figure 9A

Full unedited gel for Figure 9A

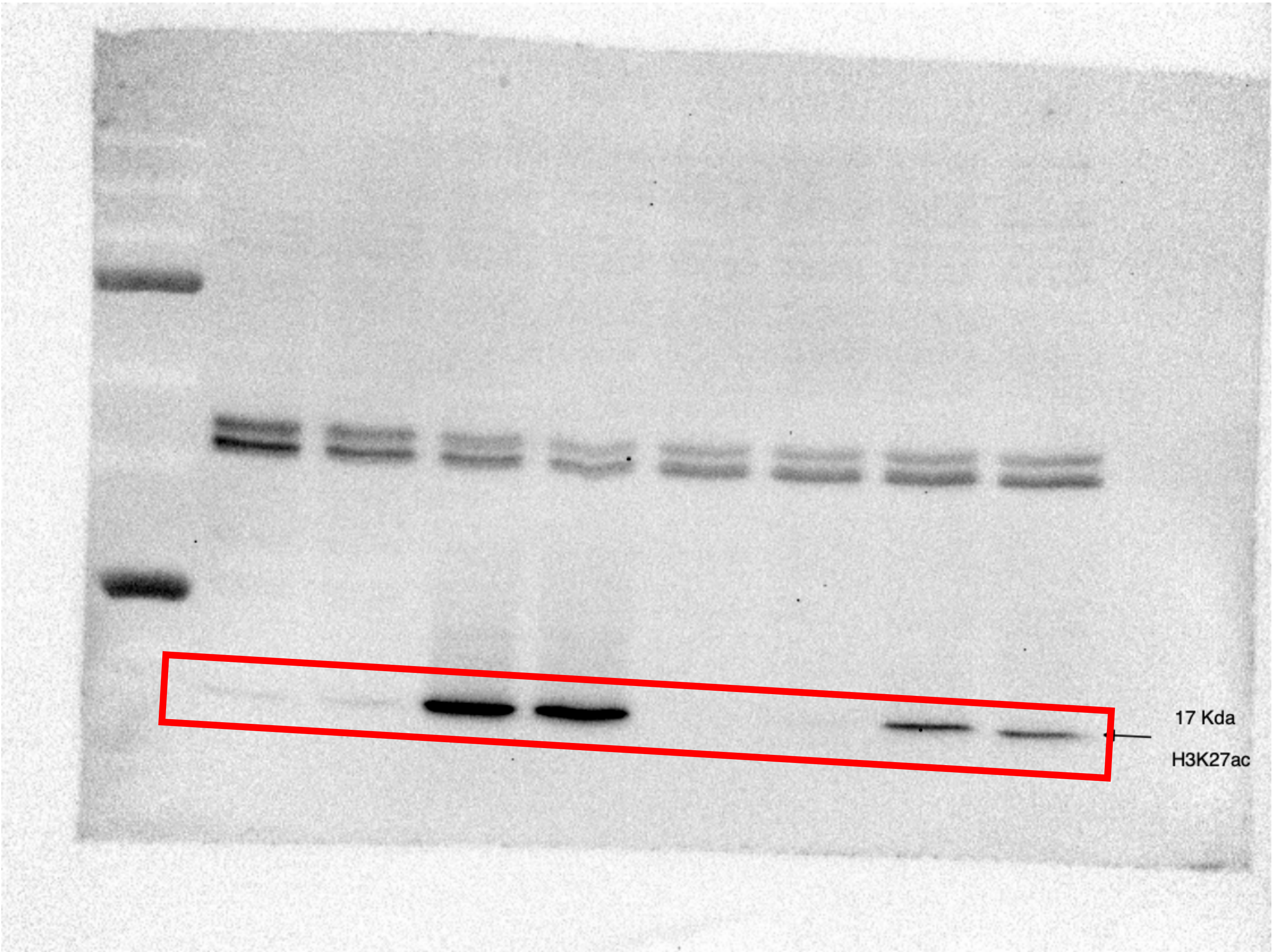

(H3K27ac,Abcam, ab4729, 1:1000)

Figure 9A

Full unedited gel for Figure 9A

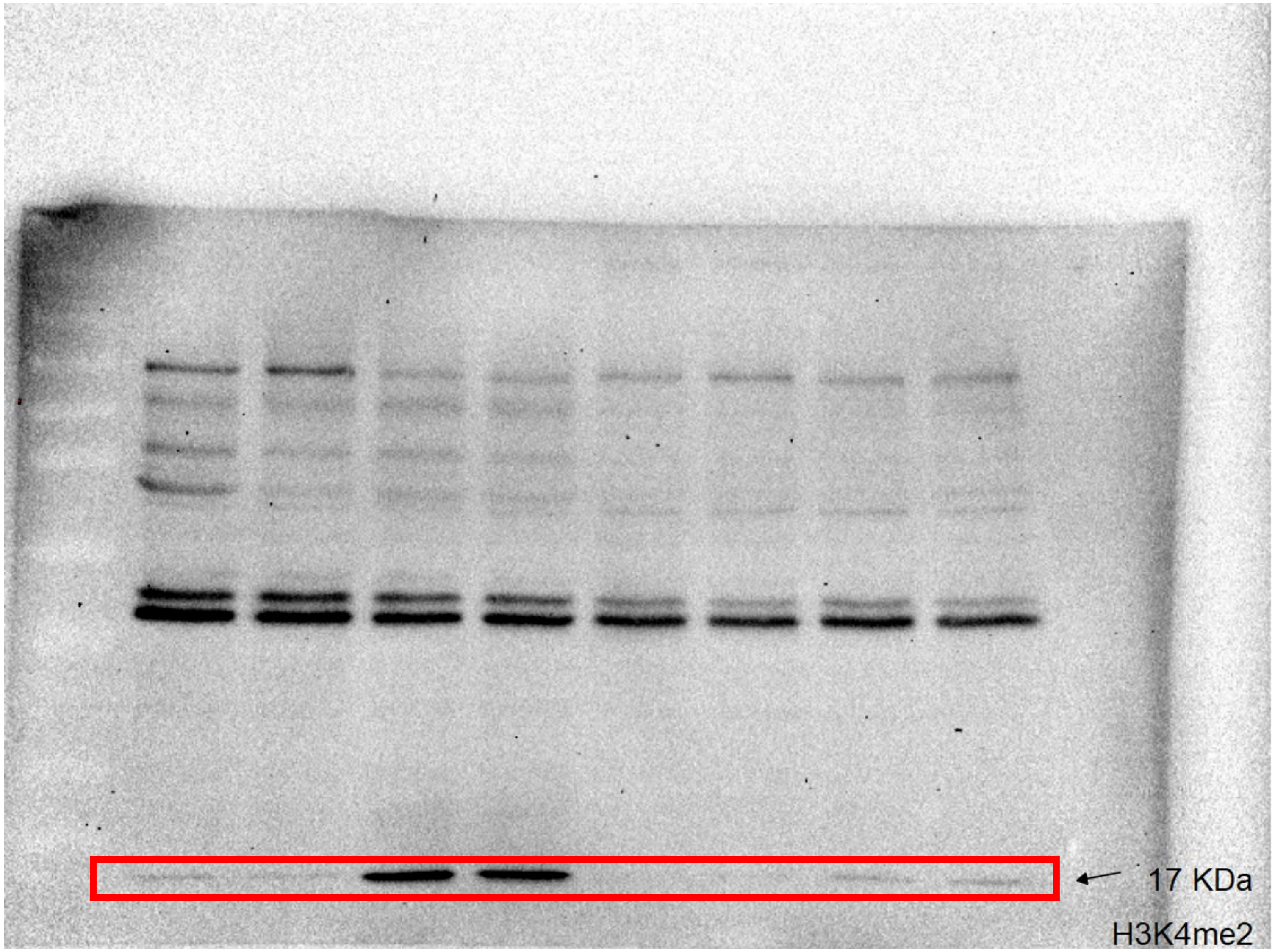

(H3K4me2, Cell Signaling, 2118L, 1:2000)

Figure 9A

Full unedited gel for Figure 9A

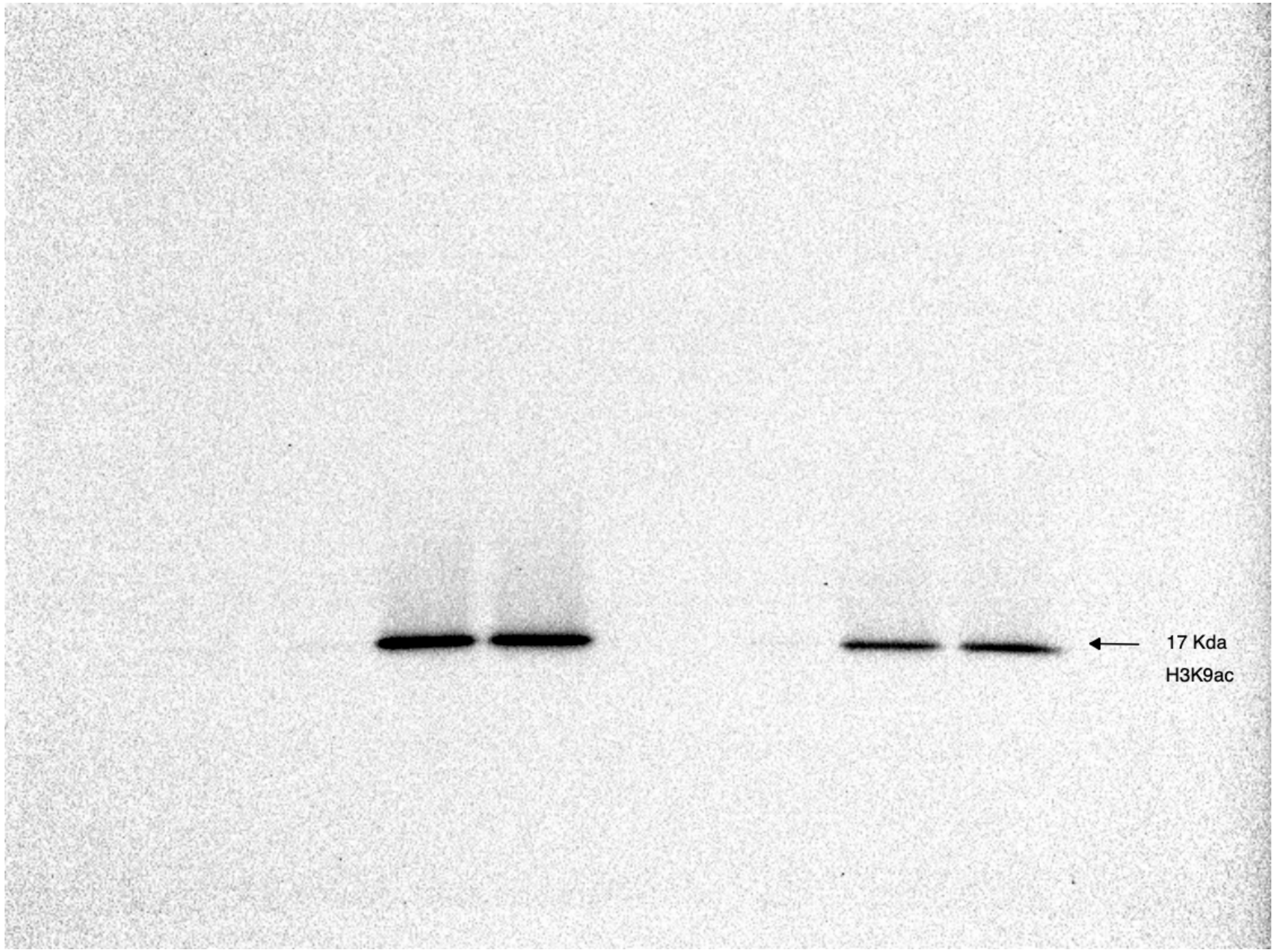

(H3K9ac, Abcam, ab32129, 1:1000)

Figure 9A

Full unedited gel for Figure 9A

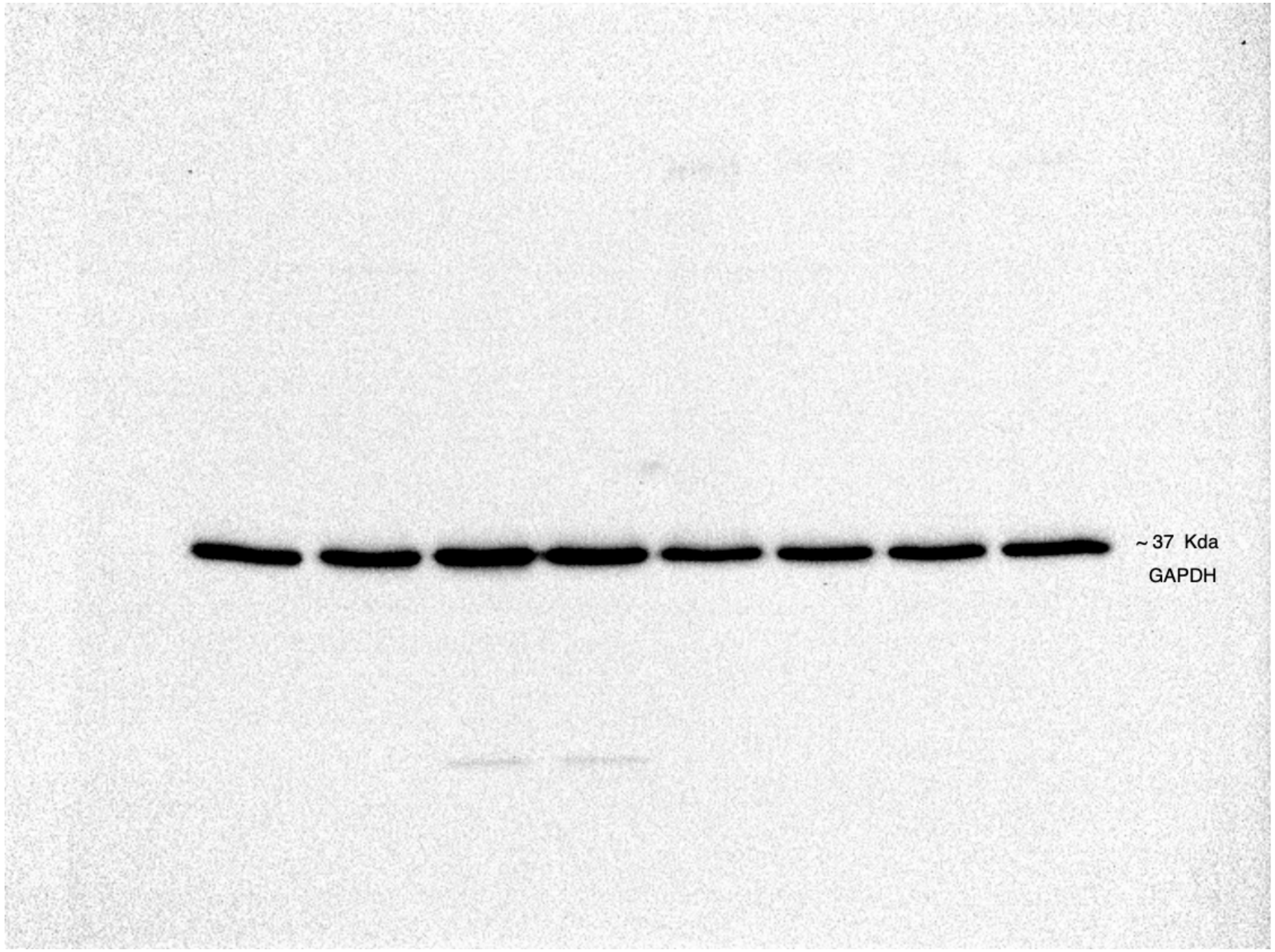

(GAPDH, Cell Signaling, 2118L, 1:2000)

Figure 9A

Full unedited gel for Figure 9A

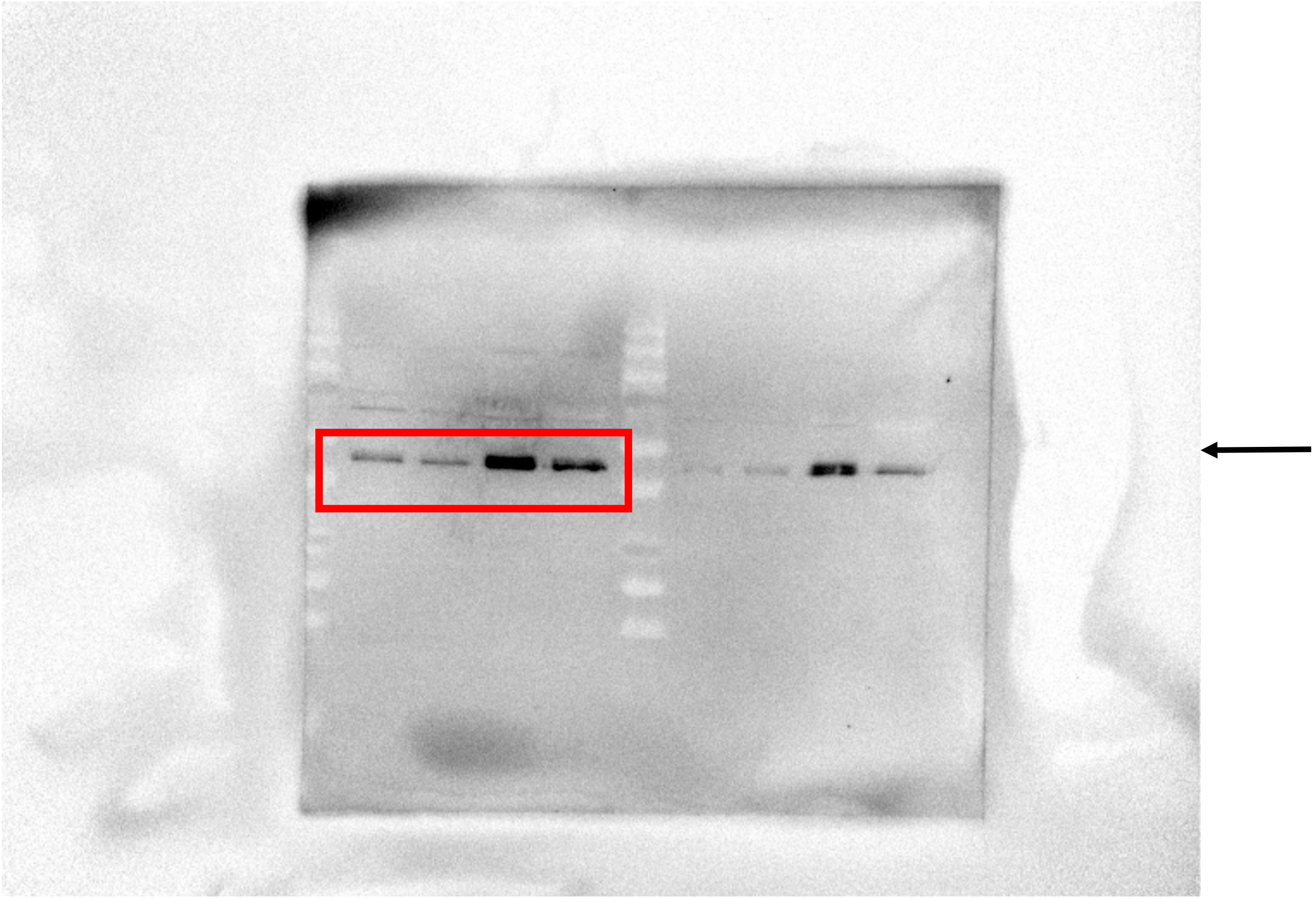

DUSP1
40 kDa
(Millipore, 07-535, 1:1000)

Figure 9A

Full unedited gel for Figure 9A

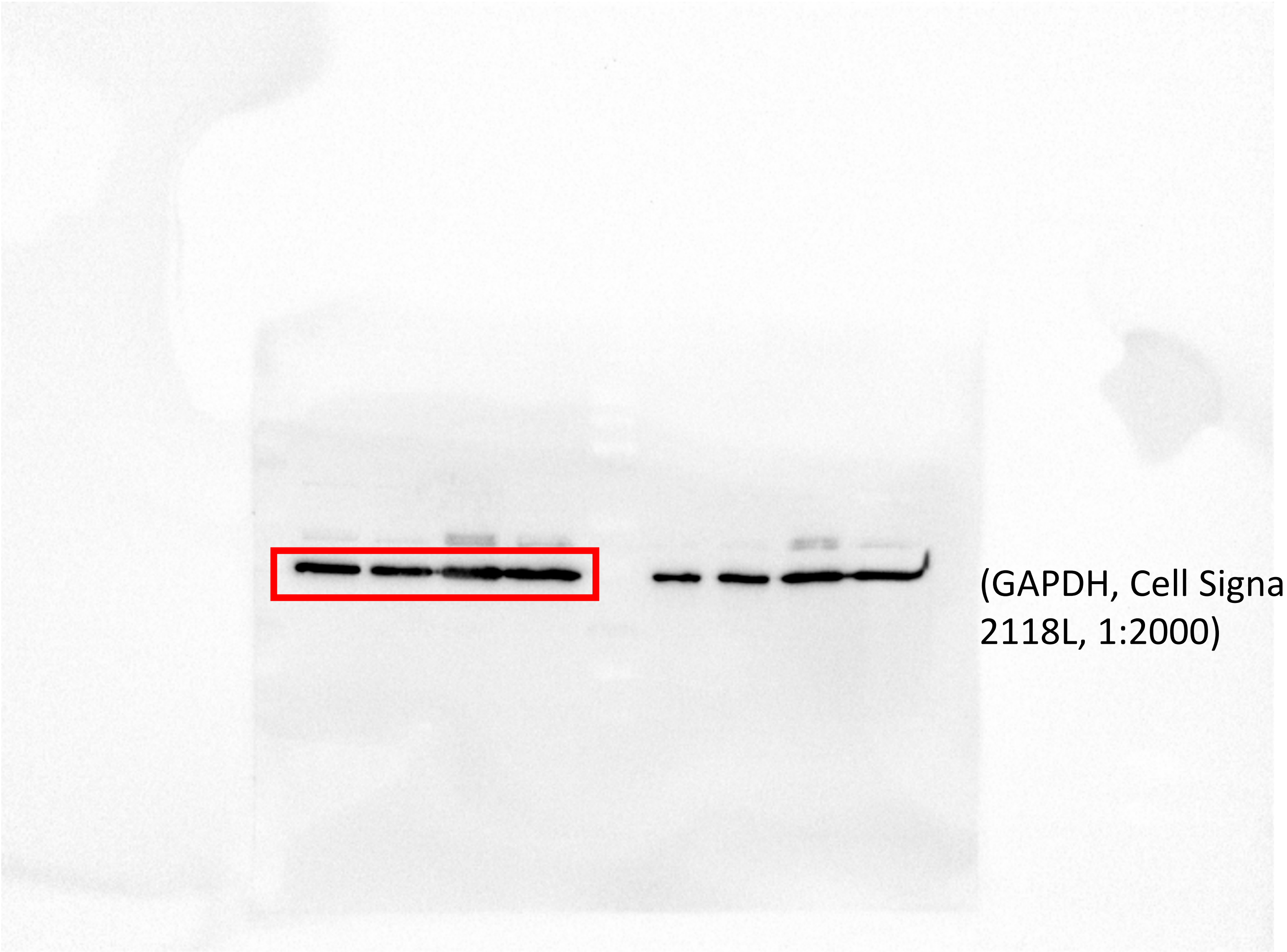

(GAPDH, Cell Signaling,
2118L, 1:2000)

Figure 9A

Full unedited gel for Figure 9A

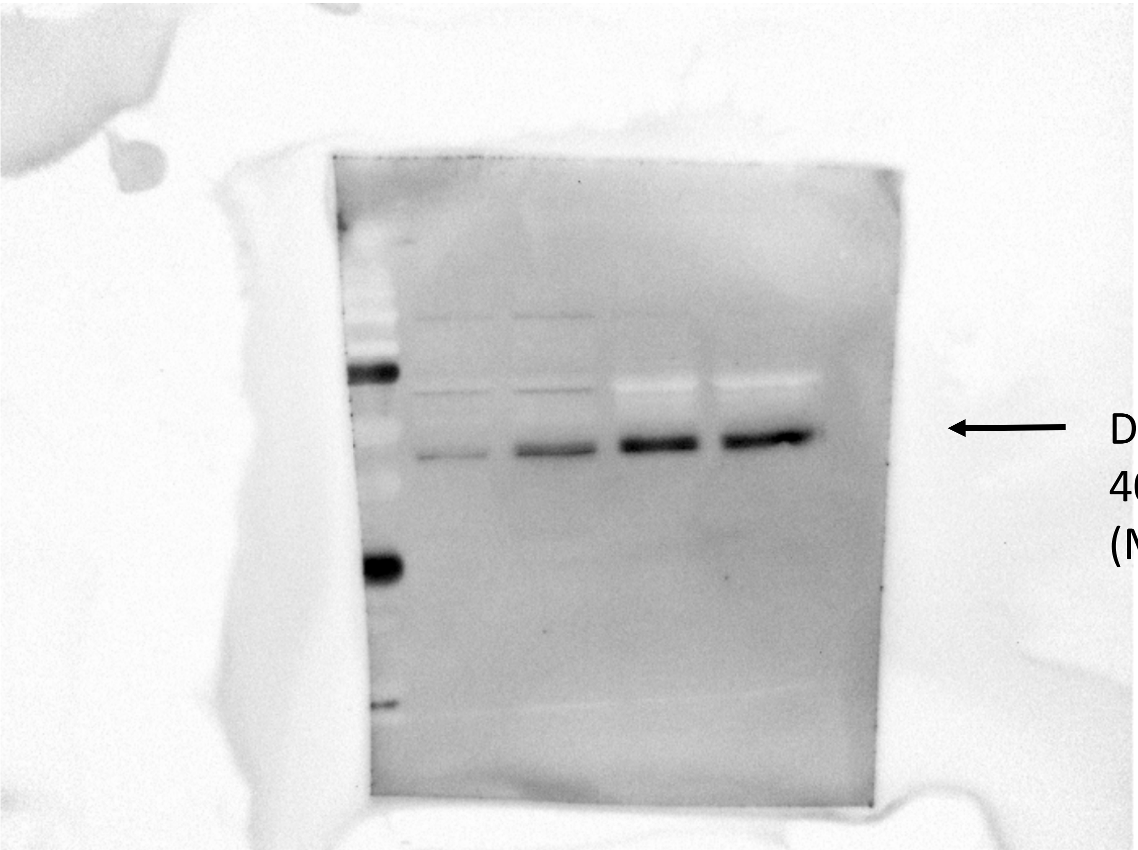

DUSP1
40 kDa
(Millipore, 07-535, 1:1000)

Figure 9A

Full unedited gel for Figure 9A

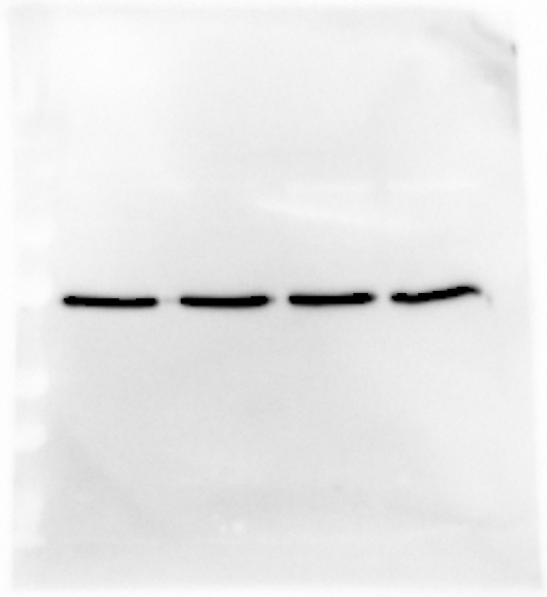

(GAPDH, Cell Signaling, 2118L, 1:2000)

Figure 9A

Full unedited gel for Figure 10C

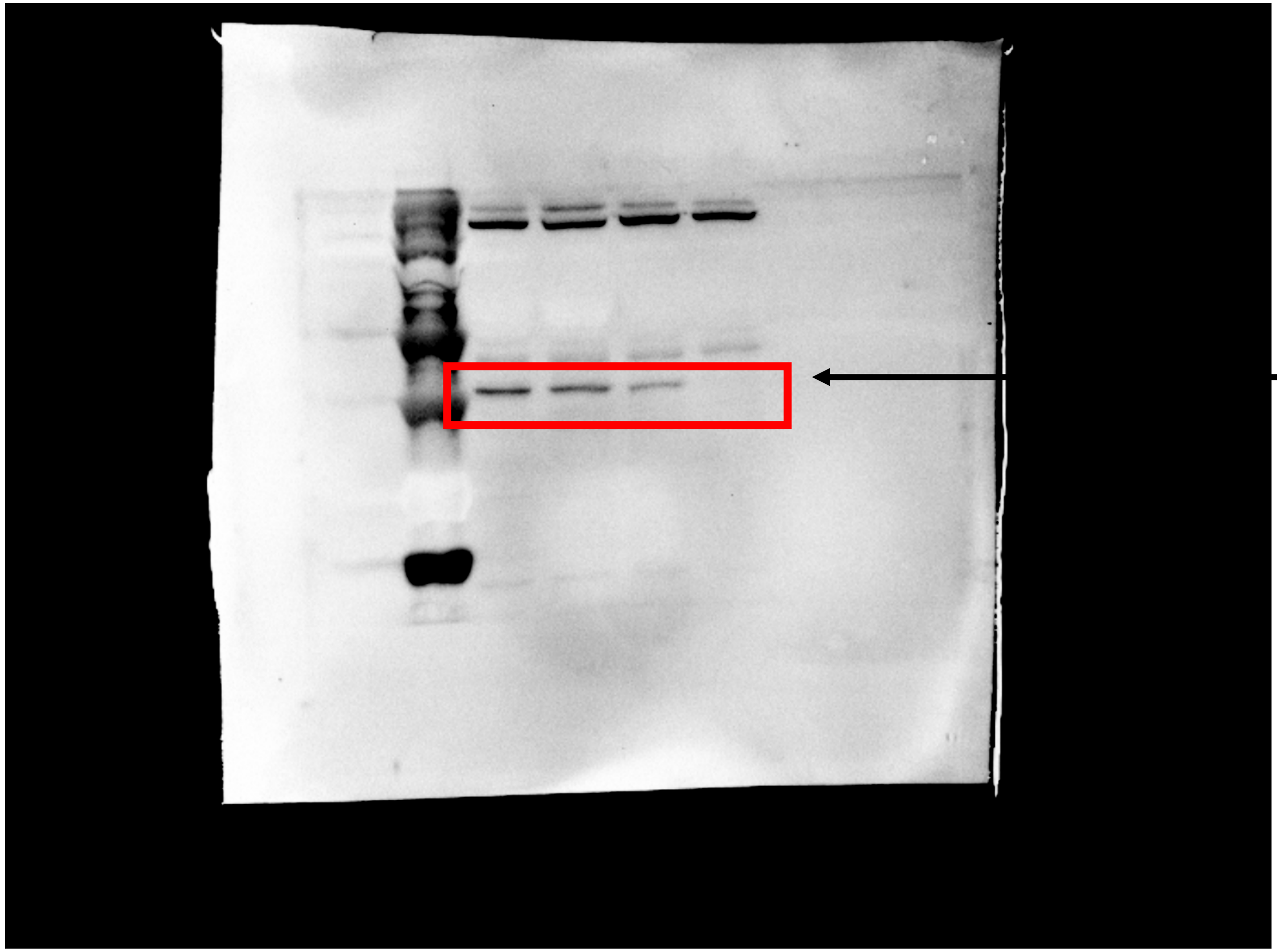

p-p38
43 kDa
(Cell Signaling, 4631s, 1:1000)

Figure 10C

Full unedited gel for Figure 10C

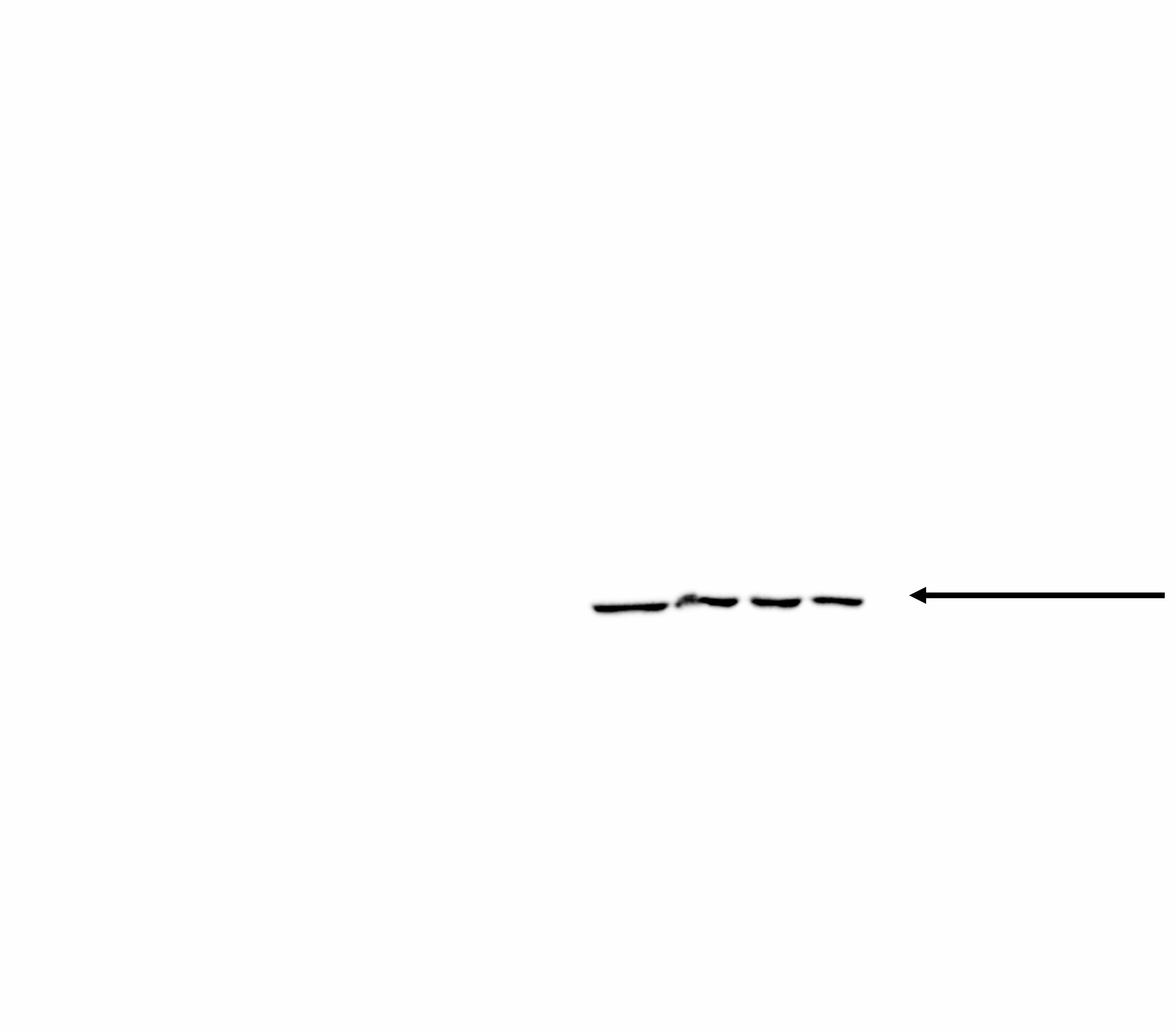

p38
40 kDa
(Cell Signaling, 9212s, 1:1000)

Figure 10C

Full unedited gel for Figure 10C

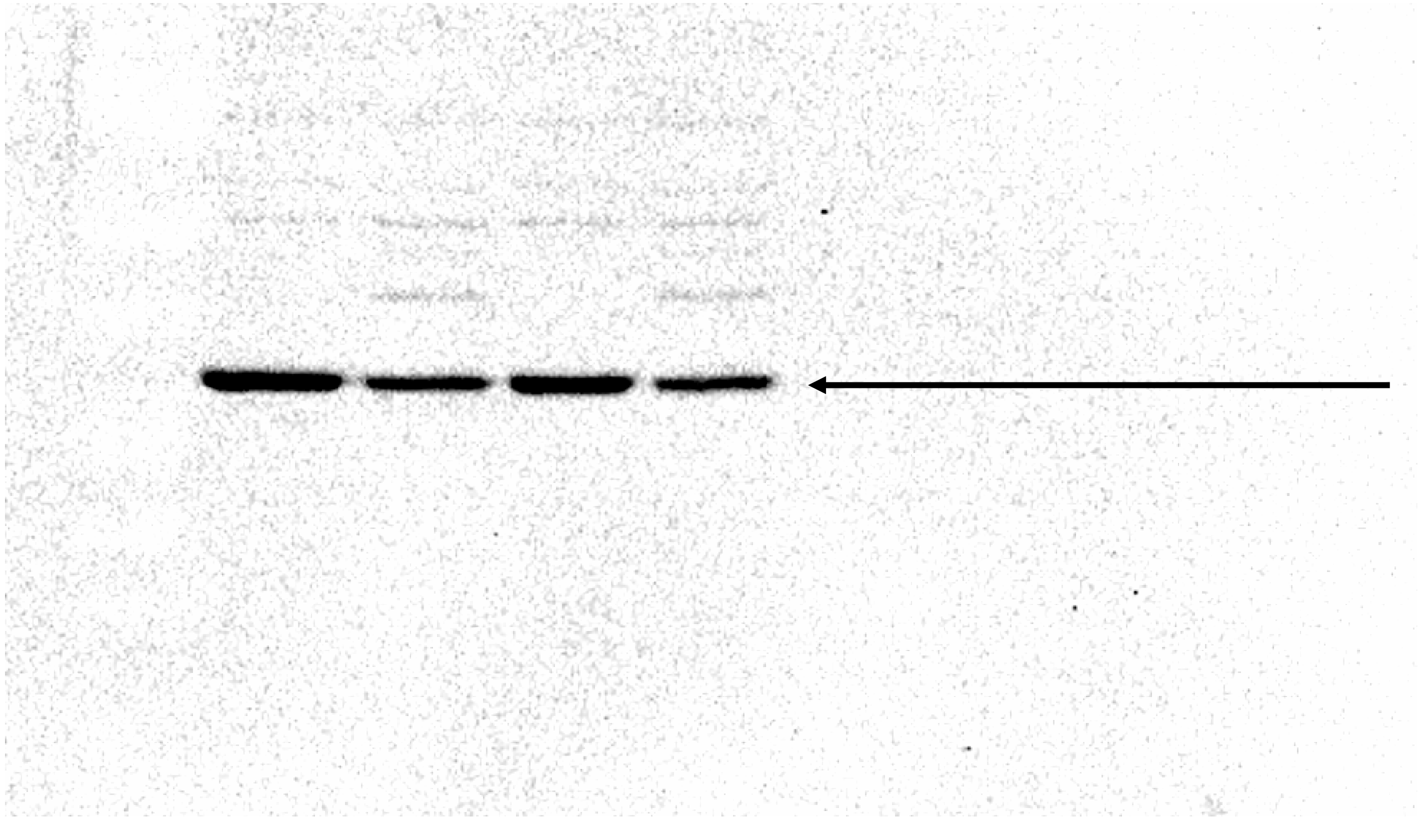

p-p38
43 kDa
(Cell Signaling, 4631s, 1:1000)

Figure 10C

Full unedited gel for Figure 10C

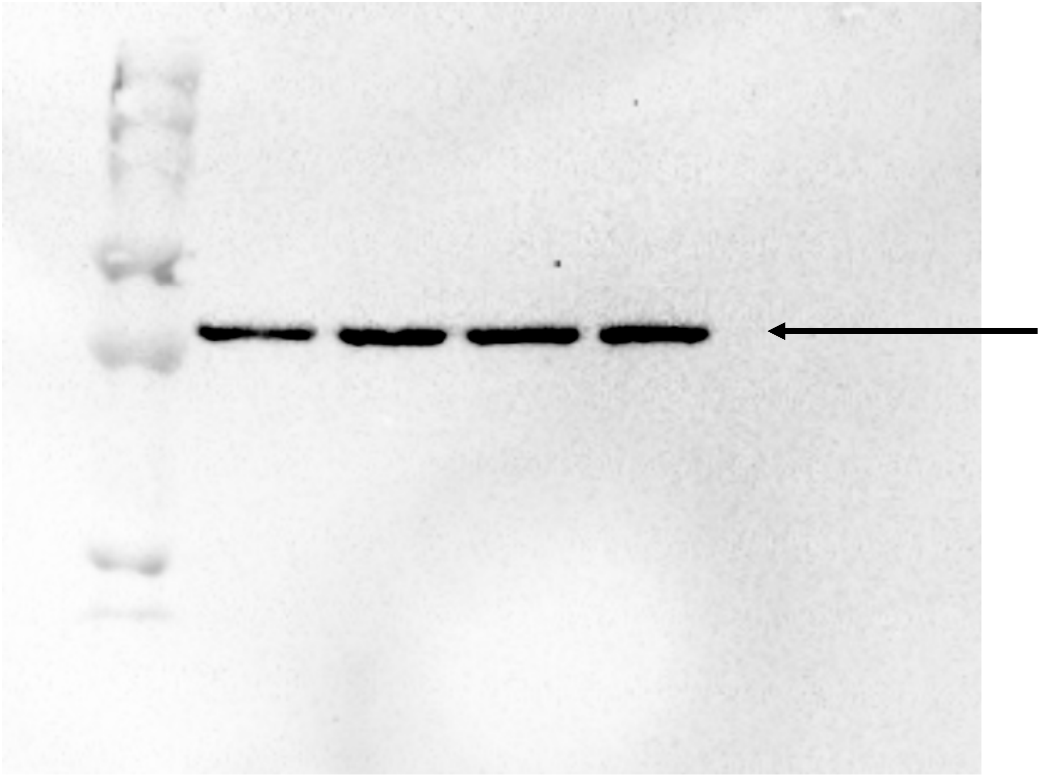

p38
40 kDa
(Cell Signaling, 9212s, 1:1000)

Figure 10C

Supplementary Figures

Full unedited gel for Supplementary Figure 1G

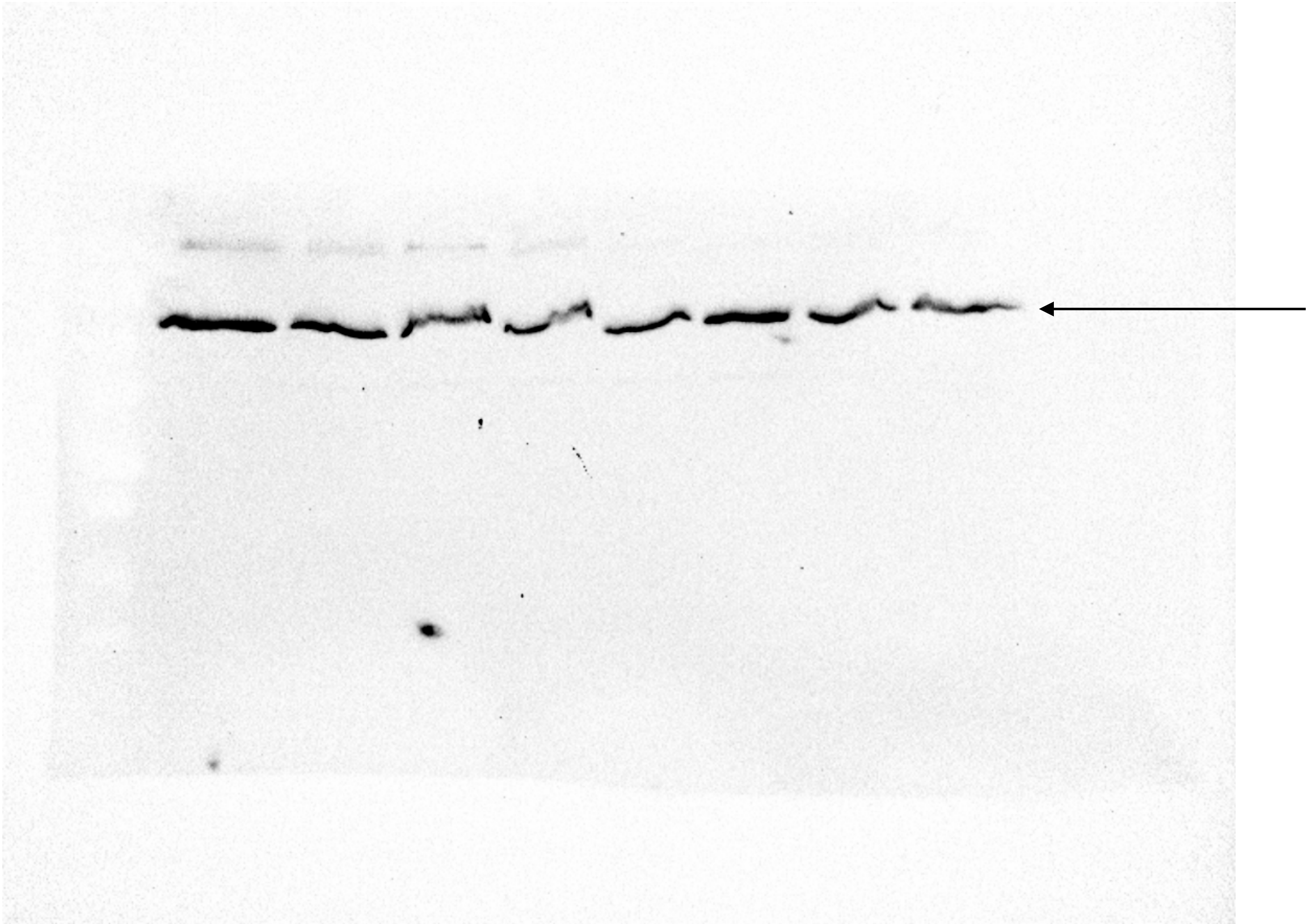

p62 kDa, p62

Supp Fig 1G

p62: 88588s, Cell Signaling

Full unedited gel for Supplementary Figure 1G

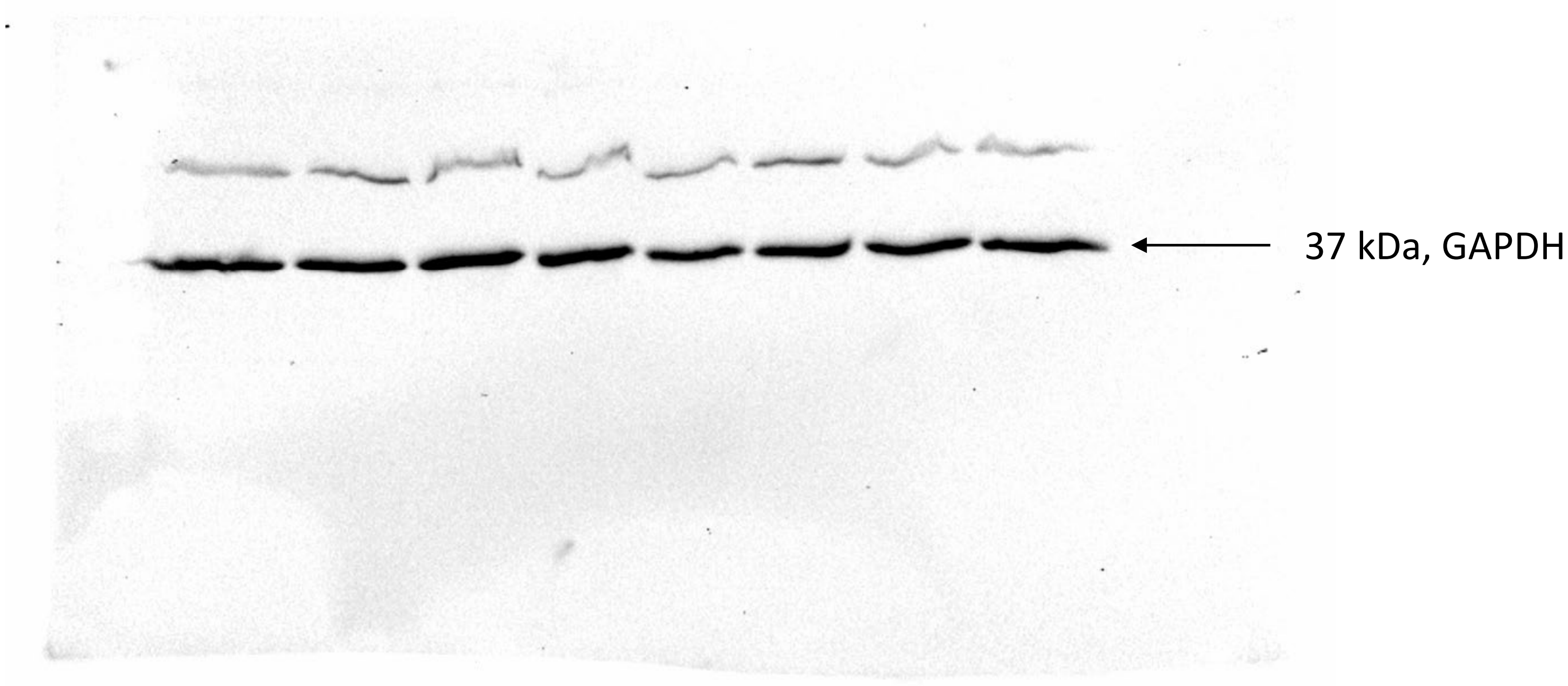

Supp Fig 1G

GAPDH: SC-365062, Santa Cruz

Full unedited gel for Supplementary Figure 1G

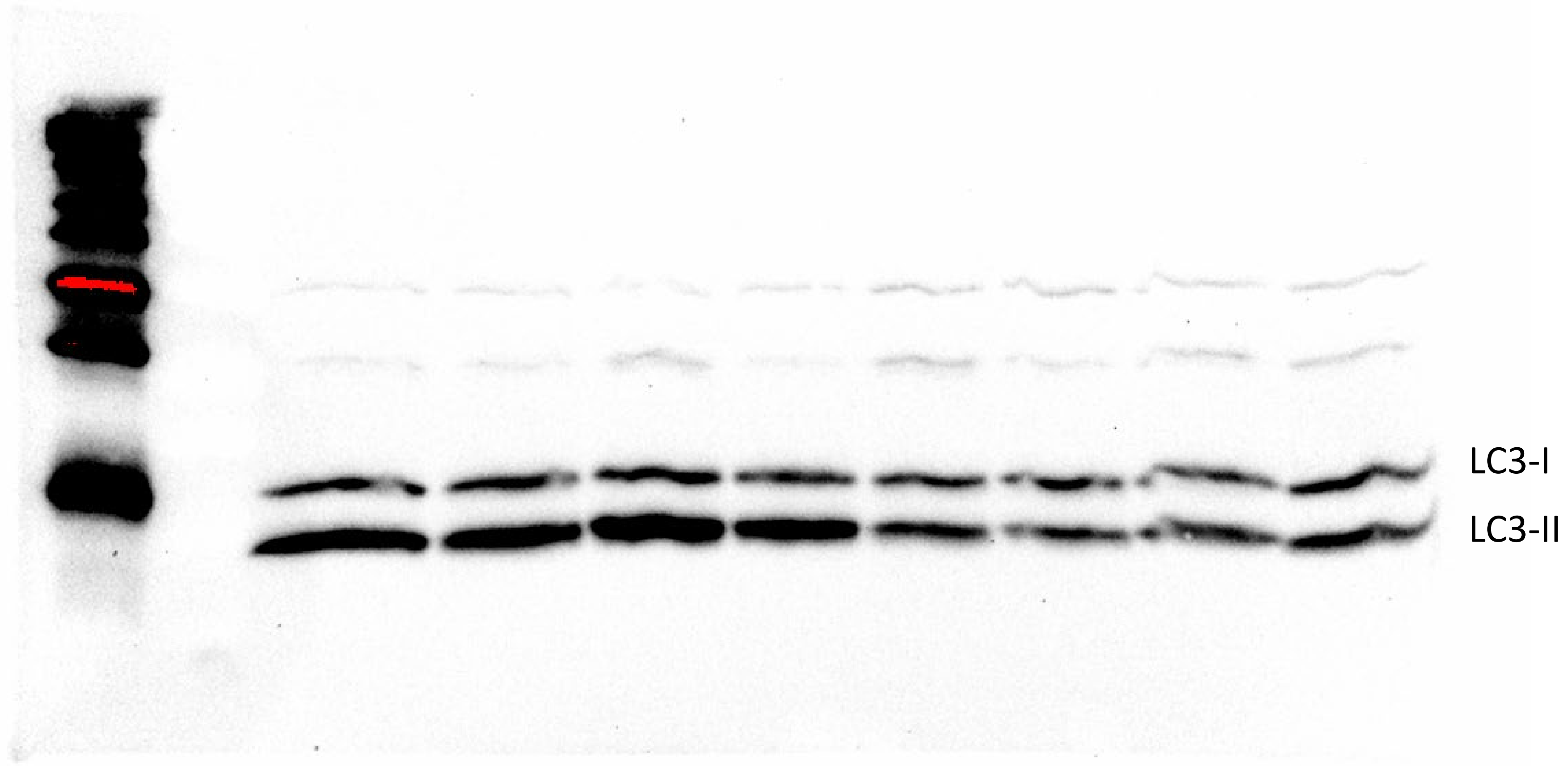

Supp Fig 1H

LC3B: 2775S, Cell Signaling

Full unedited gel for Supplementary Figure 1G

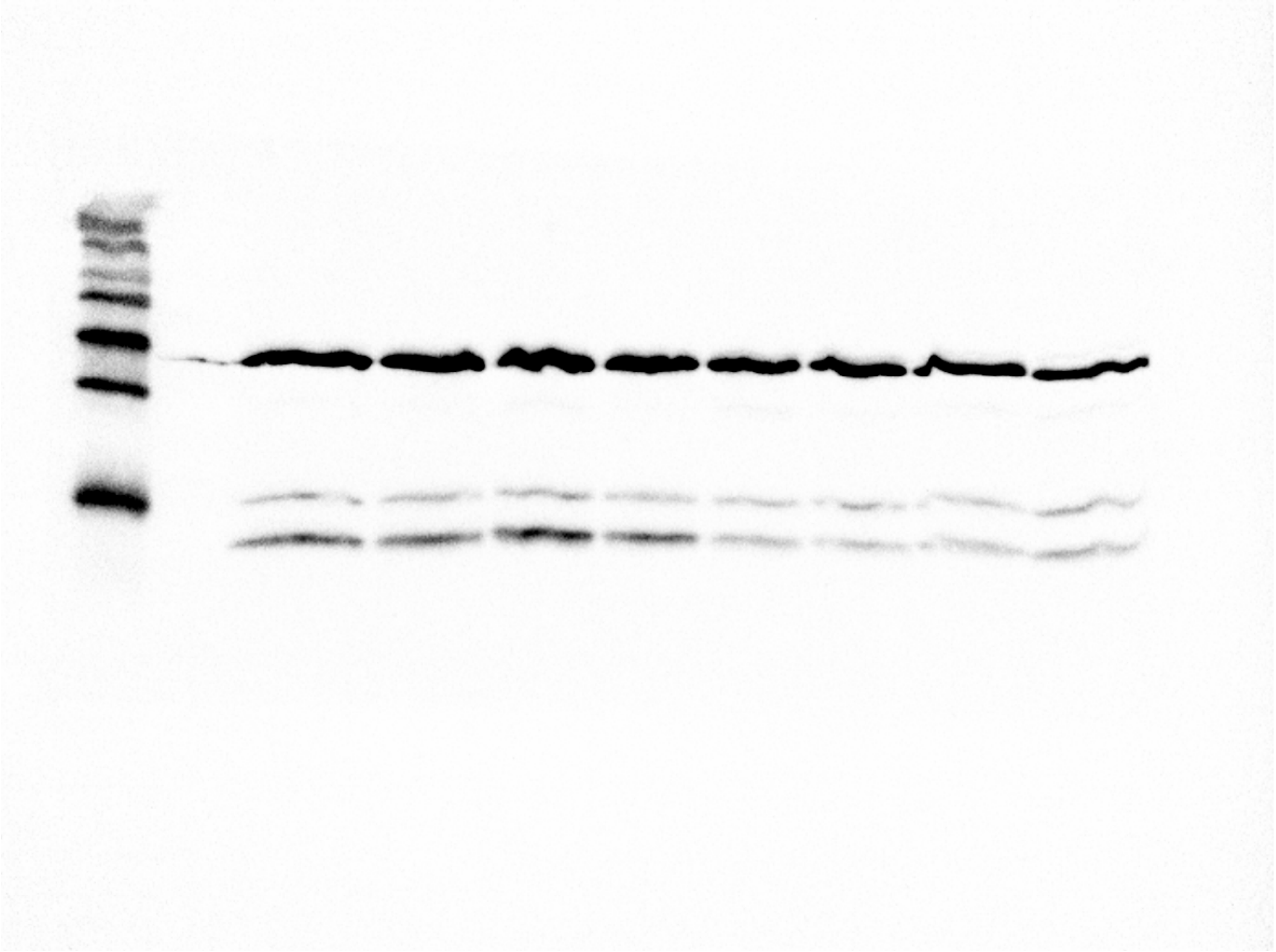

37 kDa, GAPDH

Supp Fig 1H

GAPDH: SC-365062,
Santa Cruz

Full unedited gel for Supplementary Figure 4A

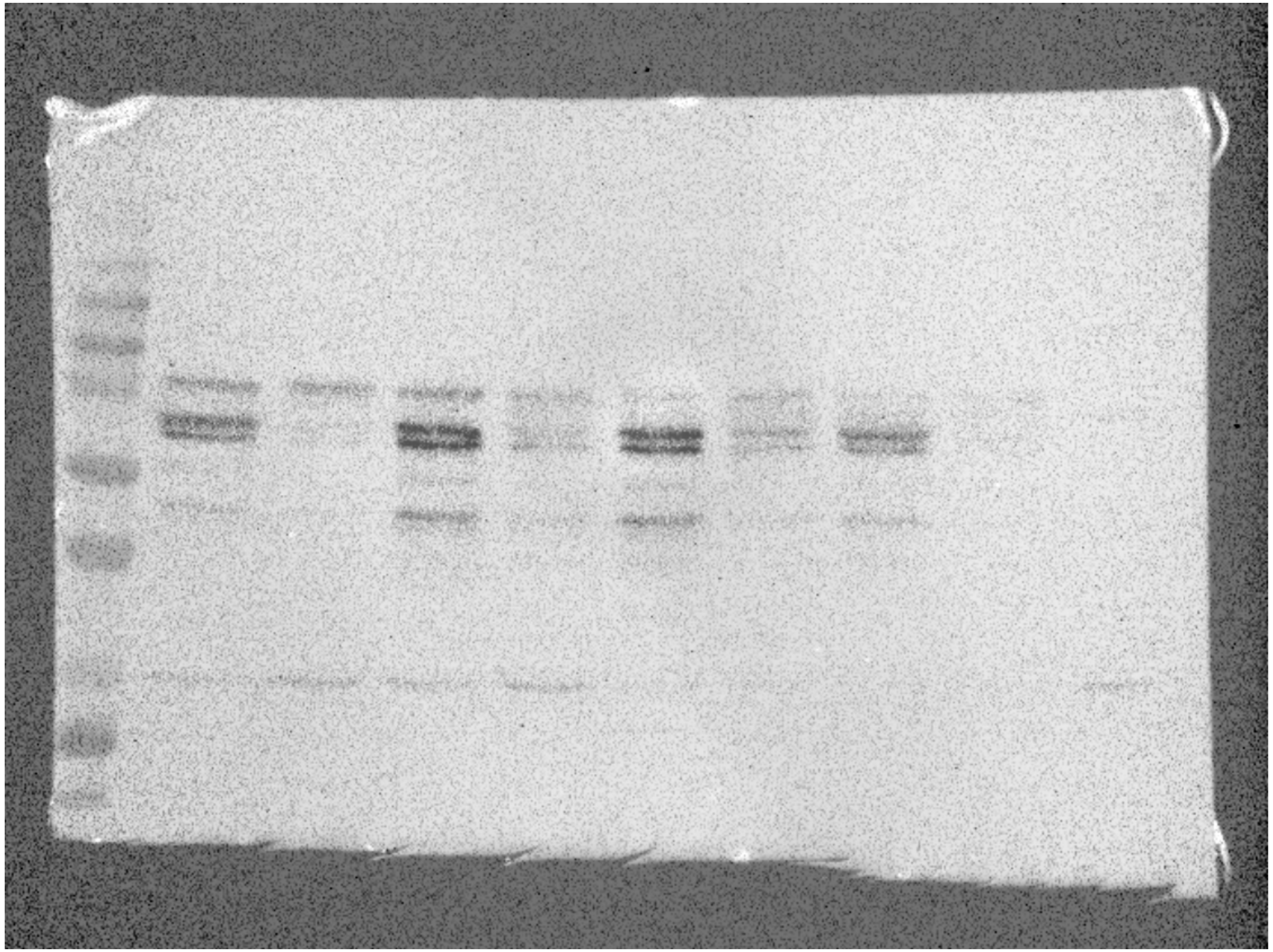

MITF
65 kDa
(Cell Signaling, 12590s, 1:1000)

Supp Fig 4A

Full unedited gel for Supplementary Figure 4A

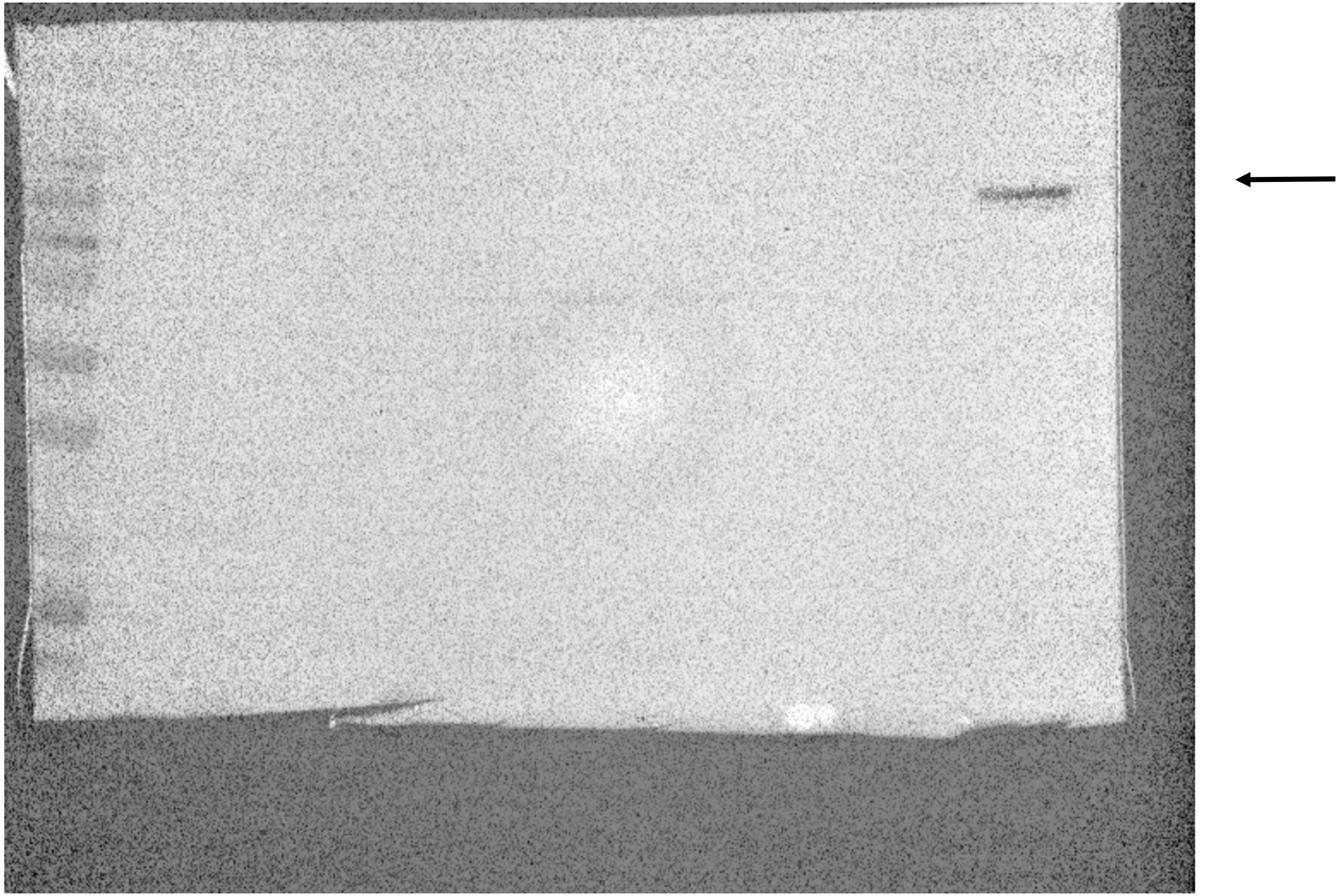

AXL
138 kDa
(Cell Signaling, 8661s, 1:1000)

AXL was run in an additional cell line (1205Lu) as a positive control. 1205Lu cells have high AXL expression. This is to prove that the absence of AXL bands in the other cell lines is due to the biology and not the quality of the antibody.

Full unedited gel for Supplementary Figure 4A

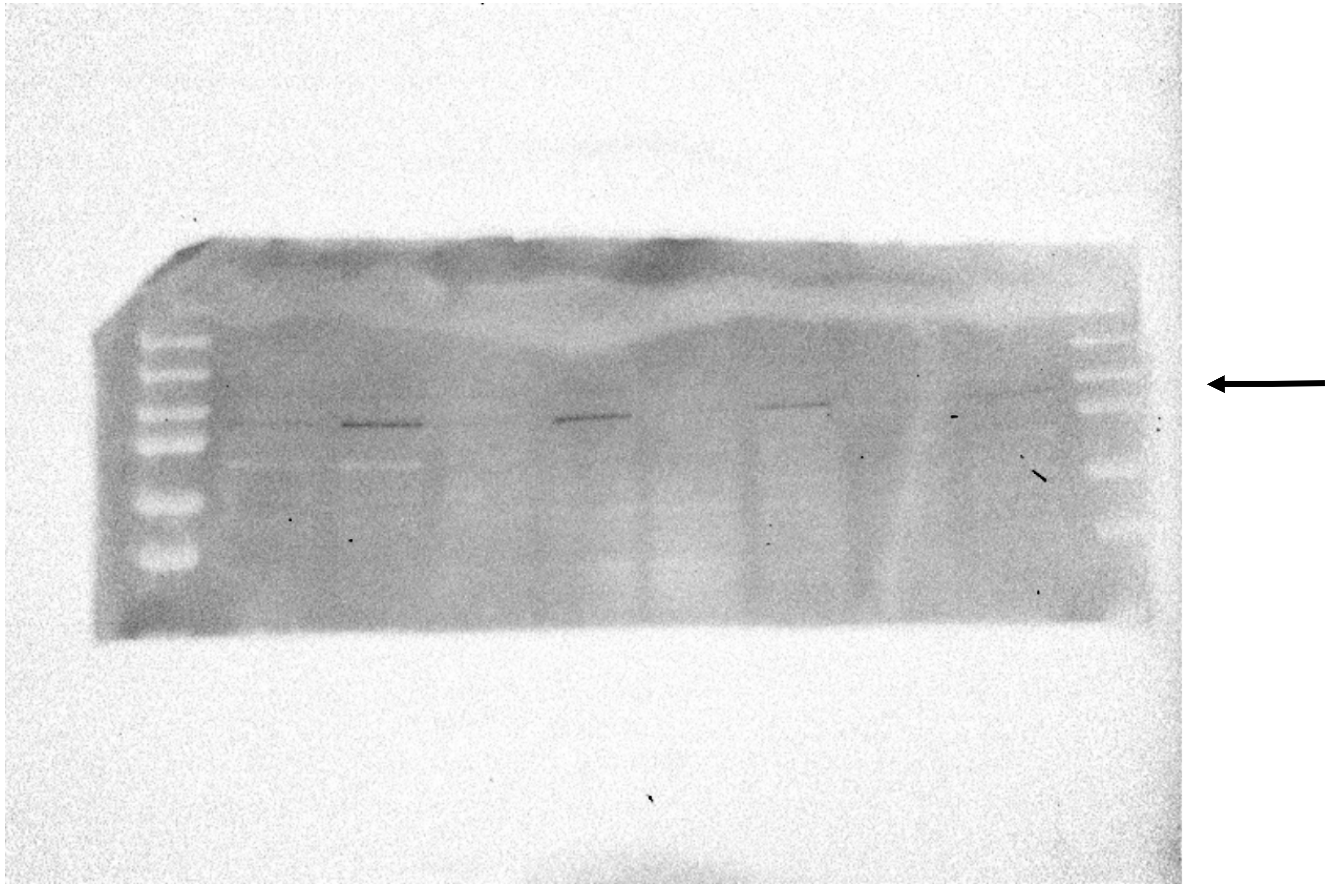

Supp Fig 4A

Full unedited gel for Supplementary Figure 4A

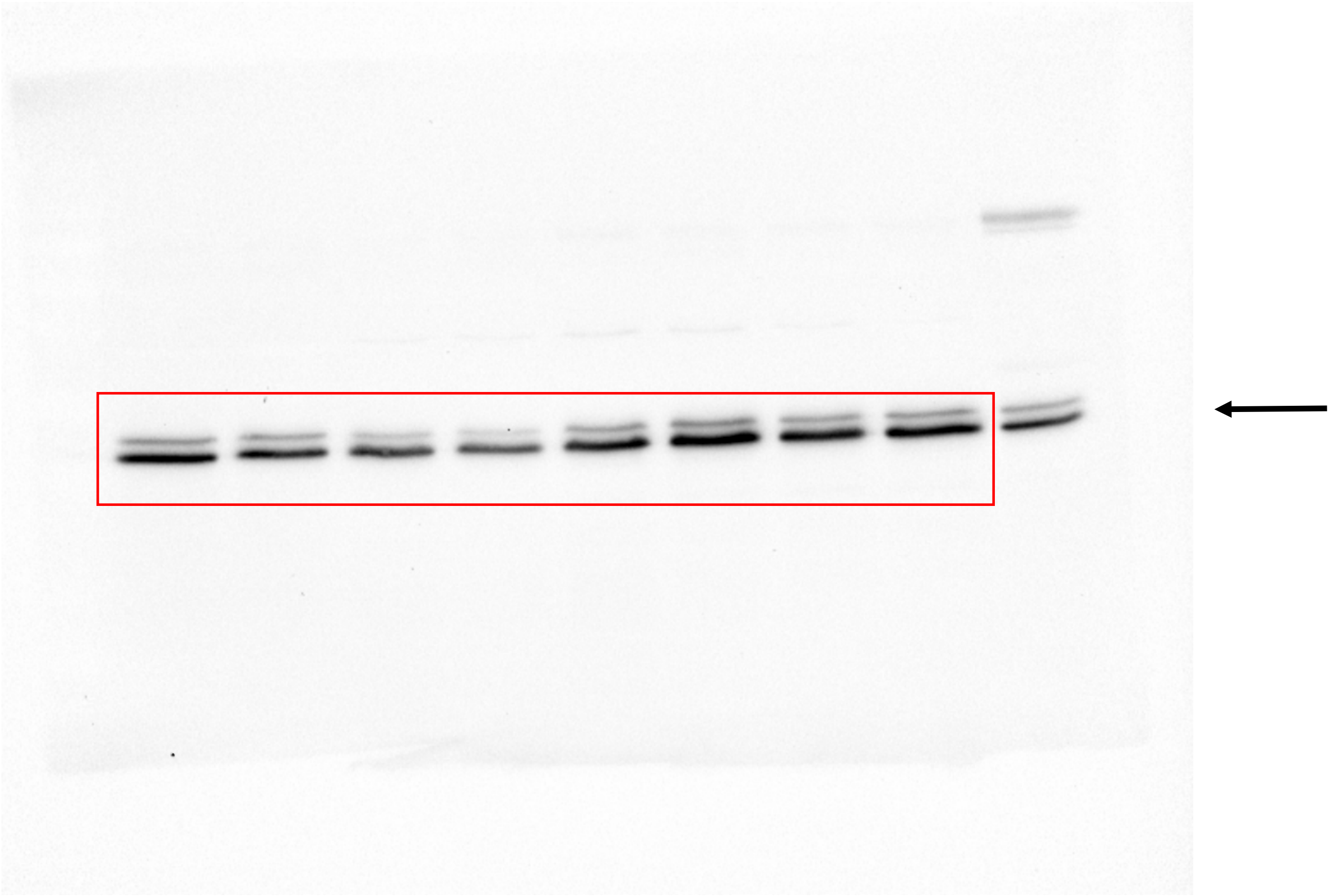

p-ERK
42,44 kDa
(Cell Signaling, 9101s, 1:1000)

Supp Fig 4A

Full unedited gel for Supplementary Figure 4A

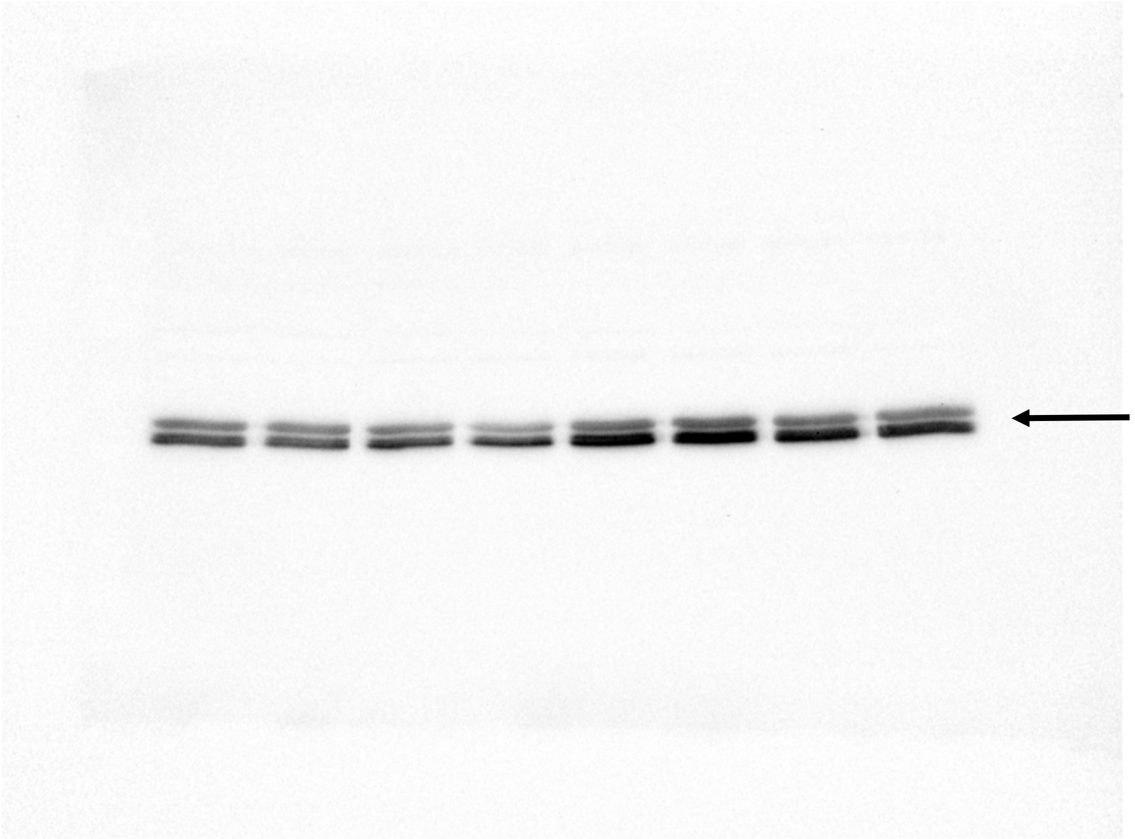

ERK – p44/42 MAPK
42,44 kDa
(Cell Signaling, 9102s, 1:1000)

Supp Fig 4A

Full unedited gel for Supplementary Figure 4A

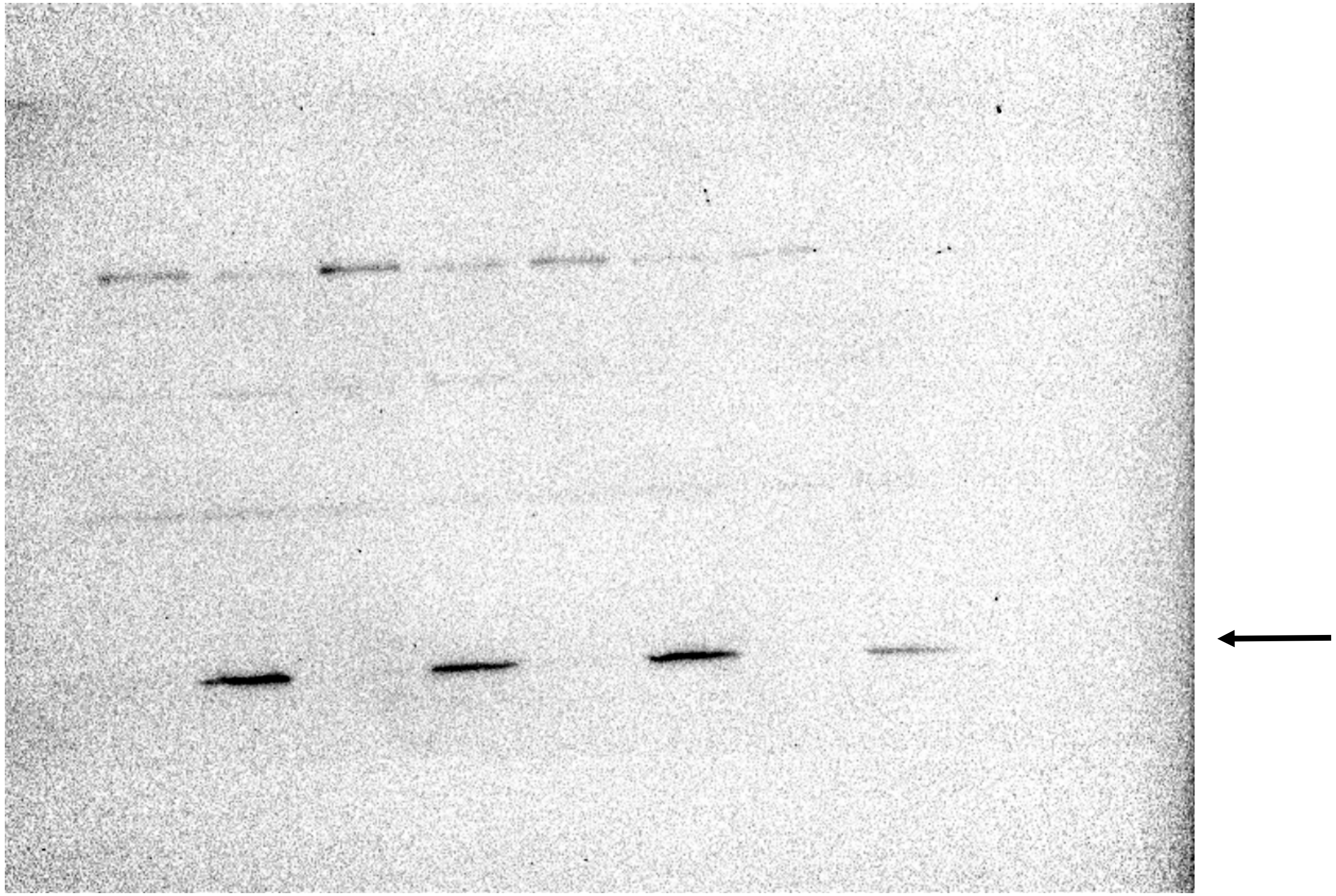

Full unedited gel for Supplementary Figure 4A

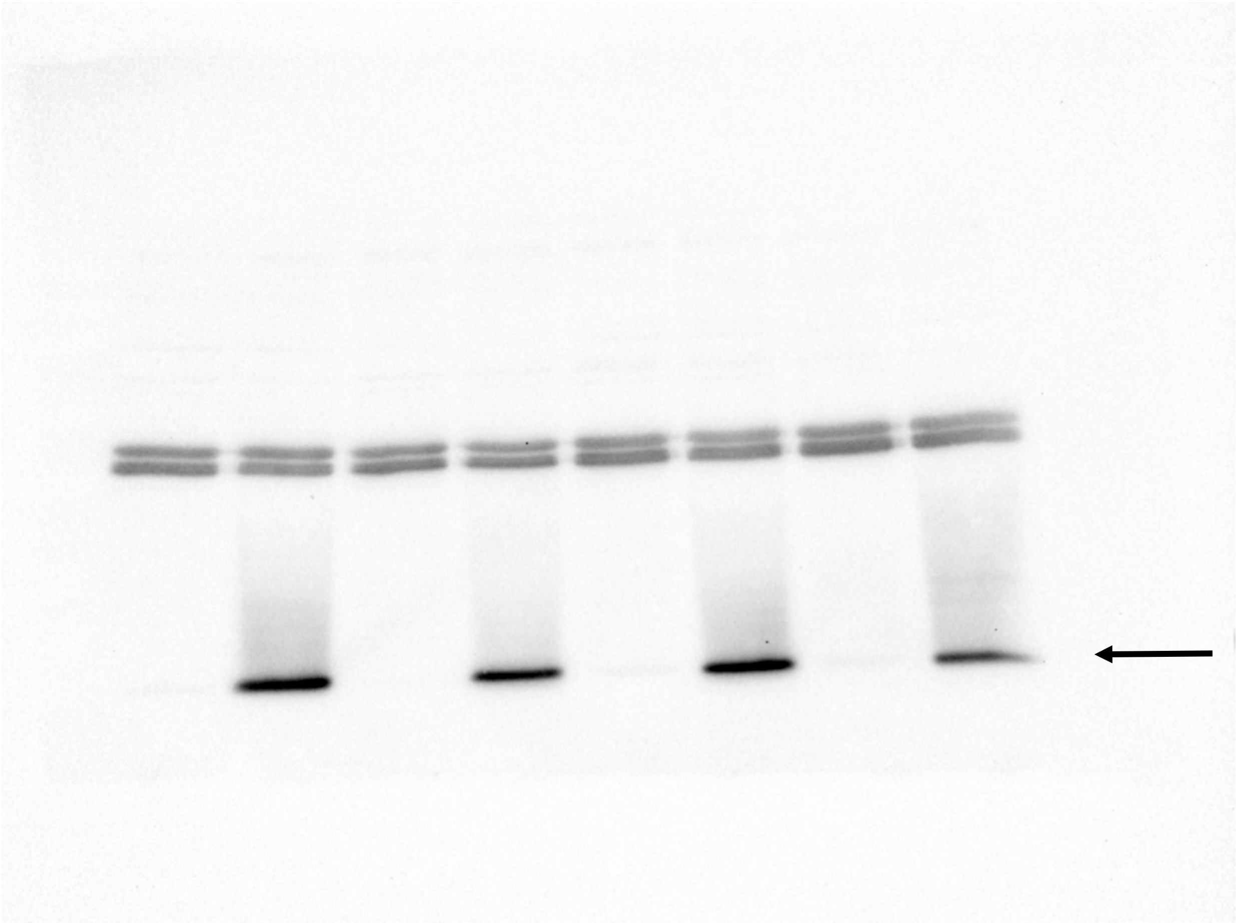

H3K9ac
18 kDa
(Abcam, ab32129, 1:1000)

Supp Fig 4A

Full unedited gel for Supplementary Figure 4A

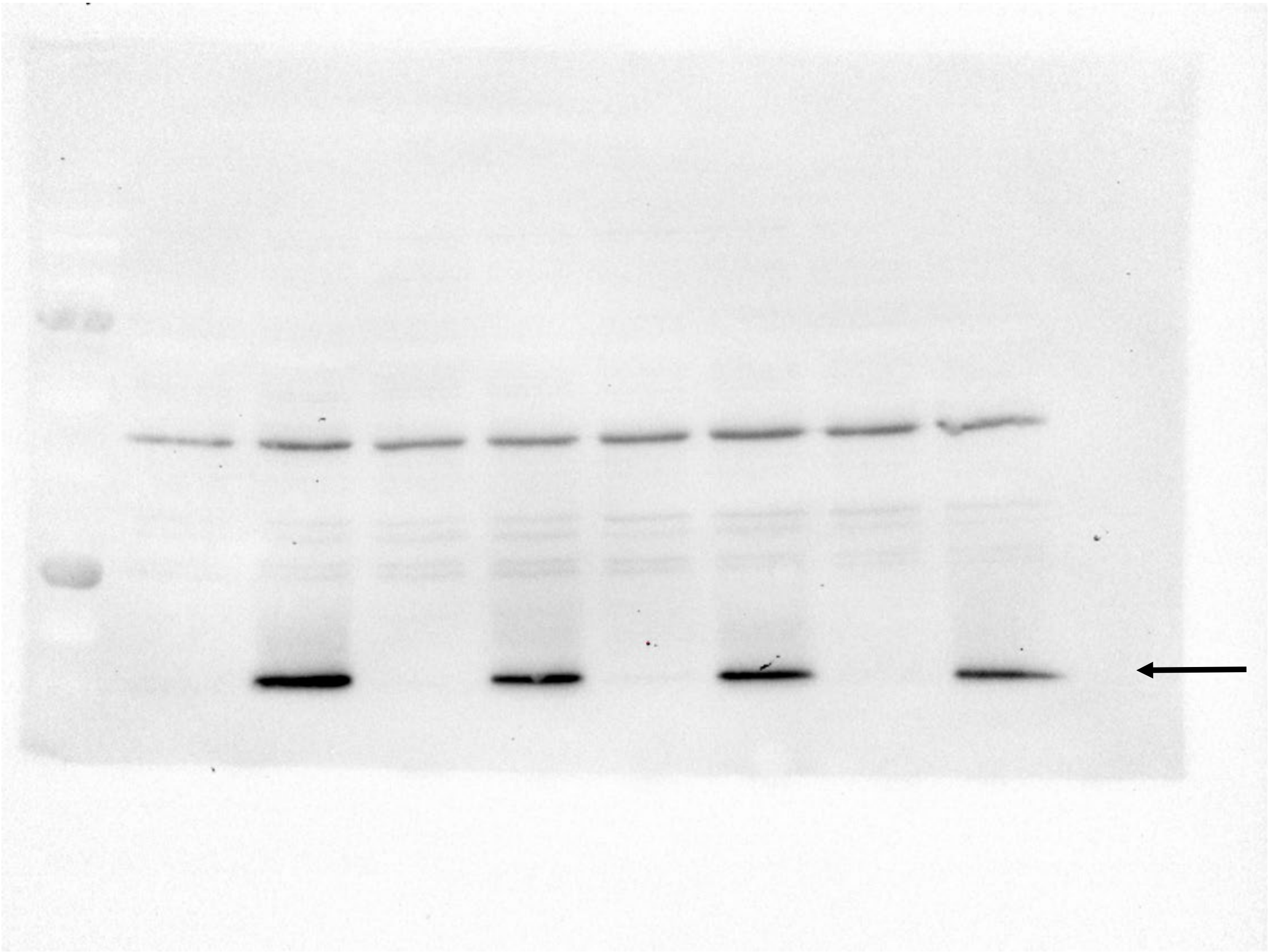

H3K27ac
15 kDa
(Abcam, ab4729, 1:1000)

Full unedited gel for Supplementary Figure 4A

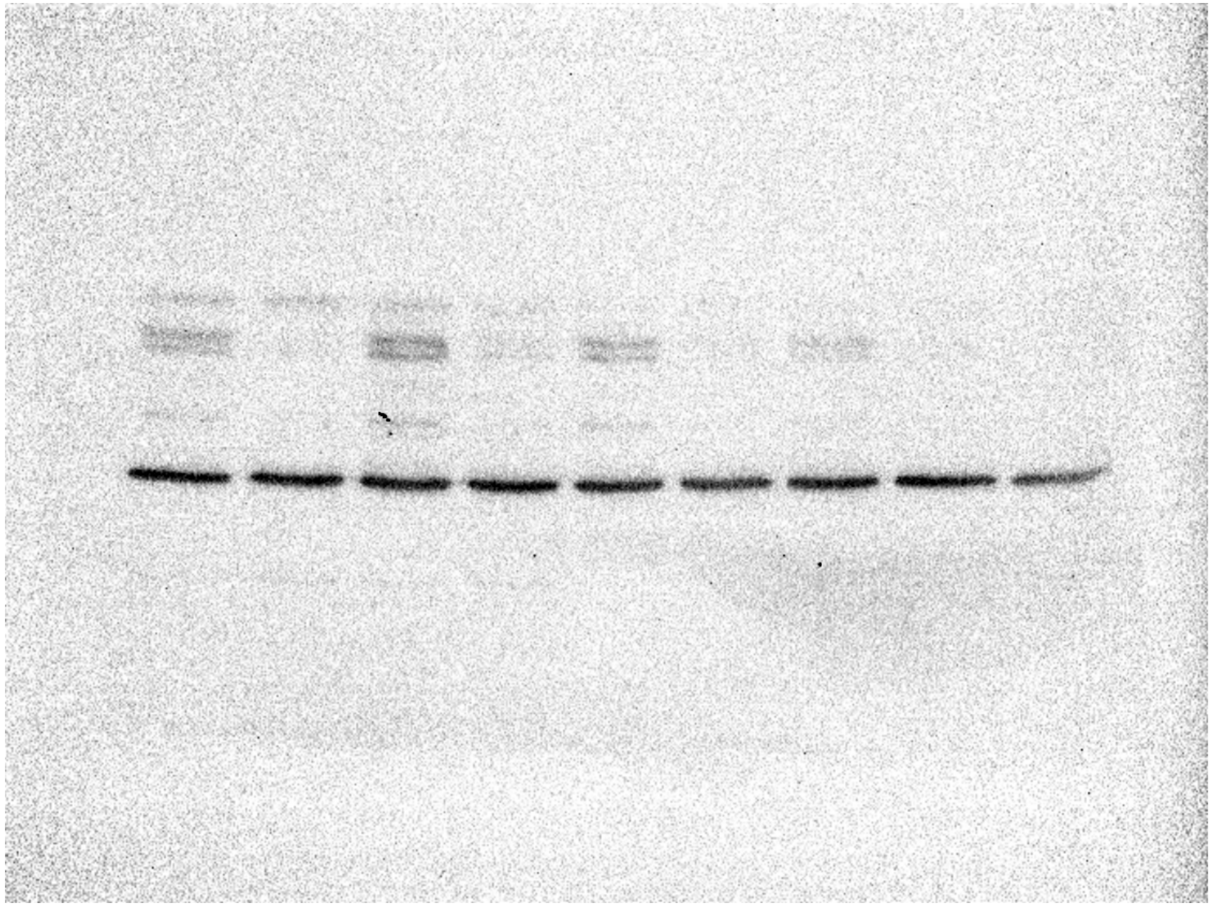

GAPDH
36 kDa
(Cell Signaling, 2118L, 1:2000)

Full unedited gel for Supplementary Figure 4B

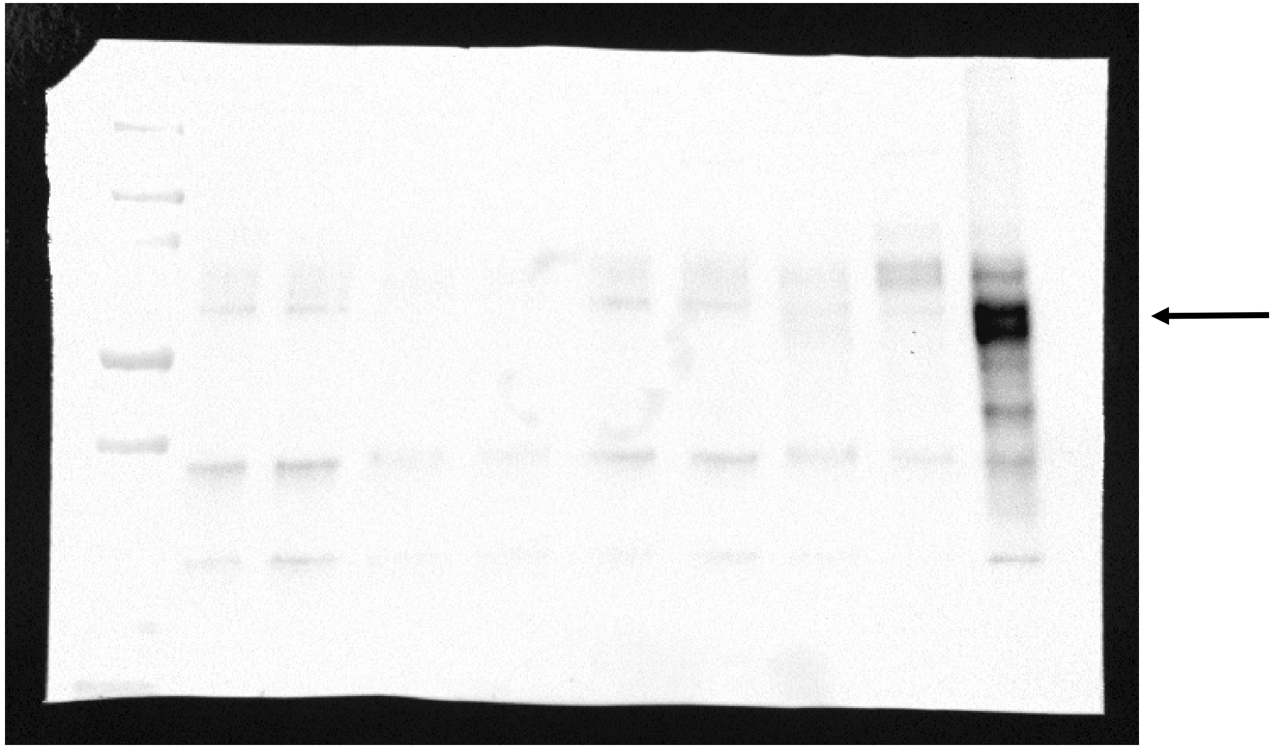

MITF
65 kDa
(Cell Signaling, 12590s, 1:1000)

Full unedited gel for Supplementary Figure 4B

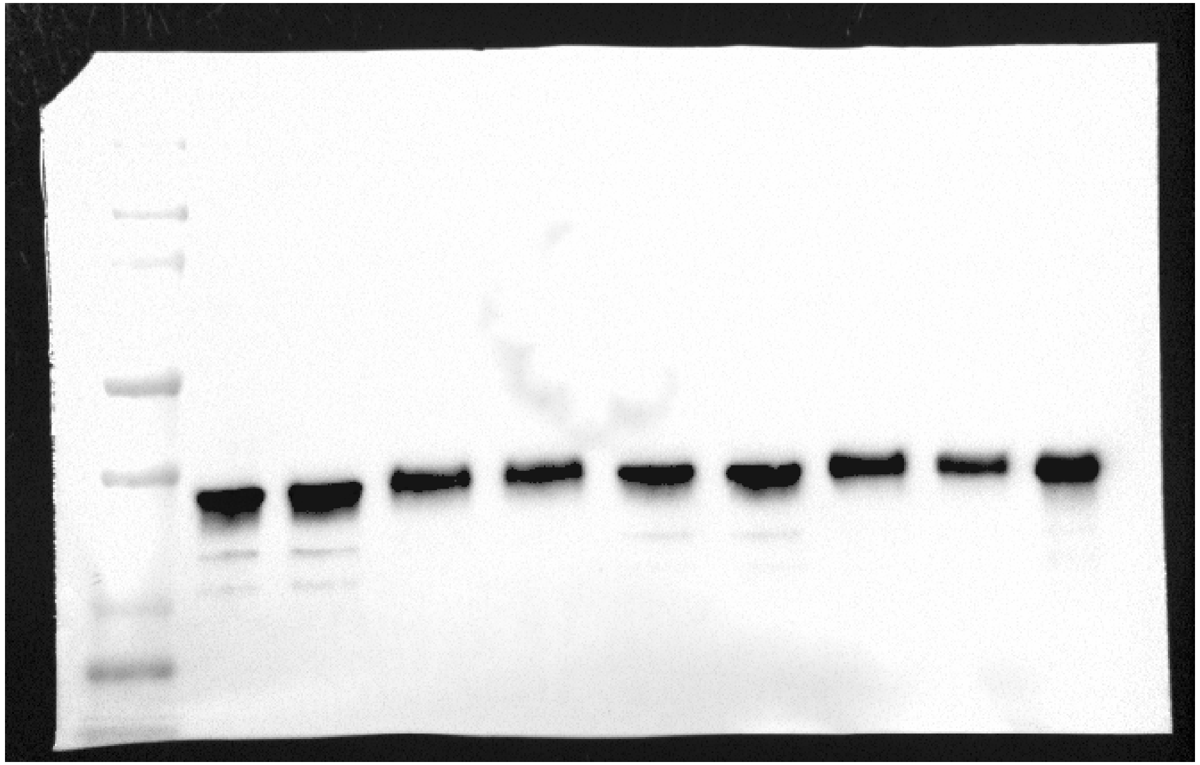

GAPDH
36 kDa
(Cell Signaling, 2118L, 1:2000)

Full unedited gel for Supplementary Figure 4B

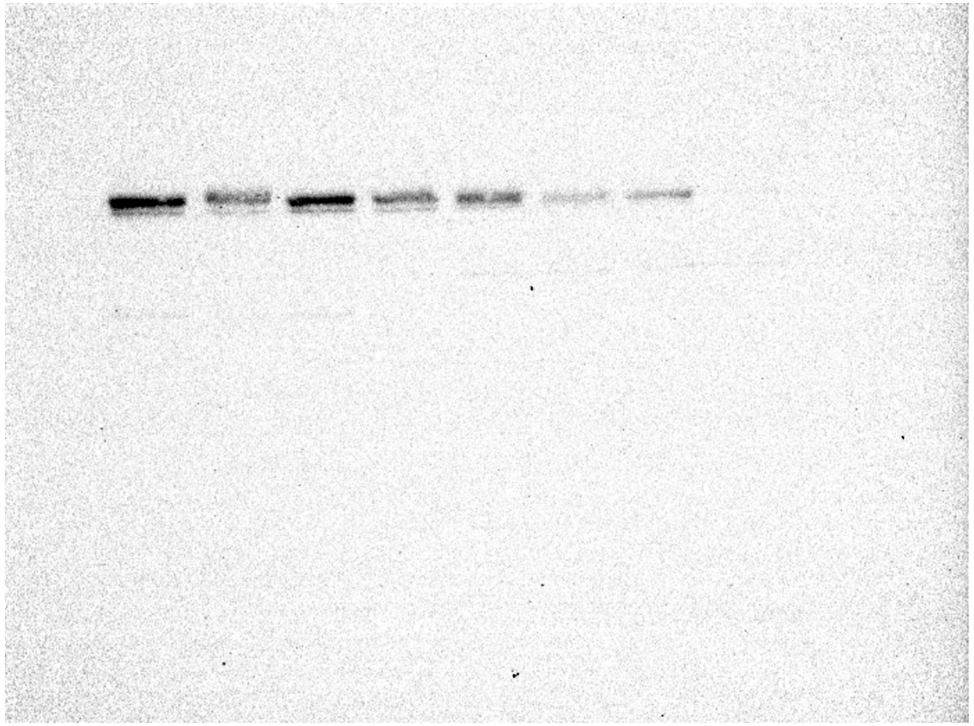

AXL
138 kDa
(Cell Signaling, 8661s, 1:1000)

Full unedited gel for Supplementary Figure 4B

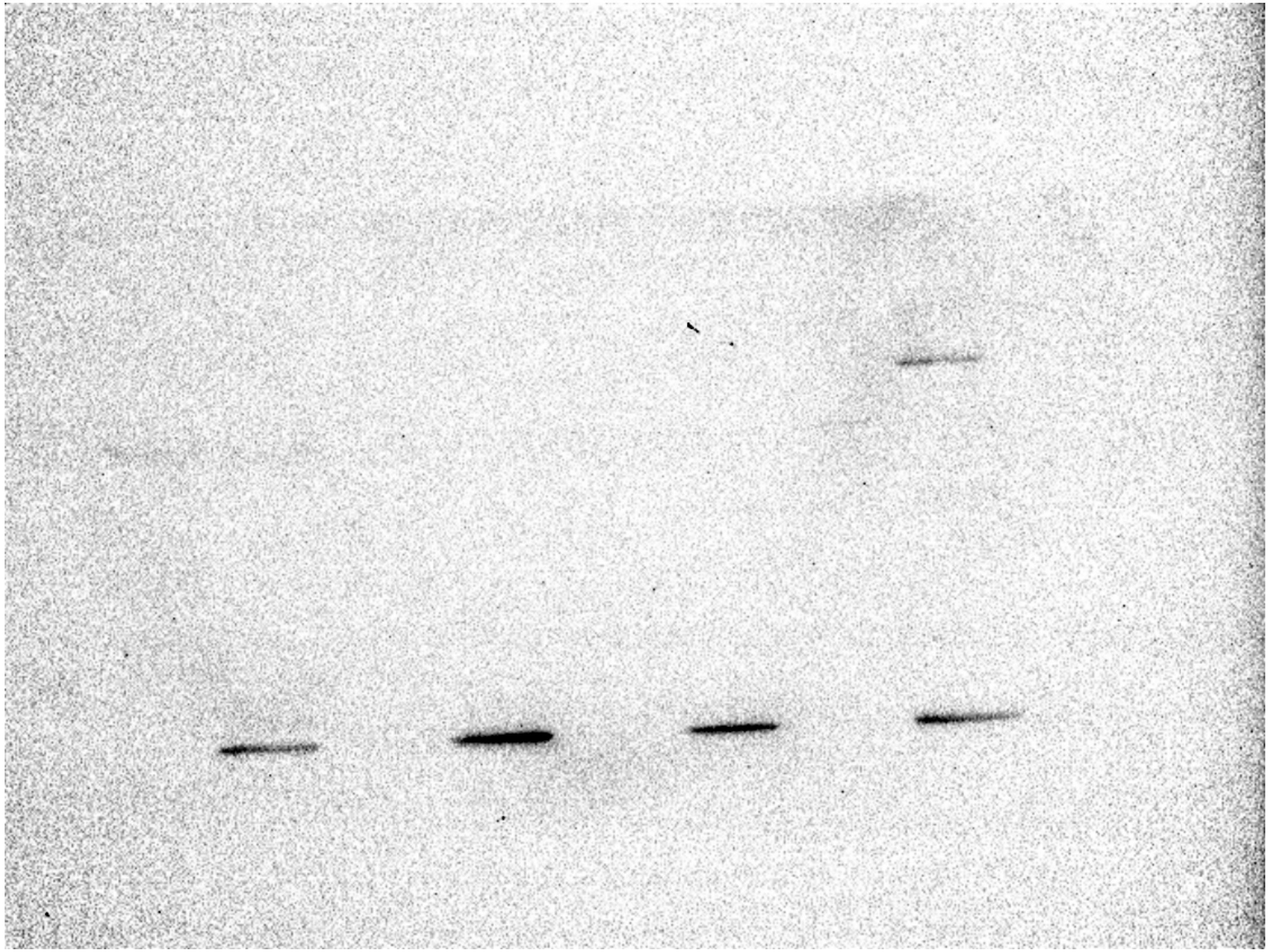

Cleaved PARP
89 kDa
(Cell Signaling, 9541s, 1:1000)

Full unedited gel for Supplementary Figure 4B

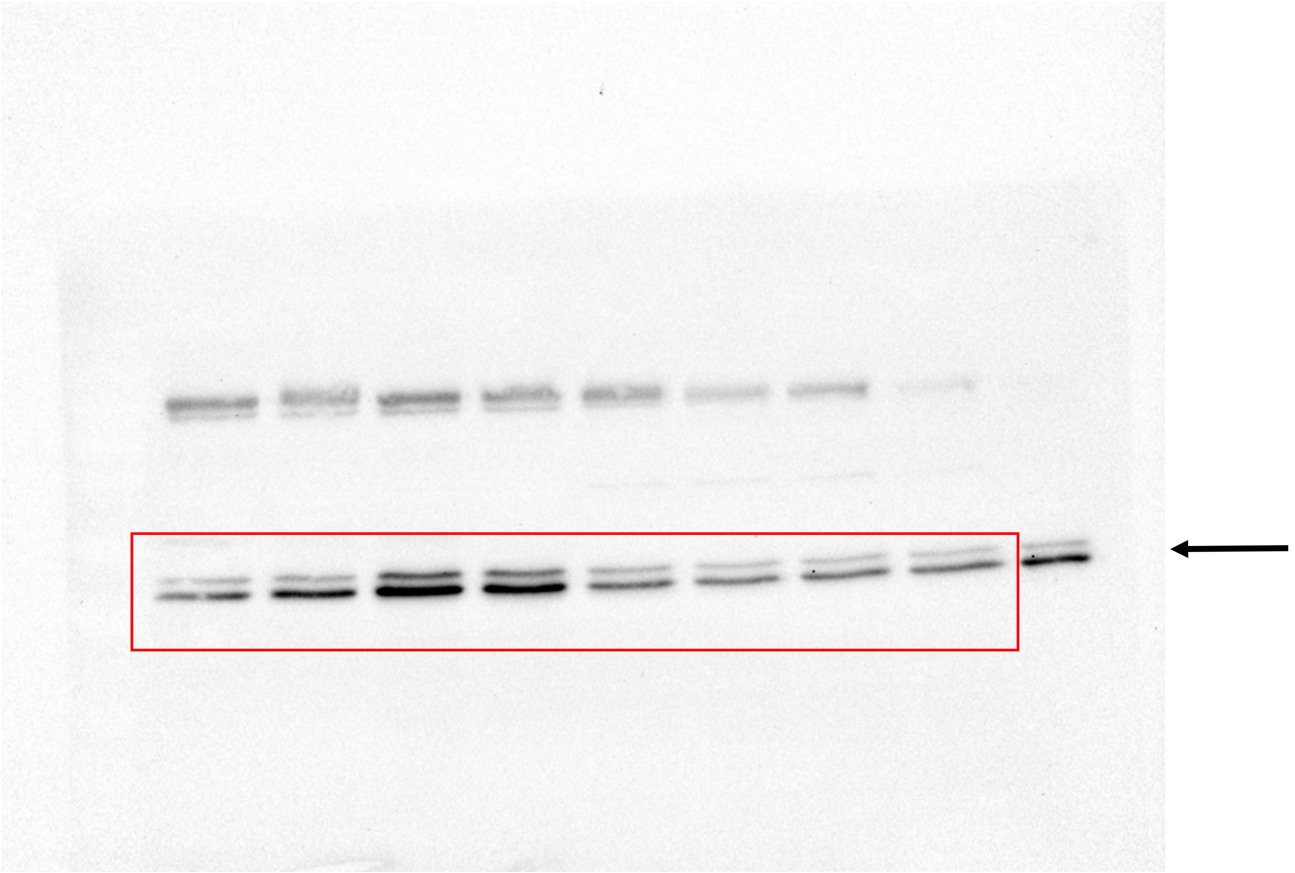

Supp Fig 4B

Full unedited gel for Supplementary Figure 4B

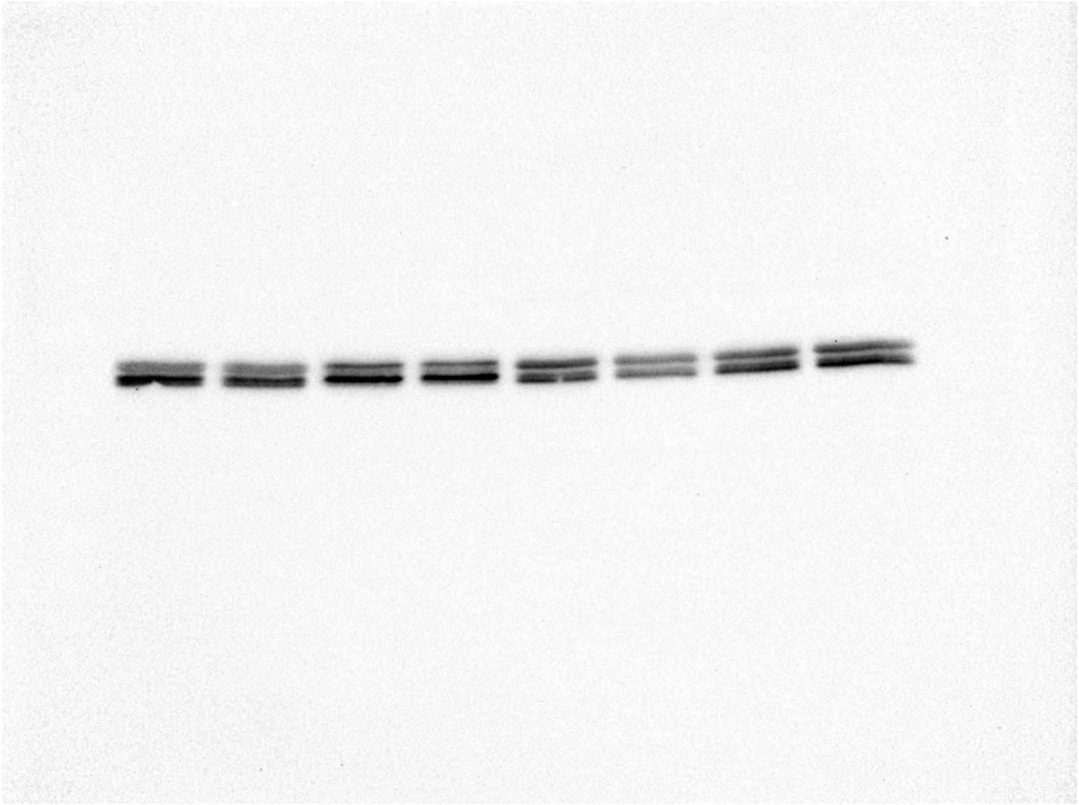

ERK – p44/42 MAPK
42,44 kDa
(Cell Signaling, 9102s, 1:1000)

Supp Fig 4B

Full unedited gel for Supplementary Figure 4B

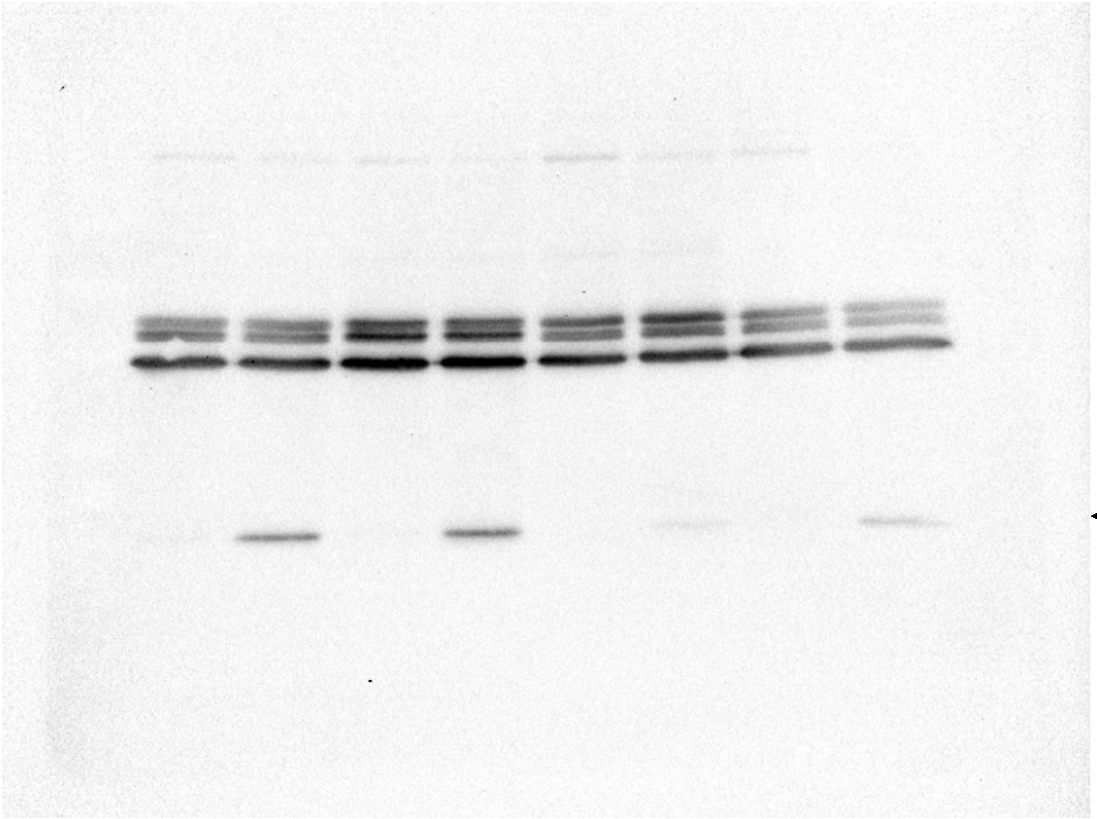

H3K4me2
18 kDa
(Abcam, ab32356, 1:5000)

Supp Fig 4B

Full unedited gel for Supplementary Figure 4B

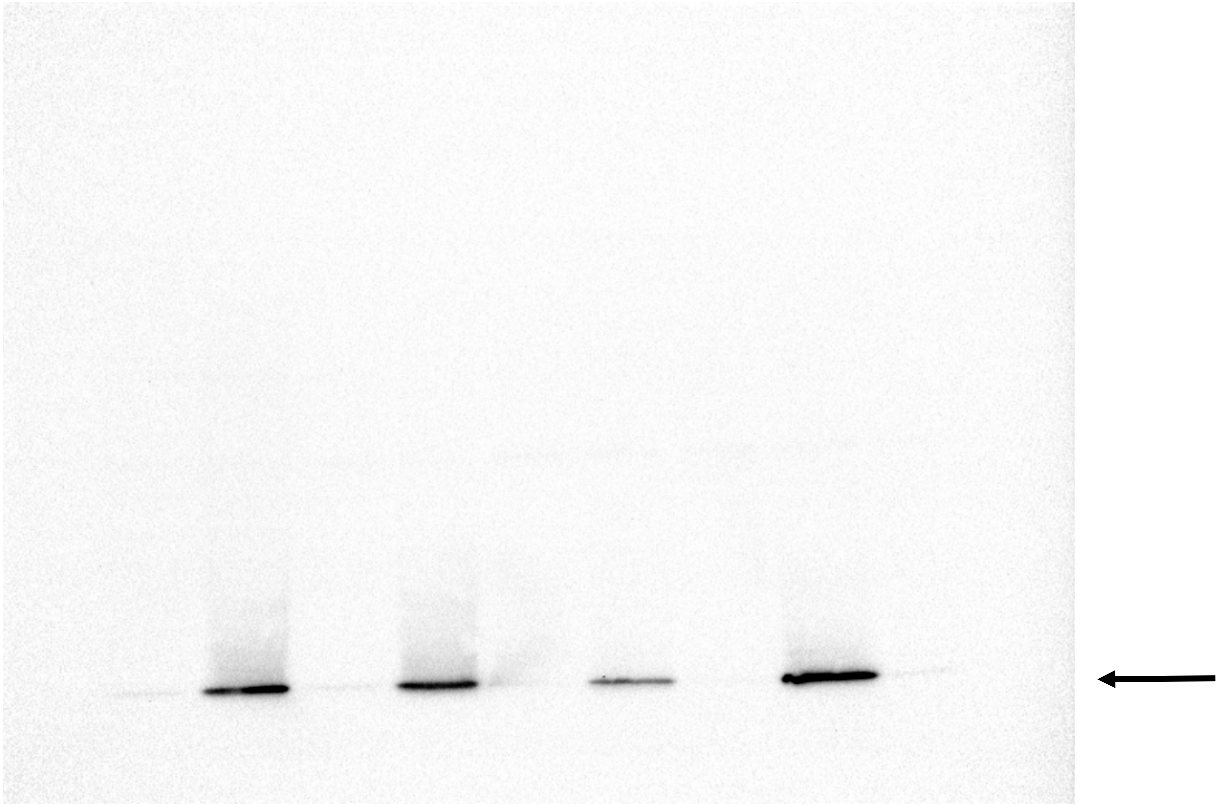

Supp Fig 4B

Full unedited gel for Supplementary Figure 4B

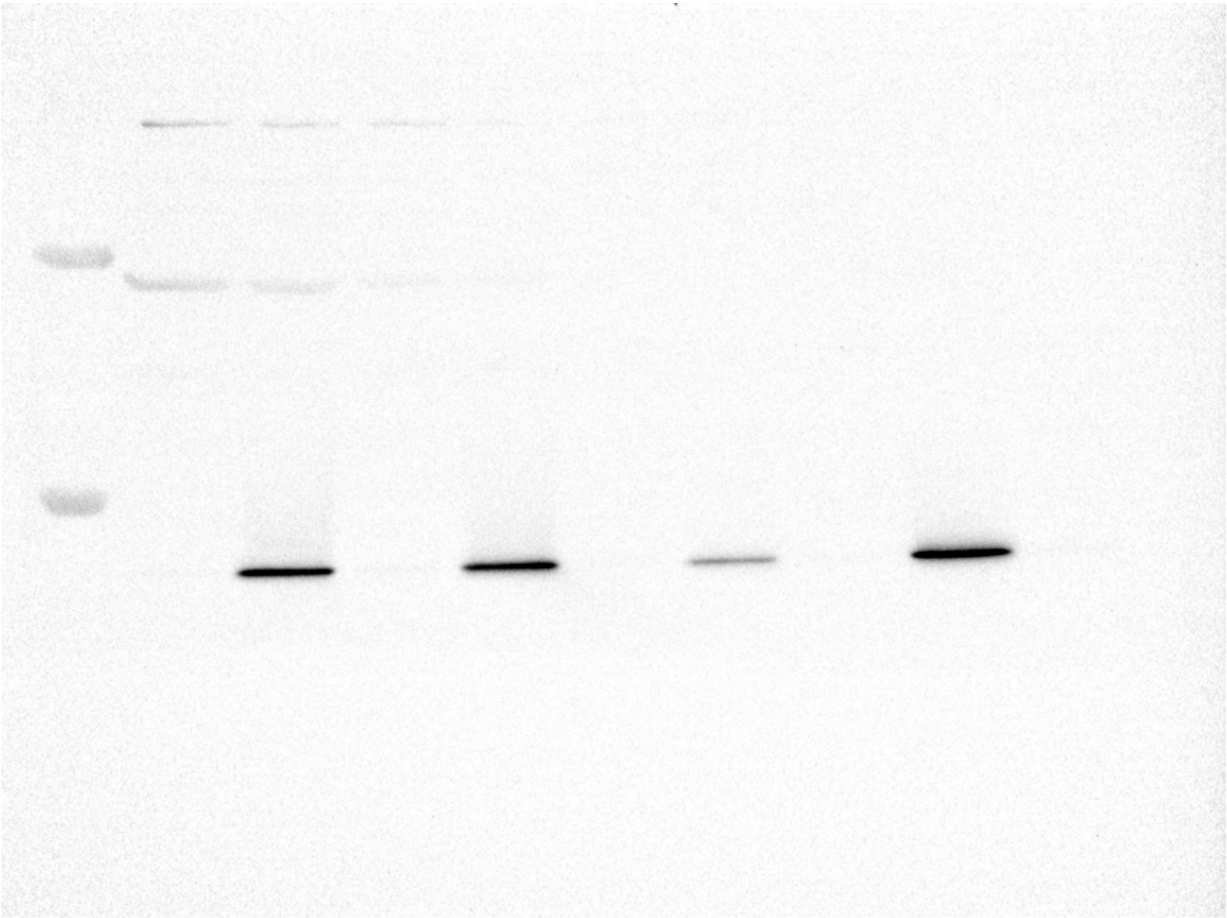

H3K27ac
15 kDa
(Abcam, ab4729, 1:1000)

Supp Fig 4B

Full unedited gel for Supplementary Figure 4B

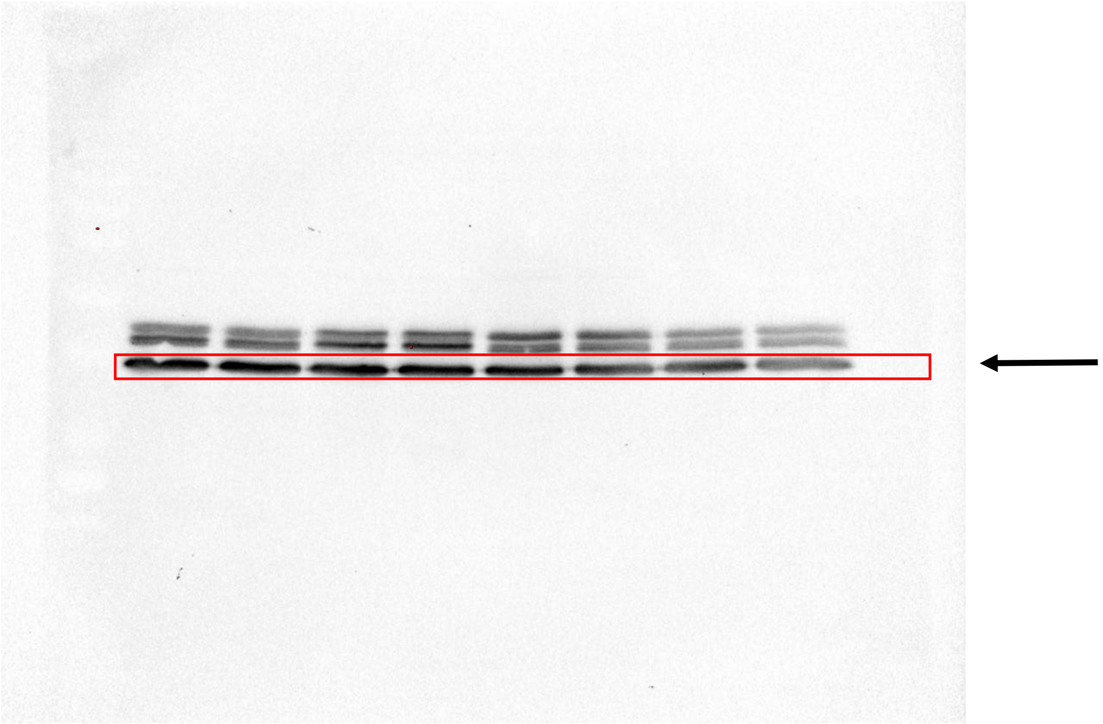

GAPDH
36 kDa
(Cell Signaling, 2118L, 1:2000)

Full unedited gel for Supplementary Figure 8B

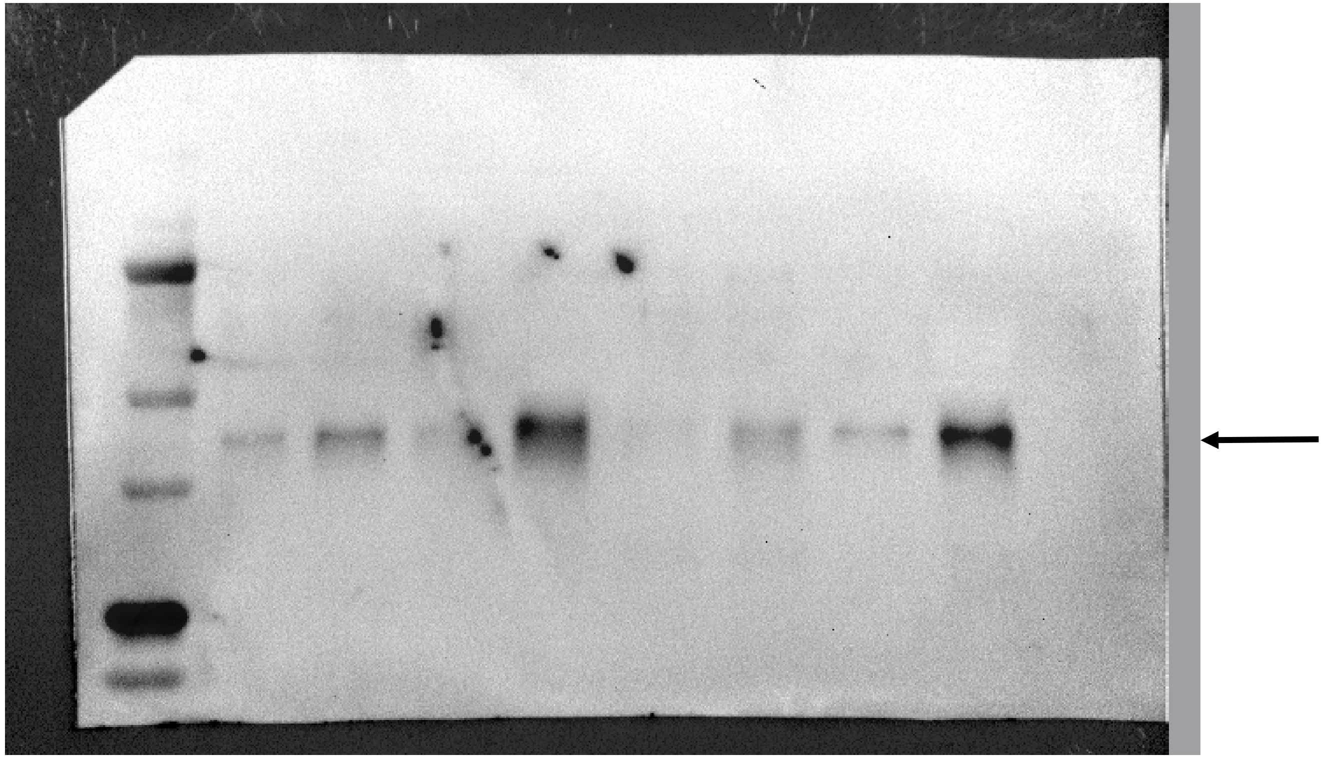

DUSP1
40 kDa
(Millipore, 07-535, 1:1000)

Full unedited gel for Supplementary Figure 8B

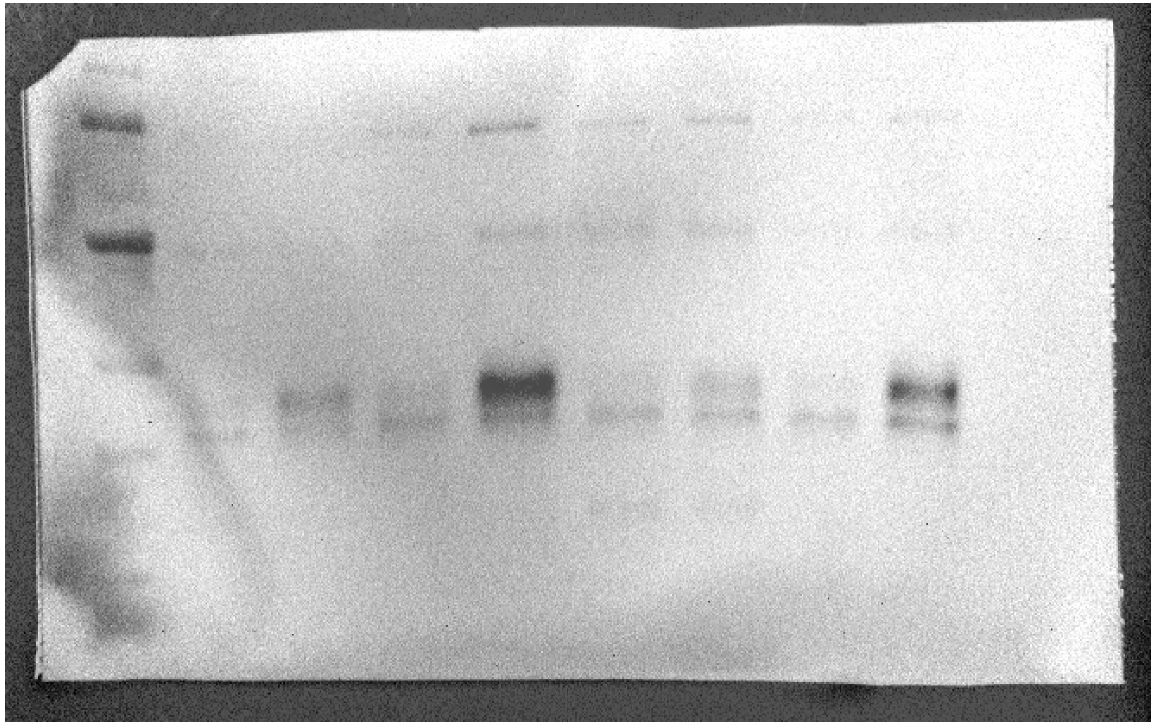

DUSP5
42 kDa
(Abcam, ab200708, 1:1000)

Sup Figure 8B

Full unedited gel for Supplementary Figure 8B

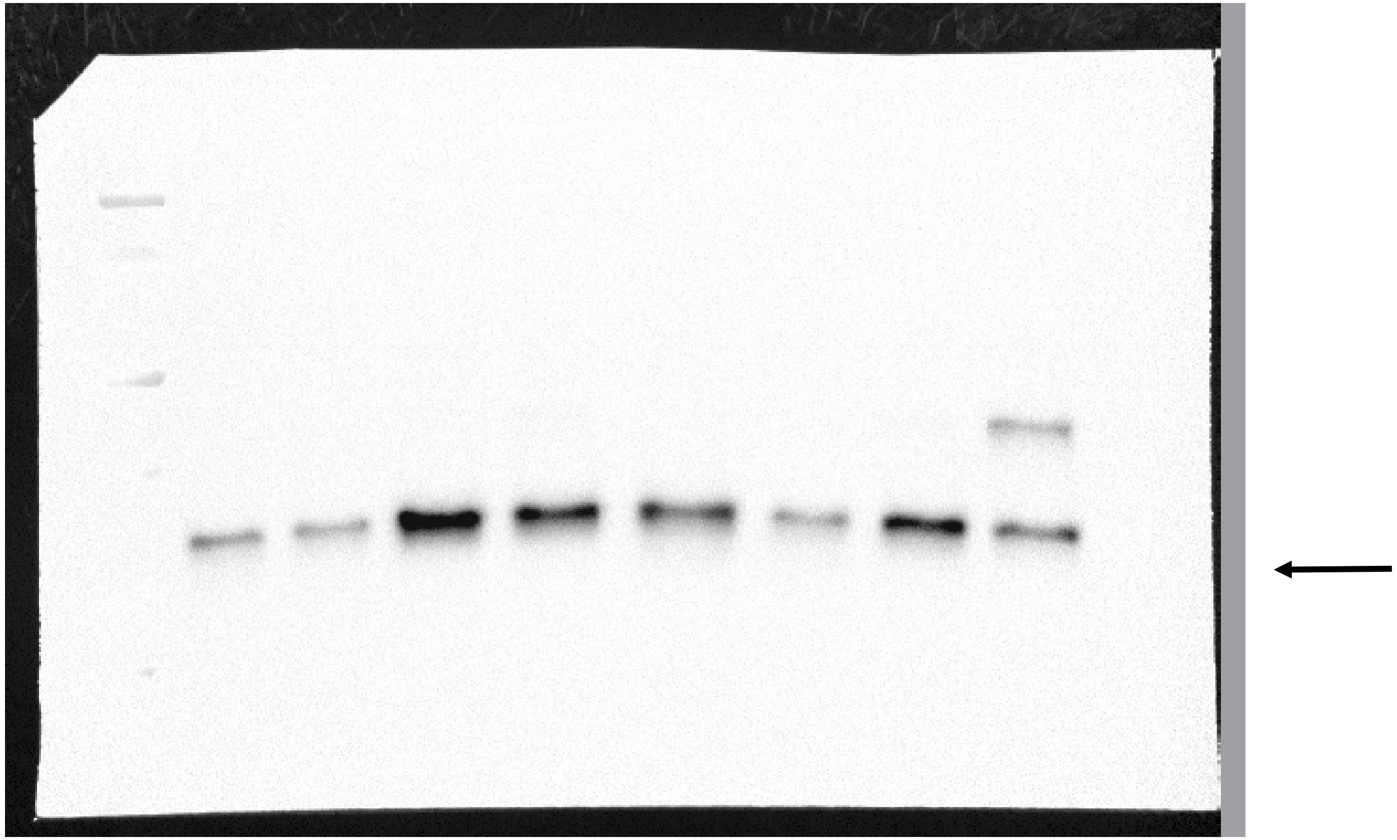

p-p38
43 kDa
(Cell Signaling, 4631s, 1:1000)

Sup Figure 8B

Full unedited gel for Supplementary Figure 8B

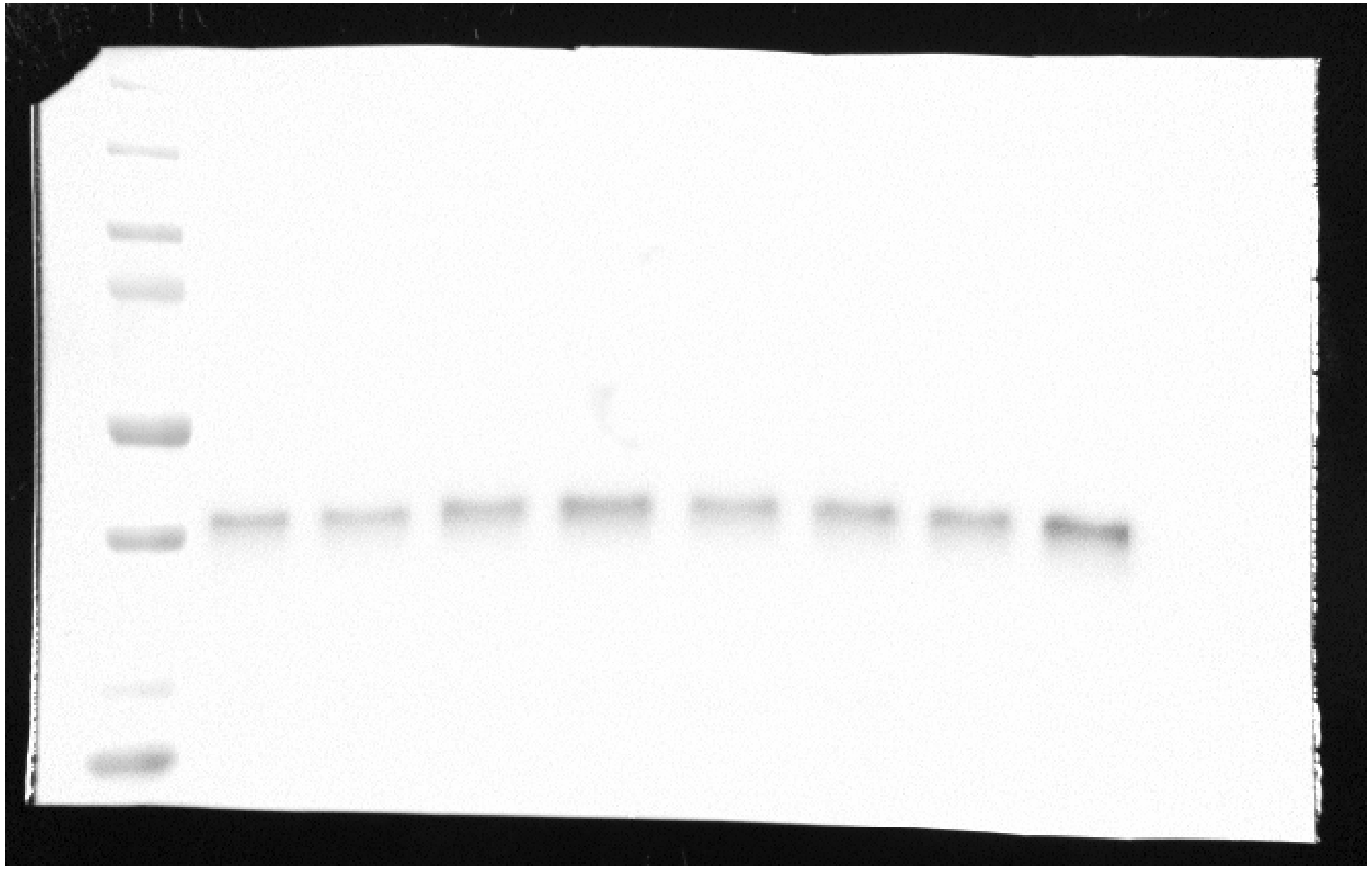

p38
40 kDa
(Cell Signaling, 9212s, 1:1000)

Full unedited gel for Supplementary Figure 8B

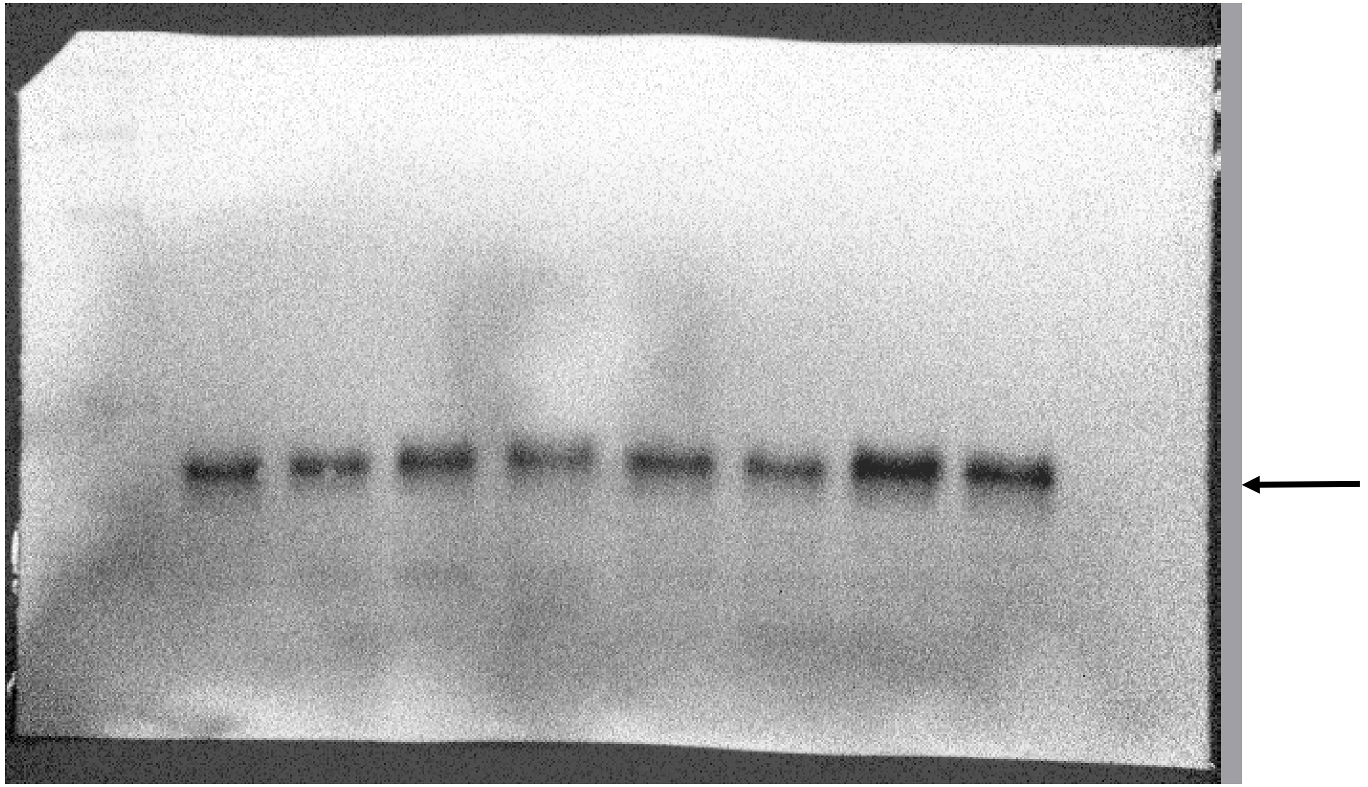

p-cJUN
40 kDa
(Cell Signaling, 9261s, 1:1000)

Sup Figure 8B

Full unedited gel for Supplementary Figure 8B

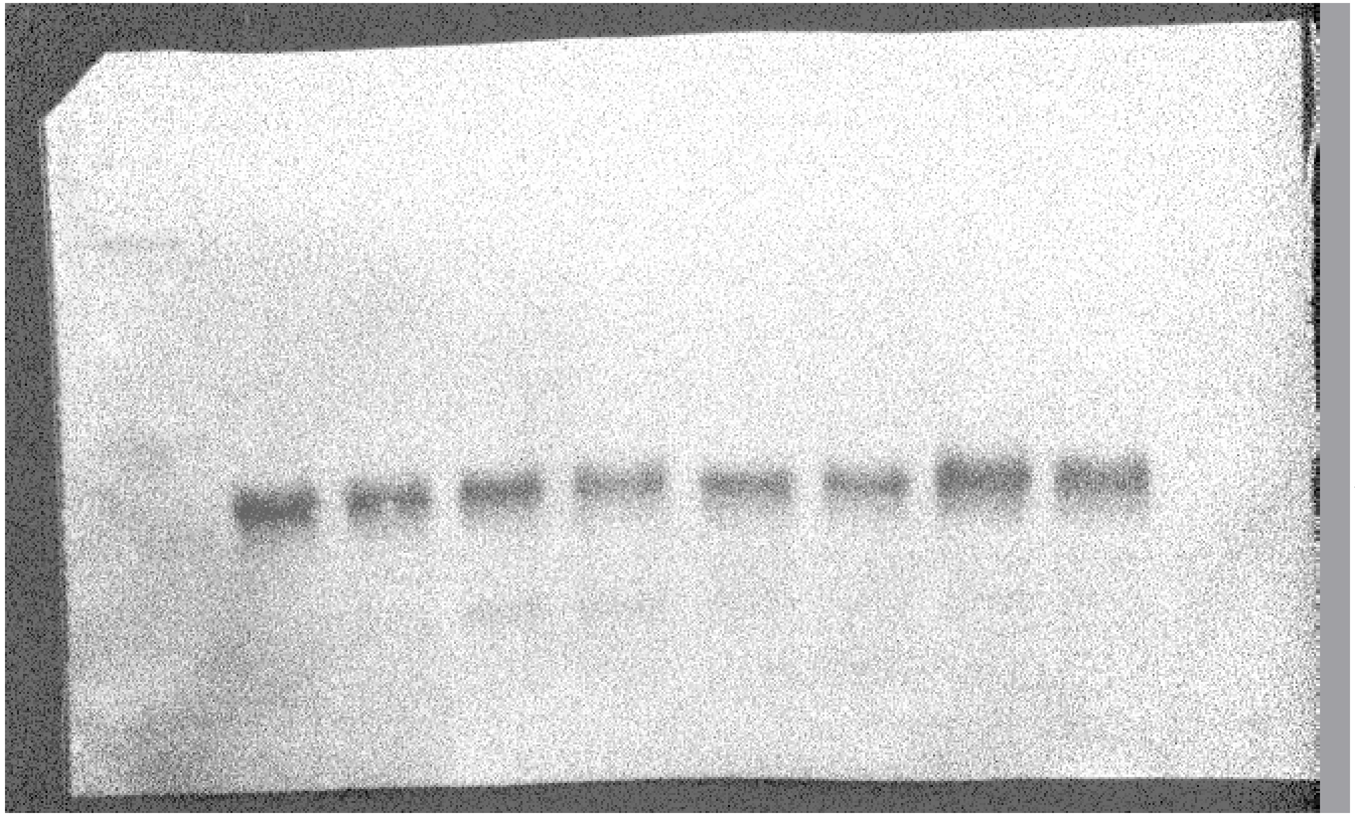

c-JUN
48 kDa
(Cell Signaling, 9165s, 1:1000)

Sup Figure 8B

Full unedited gel for Supplementary Figure 8B

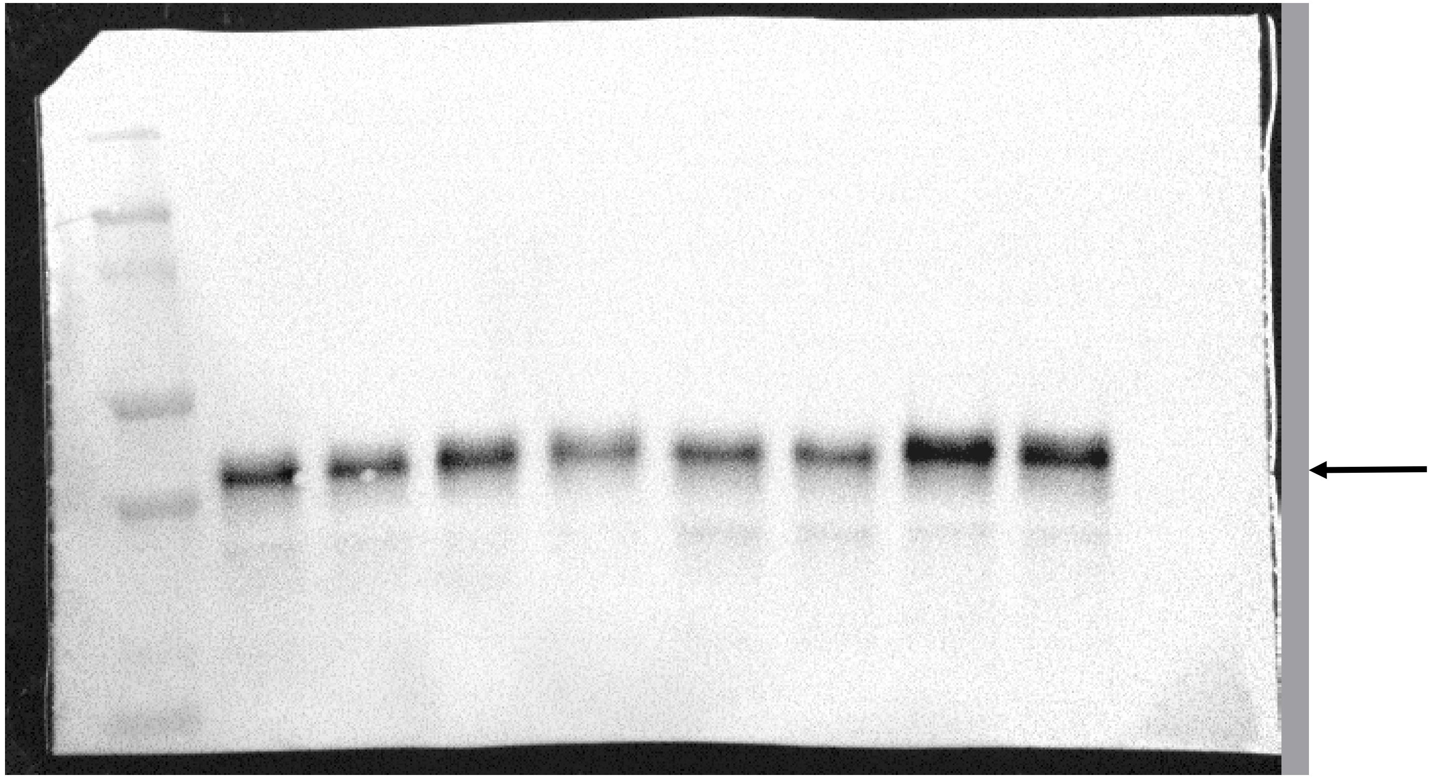

Sup Figure 8B

Full unedited gel for Supplementary Figure 8B

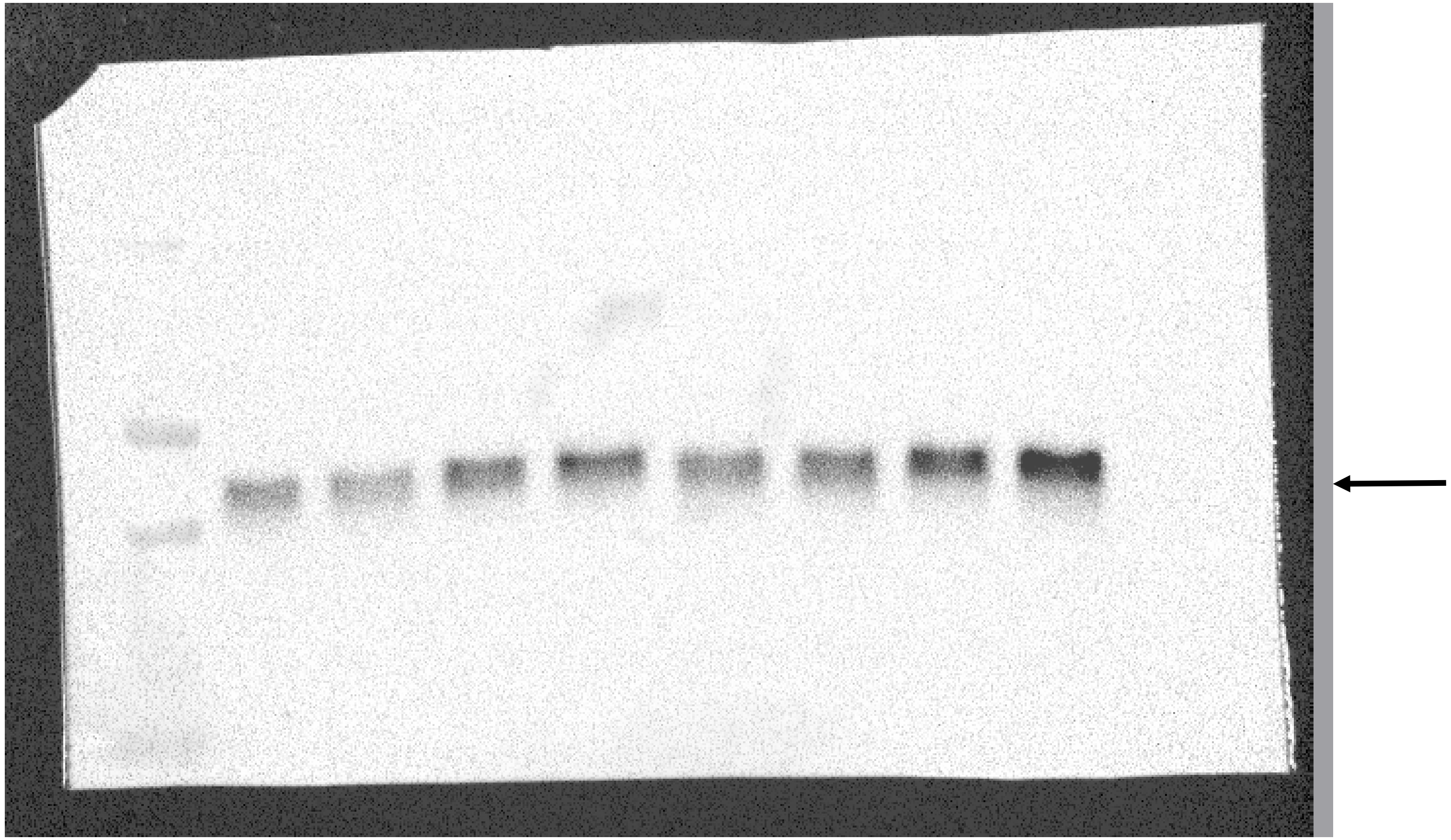

ERK – p44/42 MAPK
42,44 kDa
(Cell Signaling, 9102s, 1:1000)

Full unedited gel for Supplementary Figure 8B

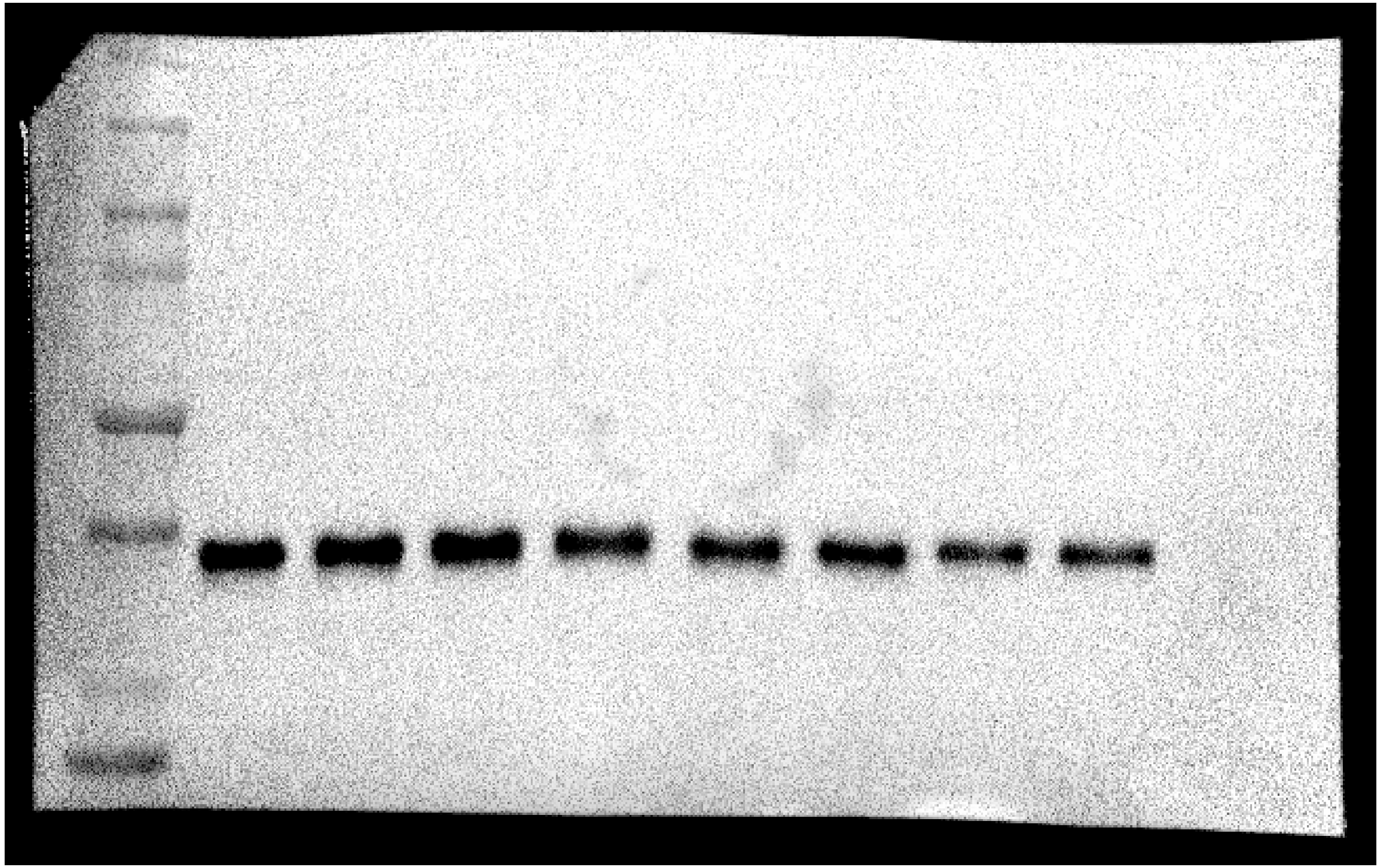

GAPDH
36 kDa
(Cell Signaling, 2118L, 1:2000)

Sup Figure 8B

Full unedited gel for Supplementary Figure 9D

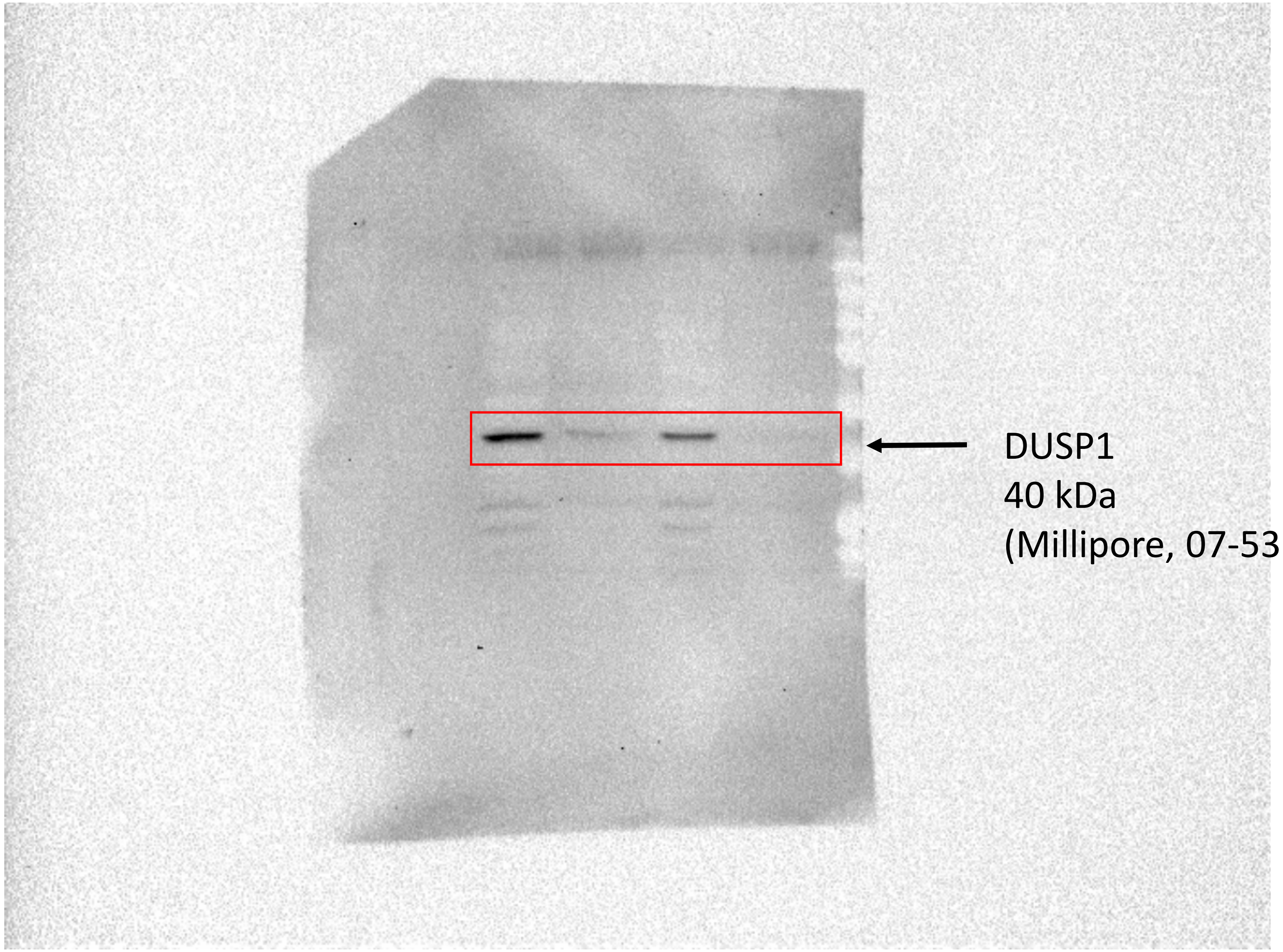

DUSP1
40 kDa
(Millipore, 07-535, 1:1000)

Supp Fig 9D

Full unedited gel for Supplementary Figure 9D

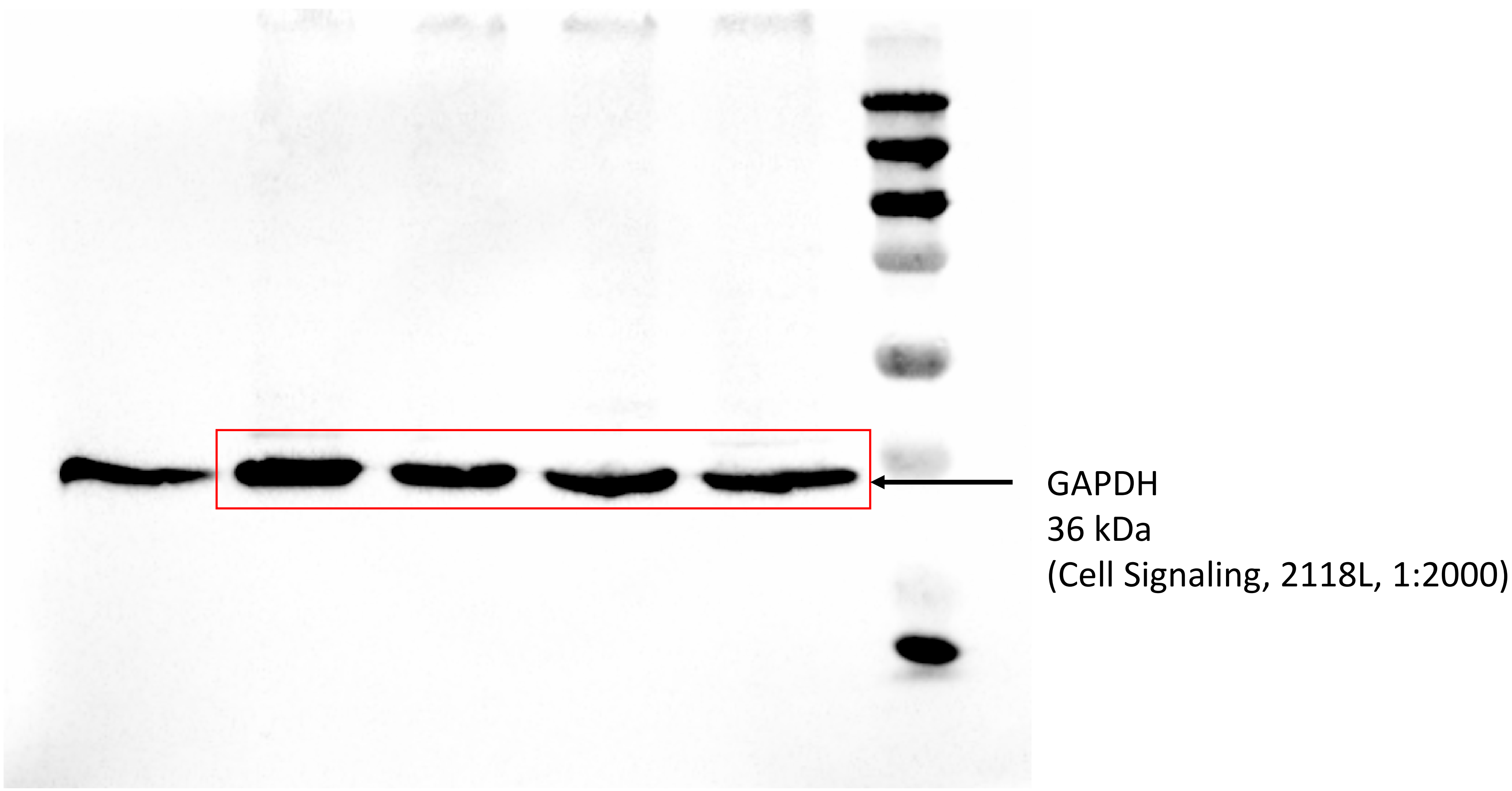

Supp Fig 9D

Full unedited gel for Supplementary Figure 9D

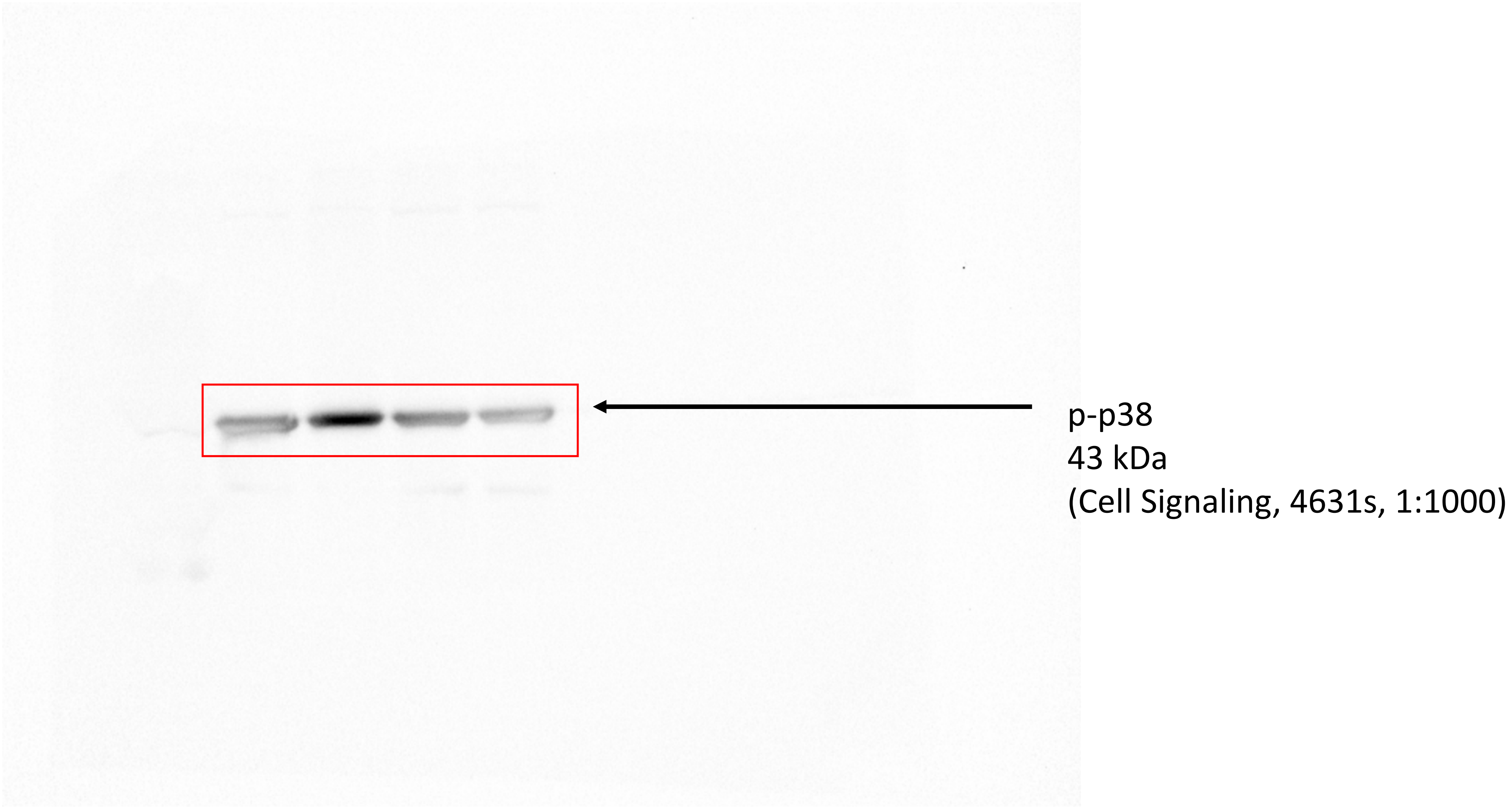

Supp Fig 9D

Full unedited gel for Supplementary Figure 9D

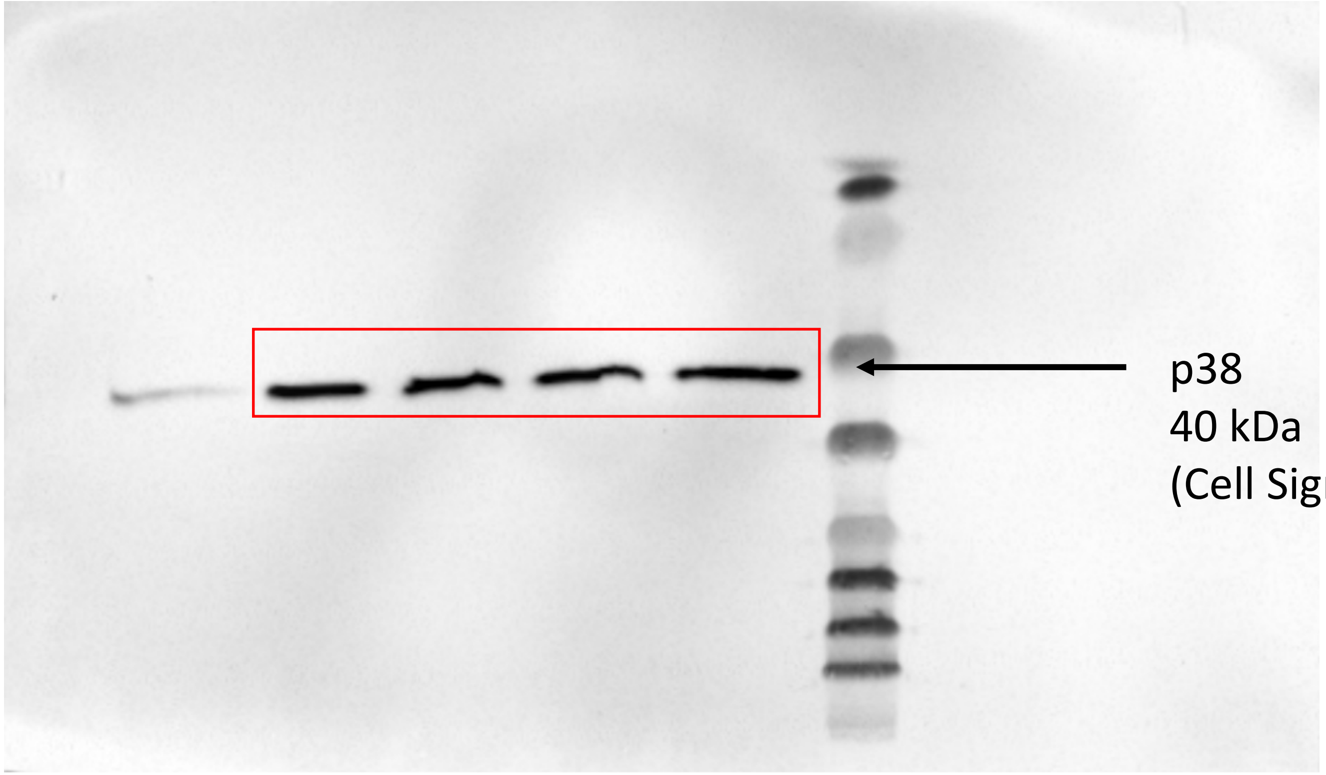

p38
40 kDa
(Cell Signaling, 9212s, 1:1000)

Supp Fig 9D
